# Non-decarbonylative photochemical *versus* thermal activation of Bu_4_N[Fe(CO)_3_(NO)] – the Fe-catalyzed Cloke–Wilson rearrangement of vinyl and arylcyclopropanes†
†Electronic supplementary information (ESI) available. CCDC 1409311–1409314. For ESI and crystallographic data in CIF or other electronic formats see DOI: 10.1039/c5sc02342d


**DOI:** 10.1039/c5sc02342d

**Published:** 2015-09-03

**Authors:** Che-Hung Lin, Dominik Pursley, Johannes E. M. N. Klein, Johannes Teske, Jennifer A. Allen, Fabian Rami, Andreas Köhn, Bernd Plietker

**Affiliations:** a Institut für Organische Chemie , Universität Stuttgart , Pfaffenwaldring 55 , DE-70569 Stuttgart , Germany . Email: bernd.plietker@oc.uni-stuttgart.de; b Mettler-Toledo GmbH , Ockerweg 3, D-35396 Giessen , Germany; c Institut für Theoretische Chemie , Universität Stuttgart , Pfaffenwaldring 55 , DE-70569 Stuttgart , Germany

## Abstract

The base metal complex Bu_4_N[Fe(CO)_3_(NO)] (TBA[Fe]) catalyzes the rearrangement of vinyl and arylcyclopropanes both under thermal or photochemical conditions to give the corresponding vinyl or aryldihydrofurans in good to excellent yields. Under photochemical conditions the reaction is performed at room temperature. Spectroscopic investigations show that the metal carbonyl catalyst is not decarbonylated. The best performance was observed at a wavelength of 415 nm. icMRCI+Q analysis of the excited singlet and triplet states of the [Fe(CO)_3_(NO)] anion was performed and used to calculate the vertical excitation energies which are in good agreement with the experimental data. CASSCF analysis indicates that the Fe center in all excited states of the ferrate becomes more electrophilic while adopting a distorted tetrahedral configuration. Both aspects have a positive synergistic effect on the formation of the initial π-complex with the incoming organic substrate.

## Abstract 2

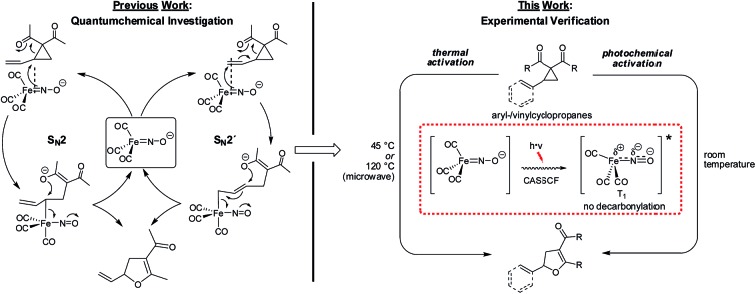
TBA[Fe] catalyzes the rearrangement of vinyl and arylcyclopropanes under thermal or photochemical conditions.

## Introduction

The photochemical activation of Fe complexes is ubiquitous in nature and has attracted significant research interest, in particular in the field of water splitting or proton reductions.^1^ In the field of non-biomimetic photocatalysis using iron–carbonyl complexes, landmark contributions from the groups of Beller^2^ and Darcel^3^ were most recently communicated. In these reports, Fe(CO)_5_^–^ or (NHC)Fe(CO)_4_^–^ catalysts (N-heterocyclic carbene) are activated through photochemical decarbonylation. Although quite common in organometallic chemistry, the exact mechanism of the photochemical activation-decarbonylation is not well understood. Important insights were gained through combined flash photolysis–ultrafast IR spectroscopy,^4^ ultrafast electron diffraction,^5^ and more recently femtosecond X-ray spectroscopy.^6^,^7^ From these data it was suggested that a decarbonylation of Fe(CO)_5_ results in the formation of an excited singlet Fe(CO)_4_, which can undergo intersystem crossing to the corresponding triplet Fe(CO)_4_. The analysis of spin-density indicated the decarbonylation to be a complex interplay of antibonding and bonding orbitals plus electron spin. In sharp contrast to the well-established photodecarbonylative activation of metal–carbonyl complexes, examples of a non-decarbonylative photochemical activation of a metal–carbonyl catalyst are not known in the literature.

**Fig. 1:**
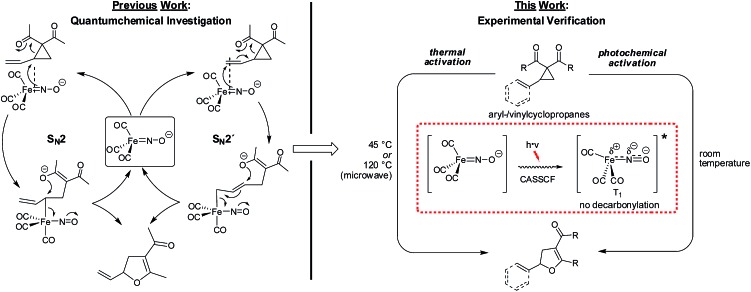
S_N_2- versus S_N_2-anti-mechanism in the TBA[Fe]-catalyzed Cloke-Wilson rearrangement of vinyl- and arylcyclopropanes.^13^,^14^

For a number of years our group has been interested in base metal catalysis using the stable and readily accessible electron-rich complex Bu_4_N[Fe(CO)_3_(NO)] (TBA[Fe]).^8^ Aiming to understand the interplay between electronic ground state structure and reactivity, a combined in-depth spectroscopic and experimental study indicated that the metal center should be regarded as being zero-valent while the negative charge is located at the NO-ligand. Importantly, CASSCF- and IBO-analysis independently showed that the Fe–NO-moiety should be considered as a singlet ground-state complex with a triplet Fe(0) atom being antiferromagnetically coupled to a triplet NO-anion *via* two non-polar covalent Fe–N(d,p)-π-bonds.^9^ In a subsequent theoretical investigation of the mechanism of the TBA[Fe]-catalyzed Cloke–Wilson rearrangement,^10^ a reaction that was observed during our studies on the Fe-catalyzed activation of allyl C–C bonds,^11^ it was found that only one out the two Fe–N–π-bonds is involved in cleaving allylic activated atom bonds *via* a ligand-centered two-electron oxidation.^12^ The oxidation state of the metal center remains as zero. Moreover, the theoretical results indicated that the Fe-catalyzed activation of a vinylcyclopropane could follow both an S_N_2′-but also an S_N_2-pathway (Fig. 1).

With regard to these previous findings we initiated a research project to investigate whether

• The ferrate complex in its unusual electronic ground-state could be activated for catalytic transformations upon irradiation with light.

• The ferrate complex remains intact or undergoes a decarbonylation upon irradiation with UV-light.

## Results and discussion

The Fe-catalyzed Cloke–Wilson rearrangement was found as a major side-reaction during our studies on Fe-catalyzed allylic C–C bond activations.^11^ At the outset of our studies the reaction was systematically optimized (Table 1). As already observed during our investigations on carbene-transfer catalysis the addition of ligands led to a significant decrease of the conversion. For the thermal reaction the use of only 1 mol% of TBA[Fe] in dichloromethane as the solvent at a temperature of 45 °C resulted in a quantitative conversion of vinylcyclopropane **1** (entry 6, Table 1). In sharp contrast the same reaction albeit at room temperature showed no conversion (entry 7, Table 1). With this reference for the thermal activation in hand we set out to investigate the influence of photochemical activation. Indeed, irradiation of the reaction mixture using a 180 W Hg-lamp at room temperature in dichloromethane gave the desired product in low yield (entry 8, Table 1). Changing the solvent to the more polar acetonitrile and increasing the catalyst loadings to 2.5 mol% resulted in a clean dihydrofuran formation at room temperature (entry 11, Table 1). Importantly, the reaction did not proceed without irradiation under otherwise identical reaction conditions.

**Table 1:** Optimization of the Cloke–Wilson rearrangement

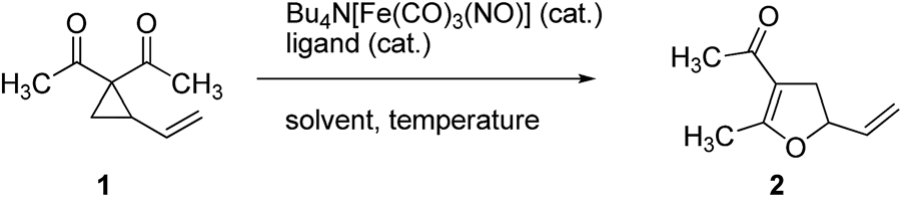						
Entry	Cat. [mol%]	Solvent	Ligand	*T* [°C]	Light	Conv.^*b*^ [%]
1^*a*^	2.5	THF	SIMes	50	—	72
2^*a*^	2.5	THF	PPh_3_	50	—	6
3^*a*^	2.5	Benzene	PPh_3_	50	—	<5
4^*a*^	2.5	CH_2_Cl_2_	PPh_3_	50	—	7
5^*a*^	2.5	CH_2_Cl_2_	—	50	—	>98
6^*a*^	1	CH_2_Cl_2_	—	45	—	>98
7^*a*^	1	CH_2_Cl_2_	—	20	—	—
8^*a*^	1	CH_2_Cl_2_	—	20	180 W (Hg)	8
9^*a*^	1	CH_3_CN	—	20	180 W (Hg)	16
10^*c*^	2.5	CH_2_Cl_2_	—	20	180 W (Hg)	85
11^*c*^	2.5	CH_3_CN	—	20	180 W (Hg)	96^*d*^
12^*c*^	2.5	CH_3_CN	—	R.T.	75 W (Xe)	93
13^*c*^	2.5	CH_3_CN	—	R.T.	23 W	92^*d*^

^*a*^14 h.

^*b*^Determined using NMR-integration using mesitylene as the internal standard.

^*c*^3 h.

^*d*^Isolated yield.

To exclude the disturbing heating effect caused by the strong UV-lamp an eventual temperature–time correlation of the reaction mixture was analyzed using in-operando-IR spectroscopy/thermometry.^15^ Starting at room temperature (20 °C) an increase of the temperature to about 28 °C was recorded, however this temperature increase correlates with the conversion rate and was attributed to the exothermicity of the reaction. Importantly, the Cloke–Wilson rearrangement did not take place at 28 °C without irradiation. Knowing that the rearrangement proceeds under photochemical conditions using a 180 W Hg-lamp we were wondering whether less-intense UV-light or even visible light irradiation could activate the complex. We were pleased to find that the use of a 75 W Xe-UV-lamp (entry 12, Table 1) and even a commercial 23 W household lamp led to the formation of dihydrofuran **2** in excellent isolated yields (entry 13, Table 1).

With these encouraging results in hand we subsequently set out to compare the reaction scope of the thermal *versus* the photochemical Fe-catalyzed Cloke–Wilson rearrangement (Table 2).

**Table 2:** Scope of the thermal *versus* photochemical Fe-catalyzed Cloke–Wilson rearrangement of vinylcyclopropanes

Entry	Substrate	Product	Cond.^*a*^	Yield^*b*^ [%]	Entry	Substrate	Product	Cond.^*a*^	Yield^*b*^ [%]
1	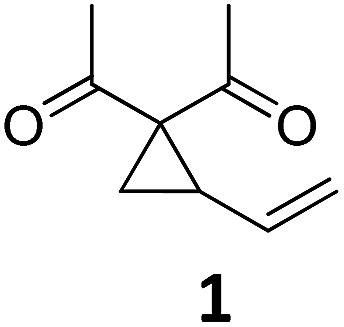	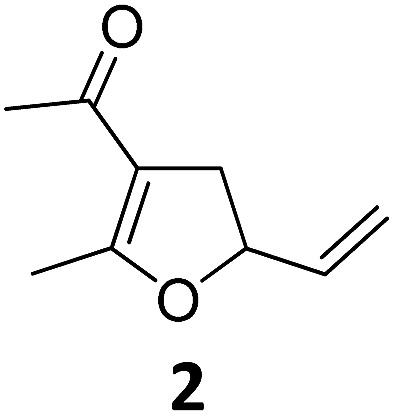	A	94	17^*c*^	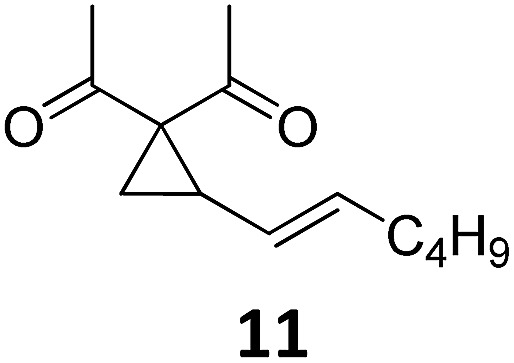	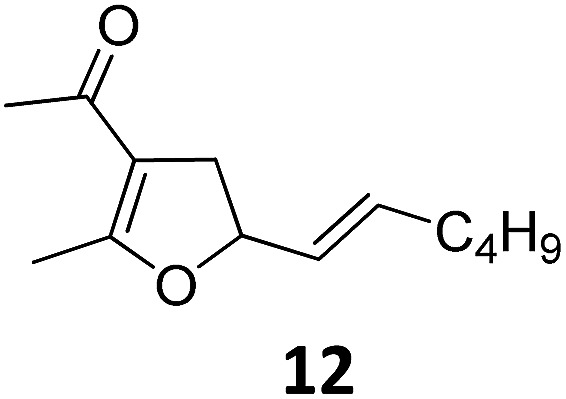	A	87
2			B	96, 93^*e*^, 92^*f*^	18^*c*^ ^,^^*g*^			B	87
3	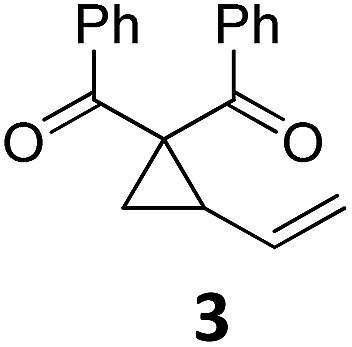	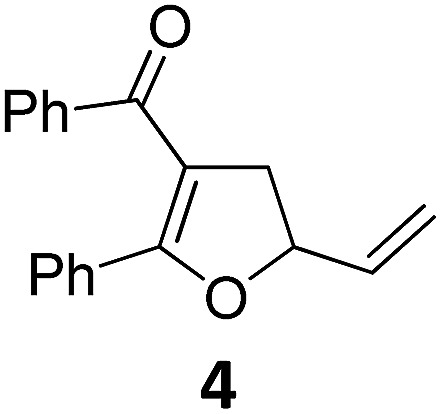	A	92	19^*c*^	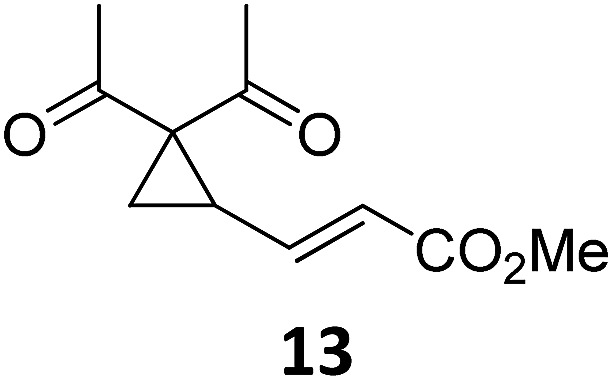	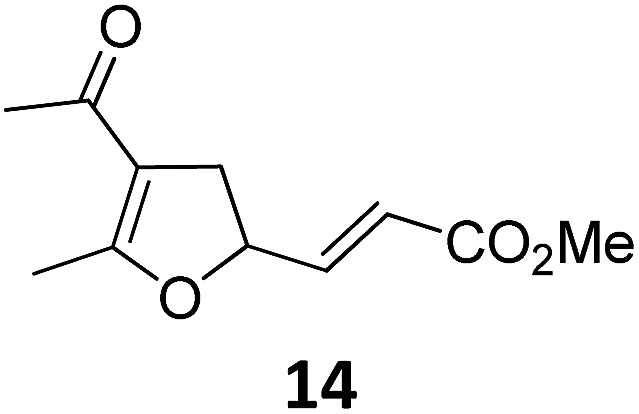	A	96
4			B	93	20^*c*^ ^,^^*g*^			B	89
5	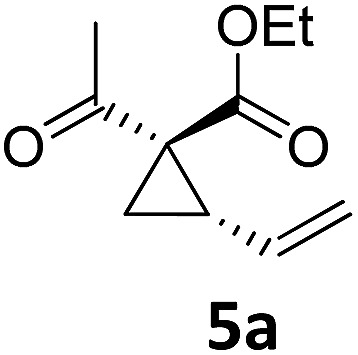	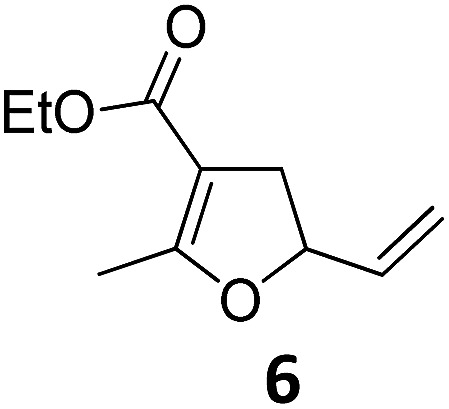	A	93	21^*c*^	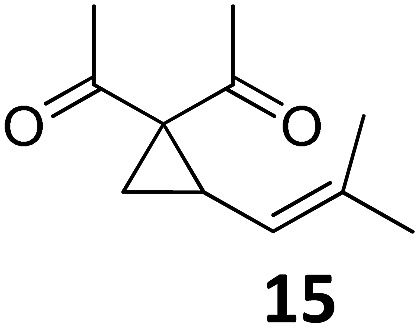	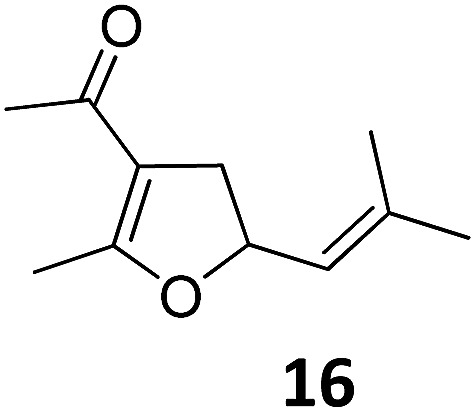	A	54
6			B	94, 93^*f*^	22^*c*^ ^,^^*g*^			B	75, 76^*f*^
7	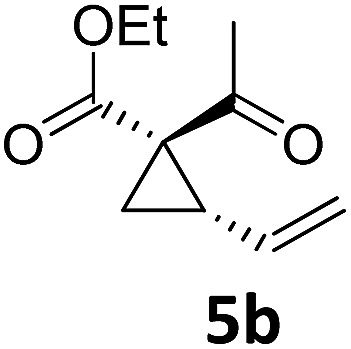		A	92	23^*c*^	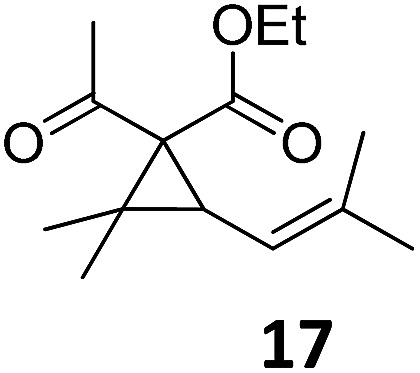	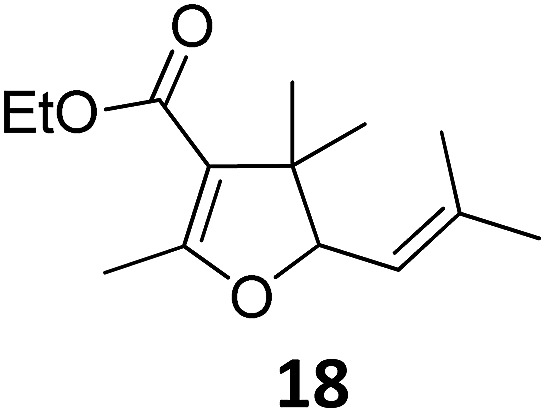	A	—
8			B	92, 92^*f*^	24^*d*^			B	98, 96^*e*^, 19^*f*^
9	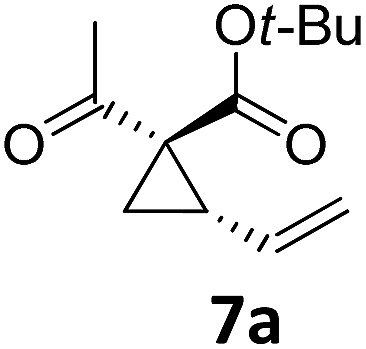	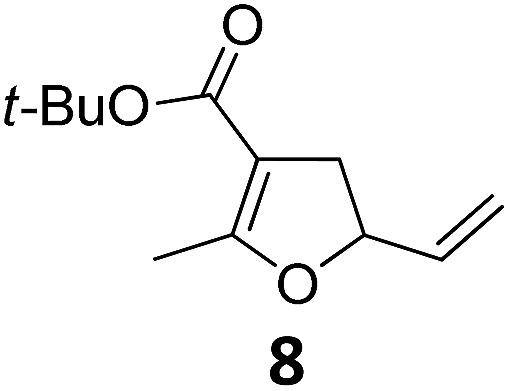	A	94	25^*c*^	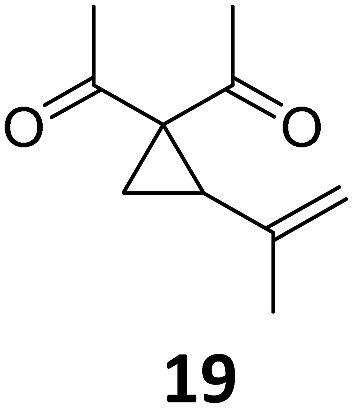	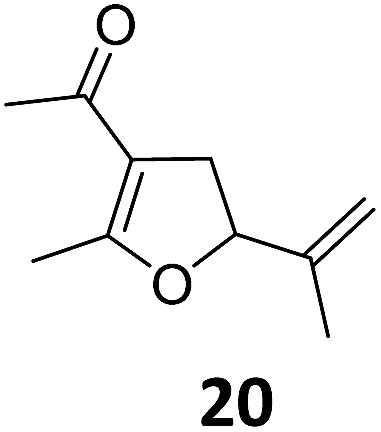	A	96
10			B	85	26			B	97
11	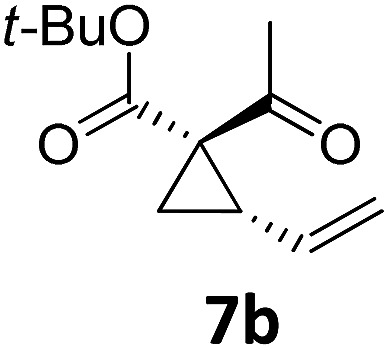		A	60	27^*c*^	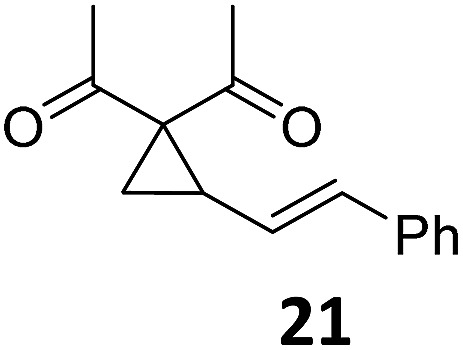	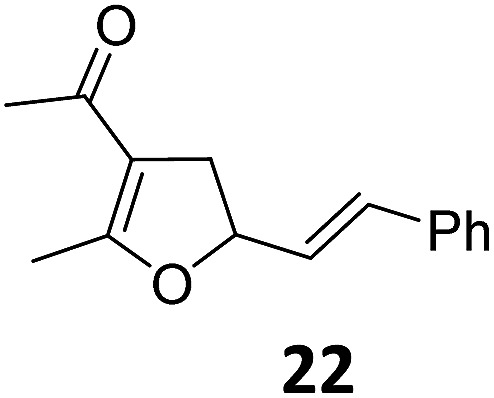	A	98
12			B	91	28^*c*^ ^,^^*g*^			B	98
13^*c*^	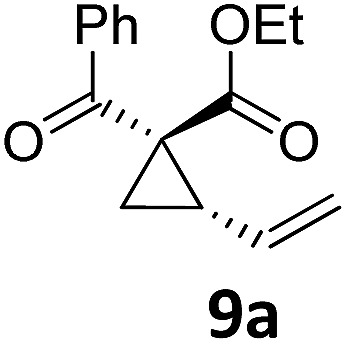	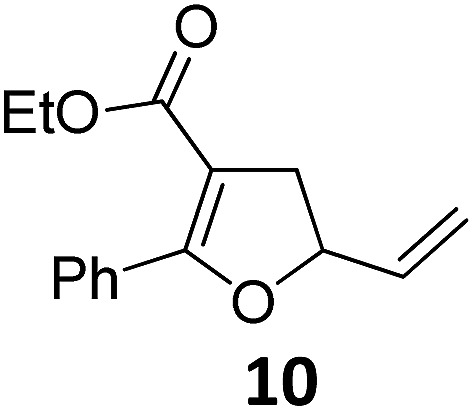	A	87	29	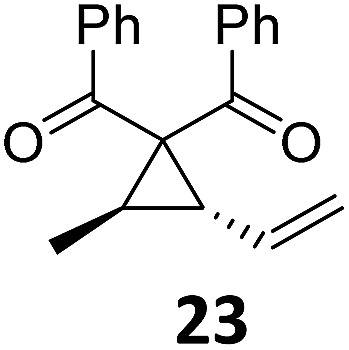	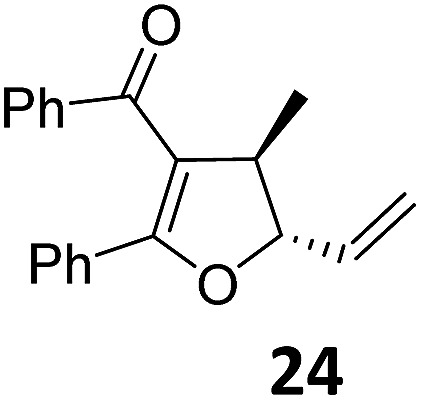	A	72
14			B	91	30			B	92
15	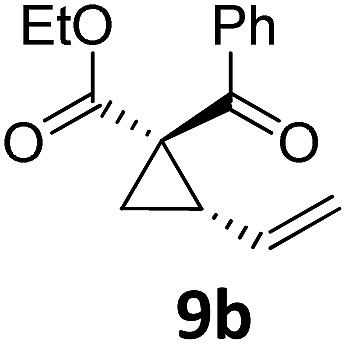		A	86					
16			B	92					

^*a*^Conditions A: 0.5 mmol of substrate, 1 mol% of TBA[Fe], 1 mL (abs.) of CH_2_Cl_2_, 45 °C, 16 h; conditions B: 0.4 mmol of substrate, 2.5 mol% of TBA[Fe], 1 mL (abs.) of CH_3_CN, 180 W (Hg-lamp), 20 °C, 3 h.

^*b*^Isolated yields.

^*c*^5 mol% of TBA[Fe].

^*d*^10 mol% of TBA[Fe] in 1 mL (abs.) of THF, 24 h.

^*e*^75 W (Xe-lamp).

^*f*^23 W compact fluorescence lamp.

^*g*^6 h.

Both protocols proved to be broadly applicable, however certain differences indicate that they do have complementary advantages/disadvantages. Stereocenters within the substrate do have a profound effect on the reaction course. Whereas both diastereomers of ethyl acetoacetate derived VCP **5** react equally well both under thermal and photochemical conditions (entries 5–8, Table 2), the corresponding *tert*-butyl acetoacetate derived VCP **7** reacts at significantly different reaction rates under thermal conditions (entries 9–16, Table 2). For the *cis*-oriented ester and vinyl group a decrease of the overall yield was observed, a result that indicates the sterically demanding *tert*-butyl ester to block the S_N_2′- (or S_N_2-) anti trajectory of the incoming nucleophilic catalyst. In contrast, both diastereomers are equally reactive under photocatalytic conditions (entries 9–12, Table 2). In order to shed more light into this unexpected result, a test reaction in which a mixture of 25 mol% of the less reactive substrate **7b** and 75 mol% of the more reactive diastereomer **7a** was subjected to the thermal reaction conditions and the conversion was analyzed after 5 h. The more reactive substrate reacted at a significantly lower rate as compared to the same reaction using diastereomerically pure VCP **7a** (18% conversion for the mixture of **7a**/**7b**, **7a** was recovered in 75% yield; **7b** was recovered in 98% yield) *versus* 28% conversion for pure **7a** (**7a** was recovered in 65% yield; reaction conditions: 0.5 mmol of **7a** or 0.4 mmol of **7a**/0.1 mmol **7b**, 1 mol% of TBA[Fe], CH_2_Cl_2_, 45 °C). Apart from the steric arguments, one of the reviewers suggested an attractive orbital overlap between the incoming ferrate catalyst and the electron-withdrawing ketone and hence a favoured elongate formation to account for the inhibiting effect of the less reactive diastereomer. Future work will focus on this interesting stereoelectronic discrimination under thermal conditions. On the contrary, the lack of this discrimination might be the result of the change in complex configuration upon irradiation (*vide infra*), which results in an overall reduction of the metal-centered electron density with the adoption of a formally coordinatively unsaturated trigonal-bipyramidal configuration. The combination of electrophilicity at the metal center and a higher accessibility of the Fe center might explain the observed high reactivity to both VCPs **7a** and **7b** (*vide infra*). Furthermore, a correlation of the yield and the power of the light source was observed (entry 24, Table 2). The thermally unreactive vinylcyclopropane **17** and TBA[Fe] could be activated at room temperature using a 23 W household lamp to give the desired product in about 19% yield. Using a 75 W Xe-lamp, full conversion and an isolated yield of 96% was obtained (entry 24, Table 2).

**Scheme 1:**
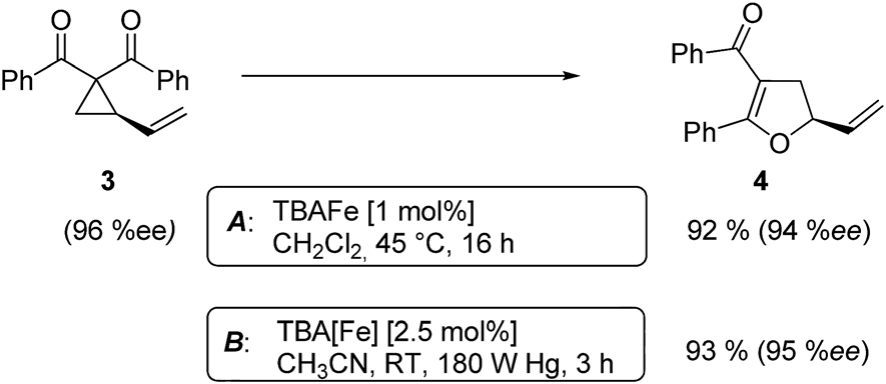
Stereoselectivity of the Cloke–Wilson rearrangement.

At this point we analyzed the stereoselective course of the TBA[Fe]-catalyzed Cloke–Wilson rearrangement (Scheme 1). Starting from the enantiomerically enriched (ee > 95%) vinylcyclopropane **3** the corresponding enantiomerically enriched product **4**, in which the new C–O bond was formed with retention of configuration, was isolated in an excellent yield and with near-complete transfer of enantiopurity both under thermal and photocatalytic conditions (Scheme 1). Importantly, the absolute configuration was unambiguously assigned using X-ray spectroscopy.^15^ This result indicates that a double S_N_2′- (or S_N_2-) mechanism is operative. Previous work from our group showed that the TBA[Fe]-catalyzed allylic substitution of allylic carbonates occurs with high regioselectivity and retention of configuration. We proposed a σ-enyl-type mechanism with a slow σ–π–σ equilibrium of the allyl-Fe complex to account for this unusual observation.8h–j More recently, however, we reported a quantum chemical study of the Cloke–Wilson rearrangement and noted that for this type of reaction both the S_N_2′- and the S_N_2-pathways could be operative.^12^

Whereas the VCP-rearrangement can follow both mechanisms, the situation changes drastically for the rearrangement of arylcyclopropanes (ACP). In this case the S_N_2-mechanism should be favourable. Consequently, TBA[Fe] was subsequently employed in the rearrangement of arylcyclopropanes, both under thermal as well as photochemical conditions. We were pleased to find the rearrangement of ACP **25** to proceed using substoichiometric amounts of the nucleophilic ferrate both under thermal conditions (120 °C, microwave, 200 W, 2 h, DMF) as well as under photochemical conditions (180 W Hg-lamp, 20 °C, DMF, 24 h). The thermal conditions are rather harsh, however, shorter reaction times and lower catalyst loadings as compared to the photochemical conditions are required (Table 3). Interestingly, DMF as a more polar solvent proved to be necessary in order to get the conversion both under thermal and photochemical conditions. In DMF, TBA[Fe] might exist as a solvent-separated ion pair with significantly increased metal-centered nucleophilicity.8*h* Different functional group compatibilities were observed. In particular halide atoms in the *para*-position of the aromatic unit had a significant effect on the rearrangement under photochemical conditions (entries 7–12, Table 3). Whereas the respective arylcyclopropanes undergo a clean rearrangement to the desired product under thermal conditions (entries 9 and 11, Table 3), lower yields for the reaction of *p*-chloro substituted ACP **39** and no conversion for *p*-bromo substituted ACP **41** were observed (entries 10 and 12, Table 3). The same effect was observed for the corresponding *meta*-substituted ACPs (entries 15–18, Table 3). Although we did not observe the product of protodehalogenation, we can not exclude this side-reaction as being operative and as leading to catalyst decomposition.

**Table 3:** Substrate scope of the Fe-catalyzed Cloke–Wilson rearrangement of arylcyclopropanes

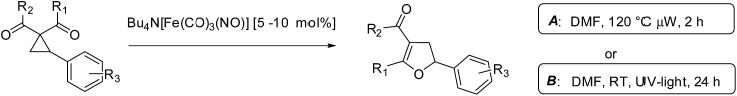									
Entry	Substrate	Product	Cond.^*a*^	Yield^*b*^ [%]	Entry	Substrate	Product	Cond.^*a*^	Yield^*b*^ [%]
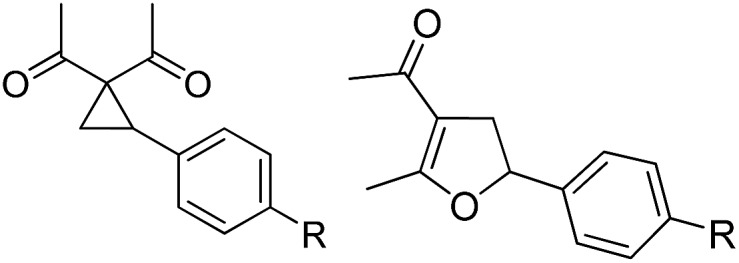					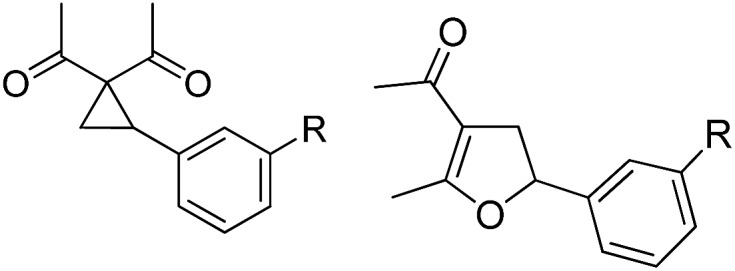				
1	**25** (R = H)	**26**	A	99	13	**37** (R = OMe)	**38**	A	85
2			B	82, 57^*c*^, 5^*d*^	14			B	93
3	**27** (R = Me)	**28**	A	99	15	**39** (R = Cl)	**40**	A	90
4			B	85	16			B	72
5	**29** (R = *t*-Bu)	**30**	A	75	17	**41** (R = Br)	**42**	A	84
6			B	77	18			B	—
7	**31** (R = F)	**32**	A	76	19	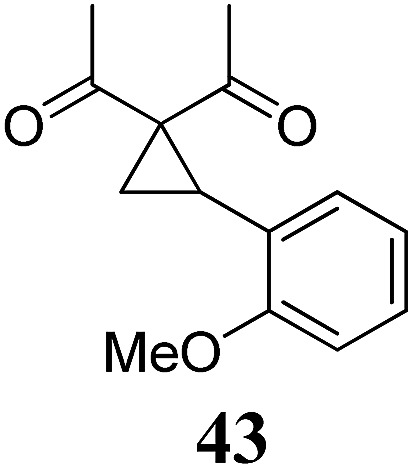	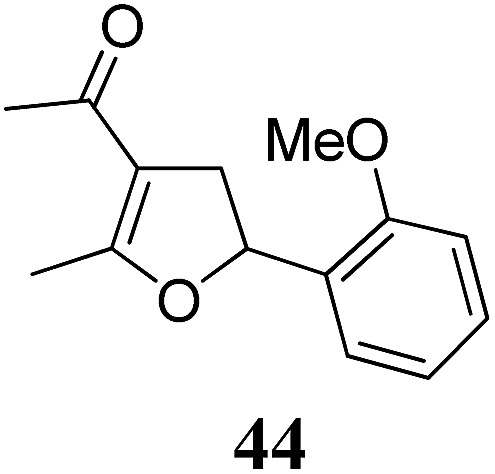	A	83
8			B	62	20			B	41
9	**33** (R = Cl)	**34**	A	92	20	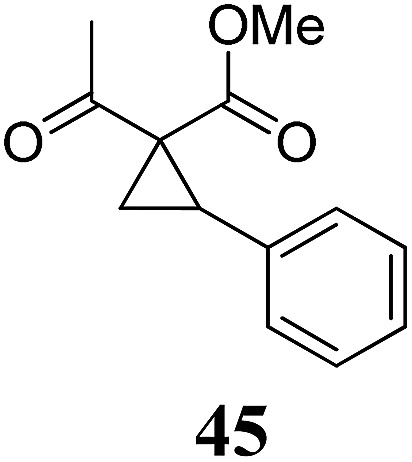	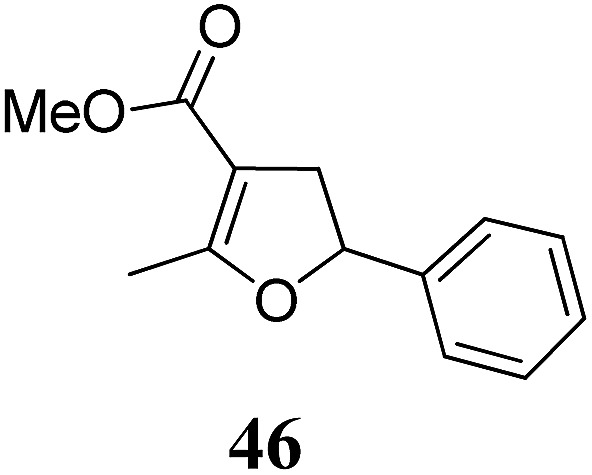	A	74
10			B	75	21			B	81
11	**35** (R = Br)	**36**	A	97	22	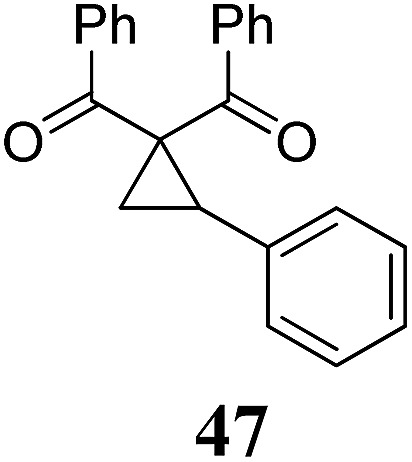	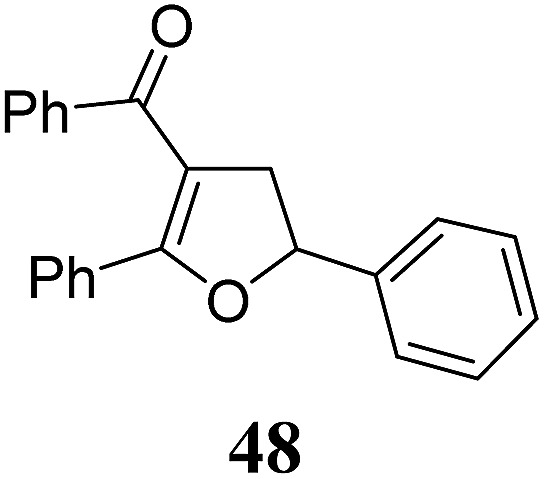	A	93
12			B	—	23			B	40

^*a*^Conditions A: 0.25 mmol of substrate, 5 mol% of TBA[Fe], 1 mL (abs.) of DMF, 120 °C, MW (200 W), 2 h; conditions B: 0.4 mmol of substrate, 10 mol% of TBA[Fe], 1 mL (abs.) of DMF, 180 W Hg-lamp, 20 °C, 24 h.

^*b*^Isolated yield.

^*c*^75 W Xe-lamp.

^*d*^23 W compact fluorescence lamp.

**Fig. 2:**
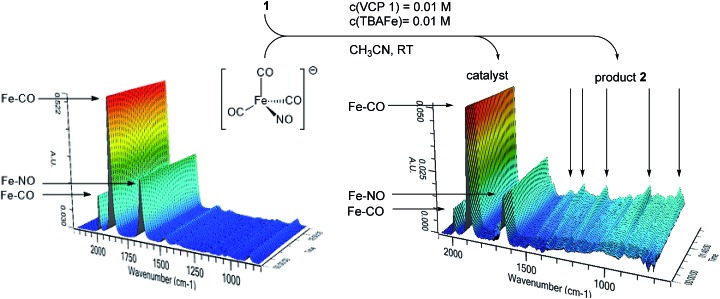
(1) *In situ* IR spectroscopic analysis of TBA[Fe] under UV-irradiation (180 W Hg-lamp), (2) *in situ* IR spectroscopic analysis of the photochemical TBA[Fe]-catalyzed Cloke–Wilson rearrangement of **1**.

In principle, different scenarios for the photochemical activation of Bu_4_N[Fe(CO)_3_(NO)] (TBA[Fe]) are possible. In order to get first insights into a probable mode of activation, an acetonitrile solution of TBA[Fe] was irradiated at room temperature with the 180 W Hg-lamp employed in the catalysis. The carbonyl and nitrosyl signals were observed using *in situ* IR-ATR spectroscopy (Fig. 2). However, even after extended irradiation times no significant change in the IR-spectrum was observed. Furthermore, repetition of the same experiment with the addition of Ph_3_P as a suitable ligand did not result in ligand exchange.^15^ Moreover, when VCP **1** was added to the mixture and the reaction course was monitored using *in situ* IR spectroscopy, no change in the TBA[Fe] signals even after full conversion was detected (Fig. 2). Hence, at the current stage of the research it seems as if a photochemically induced decarbonylation of TBA[Fe] might not be the activating step.

**Fig. 3:**
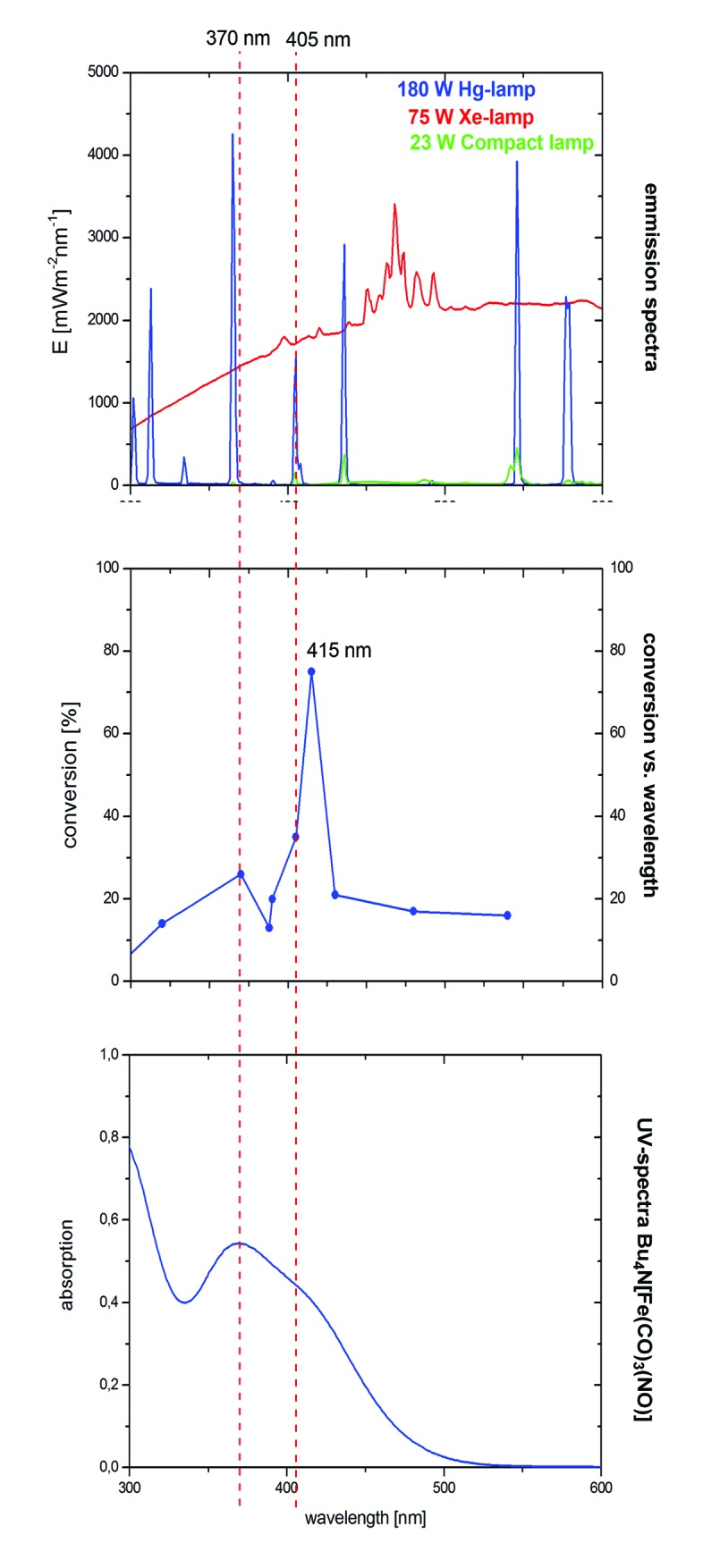
Overlay of the emission and absorption spectra with the conversion–wavelength correlation in the rearrangement of VCP **1**.

In the case of the VCP-rearrangement, irradiation using a 23 W lamp was sufficient for the activation of TBA[Fe]. Both the 180 W Hg- and the 75 W Xe-lamps have strong emissions in the UV but also in the visible light region (Fig. 3).^16^ The complex has absorption maxima at 242, 263, 292, and 370 nm. Furthermore, there is a saddle point at 410 nm. Very much to our surprise, a conversion–wavelength correlation of the TBA[Fe]-catalyzed rearrangement of **1** indicated that the complex has the highest activity at 415 nm (Fig. 3).

Interestingly, a significant drop in the conversion was observed at wavelengths below 405 and above 370 nm. Apparently, the Hg-emission line at 405 nm that was observed for the 23 W household lamp is responsible for the observed activity. At the current stage of the research we believe that this higher photon flux accounts for the increase in reactivity, *e.g.* in the rearrangement of ACPs. Based on our assumption that TBA[Fe] is the active catalyst and in order to get a deeper insight into the electronic structure of the potential activated species, quantum-chemical investigations were carried out.^15^

The ferrate, [Fe(CO)_3_(NO)], is an anionic complex, however, computations indicate that the solvated anion will not be oxidized by irradiation up to 3.5 eV (354 nm). The predicted electron affinity of the isolated neutral [Fe(CO)_3_(NO)] complex is 2.8 eV with DFT (PBE functional^17^), while MRCI+Q^18^ calculations result in 1.8 eV. Solvation effects stabilize the anion significantly, by 1 eV in apolar solvents and up to 2 eV in polar solvents, as estimated using a continuum solvation model.^19^ While a more accurate determination of the electron affinity will require more rigorous calculations, we can safely estimate that the anionic species will be stable under the herein considered experimental conditions and the catalytic activity will only be due to an excited anionic ferrate species.

The DFT-calculated equilibrium structure of the anionic species has *C*_3v_ point-group symmetry and the structure parameters are similar to the ones reported in previous works.^9^ Starting from this structure, we subsequently calculated the vertical excitation spectrum of [Fe(CO)_3_(NO)] using MRCI+Q (Table 4).

**Table 4:** Calculated vertical excitation energies of the [Fe(CO)_3_(NO)] anion^*a*^

Singlet excitations				Triplet excitations		
State (sym.)	Δ*E* [eV]	*λ* [nm]	*f* ^*b*^	State (sym.)	Δ*E* [eV]	*λ* [nm]
S_1_ (^1^A_2_)	3.26	381	0	T_1_ (^3^A_1_)	2.32	534
S_2_ (^1^E)^*c*^	3.53	351	0.008	T_2_ (^3^E)^*c*^	2.96	420
S_3_ (^1^A_1_)	3.64	340	0.013	T_3_ (^3^A_2_)	3.15	394
S_4_ (^1^E)^*c*^	4.16	298	0.002	T_4_ (^3^A_1_)	3.79	327
S_5_ (^1^A_2_)	4.34	286	0	T_5_ (^3^E)^*c*^	3.99	311
S_6_ (^1^A_1_)	4.75	261	<0.001	T_6_ (^3^A_2_)	4.31	287

^*a*^icMRCI+Q/def2-TZVPP calculations at the PBE/def2-TZVPP′ optimized structure.

^*b*^Oscillator strength (length gauge) based on the MRCI transition density.

^*c*^Average over slightly symmetry broken MRCI+Q results for the two columns of the *E* representation (deviations are <0.04 eV).

As expected from the orbital structure (both HOMO and LUMO are degenerate) we obtain four close-lying singlet excited states in the region of 3.3 to 3.6 eV of A_2_, E and A_1_ symmetry. CASSCF-based structure optimizations^15^,^20^ of the excited states indicate that these states undergo strong structural deformations with reorganization energies in the order of 1 eV. This explains the broad band observed in the experimental spectrum, ranging from 2.8 eV to 3.9 eV. We note that the transition to the lowest singlet A_2_ state is symmetry forbidden but non-vanishing absorption strength may occur in the spectrum due to vibronic coupling.

**Fig. 4:**
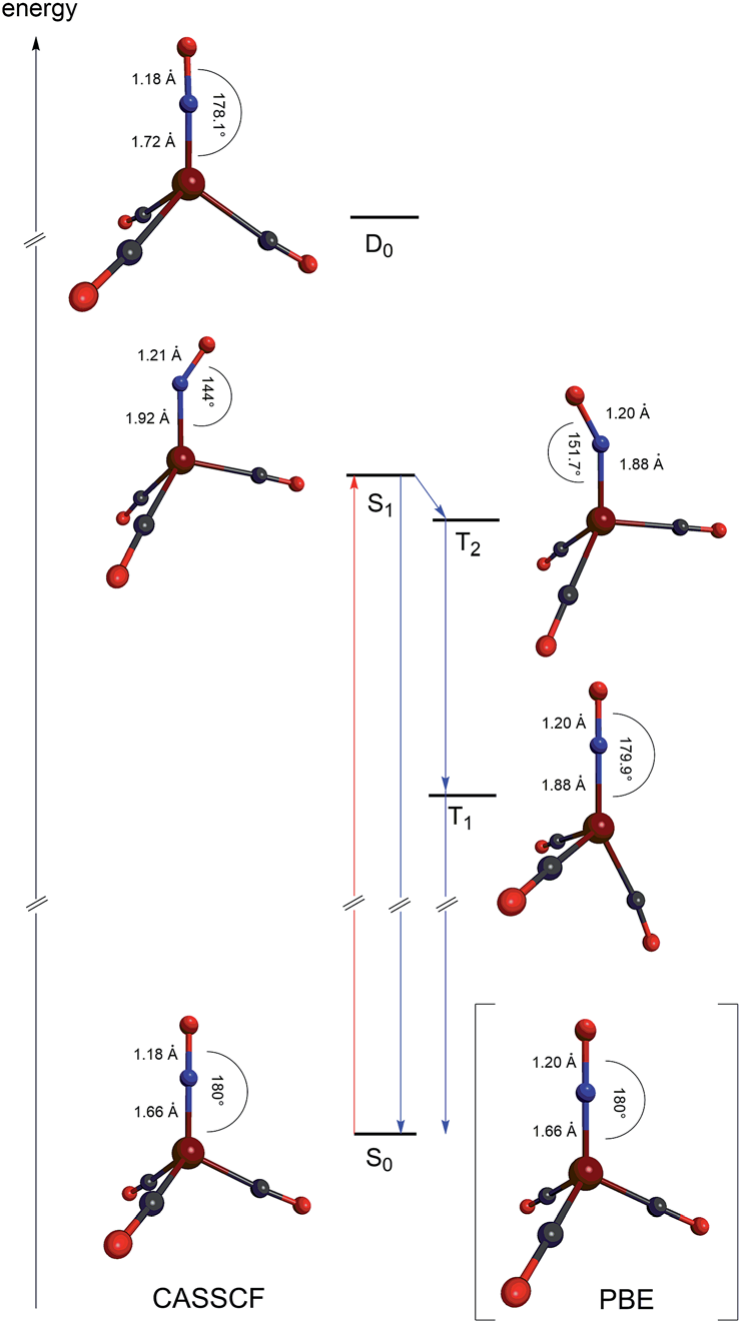
A schematic energy level diagram along with the CASSCF/def2-TZVPP equilibrium structures of [Fe(CO)_3_(NO)]. The anionic ground state S_0_, anionic lowest singlet excited state S_1_, anionic lowest triplet excited state T_1_, anionic triplet state T_2_, and neutral doublet ground state D_0_. The DFT (PBE/def2-TZVPP) derived S_0_ equilibrium structures of the anionic ferrate are shown in parentheses.

The excited state structures calculated at the CASSCF level are shown in Fig. 4, along with a schematic energy level scheme derived from MRCI+Q and CASSCF calculations.

We find that the S_1_ and T_2_ states are close in energy, in particular at the relaxed S_1_ structure where the S_1_ energy is even 0.03 eV below the T_2_ energy at the CASSCF level. This is in accordance with the S_1_ structure, which shows an Fe–N–O bond angle of 144°, indicative of the loss of π-bond character and the reduction of the NO-ligand.^15^ Electronically, two unpaired electrons reside on both Fe and NO and couple only weakly, such that the singlet and triplet are nearly isoenergetic. Even though only a weak spin–orbit coupling between S_1_ and T_2_ is calculated (6 cm^–1^ at the CASSCF level), the near-degeneracy close to the S_1_ equilibrium structure will drive efficient intersystem crossing to T_2_, followed by fast internal conversion to T_1_. Hence, we expect that the activated [Fe(CO)_3_(NO)] species is in the T_1_ state. In this state, the complex adopts an almost trigonal-bipyramidal configuration with an Fe–N–O angle of close to 180°, in which the metal center might be considered to possess an open binding site. Consequently, this complex geometry might facilitate the coordination of the olefinic substrate as the initial step of the catalytic reaction. The fast intersystem crossing from S_1_ to T_2_ and the fast internal conversion prevents a detection of the Fe–NO-species with bent NO-ligands using the spectroscopic methods used in this work. Research aiming to detect these short-lived intermediates is ongoing. The NAO charges of the lowest [Fe(CO)_3_(NO)] states are listed in Table 5.

**Table 5:** Natural atomic orbital charges of the lowest states of [Fe(CO)_3_(NO)]^*a*^

	S_0_^*b*^ {Fe^8.00^(NO)}^10^	S_1_ {Fe^7.34^(NO)}^10^	T_1_ {Fe^7.48^(NO)}^10^	T_2_ {Fe^7.36^(NO)}^10^
Fe	0.00 (–0.07)	0.66	0.52	0.64
N	–0.10 (–0.05)	–0.35	–0.32	–0.37
O	–0.42 (–0.41)	–0.43	–0.41	–0.39
C	–0.48 (–0.47)	–0.87	–0.79	–0.88

^*a*^Natural atomic orbitals (NAOs) were obtained from the state-averaged CASSCF/def2-TZVPP calculations. The corresponding CASSCF/def2-TZVPP′ equilibrium structures were used. All charges are multiples of the elementary charge *e*_0_.

^*b*^The NAO charges obtained using the PBE-derived equilibrium structure are shown in parentheses.

The NAO analysis shows that the Fe center after excitation has a positive partial charge of about 0.5*e*_0_ while significant charge transfer into the ligand backbonds is observed. The nitrogen atom of the NO-ligand is reduced by about 0.3*e*_0_. Furthermore, a significant reduction of the CO-ligands is observed for all excited states.

## Experimental section

### General remarks

The photochemical reactions were performed in borosilicate glassware. The distance between the glassware and lamp was 15 cm.

### General procedure for the thermal VCP rearrangement

TBA[Fe] (10.3 mg, 0.025 mmol) was weighed into a dried Schlenk tube. Anhydrous CH_2_Cl_2_ (5 mL) was added and the mixture was stirred until all solids dissolved. 1 mL (0.005 mmol TBA[Fe]) of this solution was transferred into a separate dried Schlenk tube which was subsequently charged with the appropriate vinylcyclopropane (0.5 mmol). The Schlenk tube was sealed under an atmosphere of dry nitrogen and heated to 45 °C for 16 h. The solvent was removed under reduced pressure and the residue was subjected to column chromatography on silica gel.

### General procedure for the photochemical VCP rearrangement

A 10 mL Schlenk tube was charged with vinylcyclopropane (0.40 mmol, 1 equiv.), TBA[Fe] (0.001 mmol, 0.025 equiv.), and CH_3_CN (1 mL) under N_2_. The reactions were carried out at room temperature under irradiation using the respective light source for 3 h. The reaction was quenched with diethyl ether and concentrated *in vacuo*. Purification using silica column chromatography afforded the desired dihydrofuran product.

### General procedure for the thermal ACP rearrangement

TBA[Fe] (5.2 mg, 0.0125 mmol, 0.05 equiv.) and the corresponding arylcyclopropane (0.25 mmol, 1 equiv.) were weighed into a dried 10 mL microwave tube. Anhydrous DMF (1 mL) was added and the tube was sealed under an atmosphere of dry nitrogen. The reaction mixture was stirred for 2 h at 120 °C under microwave conditions. The pure product was obtained after chromatography on silica gel.

### General procedure for the photochemical ACP rearrangement

A 10 mL Schlenk tube was charged with arylcyclopropane (0.40 mmol, 1 equiv.), TBA[Fe] (0.04 mmol, 0.1 equiv.), and DMF (1 mL) under N_2_. The reactions were carried out at room temperature under light irradiation for 24 h. The reaction was quenched with diethyl ether and concentrated *in vacuo*. Purification using silica column chromatography afforded the desired dihydrofuran product.

### Computational details

All computations were carried out with the Molpro 2012.1 program package.^21^ Molecular structures were determined at the DFT level of theory using the PBE and PBE0 functionals^17^ and at the state-averaged CASSCF level of theory.^20^ We employed the def2-TZVPP basis set,^22^ omitting the *g*-functions however, due to restrictions in the state-averaged CASSCF gradient code (the basis will be called def2-TZVPP′ in this work). Both the DFT closed-shell and open-shell calculations relied on a restricted Kohn–Sham scheme. Solvent effects were estimated using COSMO^19^ with standard parameters for the cavity construction and two values for the dielectric constant: *ε* = 2.5 to model apolar solvents and *ε* = ∞ to model strongly polar solvents.^15^ The CASSCF calculations were run with averaging over five states and four states in the singlet and triplet manifold of anionic [Fe(CO)_3_(NO)], respectively, and with averaging over four states for the neutral species. The active space consisted of all Fe d orbitals and the NO π and π* orbitals (12 electrons in 9 orbitals). *C*_s_ point-group symmetry of the molecule was exploited in all calculations (the actual point group of anionic [Fe(CO)_3_(NO)] is *C*_3v_ but Jahn–Teller distortions occurred for all excited and ionized states).

Vertical excitation energies were computed with internally contracted MRCI^18^ and a modified Davidson correction for disconnected quadruple excitations (icMRCI+Q).18*a* Orbitals were taken from the state-averaged CASSCF calculations, using the same active space as described above. The 1s orbitals of C, N, and O and the (1s2s2p3s3p) orbitals of Fe were excluded from the correlation treatment.

Natural atomic orbitals (NAOs) were determined from the CASSCF densities.^23^

## Conclusions

**Fig. 5:**
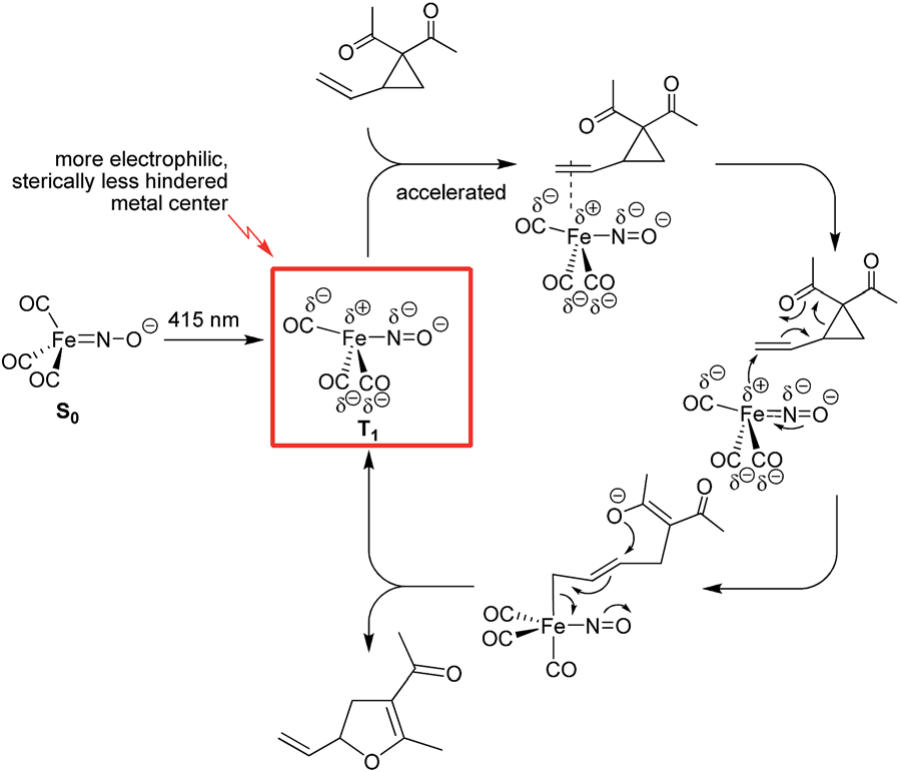
Mechanistic proposal for the photochemical Fe-catalyzed Cloke–Wilson rearrangement of VCPs following an S_N_2′-anti mechanism.

Herein we describe a comparative study of the use of Bu_4_N[Fe(CO)_3_(NO)] (TBA[Fe]) in the thermal *versus* photochemical Cloke–Wilson rearrangement of vinyl- and arylcyclopropanes. The catalyst shows good reactivity under both conditions and allows the preparation of a variety of substituted dihydrofurans under mild conditions. Upon irradiation the catalytic transformation can be performed at room temperature. In-operando spectroscopic investigations suggest that the Fe-carbonyl catalyst is probably not decarbonylated under photochemical conditions. Detailed studies on the correlation of the wavelength *versus* conversion showed that the best conversion rate is achieved at 415 nm. This value is close to a saddle point within the absorption curve of the ferrate. Quantum chemical investigations of the potential activated singlet and triplet states of the ferrate catalyst gave insight into the vertical excitation energies and show that the ferrate has an S_1_-state, from which the activated species can undergo intersystem crossing into the nearly isoenergetic triplet state, T_2_, from which the energetically favorable T_1_ state is accessible *via* internal conversion. The deviations of the Fe–N–O bond angle from 180° for S_1_ and T_2_ are indicative of a significant charge transfer into the backbond of the NO- and CO-ligands, leading to a polarization of the complex in which the metal center is partially positively charged. A significant Jahn–Teller-distortion is observed, which leads to a change of the coordination mode from the former tetrahedral 18-electron complex in the ground state S_0_ into a formally unsaturated trigonal-bipyramidal complex in the triplet state T_1_. This coordination mode and the partial positive charge of the metal center facilitate the formation of a reactive π-complex as the initial step within the proposed catalytic cycle (Fig. 5).

The electron affinity is reduced in the excited state and the electron will be more readily transferred to the substrate in an S_N_2′-anti fashion. The nitrogen and the carbon atoms of the CO-ligand act as electron-donating centers that pump electrons into the antibonding π*C=C-orbital of the C=C bond within the substrate. The allyl-Fe complex, which is formed upon electron transfer reacts with the O-nucleophile in an S_N_2′-anti fashion to regenerate the Fe complex in the T_1_ state.

**Fig. 6:**
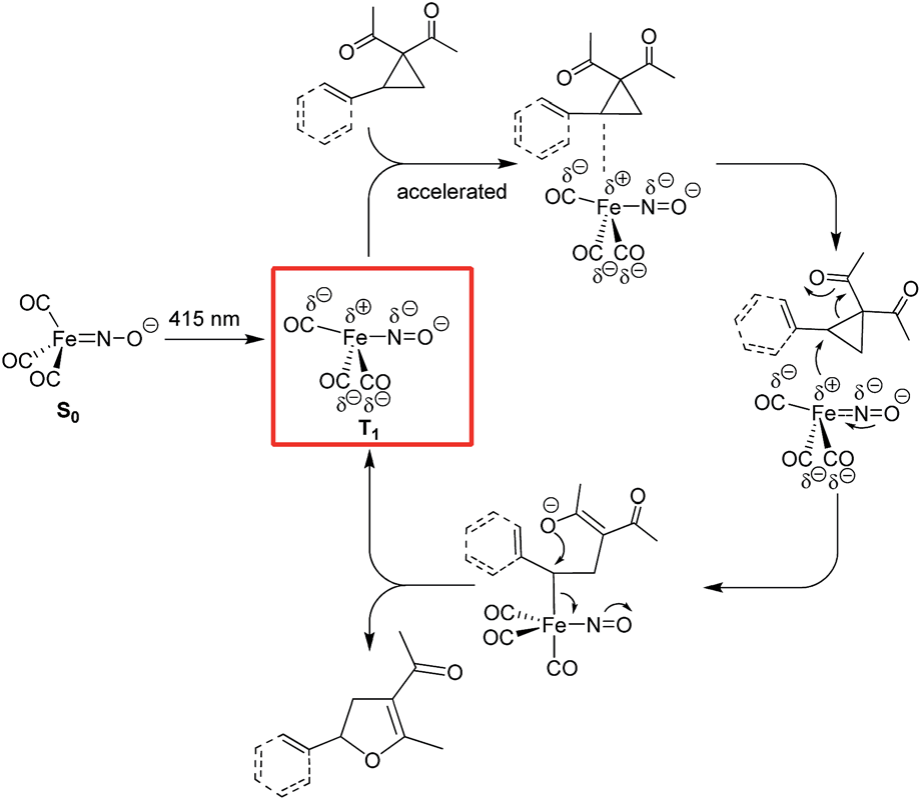
Mechanistic proposal for the photochemical Fe-catalyzed Cloke–Wilson rearrangement of VCPs/ACPs following an S_N_2-anti-mechanism.

On the contrary, the adoption of a trigonal-bipyramidal configuration in the T_1_-state allows the electron-rich, formally unsaturated and sterically less hindered Fe complex to access the σ*C–C-orbital of the tertiary benzylic (or allylic) C atom in aryl- or vinylcyclopropanes with concomitant C–C bond activation (Fig. 6). Reaction with the formed O-nucleophile in an S_N_2-fashion closes the catalytic cycle with the formation of the respective dihydrofuran products.

These results open up a new research direction using Bu_4_N[Fe(CO)_3_(NO)] as a stable catalyst that is selectively activated at 415 nm, a region in which common (non-catalytic) photochemical organic (side) reactions are not operative. Studies aiming to use this concept for the activation of less reactive C–H and C–C bonds are currently being carried out in our laboratories.

## Abbreviations

ACP: Arylcyclopropane
VCP: Vinylcyclopropane

## Supplementary Material

Supplementary information

Crystal structure data
