# Supplementary material for: Non-decarbonylative photochemical versus thermal activation of Bu4N[Fe(CO)3(NO)] – the Fe-catalyzed Cloke–Wilson rearrangement of vinyl and arylcyclopropanes
Source: Chem Sci. 2015 Sep 3;6(12):7034–43. doi: 10.1039/c5sc02342d (PMC5947516; doi:10.1039/c5sc02342d)
Supplement: Supplementary file 1 [file SC-006-C5SC02342D-s001.pdf]

Electronic Supplementary Material (ESI) for Chemical Science.
This journal is © The Royal Society of Chemistry 2015

Non-decarbonylative photochemical versus thermal activation of $Bu_4N[Fe(CO)_3(NO)]$ – the Fe-catalyzed Cloke-Wilson rearrangement of vinyl and arylcyclopropanes

*Che-Hung Lin,[a] Dominik Pursley,[a] Johannes E. M. N. Klein,[a] Johannes Teske,[a] Jennifer A. Allen,[b] Fabian Rami,[a][c] Andreas Köhn[c] and Bernd Plietker[a]**

[a] Institut für Organische Chemie, Universität Stuttgart, Pfaffenwaldring 55, DE-70569 Stuttgart, Germany, e-mail: bernd.plietker@oc.uni-stuttgart.de

[b] Mettler-Toledo GmbH, Ockerweg 3, D-35396 Giessen, Germany

[c] Institut für Theoretische Chemie, Universität Stuttgart, Pfaffenwaldring 55, DE-70569 Stuttgart, Germany.

Table of Contents

**1. General Remarks**	**S2**
**2. IR spectra of TBA[Fe]**	**S2**
**3. UV spectrum of TBA[Fe]**	**S7**
**4. Fluorescence spectrum of TBA[Fe]**	**S7**
**5. Conversion-Time and Temperature-Time Plot for the Rearrangement of VCP 1 (measured by in-situ IR-spectroscopy)**	**S8**
**6. Preparation of Vinylclopropanes**	**S8-19**
**7. Preparation of Arylcyclopropanes**	**S19-24**
**8. $NBu_4[Fe(CO)_3(NO)]$ (TBAFe)-catalyzed Cloke-Wilson Rearrangement of Vinylcyclopropanes**	**S24-38**
**9. $NBu_4[Fe(CO)_3(NO)]$ (TBAFe)-catalyzed Cloke-Wilson Rearrangement of Arylcyclopropanes**	**S38-45**
**10. ^1H and ^{13}C-NMR Spectra**	**S46-96**
**11. Additional Information for Crystals**	**S97-S144**
**12. Computed Structures and Energies**	**S145-S151**
**13. References**	**S152**

1. General Remarks

All reactions sensitive to moisture and/or air were carried out under an atmosphere of dry nitrogen (N_2) using anhydrous solvents. Solvents were either dried by passing them through commercially available columns (*n*-pentane, CH_2Cl_2) or distilling them from CaH_2 (CCl_4, $C_2H_2Cl_4$, $C_2H_4Cl_2$, PhH). THF was freshly distilled from Na/benzophenone (ketyl radical). IR spectra were recorded on a Bruker Vector 22 FT-IR spectrometer equipped with a Specac Golden Gate ATR unit. High resolution mass spectra (HRMS) were recorded using a Finnigan MAT 95 spectrometer (EI) or a Bruker micrOTOF-Q spectrometer (ESI). NMR spectra were recorded on a Bruker Avance 250MHz, 300 MHz or 500 MHz spectrometer and calibrated using the residual non-deuterated solvent signal or tetramethylsilane (TMS) as an internal standard. Column chromatography (CC) was carried out using silica gel (60 Dm, 0.040-0.063 mm) and thin layer chromatography (TLC) was carried out using silica gel plated aluminum sheets (silica gel 60, F_{254}). High performance liquid chromatography (HPLC) was carried out using a K-501 pump and a K 2400 RI-detector in combination with a Eurospher-100 Si column by KNAUER. The photochemical reactions were carried out using a Heraeus Hg-Lamp (180 W), a ORIEL Xenon-Lamp (75 W), or a MEGAMAN Compact Fluorescent Lamp (23 W, 1395 Lumen).

2. IR spectra of TBA[Fe]

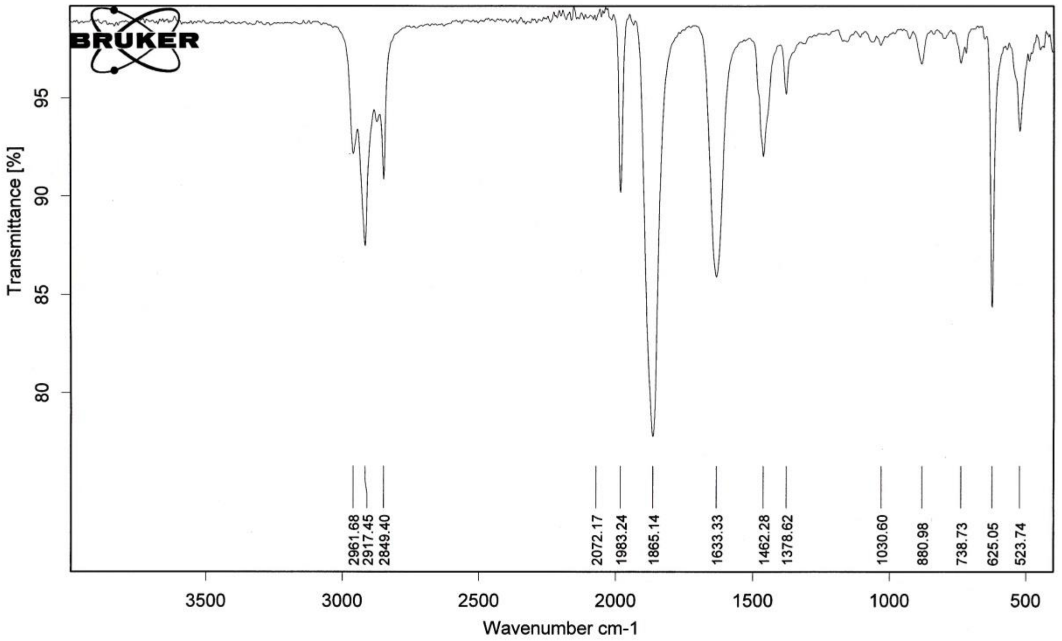

Figure S1: TBA[Fe] in CH_3CN - no irradiation.

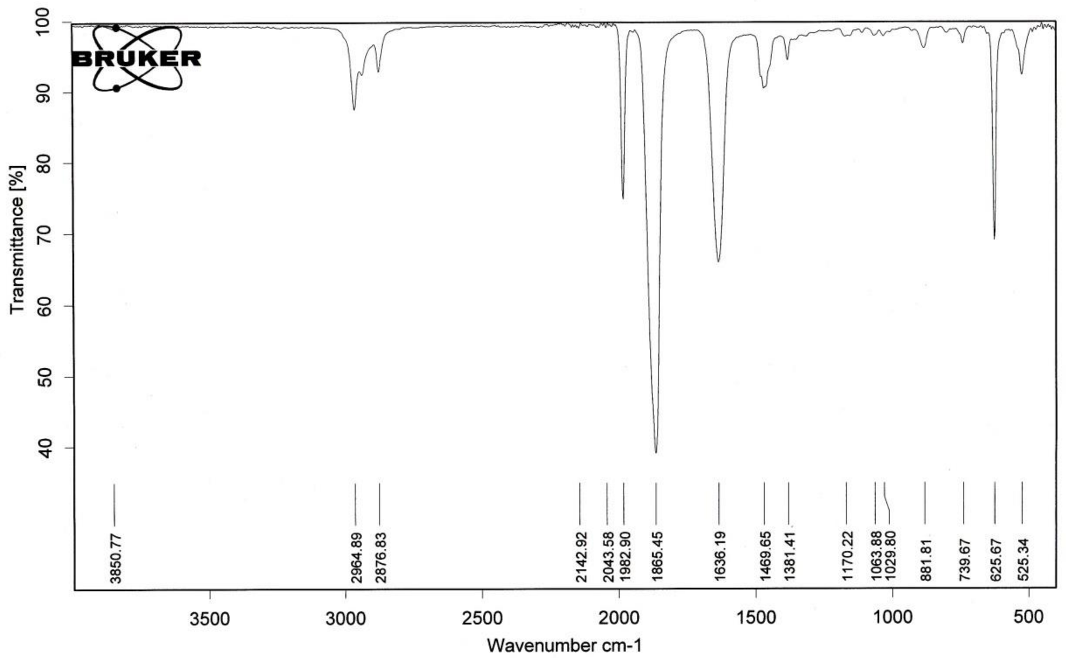

Figure S2: TBA[Fe] in CH_3CN – after 6 h irradiation (180 W, Hg lamp).

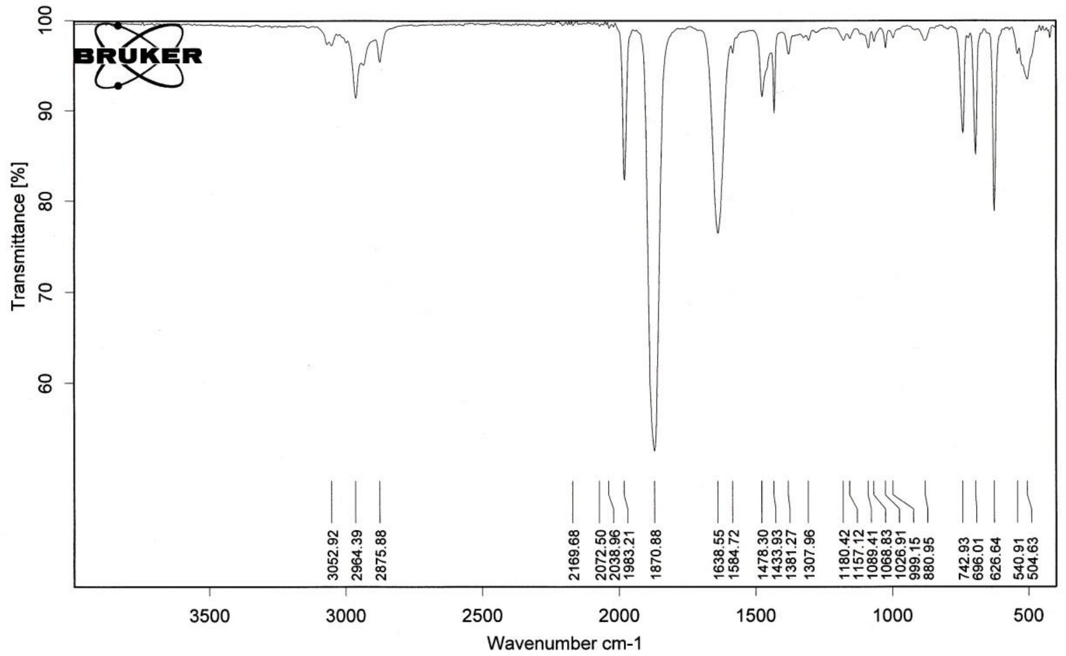

Figure S3: TBA[Fe] + PPh_3 (1.5 equiv.) in CH_3CN **-** after 4 h irradiation (180 W, Hg lamp).

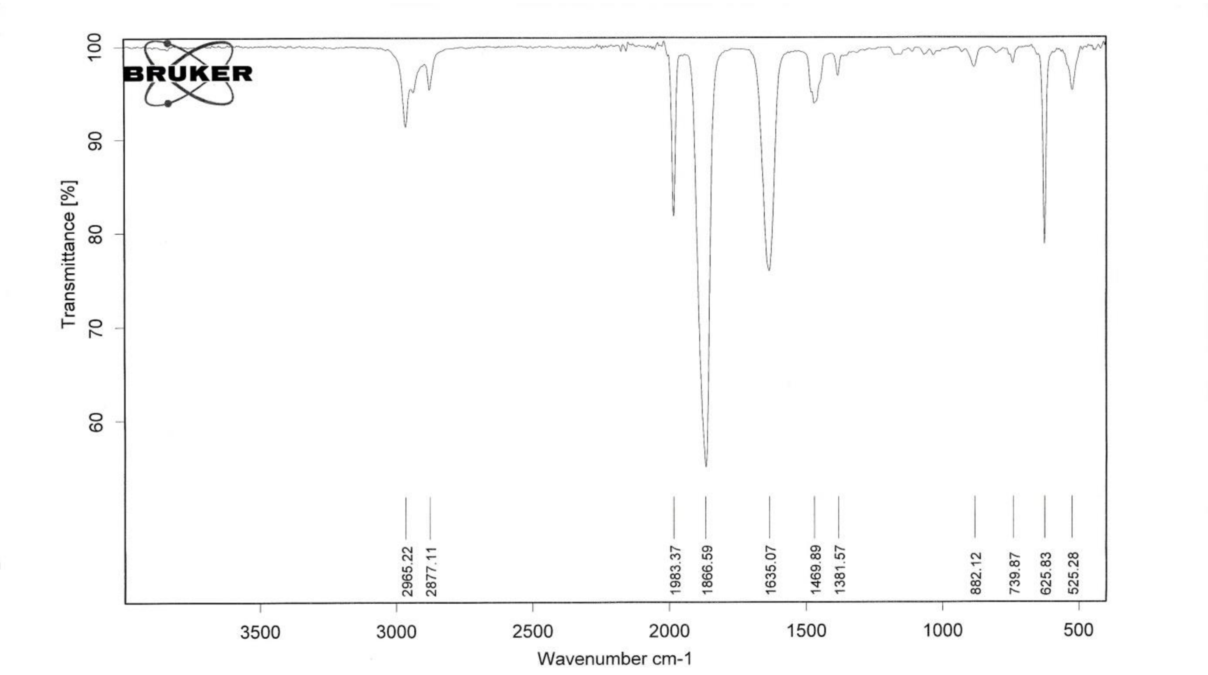

Figure S4: TBA[Fe] in DCM - no heating.

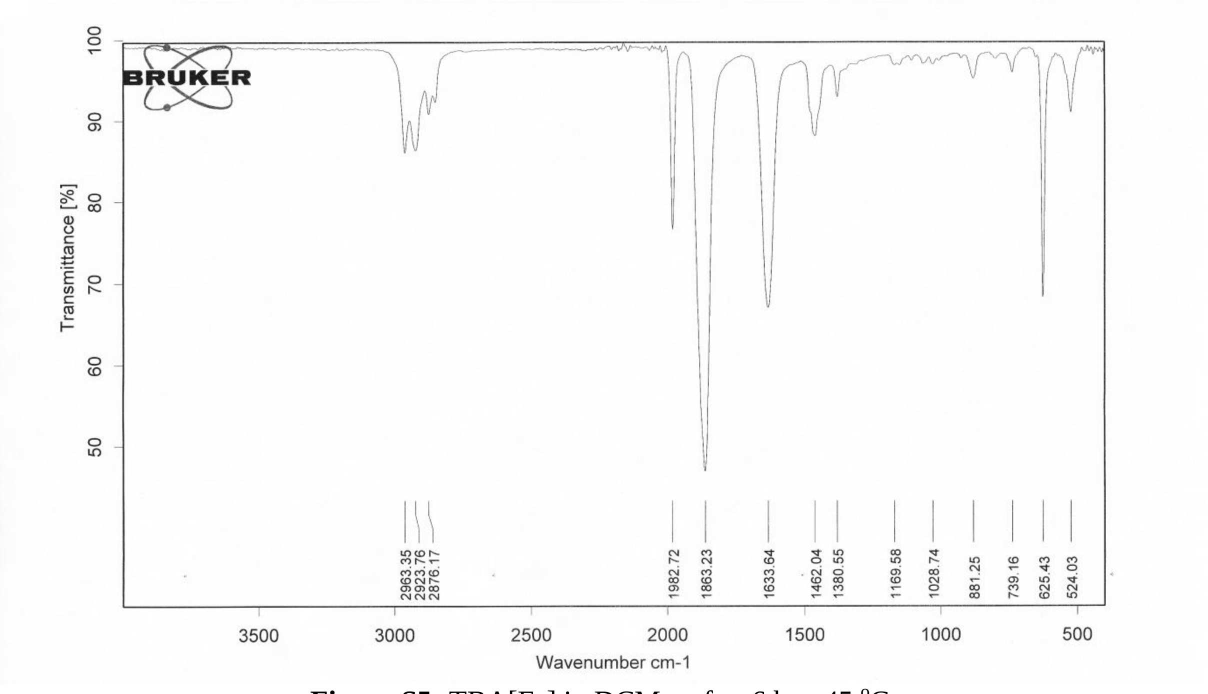

Figure S5: TBA[Fe] in DCM – after 6 h at 45 ^{o}C.

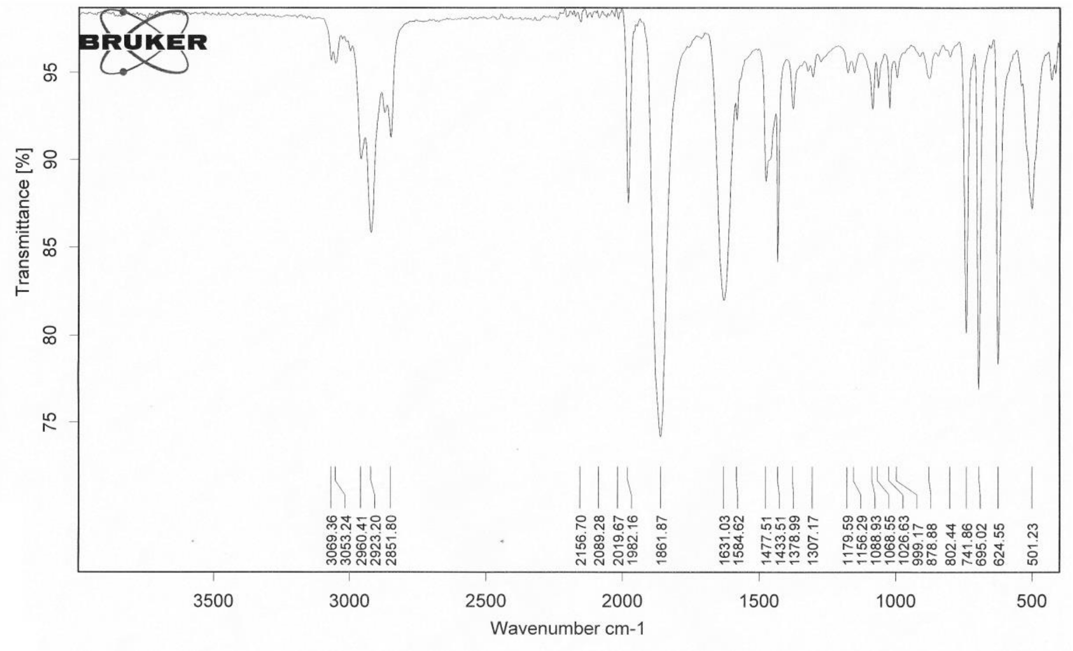

Figure S6: TBA[Fe] + PPh_3 (2 equiv.) in DCM - after 4 h at 45 ^{o}C.

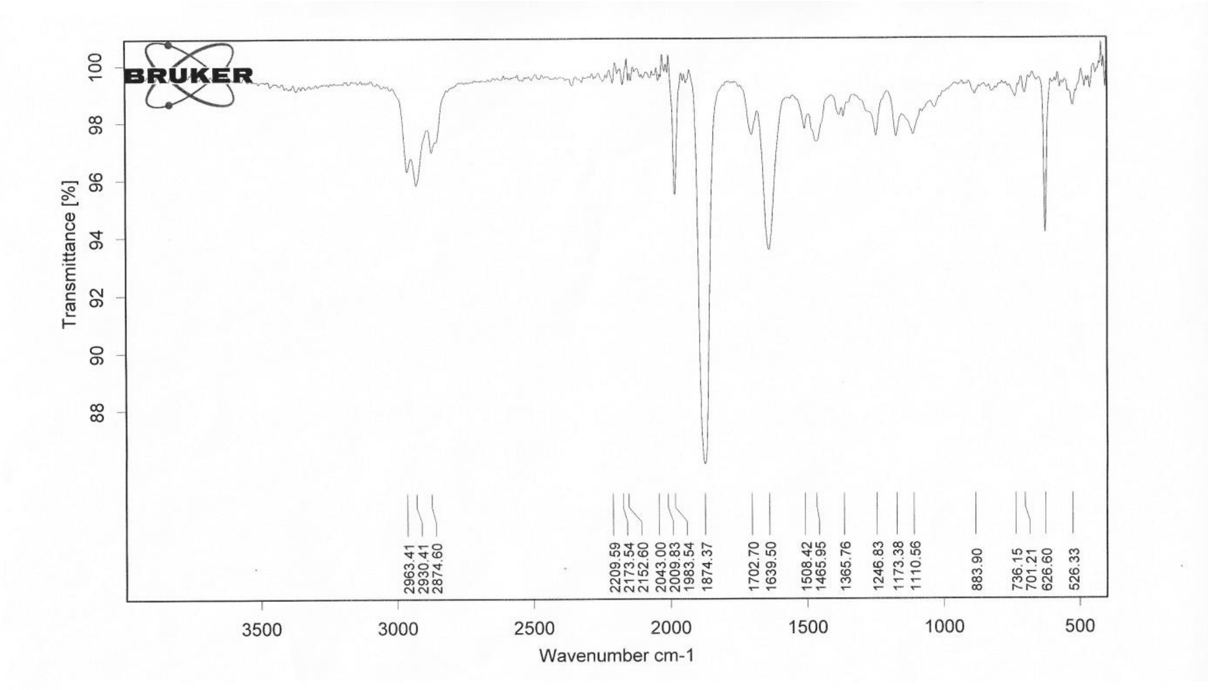

Figure S7: TBA[Fe] in Toluene - no MW.

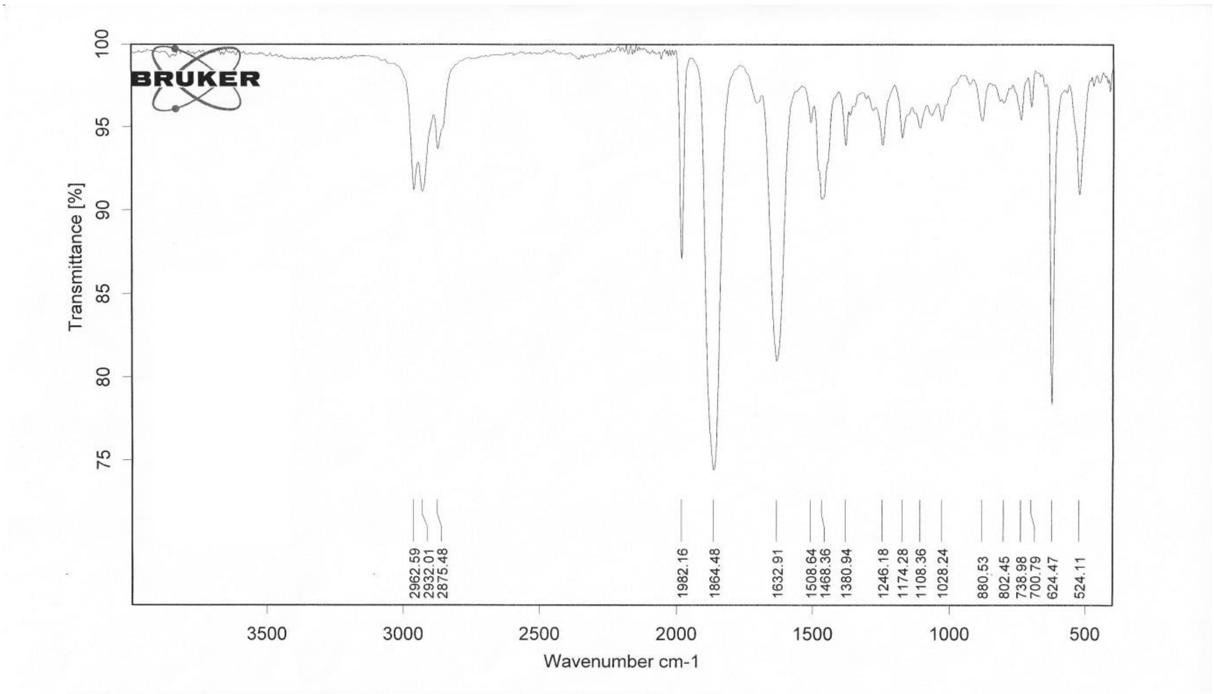

Figure S8: TBA[Fe] in Toluene - after 2 h MW at 120 ^{o}C.

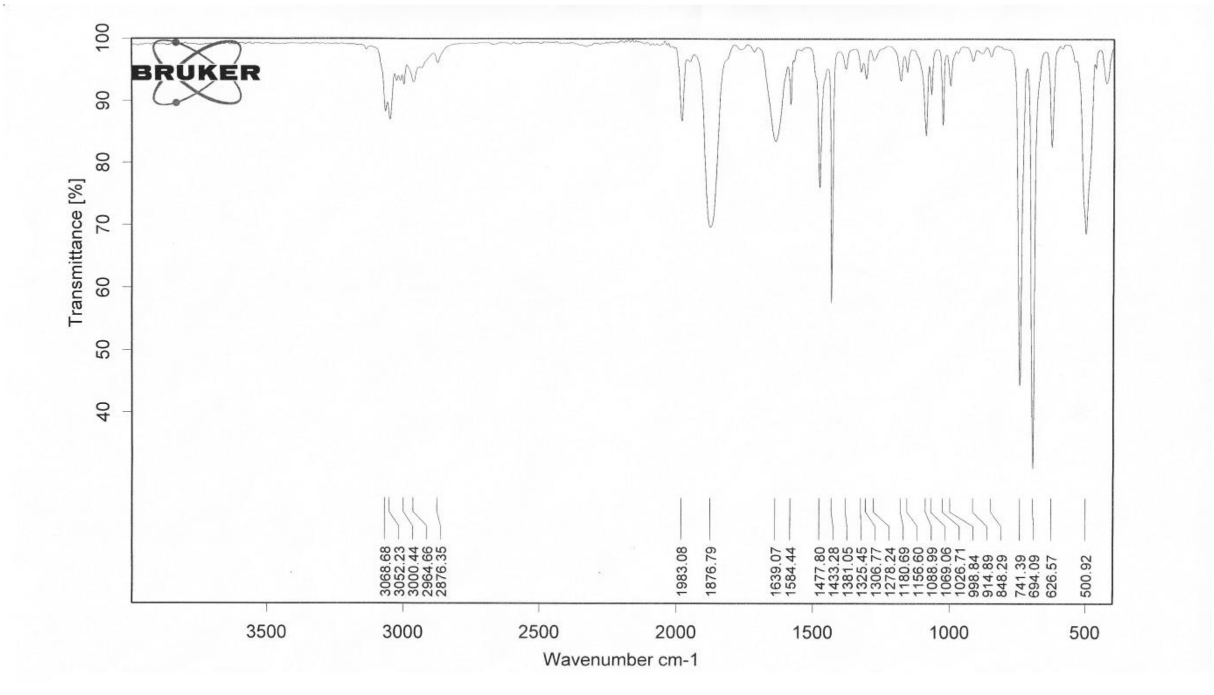

Figure S9: TBA[Fe] + PPh_3 (2 equiv.) in Toluene - after 2 h MW at 120 ^{o}C.

3. UV spectrum of TBA[Fe]

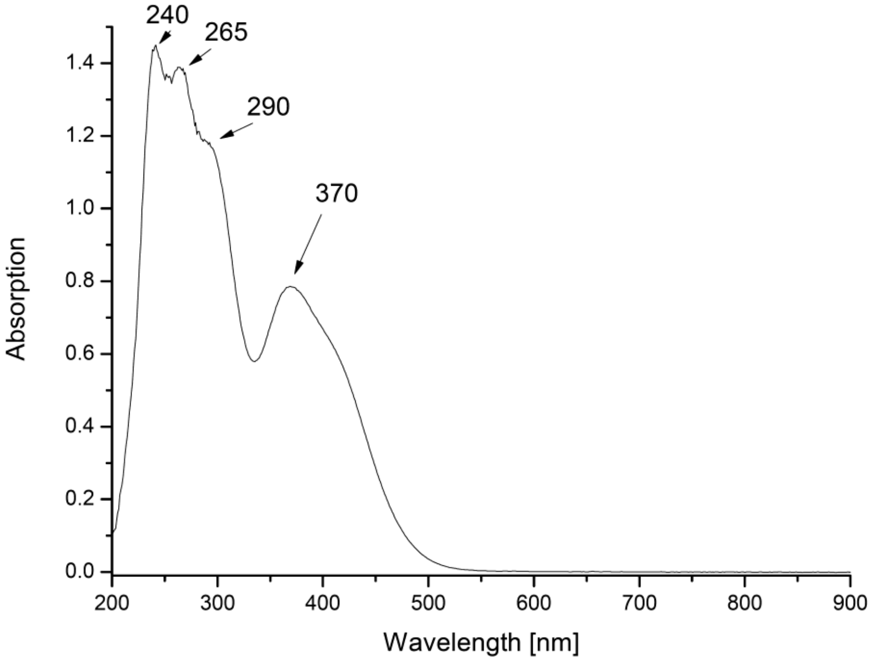

Figure S10: UV spectrum of TBA[Fe] in CH_3CN.

4. Fluorescence spectrum of TBA[Fe]

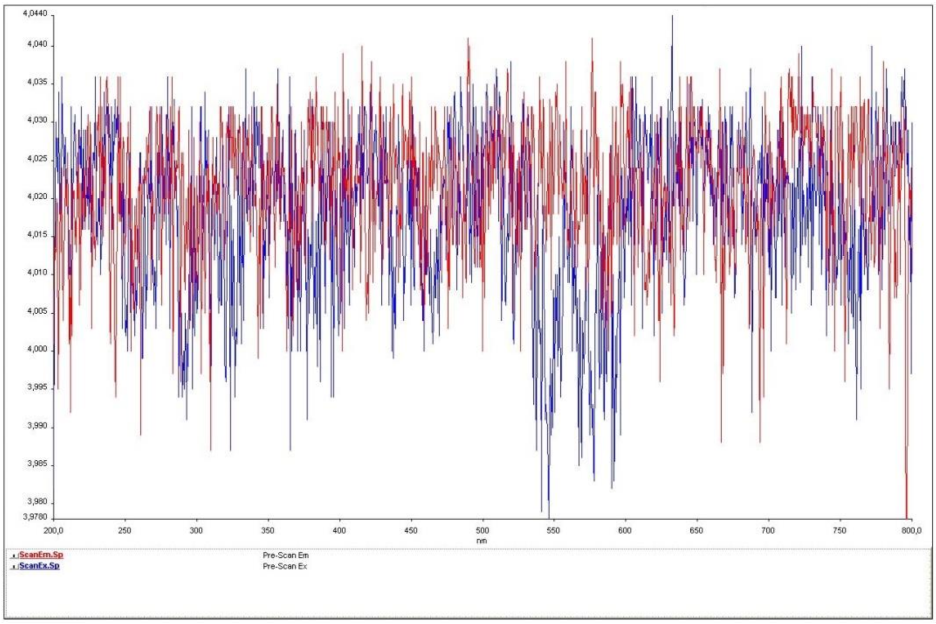

Figure S11: Fluorescence spectrum of TBA[Fe] in CH_3CN. The excitation scan (blue) and emission scan (red).

5. Conversion-Time and Temperature-Time Plot for the Rearrangement of VCP 1 (measured by in-situ IR-spectroscopy)

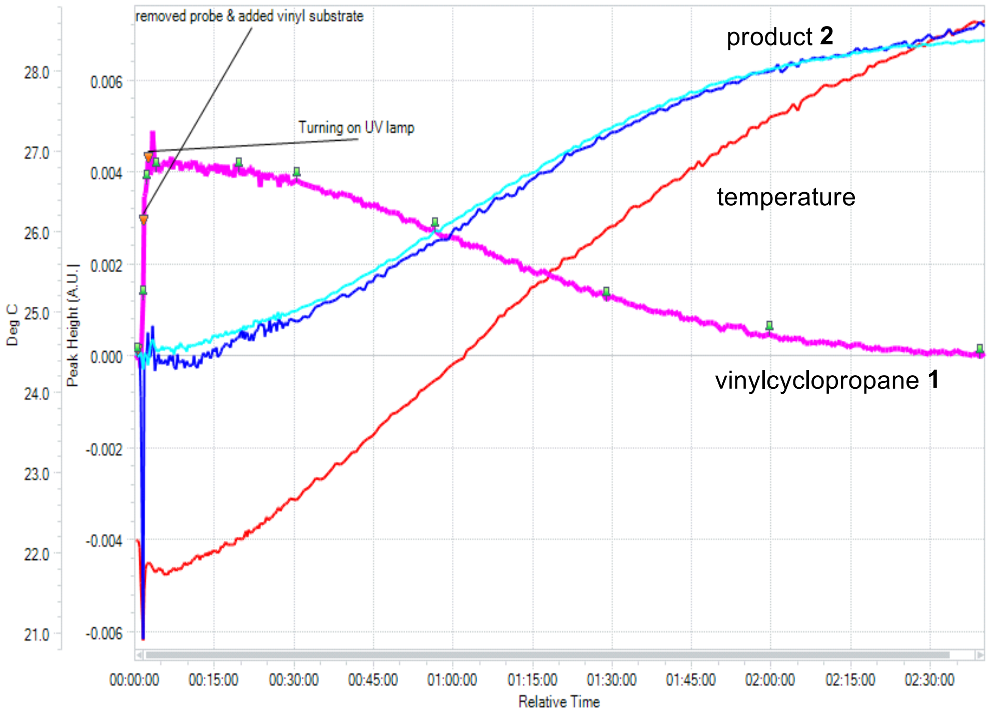

[redacted]

6. Preparation of Vinylclopropanes

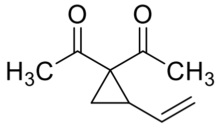

1

1,1'-(2-Vinylcyclopropane-1,1-diyl)diethanone (1):[1]

(*trans*)-1,4-Dibrombut-2-ene (4.278 g, 20 mmol, 1 equiv.) and K_2CO_3 (8.154 g, 59 mmol, 2.95 equiv.) were suspended in acetone (35 mL). 2,4-Pentanedione (2.0 mL, 20 mmol, 1.0 equiv.) was added drop wise and the mixture was heated to reflux for 14.75 h. The reaction mixture was cooled to room temperature and Et_2O (35 mL) was added. The resulting precipitate was removed via filtration and washed with Et_2O (2 x 25 mL). The solvent was removed under reduced pressure and the residue purified by column chromatography on silica gel eluting with *n*-pentane/Et_2O 4:1 (v/v) yielding 1.547 g (51%) of the desired product **1** as a colorless oil.

$\boldsymbol{R_f}$ = 0.29 (*n*-pentane/Et_2O, 4:1 (v/v)); **^{1}H-NMR** (250 MHz, $CDCl_3$) δ 5.33-5.28 (m, 2H), 5.17-5.13 (m, 1H), 2.67-2.58 (m, 1H), 2.27 (s, 3H), 2.17 (s, 3H), 1.84 (dd, *J* = 7.1, 5.3 Hz, 1H), 1.49 (dd, *J* = 8.8, 5.1 Hz, 1H) ppm; **^{13}C-NMR** (63 MHz, $CDCl_3$) δ 202.7, 202.4, 132.8, 118.9, 51.2, 32.4, 30.9, 26.9, 20.2 ppm; **IR** (ATR, neat) 3088, 3010, 1684, 1638 cm^{-1}; **GC/MS** (EI, 70 eV) m/z (%) 152 (M^+, 5), 137 (40), 109 (100), 95 (22), 91 (33), 67 (44).

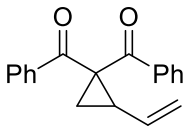

3

(2-Vinylcyclopropane-1,1-diyl)bis(phenylmethanone) (3):[2]

1,3-Diphenyl-1,3-propanedione (3.364 g, 15 mmol, 1 equiv.) was dissolved in anhydrous THF (20 mL) under an atmosphere of dry nitrogen. NaH (1.200 g, 60% in mineral oil, 30 mmol) was added as a solid and the suspension stirred for 15 min at room temperature. (*trans*)-1,4-Dibrombut-2-ene (3.158 g, 15 mmol, 1 equiv.) was added and the reaction mixture stirred for 66 h at room temperature. The resulting suspension was diluted with Et_2O and the precipitate removed via filtration and washed with Et_2O. A saturated solution of NH_4Cl (100 mL) was added. The mixture was extracted with Et_2O (2 x 150 mL) and EtOAc (100 mL). The combined organic phases were dried over $MgSO_4$ and the solvent was removed under reduced pressure. The residue was purified via column chromatography on silica gel eluting with petrol ether/EtOAc 20:1 to 10:1 (v/v) to yield a mixture of product and unreacted starting materials. Pure product was obtained via additional purification using HPLC eluting with petrol ether/EtOAc 20:1 giving 758 mg (18%) of **3** as a colorless solid.

***R**$_f$* = 0.33 (petrol ether/EtOAc, 20:1(v/v)); **^{1}H-NMR** (300 MHz, $CDCl_3$) δ 7.72-7.64 (m, 4H), 7.39-7.32 (m, 2H), 7.26-7.21 (m, 4H), 5.42-5.38 (m, 2H), 3.37-3.29 (m, 1H), 2.34 (dd, *J* = 4.4, 7.2 Hz, 1H), 1.58 (dd, *J* = 4.4, 8.6 Hz,. 1H) ppm; **^{13}C-NMR** (63 MHz, $CDCl_3$) 196.7, 196.0, 138.1, 137.7, 133.0, 132.9, 132.85, 128.6, 128.43, 128.4, 118.7, 47.7, 31.2, 21.3 ppm; **IR** (ATR, neat) 3061, 2986, 1680, 1660, 1633, 1594, 1575, 1446 cm^{-1}; **HRMS** (ESI) calc. for $[C_{19}H_{16}O_2 + Na^+]$: 299.1043, found 299.1036.

(R)-3: $[\alpha]_D^{20}$: +305.6 (c = 5.33, $CHCl_3$); **(S)-3**: $[\alpha]_D^{20}$: -305.6 (c = 5.33, $CHCl_3$).

Data File C:\HPCHEM\1\DATA\DP\DP65H200.D Sample Name: DP65h2

Chiralcel OD-H; Heptan/Isopropanol 95:5, 0.5 mL/min; 3µ
l

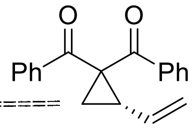

(R)-3

===

Injection Date : 27.05.15 14:26:59
Sample Name : DP65h2 Vial : 12
Acq. Operator : Dominik
Inj Volume : 3 µl

Method : C:\HPCHEM\1\METHODS\BPOD9_1.M
Last changed : 22.05.15 15:55:29 by Dominik

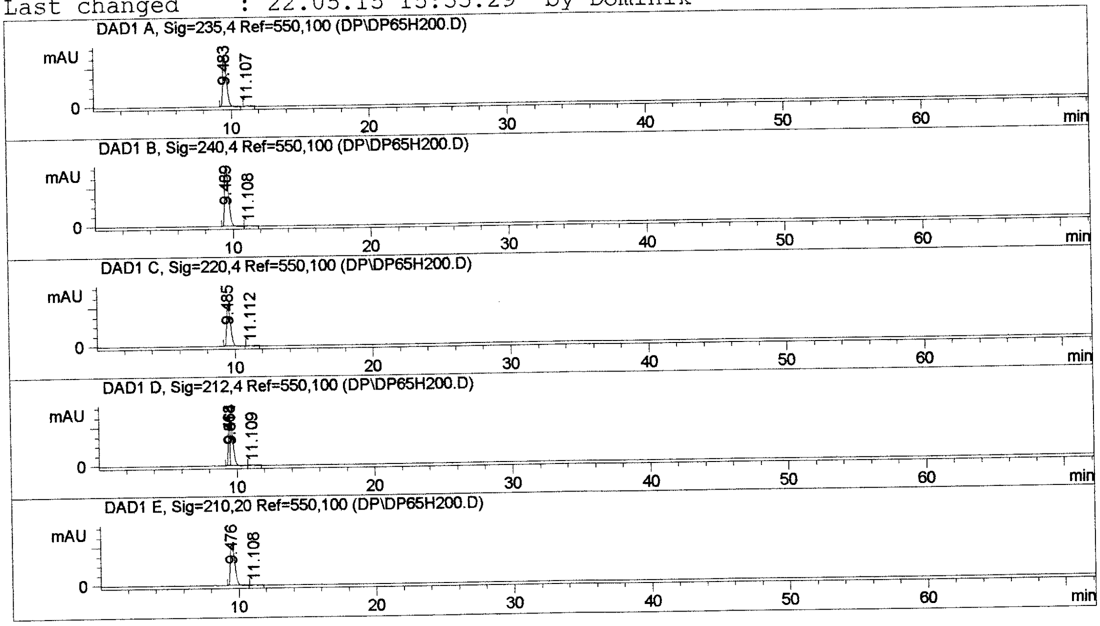

===
Area Percent Report
===

Sorted By : Signal
Multiplier : 1.0000
Dilution : 1.0000

Signal 1: DAD1 A, Sig=235,4 Ref=550,100

Peak #	RetTime [min]	Type	Width [min]	Area [mAU*s]	Height [mAU]	Area %
1	9.483	PB	0.2796	4.96877e4	2640.18140	99.4388
2	11.107	BP	0.2726	280.42484	14.31167	0.5612

Totals : 4.99682e4 2654.49306

Results obtained with enhanced integrator!

Data File C:\HPCHEM\1\DATA\DP\DP65H300.D Sample Name: DP65H3

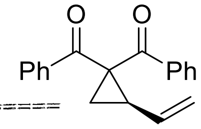

(S)-3

Chiralcel OD-H; Heptan/Isopropanol 95:5, 0.5 mL/min; 3µl

===

Injection Date : 20.05.15 15:59:54
Sample Name : DP65H3
Acq. Operator : Dominik

Vial : 12

Inj Volume : 3 µl

Acq. Method : C:\HPCHEM\1\METHODS\BPOD9_1.M
Last changed : 20.05.15 15:58:17 by Isabel
(modified after loading)
Analysis Method : C:\HPCHEM\1\METHODS\BPOD9_1.M
Last changed : 22.05.15 15:55:29 by Dominik

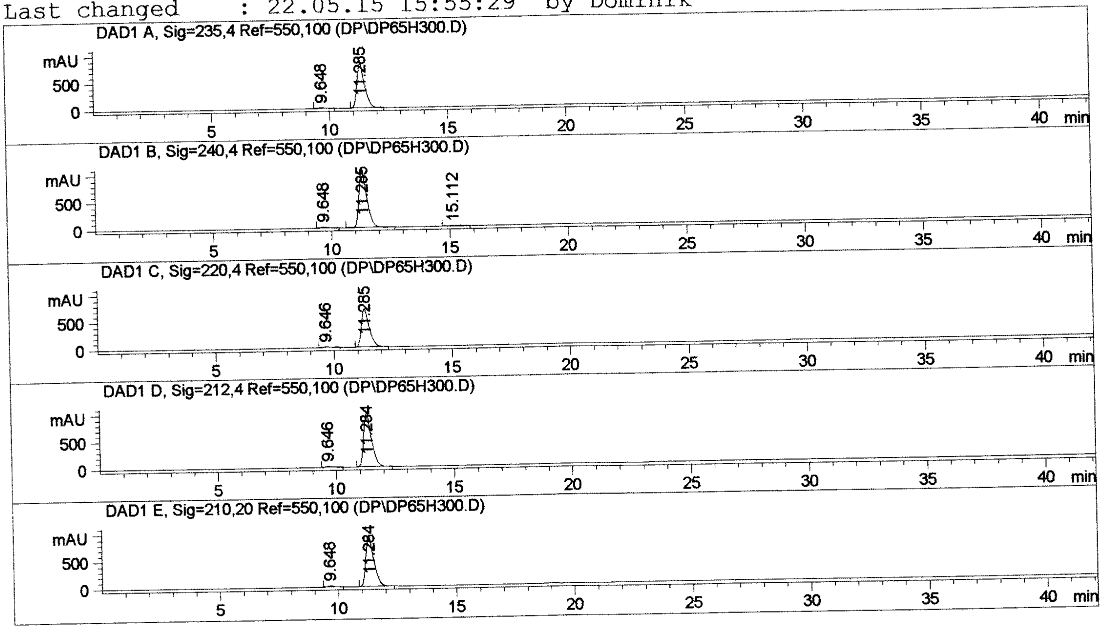

===

Area Percent Report

===

Sorted By : Signal
Multiplier : 1.0000
Dilution : 1.0000

Signal 1: DAD1 A, Sig=235,4 Ref=550,100

Peak #	RetTime [min]	Type	Width [min]	Area [mAU*s]	Height [mAU]	Area %
1	9.648	PB	0.2836	319.26849	16.65573	1.6750
2	11.285	BB	0.3328	1.87413e4	825.64966	98.3250

Totals : 1.90606e4 842.30539

Results obtained with enhanced integrator!

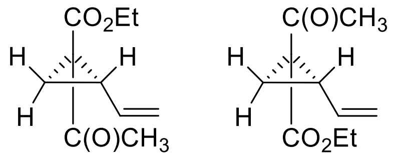

5a **5b**

(anti) and (syn) Ethyl 1-acetyl-2-vinylcyclopropanecarboxylate (5a and 5b):[1]

(*trans*)-1,4-Dibrombut-2-ene (1.390 g, 6.5 mmol) and K_2CO_3 (2.695 g, 19.5 mmol) were added to a dried flask under an atmosphere of dry nitrogen. Anhydrous ethanol (14 mL) was added followed by the drop wise addition of ethyl acetoacetate (0.8 mL, 6.5 mmol). The reaction mixture was heated to reflux for 15 h then cooled to room temperature and diluted with Et_2O (15 mL). The precipitate was removed via filtration and washed with Et_2O (2 x 10 mL). The solvent was removed under reduced pressure. The reaction gave rise to three major products: Diastereoisomer **5a** and **5b** alongside some dihydrofurane **6**. Diastereoisomer **5b** could be separated by regular column chromatography on silica gel eluting with petrol ether/EtOAc 20:1 (v/v) to give 0.457 g (39%) as a colorless oil. Diastereoisomer **5a** was purified via HPLC eluting again with petrol ether/EtOAc 20:1 (v/v) giving 0.231 g (20%) as a colorless oil. The yield of dihydrofurane **6** was not determined.

5a:

R_f = 0.29 (petrol ether/EtOAc, 20:1 (v/v)); **^{1}H-NMR** (250 MHz, $CDCl_3$) δ 5.35-5.26 (m, 2H), 5.15-5.11 (m, 1H), 4.33- 4.13 (m, 2H), 2.67-2.57 (m, 1H), 2.33 (s, 3H), 1.85 (dd, *J* = 7.5, 4.6 Hz, 1H), 1.53 (dd, *J* = 8.8, 4.6 Hz, 1H), 1.30 (t, *J* = 7.2 Hz, 3H) ppm; **^{13}C-NMR** (63 MHz, $CDCl_3$) δ 201.1, 170.4, 132.8, 118.9, 61.5, 42.7, 33.5, 30.5, 20.1, 14.1 ppm; **IR** (ATR, neat) 3088, 2984, 2929, 1702, 1637 cm^{-1}; **GC/MS** (EI, 70 eV) m/z (%) 182 (M^+, found), 139 (54), 136 (45), 135 (48), 121 (100), 94 (65), 93 (56), 67 (55), 66 (98), 65 (43).

5b:

R_f = 0.23 (petrol ether/EtOAc, 20:1 (v/v)); **^{1}H-NMR** (250 MHz, $CDCl_3$) δ 5.58-5.44 (m, 1H), 5.29 (dd, *J* = 17.0, 1.6 Hz, 1H), 5.14 (dd, *J* = 10.1, 1.6 Hz, 1H), 4.24 (q, *J* = 7.1 Hz, 2H), 2.61 (ddd, *J* = 8.4, 8.4, 8.4 Hz, 1H), 2.40 (s, 3H), 1.76 (dd, *J* = 7.7, 4.4 Hz, 1H), 1.58 (dd, *J* = 8.9, 4.4 Hz, 1H), 1.30 (t, *J* = 7.1 Hz, 3H) ppm; **^{13}C-NMR** (63 MHz, $CDCl_3$) δ 202.0, 168.8, 133.1, 118.8, 61.4, 43.2, 34.3, 29.5, 23.1, 14.2 ppm; **IR** (ATR, neat) ν 3088 , 2984 , 2938 , 1724 , 1695 , 1638 cm^{-1}; **GC/MS** (EI, 70 eV) m/z (%) 182 (M^+, found), 139 (61), 136 (43), 135 (50), 121 (100), 94 (64), 93 (56), 67 (54), 66 (97), 65 (44).

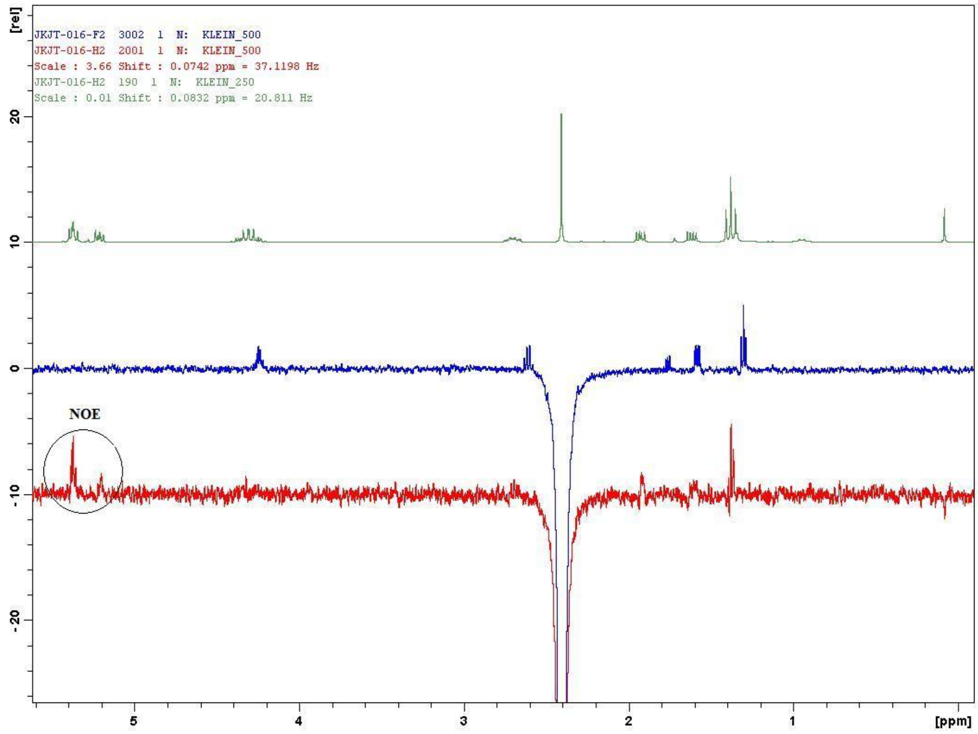

Figure S12: NOE-Spectra of **5a** (red), **5b** (blue) and the ^{1}H-spectrum of **5a** (green).

(anti) and (syn) *tert*-Butyl 1-acetyl-2-vinylcyclopropane-1-carboxylate (7a and 7b):[1]

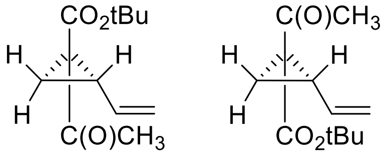

7a **7b**

NaH (0.800 g, 60% in mineral oil, 20 mmol) was suspended in anhydrous THF (16 mL) under an atmosphere of dry nitrogen. The reaction mixture was cooled to 0 °C (cryostat). *tert*-Butyl acetoacetate (1.6 mL, 9.51 mmol) was added drop wise and the reaction mixture stirred at 0 °C for 1 h. (*trans*)-1,4-Dibrombut-2-ene (2.034 g, 9.51 mmol) was added drop wise as a solution in anhydrous THF (6 mL). Upon completion of the addition the cooling bath was removed and the reaction mixture stirred for 40 h. The resulting suspension was diluted with Et_2O (20 mL) and the precipitate removed via filtration and washed with Et_2O (2 x 10 mL). The solvent was removed under reduced pressure. Diastereoisomer **7a** and **7b** alongside some dihydrofurane **8**. Diastereoisomer **7b** could be isolated via regular column chromatography on silica gel elution with petrol ether/EtOAc 20:1 (v/v) giving 0.267 g (13%) as a colorless oil. Diastereoisomer **7a** was isolated via HPLC eluting with petrol ether/EtOAc 20:1 (v/v) yielding 0.409 g (21%) as a colorless oil. The yield of dihydrofurane **8** was not determined.

7a:

$\boldsymbol{R_f}$ = 0.37 (petrol ether/EtOAc, 20:1 (v/v)), ^{1}**H-NMR** (250 MHz, $CDCl_3$) δ 5.30-5.25 (m, 2H), 5.14-5.09 (m, 1H), 2.59-2.49 (m, 1H), 2.29 (s, 3H), 1.76 (dd, *J* = 7.5, 4.5 Hz, 1H), 1.49 (s, 9H), 1.45 (dd, *J*

= 8.8, 4.6 Hz, 1H) ppm; **^{13}C-NMR** (63 MHz, $CDCl_3$) δ 201.4, 169.4, 133.1, 118.6, 82.3, 43.7, 32.8, 30.4, 28.0, 19.8 ppm; **IR** (ATR, neat) 2978, 2932, 1701, 1637 cm^{-1}; **GC/MS** (EI, 70 eV) m/z (%) 210 (M^+, found), 154 (50), 135 (32), 121 (58), 111 (45), 94 (60), 66 (37), 57 (100).

7b:

R_f = 0.29 (petrol ether/EtOAc, 20:1 (v/v)); **^{1}H-NMR** (250 MHz, $CDCl_3$) δ 5.59-5.45 (m, 1H), 5.29 (dd, *J* = 17.2, 1.6 Hz, 1H), 5.14 (dd, *J* = 10.1, 1.6 Hz, 1H), 2.58 (ddd, *J* = 8.3, 8.3, 8.3 Hz, 1H), 2.40 (s, 3H), 1.69-1.62 (m, 1H), 1.51-1.46 (m, 1H), 1.49 (s, 9H) ppm; **^{13}C-NMR** (63 MHz, $CDCl_3$) δ 202.4, 167.8, 133.3, 118.4, 82.2, 44.2, 33.6, 29.5, 28.1, 22.9 ppm; **IR** (ATR, neat) 3087, 2978, 2934, 1719, 1696, 1638 cm^{-1}; **GC/MS** (EI, 70 eV) m/z (%) 210 (M^+, found), 154 (47), 135 (32), 121 (57), 111 (48), 94 (57), 93 (29), 66 (37), 57 (100).

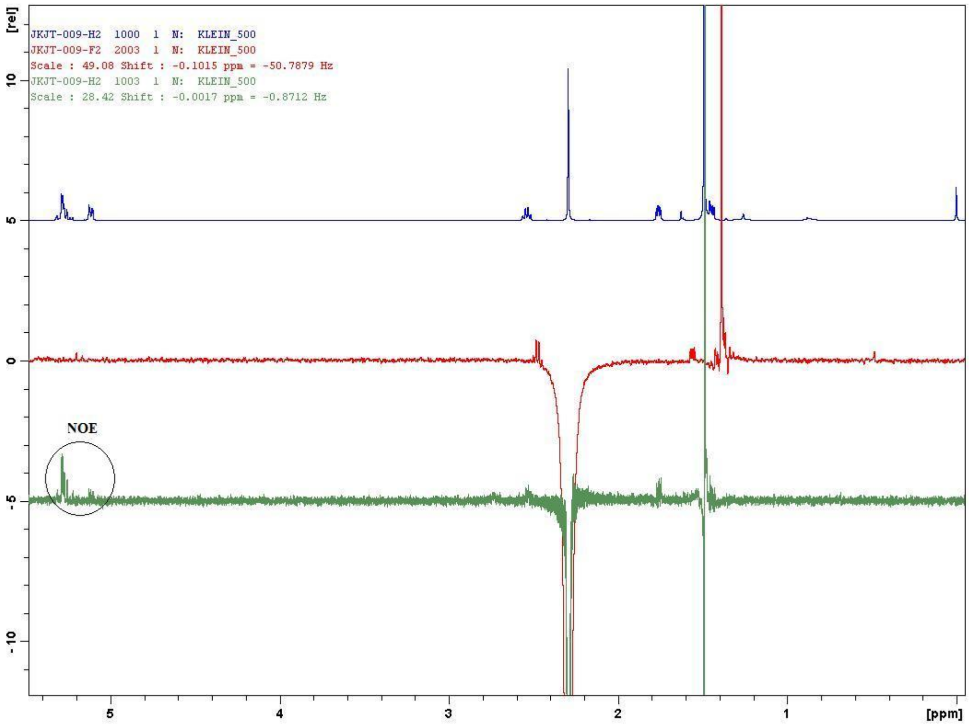

Figure S13: NOE-Spectra of **7a** (green), **7b** (red) and the ^{1}H-spectrum of **7a** (blue).

(anti) and (syn) Ethyl 1-benzoyl-2-vinylcyclopropane-1-carboxylate (9a and 9b):[3]

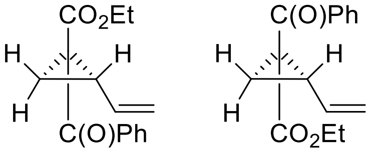

9a **9b**

Ethyl benzoylacetate (2.883 g, 15 mmol, 1 equiv.) was dissolved in anhydrous EtOH (20 mL) under an atmosphere of dry nitrogen. K_2CO_3 (4.146 g, 30 mmol, 2 equiv.) was added and the suspension stirred at room temperature for 15 min. Additional anhydrous EtOH (10 mL) was added prior to the addition of (*trans*)-1,4-dibrombut-2-ene (3.158 g, 15 mmol, 1 equiv.). The reaction mixture was stirred for 95 h

at room temperature. The reaction mixture was diluted with Et_2O and the precipitate removed via filtration and washed with additional Et_2O. The solvent was removed under reduced pressure. The residue was portioned between H_2O (30 mL) and Et_2O (40 mL). The Aqueous layer was extracted with Et_2O (3 x 40 mL). The combined organic extracts were dried over $MgSO_4$ and the solvent was removed under reduced pressure. The residue was purified via column chromatography on silica gel eluting with n-pentane/Et_2O 20:1 (v/v) followed by HPLC eluting with petrol ether/EtOAc 20:1 (v/v) giving 1.374 g (37%) of diastereoisomer **9b** as a colorless oil and 936 mg (26%) of diastereoisomer **9a** as a colorless oil. The assignment of the diastereoisomers is based on the assignment of the ethyl and *tert*-butyl esters above.

9a:

R_f = 0.33 (*n*-pentane/Et_2O, 20:1 (v/v)); **^{1}H-NMR** (300 MHz, $CDCl_3$) δ 7.84-7.80 (m, 2H), 7.55-7.750 (m, 1H), 7.45-7.40 (m, 1H), 5.33 (dd, J = 2.0, 16.9 Hz, 1H), 5.25-5.13 (m, 1H), 5.01 (dd, J = 2.0, 10.0 Hz, 1H), 4.14-3.90 (m, 2H), 2.96-2.88 (m, 1H), 1.95 (dd, J = 4.6, 7.4 Hz, 1H), 1.62 (dd, J = 4.6, 8.7 Hz, 1H), 0.92 (t, J = 7.2 Hz, 3H) ppm; **^{13}C-NMR** (75 MHz, $CDCl_3$) δ 193.7, 171.0, 137.7, 133.2, 132.8, 128.5, 128.4, 118.6, 61.5, 39.8, 32.9, 19.6, 13.6 ppm; **IR** (ATR, neat) 2982, 1722, 1677, 1638, 1598, 1580, 1449 cm^{-1}; **HRMS** (ESI) calc. for [$C_{15}H_{16}O_3$ + Na^+]: 267.0992, found 267.0977.

9b:

R_f = 0.33 (*n*-pentane/Et_2O, 20:1 (v/v)); **^{1}H-NMR** (300 MHz, $CDCl_3$) δ 7.88-7.85 (m, 2H), 7.57-7.52 (m, 1H), 7.46-7.41 (m, 2H), 5.83-5.71 (m, 1H), 5.43-5.37 (m, 1H), 5.23-5.19 (m, 1H), 4.00 (q, J = 7.4 Hz, 2H), 2.79-2.71 (m, 1H), 1.94 (dd, J = 4.8, 7.7 Hz, 1H), 1.66 (dd, 4.8, 9.0 Hz, 1H), 0.90 (t, J = 7.1 Hz, 3H) ppm; **^{13}C-NMR** (75 MHz, $CDCl_3$) δ 194.5, 169.3, 137.3, 133.1, 132.8, 128.5, 128.2, 118.8, 61.3, 40.6, 30.4, 21.6, 13.7 ppm; **IR** (ATR, neat) 3086, 2982, 1727, 1678, 1637, 1598, 1581, 1448 cm^{-1}; **HRMS** (ESI) calc. for [$C_{15}H_{16}O_3$ + Na^+]: 267.0992, found 267.0980.

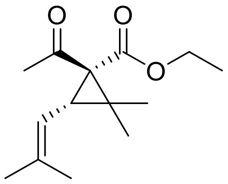

17

Ethyl 1-acetyl-2,2-dimethyl-3-(2-methylprop-1-en-1-yl)cyclopropane-1-carboxylate (17):

A solution of diisopropylamine (1.55 mL, 11 mmol, 1.1 equiv.) in THF (20 mL) was cooled to -78 °C and 1.6 M *n*-BuLi (6.87 mL, 11 mmol, 1.1 equiv.) was added dropwise. The reaction mixture was warmed to room temperature for 15 min, then re-cool to -78 °C. Ethyl chrysanthemate (2.17 mL, 10 mmol, 1 equiv.) was added dropwise in to the reaction mixture and stirred for 30 min at -78 °C then 1 h at room temperature. The reaction mixture was re-cooled to -78 °C again and acetyl chloride (0.71 mL, 10 mmol, 1 equiv.) was added dropwise. The reaction was allowed to warm to room temperature and stirred overnight. The reaction was quenched by sat. NH_4Cl, diluted with water, and extract with three time of diethyl ether. The combined organic layers were dried over $MgSO_4$, filtered and then concentrated under reduced pressure. The residue was purified by column chromatography on silica gel eluting with *n*-pentane/EA 40:1 (v/v) to afford 1.3345 g (56%) of **17** as a colorless oil.

***R**$_f$* = 0.37 (*n*-pentane /EA, 40:1 (v/v)); **^{1}H-NMR** (300 MHz, $CDCl_3$) δ 5.03-5.00 (m , 1H), 4.19 (q, *J* = 7.1 Hz, 2H), 2.58 (d, *J* = 8.0 Hz, 1H), 2.27 (s, 3H), 1.73 (s, 3H), 1.71 (s, 3H), 1.33 (s, 3H), 1.26 (t, *J* = 7.1 Hz, 3H), 1.11 (s, 3H) ppm; **^{13}C-NMR** (75 MHz, $CDCl_3$) δ 201.3, 168.6, 137.1, 117.1, 60.8, 51.2, 34.8, 33.4, 30.2, 25.7, 22.0, 18.7, 18.0, 14.1 ppm; **IR** (ATR) 2980, 2929, 1733, 1700, 1446, 1377, 1355, 1304, 1274, 1178, 1095, 852 cm^{-1}; **HRMS** (ESI) calc. for $[C_{14}H_{22}O_3 + Na^+]$: 261.1461, found: 261.1452.

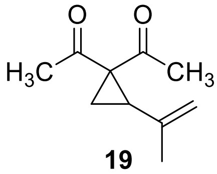

19

1,1'-(2-(Prop-1-en-2-yl)cyclopropane-1,1-diyl)bis(ethan-1-one) (19): [1]

1,4-Dibromo-2-methylbut-2-ene (1,59 g, 7 mmol, 1 equiv.) and K_2CO_3 (2,90 g, 21 mmol, 3 equiv.) were suspended in acetone (20 mL). 2,4-Pentanedione (0,7 mL, 7 mmol, 1.0 equiv.) was added drop wise and the mixture was heated to reflux for 18 h. The reaction mixture was cooled to room temperature and Et_2O (35 mL) was added. The resulting precipitate was removed via filtration and washed with Et_2O (2 x 25 mL). The solvent was removed under reduced pressure and the residue purified by column chromatography on silica gel eluting with *n*-pentane/Et_2O 4:1 (v/v) yielding 378 mg (32%) of the desired product **19** as a yellow oil.

***R**$_f$* = 0.38 (*n*-pentane/Et_2O, 4:1 (v/v)); **^{1}H-NMR** (250 MHz, $CDCl_3$) δ 4.94 (d, *J* = 0.8 Hz, 1H), 4.75 (s, 1H), 2.57 (t, *J* = 8.4 Hz, 1H), 2.23 (d, *J* = 1.2 Hz, 3H), 2.20 (d, *J* = 1.2 Hz, 3H), 1.94 (ddd, *J* = 8.0, 5.1, 1.0 Hz, 1H), 1.76 (d, *J* = 0.5 Hz, 3H), 1.50 – 1.41 (m, 1H) ppm; **^{13}C-NMR** (63 MHz, $CDCl_3$) δ 203.2, 202.5, 138.6, 113.9, 51.2, 35.9, 30.7, 27.8, 23.0, 19.2 ppm; **IR** (ATR) 2973, 2918, 1688, 1423, 1357, 1309, 1256 cm^{-1}; **GC/MS** (EI, 70 eV) m/z (%)166 (M^+, found), 151 (14), 123 (67), 43 (100); **HRMS** (EI) calc. for $[C_{10}H_{14}O_2]$: 166.0994, found: 166.0991.

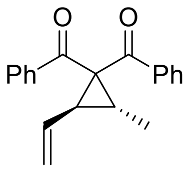

23

(2-Methyl-3-vinylcyclopropane-1,1-diyl)bis(phenylmethanone) (23):[1]

The *title compound* was synthesized according to literature[1] and was purified via column chromatography on silica gel eluting with PE/EA 40:1 (v/v) to afford **23** as a white solid.

***R**$_f$* = 0.34 (PE/EA 40:1 (v/v)); **mp** 108-111°C; **^{1}H-NMR** (300 MHz, $CDCl_3$) δ 7.73-7.66 (m, 1H), 7.66-7.59 (m, 1H), 7.42-7.30 (m, 2H), 7.30-7.18 (m, 4H) 5.42-5.32 (m, 2H), 5.06-4.98 (m, 1H) 3.12-3.03 (m, 1H) 2.84-2.73 (m, 1H), 1.12 (d, *J* = 6.4 Hz, 3H) ppm; **^{13}C-NMR** (75 MHz, $CDCl_3$) δ 196.5, 196.1,

138.6, 138.5, 133.3, 132.9, 132.8, 128.55, 128.53, 128.49, 128.42, 118.1, 53.6, 37.4, 27.3, 11.9 ppm; **IR** (ATR) 3062, 3027, 1720, 1656, 1490, 1346, 1310, 1125, 848, 761 cm^{-1}.

General Procedure I: Preparation of Olefin-Substituted VCPs[1]

The appropriate vinylcyclopropane (1 equiv.) and 2nd generation Grubbs catalyst (0.015 equiv.) was weighed into a dried Schlenk tube under an atmosphere of dry nitrogen. CH_2Cl_2 (0.6 M) was added followed by the appropriate alkene (10 equiv.). The Schlenk tube was sealed under an atmosphere of dry nitrogen and the mixture stirred at 45 °C for 16 h. The solvent was removed under reduced pressure and the residue subjected to column chromatography on silica gel.

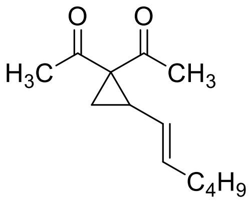

11

(*E*)-1,1'-(2-(Hex-1-en-1-yl)cyclopropane-1,1-diyl)diethanone (11):

The reaction was carried out on a 5 mmol scale based on vinylcyclopropane **1**. The *title compound* was purified via column chromatography on silica gel eluting with *n*-pentane/Et_2O 4:1 (v/v) to afford 511 mg (49%) of **11** as a tan oil.

R$_f$ = 0.39 (*n*-pentane/Et_2O, 4:1 (v/v)); **^{1}H NMR** (300 MHz, $CDCl_3$) δ 5.80 – 5.66 (m, 1H), 4.93 (dd, *J* = 15.3, 8.5 Hz, 1H), 2.57 (q, *J* = 8.3 Hz, 1H), 2.26 (s, 3H), 2.15 (s, 3H), 1.98 (m, 2H), 1.80 (dd, *J* = 7.4, 5.1 Hz, 1H), 1.47 (dd, *J* = 8.8, 5.1 Hz, 1H), 1.34 – 1.25 (m, 4H), 0.88 (m, 3H) ppm; **^{13}C NMR** (75 MHz, $CDCl_3$) δ 203.1, 136.0, 124.1, 51.2, 32.3, 32.1, 31.3, 30.9, 26.8, 22.1, 20.4, 13.8 ppm; **IR** (ATR) 2958, 2928, 2873, 2855, 1681, 1422, 1357, 1310, 1250, 1163, 1100, 968, 936 cm^{-1}; **GC/MS** (ESI) m/z (%) = 209.15 (M + H^+, 28), 191 (15), 167 (16), 149 (20), 135 (13), 123 (23), 113 (100); **HRMS** (ESI) calc. for [$C_{12}H_{20}O_2$ + Na^+]: 231.1356, found: 231.1346.

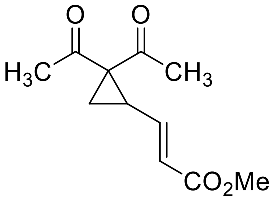

13

Methyl (*E*)-3-(2,2-diacetylcyclopropyl)acrylate (13):

The reaction was carried out on a 5 mmol scale based on vinylcyclopropane **1**. The *title compound* was purified via column chromatography on silica gel eluting with *n*-pentane/Et_2O 2:1 (v/v) to afford 450 mg (43%) of **13** as a tan oil.

R_f = 0.19 (*n*-pentane/Et_2O, 2:1 (v/v)); **^{1}H NMR** (300 MHz, $CDCl_3$) δ 6.34 (dd, *J* = 15.5, 9.9 Hz, 1H), 6.05 (d, *J* = 15.5 Hz, 1H), 3.72 (s, 3H), 2.77 – 2.64 (m, 1H), 2.29 (s, 3H), 2.22 (s, 3H), 1.91 (dd, *J* = 7.0, 5.1 Hz, 1H), 1.59 (dd, *J* = 8.6, 5.1 Hz, 1H) ppm; **^{13}C NMR** (75 MHz, $CDCl_3$) δ 201.6, 201.4, 165.9, 143.2, 124.0, 51.7, 51.7, 30.7, 30.5, 27.5, 21.4 ppm; **IR** (ATR) 2954, 2919, 2849, 1722, 1686, 1651, 1435, 1358, 1308, 1273, 1252,1204, 1148 cm^{-1}; **GC/MS** (ESI) m/z (%) = 211 (M + H^+, 6), 137 (100), 119 (18), 111 (45); **HRMS** (ESI) calc. for [$C_{11}H_{14}O_4$ + Na^+]: 233.0784, found: 233.0786.

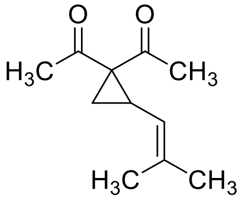

15

1,1'-(2-(2-Methylprop-1-en-1-yl)cyclopropane-1,1-diyl)diethanone (15):

The reaction was carried out on 6 mmol scale based on vinylcyclopropane **1**. The *title compound* was purified via column chromatography on aluminium oxide (neutral) eluting with *n*-pentane/Et_2O 4:1 (v/v) to afford 854 mg (79%) of **15** as a pale yellow oil.

R_f = 0.34 (*n*-pentane/Et_2O, 4:1 (v/v)); **^{1}H-NMR** (300 MHz, $CDCl_3$) δ 4.66-4.59 (m, 1H), 2.67 (dd, *J* = 8.6, 16.4 Hz, 1H), 2.22 (s, 3H), 2.18 (s, 3H), 1.78-1.72 (m, 4H), 1.69 (s, 3H), 1.52 (dd, *J* = 4.8, 8.9 Hz, 1H) ppm; **^{13}C-NMR** (75 MHz, $CDCl_3$) δ 203.3, 203.2, 138.5, 119.2, 51.0, 30.5, 29.2, 27.2, 25.6, 21.7, 18.4 ppm; **IR** (ATR) 2916, 1681, 1620, 1451, 1357, 1303, 1252, 1171, 1103, 933, 855, 823 cm^{-1}; **GC/MS** (ESI) m/z (%) = 181 (M + H^+, 15), 163 (18), 145 (35), 121 (100), 113 (17), 105 (23); **HRMS** (ESI) calc. for [$C_{11}H_{16}O_2$ + Na^+]: 203.1043, found: 203.1046.

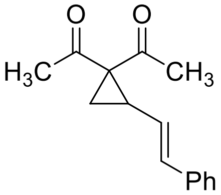

21

1,1'-(2-Styrylcyclopropane-1,1-diyl)bis(ethan-1-one) (21)

The reaction was carried out on a 7 mmol scale based on vinylcyclopropane **1**. Styrene (210 mmol, 24 mL, 30 equiv.) was used as the source of alkene. The *title compound* was purified via column chromatography on silica gel eluting with petrol ether/EtOAc 5:1 (v/v) followed by semi-prep. HPLC eluting with petrol ether/EtOAc 5:1 (v/v) to afford 340 mg (21%) of **21** as a tan oil.

R_f = 0.25 (petrol ether/EtOAc, 5:1 (v/v)); **^{1}H NMR** (300 MHz, $CDCl_3$) δ 7.31 – 7.19 (m, 5H), 6.65 (d, *J* = 15.8 Hz, 1H), 5.66 (dd, *J* = 15.8, 9.0 Hz, 1H), 2.78 (dd, *J* = 16.3, 8.7 Hz, 1H), 2.28 (s, 3H), 2.21 (s, 3H), 1.96 (dd, *J* = 7.3, 5.1 Hz, 1H), 1.59 (dd, *J* = 8.7, 5.1 Hz, 1H) ppm; **^{13}C NMR** (75 MHz, $CDCl_3$) δ

202.68, 202.65, 136.38, 134.06, 128.64, 127.80, 126.14, 124.30, 51.62, 32.72, 30.98, 27.12, 21.07 ppm; **IR** (ATR) 3026, 1682, 1598, 1492, 1448, 1358 cm^{-1}; **GC/MS** (ESI) m/z (%) = 251 (100) [M^+ + Na]; **HRMS** (ESI) calc. for [$C_{15}H_{16}O_2$ + Na^+]: 251.1043, found: 251.1030.

7. Preparation of Arylcyclopropanes

General Procedure II: Preparation of Arylcyclopropanes

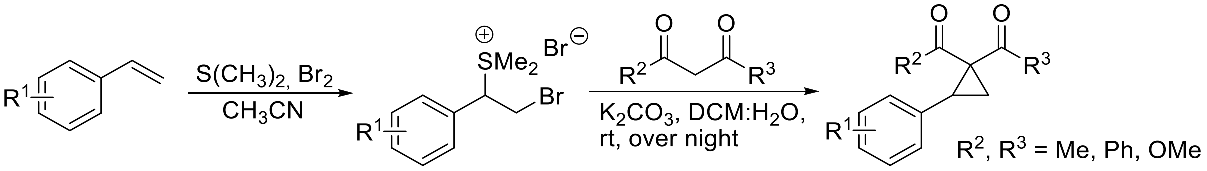

The procedure was modified from the literature.[4] A solution of bromine (1.02 mL, 20 mmol) in CH_2Cl_2 (5 mL) was added in a solution of dimethyl sulfide (7.34 mL, 100 mmol) in CH_3CN (20 mL) at 0 °C to give a yellow precipitate. The corresponding styrene derivative (20 mmol) was then added at the same temperature. The reaction mixture was allowed to warm to room temperature and stirred for 2 hours. Diethyl ether (30 mL) was added to give a precipitate, which was then filtered and washed with diethyl ether to give the corresponding bromosulfonium bromide without further purification.

The bromosulfonium bromide (10 mmol) and potassium carbonate (4.15 g, 30 mmol) were dissolved in CH_2Cl_2:H_2O (1:1) mixture (200 mL). Pentane-2,4-dione (2.05 mL, 20 mmol), 1,3-diphenyl-1,3-propanedione (4.49 g, 20 mmol) or methyl acetoacetate (2.15 mL, 20 mmol) was added and stirred at room temperature overnight. The CH_2Cl_2 layer was separated and the aqueous layer was extracted with 3 portions of CH_2Cl_2. The combined organic layers were dried over $MgSO_4$, filtered and then concentrated under reduced pressure. The residue was purified by column chromatography on silica gel eluting with *n*-pentane/Et_2O (5:1 to 2:1) to give the desired arylcyclopropane.

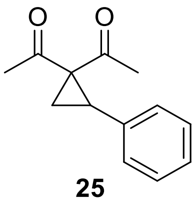

25

1,1'-(2-Phenylcyclopropane-1,1-diyl)bis(ethan-1-one) (25):[6]

The *title compound* was purified via column chromatography on silica gel eluting with *n*-pentane/Et_2O 5:1 (v/v) to afford 656 mg (22% two steps overall yield) of **25** as a colorless solid.

R$_f$ = 0.23 (*n*-pentane/Et_2O, 5:1 (v/v)); **mp** 61-62 °C [lit. 58 °C]; **^{1}H-NMR** (300 MHz, $CDCl_3$) δ 7.31-7.21 (m, 3H), 7.15-7.12 (m, 2H), 3.29 (bt, *J* = 8.5 Hz, 1H), 2.28 (s, 3H), 2.26 (dd, *J* = 5.3, 7.9 Hz, 1H), 1.81 (s, 3H), 1.65 (dd, *J* = 5.3, 9.0 Hz, 1H) ppm; **^{13}C-NMR** (75 MHz, $CDCl_3$) δ 202.7, 202.2, 134.1, 128.5, 128.4 , 127.5, 52.7, 33.6, 30.4, 27.7, 19.1 ppm; **IR** (ATR) 3098, 3065, 3020, 1702, 1681, 1453 cm^{-1}; **HRMS** (ESI): calc. for [$C_{13}H_{14}O_2$ + Na^+]: 225.0886, found: 225.0871.

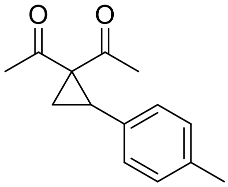

27

1,1'-(2-(*p*-Tolyl)cyclopropane-1,1-diyl)bis(ethan-1-one)) (27):[6]

The *title compound* was purified via column chromatography on silica gel eluting with *n*-pentane/Et_2O 3:1 (v/v) to afford 1000 mg (23% two steps overall yield) of **27** as a yellow solid.

***R**$_f$* = 0.38 (*n*-pentane /Et_2O, 3:1 (v/v)); **mp** 52-54 °C [lit.57.6-59 °C]; **^{1}H NMR** (300 MHz, $CDCl_3$) δ 7.10 (m, 2H), 7.06 – 7.01 (m, 2H), 3.27 (t, *J* = 8.5 Hz, 1H), 2.32 (s, 3H), 2.30 – 2.23 (m, 4H), 1.84 (s, 3H), 1.67 (dd, *J* = 8.0, 4.3 Hz, 1H) ppm; **^{13}C NMR** (75 MHz, $CDCl_3$) δ (ppm) 202.9, 202.4, 137.3, 130.9, 129.2, 128.3, 52.7, 33.6, 30.5, 27.6, 21.1, 19.1 ppm; **IR** (ATR) 3013, 2923, 1681, 1517, 1425, 1356 cm^{-1}; **HRMS** (ESI): calc. for [$C_{14}H_{16}O_2$ + Na^+]: 239.1043, found: 239.1033.

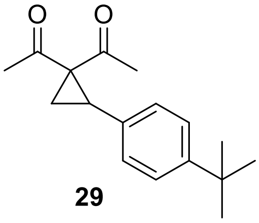

29

1,1'-(2-(4-(*tert*-Butyl)phenyl)cyclopropane-1,1-diyl)bis(ethan-1-one) (29):[5]

The *title compound* was purified via column chromatography on silica gel eluting with *n*-pentane/Et_2O 3:1 (v/v) to afford 504 mg (8% two steps overall yield) of **29** as a colorless solid.

***R**$_f$* = 0.35 (*n*-pentane /Et_2O, 3:1 (v/v)); **mp** 51-53 °C; **^{1}H NMR** (300 MHz, $CDCl_3$) δ 7.31 – 7.27 (m, 2H), 7.08 – 7.03 (m, 2H), 3.25 (t, *J* = 8.5 Hz, 1H), 2.27 (s, 3H), 2.24 (dd, *J* = 8.0, 5.3 Hz, 1H), 1.82 (s, 3H), 1.65 (dd, *J* = 9.1, 5.3 Hz, 1H), 1.28 (s, 9H) ppm; **^{13}C NMR** (75 MHz, $CDCl_3$) δ 202.9, 202.6, 150.6, 130.9, 128.0, 125.5, 52.7, 34.4, 33.5, 31.3, 30.5, 27.7, 19.2 ppm; **IR** (ATR) 2962, 2869, 1684, 1518, 1425, 1359 cm^{-1}; **HRMS** (ESI): calc. for [$C_{17}H_{22}O$ + Na^+]: 281.1512, found: 281.1494.

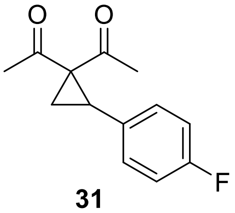

31

1,1'-(2-(4-Fluorophenyl)cyclopropane-1,1-diyl)bis(ethan-1-one) (31):

The *title compound* was purified via column chromatography on silica gel eluting with *n*-pentane/Et_2O 3:1 (v/v) to afford 815 mg (37% two steps overall yield) of **31** as a colorless oil.

***R*$_f$** = 0.17 (*n*-pentane/Et_2O, 3:1 (v/v));**^{1}H-NMR** (300 MHz, $CDCl_3$) δ 7.15-7.07 (m, 2H), 7.02-6.93 (m, 2H), 3.27 (t, *J* = 8.5 Hz, 1H), 2.28 (s, 3H), 2.22 (dd, *J* = 5.4, 7.9 Hz, 1H), 1.84 (s, 3H), 1.64 (dd, *J* = 5.4, 9.1 Hz, 1H) ppm; **^{13}C-NMR** (75 MHz, $CDCl_3$) δ 202.6, 201.9, 162.1 (d, *J* = 245.2 Hz), 130.0 (d, *J* = 8.1 Hz), 129.9 (d, *J* = 3.24 Hz), 115.5 (d, *J* = 21.5 Hz), 52.6, 32.8, 30.5, 27.8, 19.3 ppm; **IR** (ATR) 3010, 1684, 1605, 1512, 1430, 1359, 1224, 842 cm^{-1}; **HRMS** (ESI) calc. for [$C_{13}H_{13}FO_2$ + Na^+]: 243.0792, found: 243.0802.

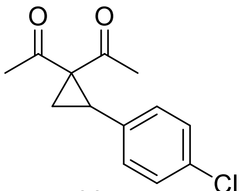

33

1,1'-(2-(4-Chlorophenyl)cyclopropane-1,1-diyl)bis(ethan-1-one) (33):[6]

The *title compound* was purified via column chromatography on silica gel eluting with *n*-pentane/Et_2O 2:1 (v/v) to afford 899 mg (38% two steps overall yield) of **33** as a colorless solid.

***R*$_f$** = 0.28 (*n*-pentane/Et_2O, 2:1 (v/v)); **mp** 77-78 ^oC [lit. 76.6-77.5 ^oC]; **^{1}H-NMR** (300 MHz, $CDCl_3$) δ 7.29-7.22 (m, 2H), 7.10-7.04 (m, 2H), 3.26 (t, *J* = 8.5 Hz, 1H), 2.28 (s, 3H), 2.22 (dd, *J* = 5.3, 7.8 Hz, 1H), 1.86 (s, 3H), 1.64 (dd, *J* = 5.4, 9.1 Hz, 1H) ppm; **^{13}C-NMR** (75 MHz, $CDCl_3$) δ 202.4, 201.7, 133.4, 132.7, 129.7, 128.7, 52.7, 32.8, 30.5, 27.8, 19.1 ppm; **IR** (ATR) 3087, 3053, 1705, 1670, 1593, 1494, 1404, 1373, 1104, 851, 592, 560 cm^{-1}; **HRMS** (ESI) calc. for [$C_{13}H_{13}ClO_2$ + Na^+]: 259.0496, found: 259.0485.

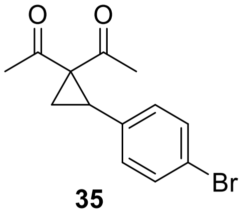

35

1,1'-(2-(4-Bromophenyl)cyclopropane-1,1-diyl)bis(ethan-1-one) (35):[6]

The *title compound* was purified via column chromatography on silica gel eluting with *n*-pentane/Et_2O 3:1 (v/v) to afford 534 mg (19% two steps overall yield) of **35** as a colorless solid.

***R*$_f$** = 0.3 (*n*-pentane/Et_2O, 3:1 (v/v)); **mp** 75-76 ^oC [lit. 72.2-73.2 ^oC]; **^{1}H-NMR** (300 MHz, $CDCl_3$) δ 7.45-7.37 (m, 2H), 7.06-6.97 (m, 2H), 3.24 (t, *J* = 8.5, 1H), 2,28 (s, 3H), 2,22 (dd, *J* = 5.4, 7.8 Hz, 1H), 1.86 (s, 3H), 1.63 (dd, *J* = 5.4, 9.0 Hz, 1H) ppm; **^{13}C-NMR** (75 MHz, $CDCl_3$) δ 202.4, 201.7, 133.3, 131.7, 130.1, 121.6, 52.7, 32.8, 30.5, 27.8, 19.1 ppm; **IR** (ATR) 3008, 1681, 1491, 1357, 1027, 822 cm^{-1}; **HRMS** (ESI) calc. for [$C_{13}H_{13}BrO_2$ + Na^+]: 280.0099, found: 280.0103.

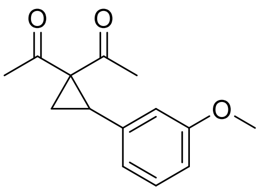

37

1,1'-(2-(3-Methoxyphenyl)cyclopropane-1,1-diyl)bis(ethan-1-one) (37):

The *title compound* was purified via column chromatography on silica gel eluting with *n*-pentane/Et_2O 2:1 (v/v) to afford 813 mg (35% two steps overall yield) of **37** as a colorless oil.

***R**$_f$* = 0.21 (*n*-pentane/Et_2O, 2:1 (v/v)); **^{1}H-NMR** (300 MHz, $CDCl_3$) δ 7.19 (t, *J* = 7.92 Hz, 1H), 6.81-6.75 (m, 1H), 6.74-6.65 (m, 2H), 3.78 (s, 3H) 3.26 (t, *J* = 8.5 Hz, 1H), 2.28 (s, 3H), 2.22 (dd, *J* = 5.3, 7.9 Hz, 1H), 1.84 (s, 3H), 1.64 (dd, *J* = 5.3, 9.0 Hz, 1H) ppm; **^{13}C-NMR** (75 MHz, $CDCl_3$) δ 202.7, 202.3, 159.6, 135.8, 129.5, 120.6, 114.1, 113.0, 55.2, 52.6, 33.5, 30.4, 27.7, 19.3 ppm; **IR** (ATR) 3006, 2926, 2838, 1683, 1601, 1584, 1492, 1358, 1256, 783, 721 cm^{-1}; **HRMS** (ESI) calc. for [$C_{14}H_{16}O_3$ + Na^+]: 255.0992, found: 255.0981.

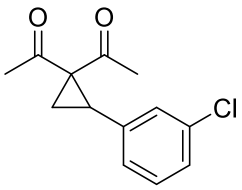

39

1,1'-(2-(3-Chlorophenyl)cyclopropane-1,1-diyl)bis(ethan-1-one) (39):

The *title compound* was purified via column chromatography on silica gel eluting with *n*-pentane/Et_2O 3:1 (v/v) to afford 402 mg (17% two steps overall yield) of **39** as a colorless oil.

***R**$_f$* = 0.24 (*n*-pentane/Et_2O, 3:1 (v/v)); **^{1}H-NMR** (300 MHz, $CDCl_3$) δ 7.24-7.19 (m, 2H), 7.18-7.14 (m, 1H), 7.04-6.96 (m, 1H), 3.26 (t, *J* = 8.5 Hz, 1H), 2.29 (s, 3H), 2.22 (dd, *J* = 5.3, 7.9 Hz, 1H), 1.88 (s, 3H), 1.64 (dd, *J* = 5.4, 9.1 Hz, 1H) ppm; **^{13}C-NMR** (75 MHz, $CDCl_3$) δ 202.3, 201.6, 136.4, 134.4, 129.7, 128.7, 127.8, 126.5, 52.6, 32.7, 30.5, 27.8, 19.1 ppm; **IR** (ATR) 3009, 2927, 1687, 1598, 1421, 1309, 781, 699 cm^{-1}; **HRMS** (ESI) calc. for [$C_{13}H_{13}ClO_2$ + Na^+]: 259.0496, found: 259.0483.

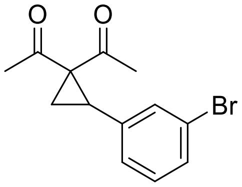

41

1,1'-(2-(3-Bromophenyl)cyclopropane-1,1-diyl)bis(ethan-1-one) (41):

The *title compound* was purified via column chromatography on silica gel eluting with *n*-pentane/Et_2O 3:1 (v/v) to afford 712 mg (13% two steps overall yield) of **41** as a colorless oil.

R$_f$ = 0.33 (*n*-pentane /Et_2O, 3:1 (v/v)); **^{1}H NMR** (300 MHz, $CDCl_3$) δ 7.40 – 7.35 (m, 1H), 7.32 (t, *J* = 1.8 Hz, 1H), 7.15 (t, *J* = 7.8 Hz, 1H), 7.04 (d, *J* = 7.8 Hz, 1H), 3.25 (t, *J* = 8.4 Hz, 1H), 2.29 (s, 3H), 2.22 (dd, *J* = 7.9, 5.4 Hz, 1H), 1.88 (s, 3H), 1.63 (dd, *J* = 9.0, 5.4 Hz, 1H) ppm; **^{13}C NMR** (75 MHz, $CDCl_3$) δ 202.3, 201.6, 136.7, 131.7, 130.7, 130.0, 126.9, 122.6, 52.6, 32.7, 30.5, 27.9, 19.1 ppm; **IR** (ATR) 3059, 3010, 2923, 1683, 1594, 1564, 1478, 1421, 1357 cm^{-1}; **HRMS** (ESI): calc. for [$C_{13}H_{13}BrO_2$ + Na^+]: 304.9972, found: 304.9974.

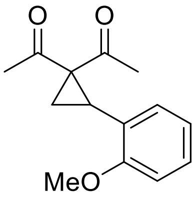

43

1,1'-(2-(2-Methoxyphenyl)cyclopropane-1,1-diyl)bis(ethan-1-one) (43):

The *title compound* was purified via column chromatography on silica gel eluting with *n*-pentane/Et_2O 3:1 (v/v) to afford 760 mg (43% two steps overall yield) of **43** as a colorless solid.

R$_f$ = 0.24 (*n*-pentane /Et_2O, 3:1 (v/v)); **mp** 60-63 °C; **^{1}H NMR** (300 MHz, $CDCl_3$) δ 7.22 (m, 1H), 6.98 (m, 1H), 6.87 (m, 2H), 3.84 (s, 3H), 3.24 (t, *J* = 8.7 Hz, 1H), 2.30 – 2.25 (m, 4H), 1.82 (s, 3H), 1.69 (dd, *J* = 9.0, 5.3 Hz, 1H) ppm; **^{13}C NMR** (75 MHz, $CDCl_3$) δ 203.9, 202.2, 158.6, 128.9, 128.6, 122.6, 120.4, 109.8, 55.3, 51.6, 30.2, 29.4, 27.1, 17.5 ppm; **IR** (ATR) 3006, 2939, 2838, 1682, 1601, 1495 cm^{-1}; **HRMS** (ESI): calc. for [$C_{14}H_{16}O_3$ + Na^+]: 255.0992, found: 255.0982.

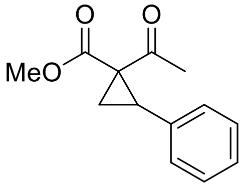

45

Methyl 1-acetyl-2-phenylcyclopropane-1-carboxylate (45)

The *title compound* was purified via column chromatography on silica gel eluting with *n*-pentane/Et_2O 5:1 (v/v) to afford 806 mg (24% two steps overall yield) of **45** as a colorless oil as a 3:1 mixture of *cis*- and *trans*-isomers.

R$_f$= 0.45 (*n*-pentane/Et_2O, 5:1 (v/v)); **^{1}H-NMR** (300 MHz, $CDCl_3$) δ 7.30-7.11 (m, 5H cis, 5H trans), 3.81 (s, 3H trans); 3.35 (s, 3H cis), 3.28 (bt, *J* = 8.7 Hz, 1H cis), 3.27 (bt, *J* = 8.7 Hz, 1H trans), 2.45 (s, 3H cis), 2.31 (dd, *J* = 5.0, 8.1 Hz, 1H trans), 2.24 (dd, *J* = 4.6, 8.1 Hz, 1H cis), 1.93 (s, 3H trans), 1.74 (dd, *J* = 4.7, 9.2 Hz, 1H cis), 1.71 (dd, *J* = 5.1 9.1 Hz, 1H trans) ppm; **^{13}C-NMR** (75 MHz, $CDCl_3$) δ 202.2 (cis), 199.9 (trans), 171.0 (trans), 168.7 (cis), 134.8 (cis), 133.7 (trans), 128.7 (cis),

128.4 (trans), 128.3 (trans), 128.1 (cis), 127.5 (trans), 127.4 (cis), 52.6 (trans), 51.9 (cis), 44.6 (cis), 44.2 (trans), 35.5 (cis), 34.5 (trans), 30.2 (trans), 29.6 (cis), 21.6 (cis), 17.8 (trans) ppm; **IR** (ATR) 3031, 3005, 2952, 1733, 1690, 1604, 1499, 1454, 1435 cm^{-1}; **HRMS** (EI): calc. for [$C_{13}H_{14}O_3$]: 218.0943, found: 218.0947.

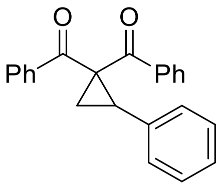

47

(2-Phenylcyclopropane-1,1-diyl)bis(phenylmethanone) (47)

The *title compound* was purified via column chromatography on silica gel eluting with *n*-pentane/Et_2O 10:1 (v/v) to afford 1.16 g (23% two steps overall yield) of **47** as a colorless solid.

$\boldsymbol{R_f}$ = 0.37 (*n*-pentane/Et_2O, 10:1 (v/v)); **mp** 130-131 °C; **^{1}H-NMR** (300 MHz, $CDCl_3$) δ 7.78-7.73 (m, 2H), 7.59-7.56 (m, 2H); 7.39-7.34 (m, 1H), 7.28-7.22 (m, 5H), 7.18- 7.05 (m, 5H), 3.99 (bt, *J* = 8.5 Hz, 1H), 2.84 (dd, *J* = 4.8, 8.2 Hz, 1H), 1.73 (dd, *J* = 5.0, 9.1 Hz, 1H) ppm; **^{13}C-NMR** (75 MHz, $CDCl_3$) δ 197.0, 194.4, 137.8, 137.7, 134.1, 132.9, 132.7, 128.6, 128.5, 128.4, 128.2, 128.1, 127.1, 49.8, 32.4, 19.7; **IR** (ATR) 3085, 3064, 3004, 1656, 1595, 1576, 1499, 1447 cm^{-1}; **HRMS** (ESI): calc. for [($C_{23}H_{18}O_2$ + Na^+]: 349.1199, found: 349.1195.

8. $NBu_4[Fe(CO)_3(NO)]$ (TBAFe)-catalyzed Cloke-Wilson Rearrangement of Vinylcyclopropanes

General procedure III: Thermal conditions for the CWR of VCPs

TBAFe (10.3 mg, 0.025 mmol) was weighed into a dried Schlenk tube. Anhydrous CH_2Cl_2 (5 mL) was added and the mixture stirred until homogenous. 1 mL (0.005 mmol TBAFe) of this solution was transferred to a separate dried Schlenk tube which was subsequently charged with the appropriate vinylcyclopropane (0.5 mmol). The Schlenk tube was sealed under an atmosphere of dry nitrogen and heated to 45 °C for 14 h. The solvent was removed under reduced pressure and the residue subjected to column chromatography on silica gel.

General procedure IV: UV-Light conditions for the CWR of VCPs

A 10-mL Schlenk tube was charged with vinylcyclopropane (0.40 mmol, 1 equiv.), TBAFe (0.001 mmol, 0.025 equiv.), and CH_3CN (1 mL) under N_2. The reactions were carried out at room temperature under irradiation of UV light (180 W, Hg lamp or 75 W, Xenon lamp at distance of 15 cm) or Visible light (23 W, Compact Fluorescent Lamp at distance of 15 cm) for 3 h. The reaction was quenched with diethyl ether and concentrated *in vacuo*. Purification by silica column chromatography afforded the desired dihydrofuran product.

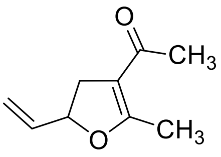

2

1-(2-Methyl-5-vinyl-4,5-dihydrofuran-3-yl)ethanone (2):[1]

Thermal condition:
The *title compound* was purified via column chromatography on silica gel eluting with *n*-pentane/Et_2O 2:1 (v/v) to afford 71.3 mg (94%) of **2** as a colorless oil.

UV-Light (180 W, Hg Lamp) condition:
The *title compound* was purified via column chromatography on silica gel eluting with *n*-pentane/Et_2O 2:1 (v/v) to afford 58.4 mg (96%) of **2** as a colorless oil.

UV-Light (75 W, Xenon Lamp) condition:
The *title compound* was purified via column chromatography on silica gel eluting with *n*-pentane/Et_2O 2:1 (v/v) to afford 56.6 mg (93%) of **2** as a colorless oil.

Visible-Light (23 W, Compact Fluorescent Lamp) condition:
The *title compound* was purified via column chromatography on silica gel eluting with *n*-pentane/Et_2O 2:1 (v/v) to afford 56.0 mg (92%) of **2** as a colorless oil.

R_f = 0.16 (*n*-pentane/Et_2O, 2:1 (v/v)); **^{1}H-NMR** (250 MHz, $CDCl_3$) δ 5.93 (ddd, *J* = 17.0, 10.3, 6.6 Hz, 1H), 5.31 (dt, *J* = 17.1, 1.1 Hz, 1H), 5.22 (dt, *J* = 10.4, 1.1 Hz, 1H), 5.09-4.99 (m, 1H), 3.18-3.08 (m, 1H), 2.78-2.69 (m, 1H), 2.24 (t, *J* = 1.4 Hz, 3H), 2.20 (s, 3H) ppm; **^{13}C-NMR** (63 MHz, $CDCl_3$) δ 194.4, 167.3, 136.7, 116.9, 111.9, 82.6, 36.3, 29.4, 15.0 ppm; **IR** (ATR, neat) 3409, 3086, 2989, 2923, 2864, 1670, 1593 cm^{-1}; **GC/MS** (EI, 70 eV) m/z (%) 152 (M^+, 100), 137 (36), 109 (72), 95 (26), 91 (40), 67 (45).

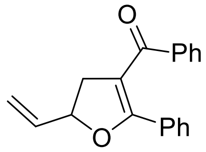

4

Phenyl(2-phenyl-5-vinyl-4,5-dihydrofuran-3-yl)methanone (4):[7]

Thermal condition:
The *title compound* was purified via column chromatography on silica gel eluting with *n*-pentane/Et_2O 10:1 (v/v) to afford 126.5 mg (92%) of **4** as a colorless solid.

UV-Light (180 W, Hg Lamp) condition:
The *title compound* was purified via column chromatography on silica gel eluting with *n*-pentane/Et_2O 10:1 (v/v) to afford 102.8 mg (93%) of **4** as a colorless solid.

R_f = 0.13 (*n*-pentane/Et_2O, 10:1 (v/v)); **^{1}H-NMR** (300 MHz, $CDCl_3$) δ 7.45-7.43 (m, 2H), 7.24-7.15 (m, 4H), 7.10-7.03 (m, 4H), 6.10 (ddd, *J* = 6.5, 10.2, 17.0 Hz, 1H), 5.46 (td, *J* = 1.1 Hz, 17.1 Hz, 1H), 5.23-5.33 (m, 2H), 3.45 (dd, *J* = 9.9, 14.7 Hz, 1H), 3.14 (dd, *J* = 8.3, 14.9 Hz, 1H) ppm; **^{13}C-NMR** (63 MHz, $CDCl_3$) δ 193.4, 165.5, 139.0, 136.6, 131.1, 130.0, 129.9, 129.4, 128.9, 127.6, 127.58, 117.2, 111.7, 82.5, 38.7 ppm; **IR** (ATR, neat) 3081, 3027, 2985, 2848, 1615, 1600, 1587, 1565, 1487 cm^{-1}; **HRMS** (ESI) calc. for [$C_{19}H_{16}O_2$+Na^+]: 299.1043, found 299.1054.

(R)-4: $[\alpha]_D^{20}$: -22.5 (c = 5.33, $CHCl_3$); **(S)-4**: $[\alpha]_D^{20}$: +22.5 (c = 5.33, $CHCl_3$).

Data File C:\HPCHEM\1\DATA\DP\DP26A000.D Sample Name: DP26A

Chiralcel OD-H; Heptan/Isopropanol 95:5, 0.5 mL/min; 3µ
l

===

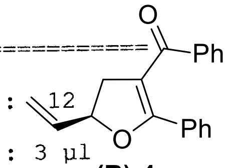

Injection Date : 26.05.15 15:59:57
Sample Name : DP26A
Acq. Operator : Dominik
Vial : 12
Inj Volume : 3 µl

(R)-4
Product of thermal condition

Method : C:\HPCHEM\1\METHODS\BPOD9_1.M
Last changed : 22.05.15 15:55:29 by Dominik

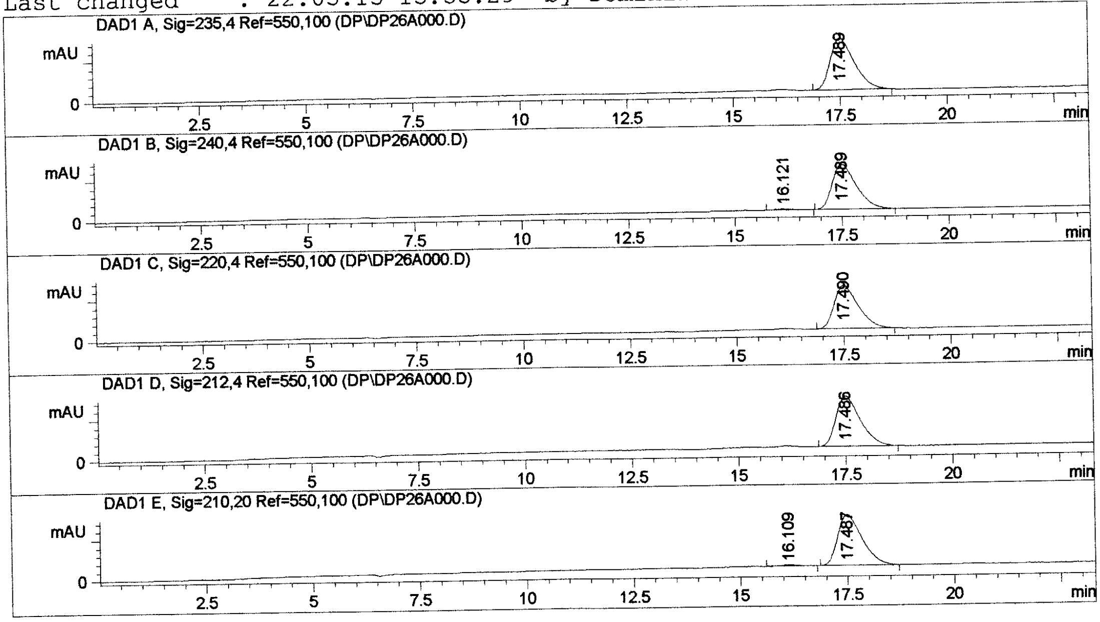

===
Area Percent Report
===

Sorted By : Signal
Multiplier : 1.0000
Dilution : 1.0000

Signal 1: DAD1 A, Sig=235,4 Ref=550,100

Peak #	RetTime [min]	Type	Width [min]	Area [mAU*s]	Height [mAU]	Area %
1	17.489	BB	0.5846	2485.33032	62.94952	100.0000

Totals : 2485.33032 62.94952

Results obtained with enhanced integrator!

Data File C:\HPCHEM\1\DATA\DP\DP26B000.D Sample Name: DP26B

Chiralcel OD-H; Heptan/Isopropanol 95:5, 0.5 mL/min; 3µ
l

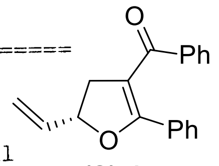

(S)-4
Product of thermal condition

===

Injection Date	:	26.05.15 16:25:38
Sample Name	:	DP26B
Acq. Operator	:	Dominik
Method	:	C:\HPCHEM\1\METHODS\BPOD9_1.M
Last changed	:	22.05.15 15:55:29 by Dominik

Vial : 12
Inj Volume : 3 µl

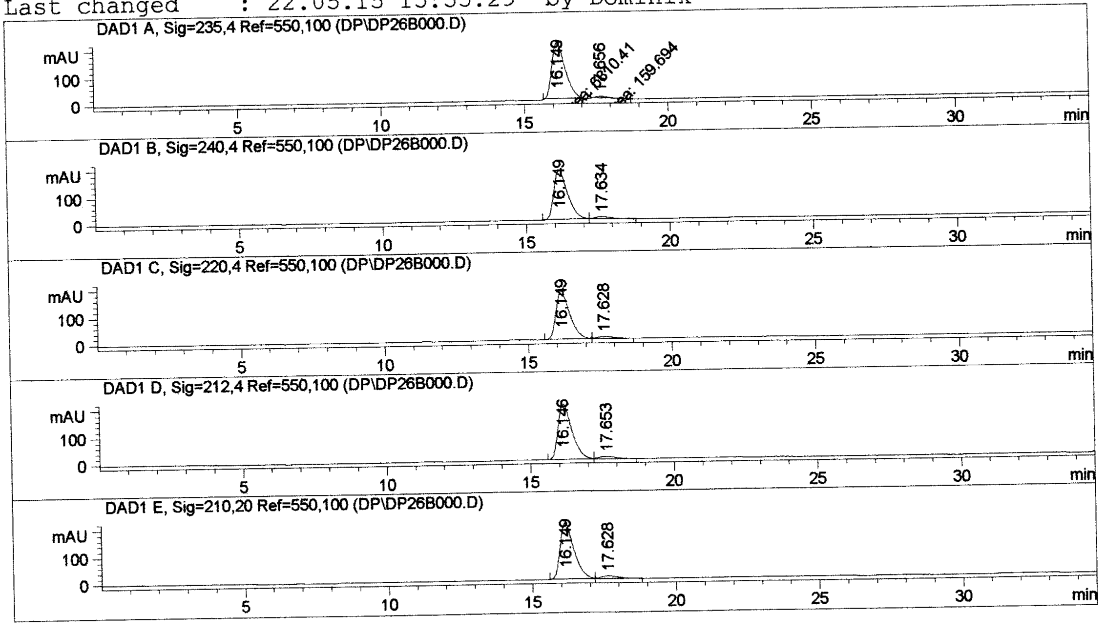

===

Area Percent Report

===

Sorted By	:	Signal
Multiplier	:	1.0000
Dilution	:	1.0000

Signal 1: DAD1 A, Sig=235,4 Ref=550,100

Peak #	RetTime [min]	Type	Width [min]	Area [mAU*s]	Height [mAU]	Area %
1	16.149	MM	0.5504	6810.40723	206.22845	97.7089
2	17.656	MM	0.4684	159.69370	5.68170	2.2911

Totals : 6970.10092 211.91015

Results obtained with enhanced integrator!

Data File C:\HPCHEM\1\DATA\DP\CL-24A00.D Sample Name: CL-24A

Chiralcel OD-H; Heptan/Isopropanol 95:5, 0.5 mL/min; 3µ
l

===

Injection Date : 22.05.15 14:34:47
Sample Name : CL-24A Vial : 11
Acq. Operator : Dominik
Inj Volume : 3 µl

Acq. Method : C:\HPCHEM\1\METHODS\BPOD9_1.M
Last changed : 22.05.15 14:33:48 by Dominik
(modified after loading)
Analysis Method : C:\HPCHEM\1\METHODS\BPOD9_1.M
Last changed : 22.05.15 15:55:29 by Dominik

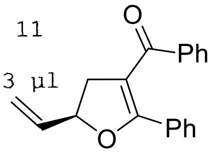

(R)-4

Product of photochemical condition

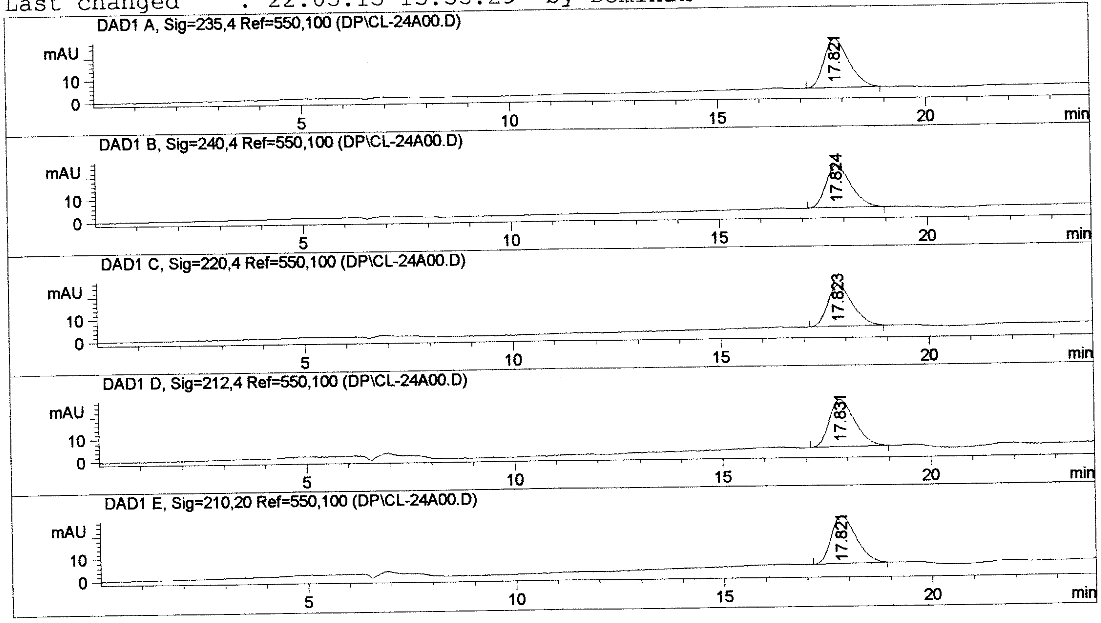

===

Area Percent Report

===

Sorted By : Signal
Multiplier : 1.0000
Dilution : 1.0000

Signal 1: DAD1 A, Sig=235,4 Ref=550,100

Peak #	RetTime [min]	Type	Width [min]	Area [mAU*s]	Height [mAU]	Area %
1	17.821	BB	0.5863	908.40143	21.97522	100.0000

Totals : 908.40143 21.97522

Results obtained with enhanced integrator!

Data File C:\HPCHEM\1\DATA\DP\CL-24B00.D   Sample Name: CL-24B

Chiralcel OD-H; Heptan/Isopropanol 95:5, 0.5 mL/min; 3µ
l

===

Injection Date : 22.05.15 14:59:51
Sample Name : CL-24B
Acq. Operator : Dominik
Vial : 10
Inj Volume : 3 µl

Acq. Method : C:\HPCHEM\1\METHODS\BPOD9_1.M
Last changed : 22.05.15 14:33:48 by Dominik
(modified after loading)
Analysis Method : C:\HPCHEM\1\METHODS\BPOD9_1.M
Last changed : 22.05.15 15:55:29 by Dominik

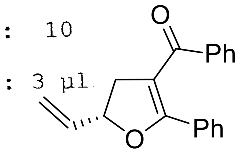

(S)-4

Product of photochemical condition

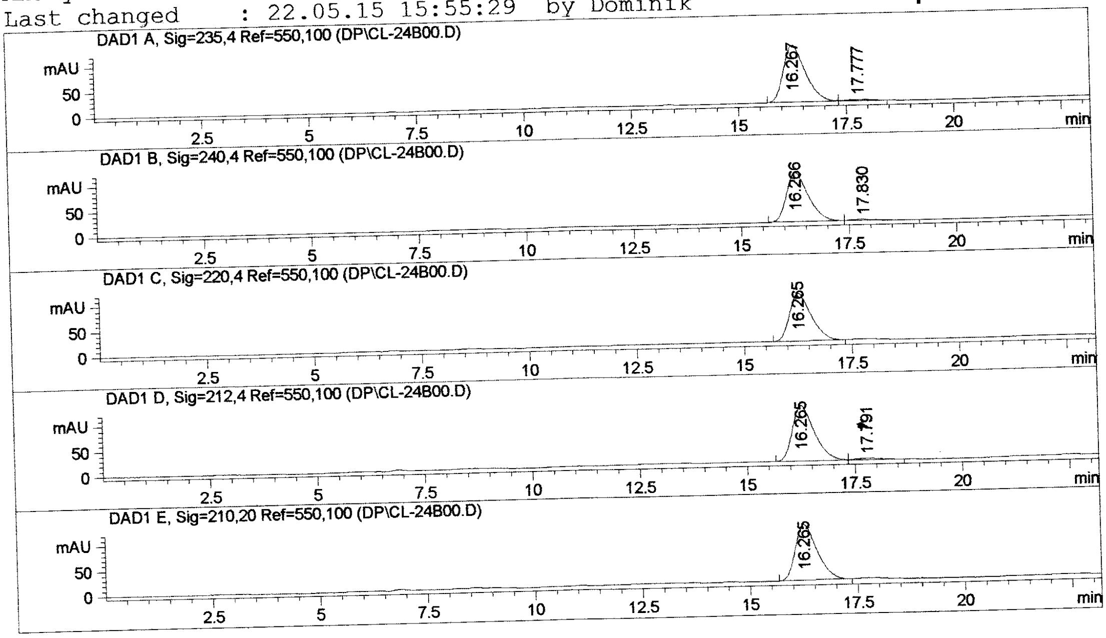

===

Area Percent Report

===

Sorted By : Signal
Multiplier : 1.0000
Dilution : 1.0000

Signal 1: DAD1 A, Sig=235,4 Ref=550,100

Peak #	RetTime [min]	Type	Width [min]	Area [mAU*s]	Height [mAU]	Area %
1	16.267	BB	0.5276	4053.75537	113.80764	97.3832
2	17.777	BB	0.4835	108.92802	2.71816	2.6168

Totals : 4162.68339 116.52580

Results obtained with enhanced integrator!

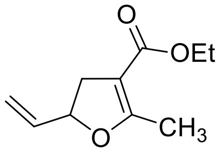

6

Ethyl 2-methyl-5-vinyl-4,5-dihydrofuran-3-carboxylate (6):[1]

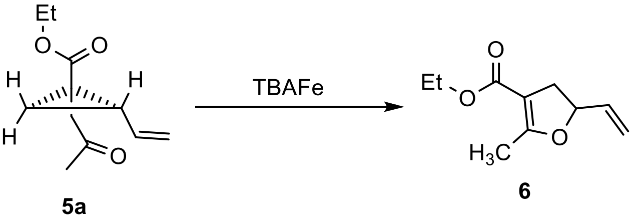

Thermal condition:
Starting from diastereoisomer **5a**: The *title compound* was purified via column chromatography on silica gel eluting with *n*-pentane/Et_2O 15:1 (v/v) to afford 84.6 mg (93%) of **6** as a colorless oil.

UV-Light (180 W, Hg Lamp) condition:
Starting from diastereoisomer **5a**: The *title compound* was purified via column chromatography on silica gel eluting with *n*-pentane/Et_2O 15:1 (v/v) to afford 68.5 mg (94%) of **6** as a colorless oil.

Visible-Light (23 W, Compact Fluorescent Lamp) condition:
Starting from diastereoisomer **5a**: The *title compound* was purified via column chromatography on silica gel eluting with *n*-pentane/Et_2O 15:1 (v/v) to afford 67.8 mg (93%) of **6** as a colorless oil.

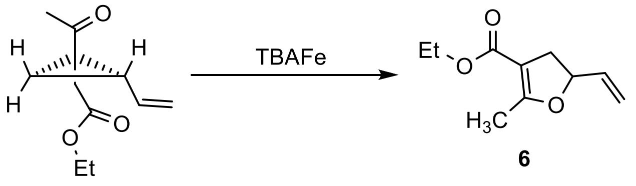

Thermal condition:
Starting from diastereoisomer **5b**: The *title compound* was purified via column chromatography on silica gel eluting with *n*-pentane/Et_2O 15:1 (v/v) to afford 83.9 mg (92%) of **6** as a colorless oil.

UV-Light (180 W, Hg Lamp) condition:
Starting from diastereoisomer **5b**: The *title compound* was purified via column chromatography on silica gel eluting with *n*-pentane/Et_2O 15:1 (v/v) to afford 67.1 mg (92%) of **6** as a colorless oil.

Visible-Light (23 W, Compact Fluorescent Lamp) condition:
Starting from diastereoisomer **5b**: The *title compound* was purified via column chromatography on silica gel eluting with *n*-pentane/Et_2O 15:1 (v/v) to afford 67.1 mg (92%) of **6** as a colorless oil.

R_f = 0.26 (*n*-pentane/Et_2O, 15:1 (v/v)); **^{1}H-NMR** (250 MHz, $CDCl_3$) δ 5.93 (ddd, *J* = 17.1, 10.3, 6.6 Hz, 1H), 5.29 (dt, *J* = 17.1, 1.2 Hz, 1H), 5.20 (dt, *J* = 10.3, 1.1 Hz, 1H), 5.08-4.98 (m, 1H), 4.17 (q, *J* = 7 Hz, 2H), 3.12-3.01 (m, 1H), 2.72-2.62 (m, 1H), 2.21 (t, *J* = 1.5 Hz, 3H), 1.28 (t, *J* = 7.1 Hz, 3H) ppm; **^{13}C-NMR** (63 MHz, $CDCl_3$) δ 167.5, 166.1, 136.9, 116.6, 101.7, 82.5, 59.5, 35.6, 14.4, 14.1 ppm; **IR** (ATR, neat) 2981, 2928, 2870, 1695, 1642 cm^{-1}; **GC/MS** (EI, 70 eV) m/z (%) 182 (M^+, 54), 139 (53), 137 (55), 135 (46), 121 (100), 94 (64), 93 (54), 67 (50), 66 (83), 65 (30), 55 (25).

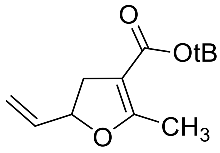

8

***tert*-Butyl 2-methyl-5-vinyl-4,5-dihydrofuran-3-carboxylate (8):**[1]

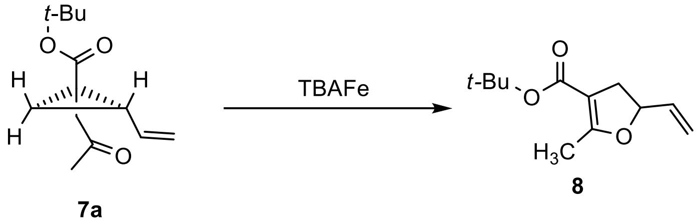

7a

Thermal condition:

Starting from diastereoisomer **7a**: The *title compound* was purified via column chromatography on silica gel eluting with *n*-pentane/Et_2O 15:1 (v/v) to afford 98.7 mg (94%) of **8** as a colorless oil.

UV-Light (180 W, Hg Lamp) condition:

Starting from diastereoisomer **7a**: The *title compound* was purified via column chromatography on silica gel eluting with *n*-pentane/Et_2O 15:1 (v/v) to afford 71.5 mg (85%) of **8** as a colorless oil.

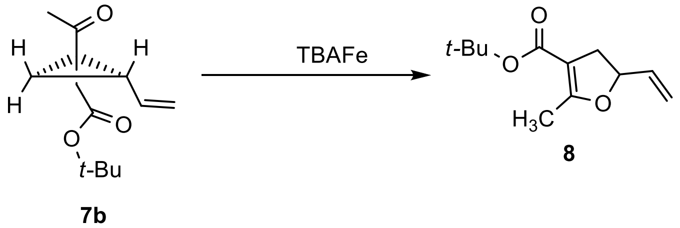

7b

Thermal condition:

Starting from diastereoisomer **7b**: The *title compound* was purified via column chromatography on silica gel eluting with *n*-pentane/Et_2O 15:1 (v/v) to afford 62.9 mg (60%) of **8** as a colorless oil.

UV-Light (180 W, Hg Lamp) condition:

Starting from diastereoisomer **7b**: The *title compound* was purified via column chromatography on silica gel eluting with *n*-pentane/Et_2O 15:1 (v/v) to afford 76.5 mg (91%) of **8** as a colorless oil.

R_f = 0.37 (*n*-pentane/Et_2O, 15:1 (v/v)); **^{1}H-NMR** (250 MHz, $CDCl_3$) δ 5.93 (ddd, *J* = 17.0, 10.3, 6.7 Hz 1H), 5.29 (dt, *J* = 17.2, 1.2 Hz, 1H), 5.19 (dt, *J* = 10.4, 1.1 Hz, 1H), 5.04-4.94 (m, 1H), 3.07-2.97

(m, 1H), 2.68-2.58 (m, 1H), 2.17 (t, *J* = 1.6 Hz, 3H), 1.48 (s, 9H) ppm; **^{13}C-NMR** (63 MHz, $CDCl_3$) δ 166.2, 165.6, 137.1, 116.5, 103.1, 82.2, 79.5, 35.9, 28.4, 14.1 ppm; **IR** (ATR, neat) 2975, 2928, 2869, 1687, 1645 cm^{-1}; **GC/MS** (EI, 70 eV) m/z (%) 210 (M$^+$, 32), 154 (63), 137 (63), 135 (55), 121 (91), 111 (88), 94 (100), 93 (47), 66 (56), 57 (46).

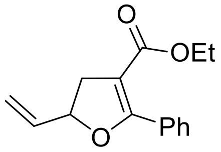

10

Ethyl 2-phenyl-5-vinyl-4,5-dihydrofuran-3-carboxylate (10):[8]

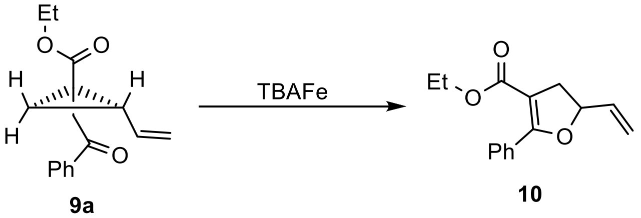

Thermal condition:
Starting from diastereoisomer **9a**: 5 mol% TBAFe were used The *title compound* was purified via column chromatography on silica gel eluting with *n*-pentane/Et_2O 10:1 (v/v) to afford 106.4 (87%) of **10** as a colorless oil.

UV-Light (180 W, Hg Lamp) condition:
Starting from diastereoisomer **9a**: The *title compound* was purified via column chromatography on silica gel eluting with *n*-pentane/Et_2O 10:1 (v/v) to afford 88.9 (91%) of **10** as a colorless oil.

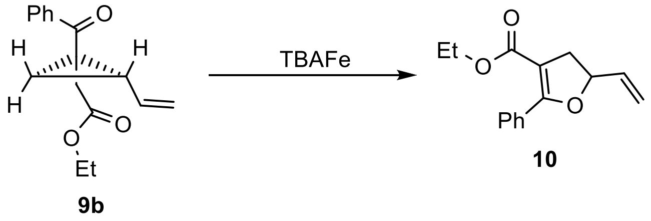

Thermal condition:
Starting from diastereoisomer **9b**: The *title compound* was purified via column chromatography on silica gel eluting with *n*-pentane/Et_2O 10:1 (v/v) to afford 104.9 mg (86%) of **10** as a colorless oil.

UV-Light (180 W, Hg Lamp) condition:
Starting from diastereoisomer **9b**: The *title compound* was purified via column chromatography on silica gel eluting with *n*-pentane/Et_2O 10:1 (v/v) to afford 89.9 mg (92%) of **10** as a colorless oil.

R_f = 0.27 (*n*-pentane/Et_2O, 10:1 (v/v)); **^{1}H-NMR** (250 MHz, $CDCl_3$) δ 7.81-7.77 (m, 2H), 7.42-7.33 (m, 3H), 6.03 (ddd, *J* = 6.5, 10.3, 17.1 Hz, 1H), 5.41-5.12 (m, 3H), 4.13 (q, *J* = 7.2 Hz, 2H), 3.30 (dd, *J* = 10.6, 15.1 Hz, 1H), 2.92 (dd, *J* = 8.3, 15.1 Hz, 1H), 1.20 (t, *J* = 7.2, 3H) ppm; **^{13}C NMR** (63 MHz, $CDCl_3$) δ 165.2, 164.6, 136.8, 130.3, 129.9, 129.3, 127.6, 116.8, 102.1, 82.0, 59.7, 37.3, 14.2 ppm; **IR**

(ATR) 2980, 2867, 1703, 1680, 1623, 1597, 1574, 1493, 1446 cm^{-1}; **HRMS (ESI)** calc. for [$C_{15}H_{16}O_3$+Na^+] 267.0992, found 267.0994.

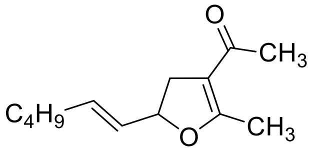

12

(*E*)-1-(5-(hex-1-en-1-yl)-2-methyl-4,5-dihydrofuran-3-yl)ethan-1-one (12):

Thermal condition:
This reaction was performed using 5 mol-% TBAFe. The *title compound* was purified via column chromatography on silica gel eluting with *n*-pentane/Et_2O 4:1 (v/v) to afford 90 mg (87%) of **12** as a brown oil.

UV-Light (180 W, Hg Lamp) condition:
This reaction was performed by using 5 mol-% TBAFe for 6 hours. The *title compound* was purified via column chromatography on silica gel eluting with *n*-pentane/Et_2O 4:1 (v/v) to afford 72.5 mg (87%) of **12** as a yellow oil.

R_f = 0.22 (*n*-pentane/Et_2O, 4:1 (v/v)); **^{1}H NMR** (300 MHz, $CDCl_3$) δ 5.76 (m, 1H), 5.61 – 5.51 (m, 1H), 5.00 (dd, *J* = 18.0, 8.0 Hz, 1H), 3.09 (m, 1H), 2.71 (m, 1H), 2.22 (t, *J* = 1.3 Hz, 3H), 2.19 (s, 3H), 2.07 (dd, *J* = 13.6, 6.8 Hz, 2H), 1.44 – 1.27 (m, 4H), 0.90 (t, *J* = 7.1 Hz, 3H) ppm; **^{13}C NMR** (75 MHz, $CDCl_3$) δ 194.6, 167.4, 135.1, 128.5, 112.1, 83.2, 36.7, 31.8, 30.9, 29.4, 22.2, 15.0, 13.9 ppm; **IR** (ATR) 2957, 2927, 2857, 1671, 1591, 1381, 1216, 1132, 1061, 967, 926, 625 cm^{-1}; **GC/MS** (ESI) m/z (%)209 (M + H^+, 25), 167 (16), 149 (18), 123 (25), 113 (100); **HRMS** (ESI) calc. for [$C_{13}H_{20}O_2$ + Na^+] 231.1356, found: 231.1358.

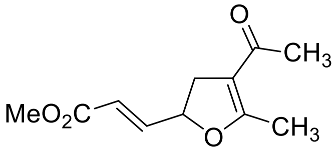

14

Methyl (*E*)-3-(4-acetyl-5-methyl-2,3-dihydrofuran-2-yl)acrylate (14):

Thermal condition:
This reaction was performed using 5 mol-% TBAFe. The *title compound* was purified via column chromatography on silica gel eluting with *n*-pentane/Et_2O 4:1 (v/v) to afford 101 mg (96%) of **14** as a red oil.

UV-Light (180 W, Hg Lamp) condition:

This reaction was performed by using 5 mol-% TBAFe for 6 hours. The *title compound* was purified via column chromatography on silica gel eluting with *n*-pentane/Et_2O 4:1 (v/v) to afford 74.8 mg (89%) of **14** as a yellow oil.

R R_f = 0.19 (*n*-pentane /Et_2O, 2:1 (v/v)); **^{1}H NMR** (300 MHz, $CDCl_3$) δ 6.93 (dd, *J* = 15.6, 4.8 Hz, 1H), 6.02 (d, *J* = 15.6 Hz, 1H), 5.28 – 5.16 (m, 1H), 3.76 (s, 3H), 3.30 – 3.15 (m, 1H), 2.77 (dd, *J* = 14.0, 7.1 Hz, 1H), 2.26 (s, 3H), 2.21 (s, 3H) ppm; **^{13}C NMR** (75 MHz, $CDCl_3$) δ 194.0, 166.8, 166.2, 145.2, 120.8, 111.6, 79.5, 51.7, 36.0, 29.4, 14.7 ppm; **IR** (ATR) 2953, 2923, 1722, 1673, 1597,1435, 1389, 1362, 1305, 1272, 1219, 1172, 987, 928, 732, 625 cm^{-1}; **GC/MS** (ESI) m/z (%) 211 (M + H^+, 17), 137 (100), 119 (12); **HRMS** (ESI) calc. for [$C_{11}H_{14}O_4$ + H^+]: 211.0965, found: 211.0961.

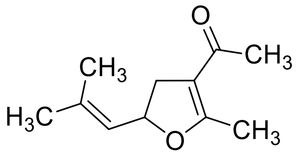

16

1-(2-Methyl-5-(2-methylprop-1-en-1-yl)-4,5-dihydrofuran-3-yl)ethan-1-one (16):

Thermal condition:
The reaction was performed on a 0.275 mmol scale using 5 mol-% TBAFe. The *title compound* was purified via column chromatography on silica gel eluting with *n*-pentane/Et_2O 4:1 (v/v) to afford 27 mg (54%) of **16** as a purple oil.

UV-Light (180 W, Hg Lamp) condition:
This reaction was performed by using 5 mol-% TBAFe for 6 hours. The *title compound* was purified via column chromatography on silica gel eluting with *n*-pentane/Et_2O 4:1 (v/v) to afford 54.1 mg (75%) of **16** as a colorless oil.

Visible-Light (23 W, Compact Fluorescent Lamp) condition:
This reaction was performed by using 5 mol-% TBAFe for 6 hours. The *title compound* was purified via column chromatography on silica gel eluting with *n*-pentane/Et_2O 4:1 (v/v) to afford 54.8 mg (76%) of **16** as a colorless oil.

R_f = 0.25 (*n*-pentane/Et_2O, 4:1(v/v)); **^{1}H NMR** (300 MHz, $CDCl_3$) δ 5.39 – 5.23 (m, 2H), 3.17 – 3.02 (m, 1H), 2.73 – 2.58 (m, 1H), 2.21 (t, *J* = 1.4 Hz, 3H), 2.19 (s, 3H), 1.78 (s, 3H), 1.74 (s, 3H) ppm; **^{13}C NMR** (75 MHz, $CDCl_3$) δ 194.7, 167.9, 138.5, 124.2, 112.1, 79.3, 37.1, 29.4, 25.8, 18.3, 15.2 ppm; **IR** (ATR) ν 2972, 2916, 2859, 1714, 1669, 1589, 1451, 1380, 1216, 1132, 928, 865, 625 cm^{-1}; **GC/MS** (ESI) m/z (%) 181 (M^+, 21), 163 (18), 145 (25),123 (100), 113 (24),105 (25); **HRMS** (ESI) calc. for [$C_{11}H_{16}O_2$ + Na^+]: 203.1043, found: 203.1039.

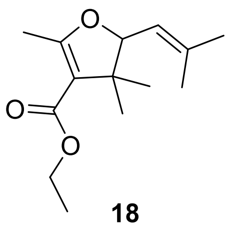

18

Ethyl 2,4,4-trimethyl-5-(2-methylprop-1-en-1-yl)-4,5-dihydrofuran-3-carboxylate (18):

UV-Light (180 W, Hg Lamp) condition:
This reaction was performed by using 10 mol-% TBAFe in THF (1 ml) for 24 hours. The *title compound* was purified via column chromatography on silica gel eluting with PE/EA 20:1 (v/v) to afford 93.3 mg (98%) of **18** as a colorless oil.

UV-Light (75 W, Xenon Lamp) condition:
This reaction was performed by using 10 mol-% TBAFe in THF (1 ml) for 24 hours. The *title compound* was purified via column chromatography on silica gel eluting with PE/EA 20:1 (v/v) to afford 91.5 mg (96%) of **18** as a colorless oil.

Visible-Light (23 W, Compact Fluorescent Lamp) condition:
This reaction was performed by using 10 mol-% TBAFe in THF (1 ml) for 24 hours. The *title compound* was purified via column chromatography on silica gel eluting with PE/EA 20:1 (v/v) to afford 18.1 mg (19%) of **18** as a colorless oil.

***R**$_f$* = 0.35 (PE/EA 20:1(v/v)); **^{1}H-NMR** (300 MHz, $CDCl_3$) δ 5.32 (qd, *J* = 1.3, 9.8 Hz, 1H), 4.78 (d, *J* = 9.8 Hz, 1H), 4.18 (dq, *J* = 1.7, 7.1 Hz, 2H), 2.17 (s, 3H), 1.82 (d, *J* = 1.2 Hz, 3H), 1.74 (d, *J* = 1.3 Hz, 3H), 1.30 (t, *J* = 7.2 Hz, 3H), 1.23 (s, 3H), 1.05 (s, 3H) ppm; **^{13}C-NMR** (75 MHz, $CDCl_3$) δ 167.7, 166.1, 139.7, 119.2, 119.9, 88.5, 59.0, 46.2, 26.2, 25.6, 21.8, 18.4, 14.9, 14.3 ppm; **IR** (ATR) 1693, 1628, 1334, 1308, 1253, 1078, 1061, 976, 951, 776 cm^{-1}; **HRMS** (ESI) calc. for [$C_{14}H_{22}O_3$ + Na^+]: 261.1461, found: 261.1460.

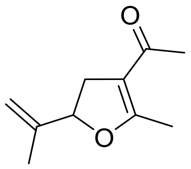

20

1-(2-Methyl-5-(prop-1-en-2-yl)-4,5-dihydrofuran-3-yl)ethan-1-one (20)

Thermal condition:

This reaction was performed using 5 mol-% TBAFe. The *title compound* was purified via column chromatography on silica gel eluting with *n*-pentane/Et_2O 2:1 (v/v) to afford 80 mg (96%) of **20** as a yellow oil.

UV-Light (180 W, Hg Lamp) condition:
The *title compound* was purified via column chromatography on silica gel eluting with *n*-pentane/Et_2O 2:1 (v/v) to afford 64.5 mg (97%) of **20** as a yellow oil.

R_f = 0.46 (*n*-pentane/Et_2O 2:1 (v/v)); **^{1}H NMR** (300 MHz, $CDCl_3$) δ 5.05 – 4.97 (m, 2H), 4.91 – 4.88 (m, 1H), 3.09 (ddq, *J* = 13.5, 10.6, 1.4 Hz, 1H), 2.76 (ddq, *J* = 14.1, 8.4, 1.5 Hz, 1H), 2.25 (t, *J* = 1.5 Hz, 3H), 2.21 (s, 3H), 1.74 (s, 3H) ppm; **^{13}C NMR** (75 MHz, $CDCl_3$) δ 194.4, 167.6, 143.3, 112.1, 112.0, 84.8, 35.3, 29.4, 17.0, 14.9 ppm; **IR** (Film) 2918, 2865, 1672, 1598, 1389, 1219 cm^{-1}; **GC/MS** (EI, 70 eV) m/z (%) = 166 (30) [M^+], 123 (37), 43 (100) **HRMS** (EI) calc. for [$C_{10}H_{14}O_2$]: 166.0994, found: 166.0995.

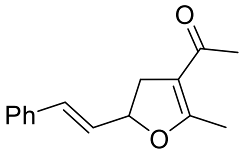

22

(*E*)-1-(2-Methyl-5-styryl-4,5-dihydrofuran-3-yl)ethan-1-one (22)

Thermal condition:
This reaction was performed on a 0.25 mmol scale using 5 mol-% TBAFe. The *title compound* was purified via column chromatography on silica gel eluting with *n*-pentane/Et_2O 2:1 (v/v) to afford 56 mg (98%) of **22** as a brown oil.

UV-Light (180 W, Hg Lamp) condition:
This reaction was performed by using 5 mol-% TBAFe for 6 hours. The *title compound* was purified via column chromatography on silica gel eluting with *n*-pentane/Et_2O 2:1 (v/v) to afford 89.5 mg (98%) of **22** as a brown oil.

R_f = 0.25 (*n*-pentane/Et_2O, 4:1(v/v)); **^{1}H NMR** (300 MHz, $CDCl_3$) δ 7.43 – 7.23 (m, 5H), 6.62 (d, *J* = 15.8 Hz, 1H), 6.26 (dd, *J* = 15.8, 7.3 Hz, 1H), 5.26 – 5.15 (m, 1H), 3.19 (m, 1H), 2.83 (m, 1H), 2.26 (t, *J* = 1.4 Hz, 3H), 2.21 (s, 3H) ppm; **^{13}C NMR** (75 MHz, $CDCl_3$) δ 194.46, 167.39, 135.97, 132.57, 128.66, 128.22, 127.63, 126.71, 112.09, 82.74, 36.76, 29.48, 15.08 ppm; **IR** (Film) 2922, 2863, 1669, 1592, 1494, 1449, 1423, 1219 cm^{-1}; **HRMS** (ESI) calc. for [$C_{15}H_{16}O_2$ + Na^+] 251.1043, found: 251.1040.

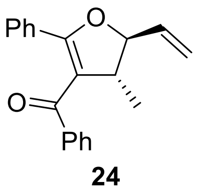

24

(4-Methyl-2-phenyl-5-vinyl-4,5-dihydrofuran-3-yl)(phenyl)methanone (24):[1]

Thermal condition:
The *title compound* was purified via column chromatography on silica gel eluting with PE/EA 20:1 (v/v) to afford 52 mg (72%) of **24** as a colorless oil.

UV-Light (180 W, Hg Lamp) condition:

The *title compound* was purified via column chromatography on silica gel eluting with PE/EA 20:1 (v/v) to afford 106.7 mg (92%) of **24** as a colorless oil.

***R**_f* = 0.33 (PE/EA, 20:1 (v/v)); **^{1}H-NMR** (300 MHz, $CDCl_3$) δ 7.56-7.47 (m, 2H), 7.27-7.19 (m, 3H), 7.19-7.12 (m, 1H), 7.12-7.00 (m, 4H), 6.07 (ddd, *J* = 6.8, 10.4, 17.1 Hz, 1H), 5.44 (td, *J* = 1.2, 17.2 Hz, 1H), 5.30 (td, *J* = 1.0, 10.4 Hz, 1H), 4.77- 4.69 (m, 1H), 3.58-3.46 (m, 1H), 1.36 (d, *J* = 6.6 Hz, 3H) ppm; **^{13}C-NMR** (75 MHz, $CDCl_3$) δ 193.8, 164.3, 139.2, 135.9, 131.4, 130.03, 129.97, 129.4, 129.0, 127.7, 127.6, 117.5, 116.8, 90.2, 46.5, 17.9 ppm; **IR** (ATR) 3061, 2958, 1721, 1613, 1593, 1572, 1490, 1447, 1158, 889, 729, 692 cm^{-1}; **HRMS** (ESI) calc. for [$C_{20}H_{18}O_2$ + Na^+]: 313.1199, found: 313.1193.

9. NBu$_4$[Fe(CO)$_3$(NO)] (TBAFe)-catalyzed Cloke-Wilson Rearrangement of Arylcyclopropanes

Table 1. Optimization of Cloke-Wilson Rearrangement of Arylcyclopropanes under Thermal/Microwave Condition.

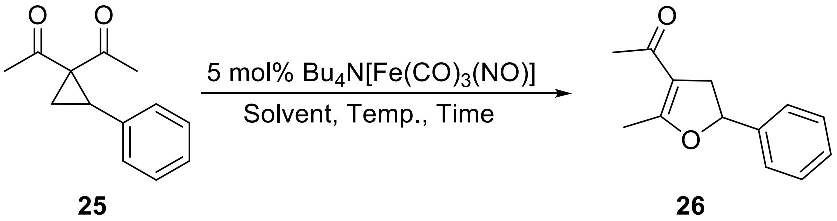

Entry[a]	Solvent	Temp./heating method	Time	Conversion[b]
1	CH_2Cl_2	45 °C (oil bath)	14 h	< 5%
2	THF	120 °C (oil bath)	14.5 h	< 5%
3	THF	120 °C (MW)	1 h	< 5%
4	PhMe	120 °C (MW)	1 h	< 5%
5	CH_3CN	120 °C (MW)	1 h	8%
6	*n*-Pentane	120 °C (MW)	1 h	11%
7	DMF	120 °C (MW)	1 h	66%
8	DMF	120 °C (MW)	1 h	< 5%
10	DMF	120 °C (oil bath)	1 h	16%
11	DMF	120 °C (MW)	2 h	79%

[a] Reaction were performed on a 0.25 mmol scale in absolute solvents. [b] Determined via GC-intergration.

Table 2. Optimization of Cloke-Wilson Rearrangement of Arylcyclopropanes under UV-Light Condition.

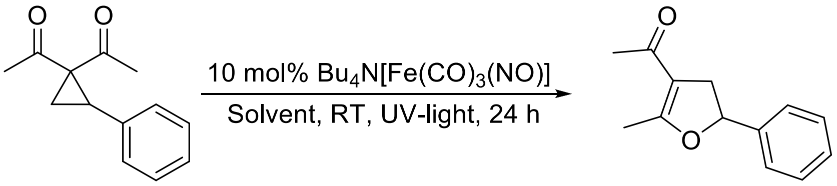

25 **26**

Entry[a]	Solvent	Yield[b]
1	DMF	82
2	MeOH	-
3	CH_3CN	-
4	CH_2Cl_2	-
5	THF	-
6	*n*-Pentane	-

[a] 0.4 mmol substrate, 10 mol% TBA[Fe], 1 mL solvent, 180 W Hg-lamp, 20 °C, 24 h. [b] Isolated yield.

General procedure V: Microwave conditions for the CWR of ACPs

TBAFe (5.2 mg, 0.0125 mmol, 0.05 equiv.) and the corresponding ACP (0.25 mmol, 1 equiv.) were weighed into a dried 10 mL microwave tube. Anhydrous DMF (1 mL) was added and the tube was sealed under an atmosphere of dry nitrogen. The reaction mixture was stirred for 2 h at 120 °C under microwave conditions. The product was obtained via chromatography on silica gel.

General procedure VI: UV-Light conditions for the CWR of ACPs

A 10-mL Schlenk tube was charged with Arylcyclopropane (0.40 mmol, 1 equiv.), TBAFe (0.04 mmol, 0.1 equiv.), and DMF (1 mL) under N_2. The reactions were carried out at room temperature under irradiation of UV light (180 W, Hg lamp or 75 W, Xe lamp at distance of 15 cm) or Visible light (23 W, Compact Fluorescent Lamp at distance of 15 cm) for 24 h. The reaction was quenched with diethyl ether and concentrated *in vacuo*. Purification by silica column chromatography afforded the desired dihydrofuran product.

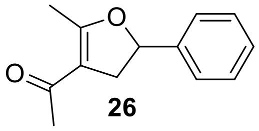

26

1-(2-Methyl-5-phenyl-4,5-dihydrofuran-3-yl)ethan-1-one (26)[9]

Thermal condition:
The *title compound* was purified by column chromatography on silica gel eluting with *n*-pentane/Et_2O 2:1 (v/v) to afford 50 mg (99%) of **26** as a colorless oil.

UV-Light (180 W, Hg Lamp) condition:

The *title compound* was purified by column chromatography on silica gel eluting with *n*-pentane/Et_2O 2:1 (v/v) to afford 66.3 mg (82%) of **26** as a colorless oil.

UV-Light (75 W, Xenon Lamp) condition:
The *title compound* was purified by column chromatography on silica gel eluting with *n*-pentane/Et_2O 2:1 (v/v) to afford 46.1 mg (57%) of **26** as a colorless oil.

Visible-Light (23 W, Compact Fluorescent Lamp) condition:
The *title compound* was purified by column chromatography on silica gel eluting with *n*-pentane/Et_2O 2:1 (v/v) to afford 4.0 mg (5%) of **26** as a colorless oil.

R_f = 0.46 (*n*-Pentane/Et_2O, 2:1 (v/v)); **^{1}H NMR** (300 MHz, CDCl3) δ 7.41-7.29 (m, 5H), 5.60 (dd, *J* = 8.4, 10.6 Hz, 1H), 3.40 (qdd, *J* = 1.5, 10.7, 14.3 Hz, 1H), 2.97 (qdd, *J* = 1.5, 8.4, 14.2 Hz, 1H), 2.31 (t, *J* = 1.5 Hz, 3H), 2.22 (s, 3H) ppm; **^{13}C NMR** (75 MHz, CDCl3) δ 194.4, 167.4, 141.2, 128.7, 128.2, 125.6, 111.8, 83.1, 38.7, 29.4, 15.0 ppm; **IR** (ATR) 3063, 3032, 3002, 2954, 2919, 2865, 1670, 1595, 1495, 1452, 1423 cm^{-1}; **HRMS** (ESI): calc. for [$C_{13}H_{14}O_2 + Na^+$]: 225.0886, found: 225.0892.

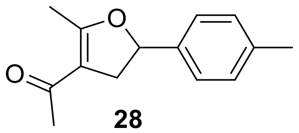

28

1-(2-Methyl-5-(p-tolyl)-4,5-dihydrofuran-3-yl)ethan-1-one (28)[9]

Thermal condition:
The *title compound* was purified by column chromatography on silica gel eluting with *n*-pentane/Et_2O 3:1 (v/v) to afford 54 mg (99%) of **28** as a yellow oil.

UV-Light (180 W, Hg Lamp) condition:
The *title compound* was purified by column chromatography on silica gel eluting with *n*-pentane/Et_2O 3:1 (v/v) to afford 73.5 mg (85%) of **28** as a colorless oil.

R_f = 0.21 (*n*-pentane/Et_2O, 3:1 (v/v)); **^{1}H NMR** (300 MHz, $CDCl_3$) δ 7.25 – 7.15 (m, 4H), 5.56 (dd, *J* = 10.6, 8.4 Hz, 1H), 3.36 (m, 1H), 2.96 (m, 1H), 2.35 (s, 3H), 2.30 (t, *J* = 1.5 Hz, 3H), 2.21 (s, 3H) ppm; **^{13}C NMR** (75 MHz, $CDCl_3$) δ 194.5, 167.5, 138.3, 138.1, 129.4, 125.7, 111.9, 83.3, 38.7, 29.5, 21.2, 15.0 ppm; **IR** (Film) 2921 (w), 2864 (w), 1670 (m), 1592 (s), 1516 (m), 1423 (m), 1381 (m),1359 (m) cm^{-1}; **GC/MS** (ESI) m/z (%) = 239 (100) [M^+ + Na]; **HRMS** (ESI) calc. for [$C_{14}H_{16}O_2 + Na^+$]: 239.1043, found: 239.1033.

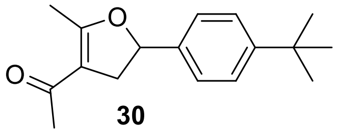

30

1-(5-(4-(*tert*-Butyl)phenyl)-2-methyl-4,5-dihydrofuran-3-yl)ethan-1-one (30)[5]

Thermal condition:

The *title compound* was purified by column chromatography on silica gel eluting with *n*-pentane/Et_2O 3:1 (v/v) to afford 48 mg (75%) of **30** as a yellow oil.

UV-Light (180 W, Hg Lamp) condition:
The *title compound* was purified by column chromatography on silica gel eluting with *n*-pentane/Et_2O 3:1 (v/v) to afford 79.6 mg (77%) of **30** as a yellow oil.

R_f = 0.31 (*n*-pentane/Et_2O, 3:1 (v/v)); **^{1}H NMR** (300 MHz, $CDCl_3$) δ 7.43 – 7.38 (m, 2H), 7.27 (dt, *J* = 2.0, 1.2 Hz, 2H), 5.57 (dd, *J* = 10.6, 8.4 Hz, 1H), 3.37 (m, 1H), 3.00 (m, 1H), 2.30 (t, *J* = 1.5 Hz, 3H), 2.21 (s, 3H), 1.32 (s, 9H) ppm; **^{13}C NMR** (75 MHz, $CDCl_3$) δ 194.5, 167.5, 151.4, 138.2, 125.7, 125.6, 111.9, 83.2, 38.5, 34.6, 31.3, 29.5, 15.0 ppm; **IR** (Film) 2959 (m), 2867 (w), 1671 (m), 1595 (s), 1512 (w), 1382 (m) cm^{-1}; **GC/MS** (EI, 70 eV) m/z (%) = 258 (100) [M^+], 243 (69), 169 (25), 57 (25), 43 (57); **HRMS** (EI) calc. for [$C_{17}H_{22}O_2$]: 258.1620, found: 258.1622.

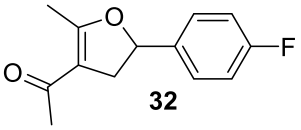

32

1-(5-(4-Fluorophenyl)-2-methyl-4,5-dihydrofuran-3-yl)ethan-1-one (32)[10]

Thermal condition:
The *title compound* was purified by column chromatography on silica gel eluting with *n*-pentane/Et_2O 3:1 (v/v) to afford 42 mg (76%) of **32** as a yellow oil.

UV-Light (180 W, Hg Lamp) condition:
The *title compound* was purified by column chromatography on silica gel eluting with *n*-pentane/Et_2O 3:1 (v/v) to afford 54.6 mg (62%) of **32** as a yellow oil.

R_f = 0.21 (*n*-pentane/Et_2O, 3:1 (v/v)); **^{1}H-NMR** (300 MHz, $CDCl_3$) δ 7.36-7.26 (m, 2H), 7.12-7.01 (m, 2H), 5.57 (dd, *J* = 8.4, 10.4 Hz, 1H), 3.46-3.32 (m, 1H), 3.00-2.88 (m, 1H), 2.30 (t, *J* = 1.4 Hz, 3H), 2.22 (s, 3H) ppm; **^{13}C-NMR** (75 MHz, $CDCl_3$) δ 194.3, 167.2, 162.5 (d, *J* = 245.5 Hz), 137.1 (d, *J* = 3.2 Hz), 127.5 (d, *J* = 8.2 Hz), 115.6 (d, *J* = 21.2 Hz), 111.9, 82.5, 38.8, 29.5, 15.0 ppm; **IR** (ATR) 2924, 1671, 1593, 1510, 1424, 1216, 929, 833, 624. 611 cm^{-1}; **HRMS** (ESI) calc. for [$C_{13}H_{13}FO_2$ + Na^+]: 243.0792, found: 243.0785.

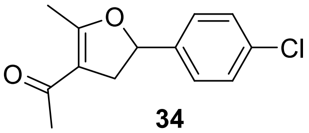

34

1-(5-(4-Chlorophenyl)-2-methyl-4,5-dihydrofuran-3-yl)ethan-1-one (34)[9]

Thermal condition:
The *title compound* was purified by column chromatography on silica gel eluting with *n*-pentane/Et_2O 3:1 (v/v) to afford 54 mg (92%) of **34** as a yellow oil.

UV-Light (180 W, Hg Lamp) condition:

The *title compound* was purified by column chromatography on silica gel eluting with *n*-pentane/Et_2O 3:1 (v/v) to afford 71.3 mg (75%) of **34** as a yellow oil.

R_f = 0.18 (*n*-pentane/Et_2O, 3:1 (v/v)); **^{1}H-NMR** (300 MHz, CDCl3) δ 7.39-7.32 (m, 2H), 7.30-7.22 (m, 2H), 5.56 (dd, *J* = 8.3, 10.7 Hz, 1H), 3.48-3.33 (m, 1H), 2.97-2.86 (m, 1H), 2.30 (t, *J* = 1.5 Hz, 3H), 2.21 (s, 3H) ppm; **^{13}C-NMR** (75 MHz, $CDCl_3$) δ 194.3, 167.2, 139.8, 134.0, 128.9, 127.0, 111.9, 82.3, 38.8, 29.5, 15.0 ppm; **IR** (ATR) 2921, 1672, 1592, 1492, 1381, 1215, 1134, 930, 624 cm^{-1}; **HRMS** (ESI) calc. for [$C_{13}H_{13}ClO_2$ + Na^+]: 259.0496, found: 259.0484.

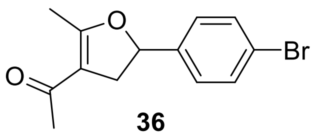

36

1-(5-(4-Bromophenyl)-2-methyl-4,5-dihydrofuran-3-yl)ethan-1-one (36)[10]

Thermal condition:
The *title compound* was purified by column chromatography on silica gel eluting with *n*-pentane/Et_2O 3:1 (v/v) to afford 68 mg (97%) of **36** as a yellow oil.

R_f = 0.22 (*n*-pentane/Et_2O, 3:1 (v/v)); **^{1}H NMR** (300 MHz, $CDCl_3$) δ 7.54 – 7.48 (m, 2H), 7.24 – 7.17 (m, 2H), 5.55 (dd, *J* = 10.7, 8.3 Hz, 1H), 3.39 (m, 1H), 2.91 (m, 1H), 2.31 (t, *J* = 1.5 Hz, 3H), 2.22 (s, 3H) ppm; **^{13}C NMR** (75 MHz, $CDCl_3$) δ 194.3, 167.2, 140.4, 131.9, 127.3, 122.1, 111.9, 82.4, 38.7, 29.5, 15.0 ppm; **IR** (Film) 2922 (w), 2866 (w), 1671 (m), 1601 (s), 1488 (m), 1382 (m) cm^{-1}; **GC/MS** (EI, 70 eV) m/z (%) = 280 (63) [M^+], 186 (28), 115 (23), 14,5 (100); **HRMS** (EI) calc. for [$C_{13}H_{13}BrO_2$]: 280.0099, found: 280.0097.

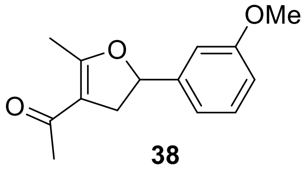

38

1-(5-(3-Methoxyphenyl)-2-methyl-4,5-dihydrofuran-3-yl)ethan-1-one (38)

Thermal condition:
The *title compound* was purified by column chromatography on silica gel eluting with *n*-pentane/Et_2O 2:1 (v/v) to afford 49 mg (85%) of **38** as a yellow oil.

UV-Light (180 W, Hg Lamp) condition:
The *title compound* was purified by column chromatography on silica gel eluting with *n*-pentane/Et_2O 2:1 (v/v) to afford 86.4 mg (93%) of **38** as a yellow oil.

R_f = 0.27 (*n*-pentane/Et_2O, 2:1 (v/v)); **^{1}H-NMR** (300 MHz, $CDCl_3$) δ 7.34-7.26 (m, 3H), 6.94-6.82 (m, 1H), 5.56 (dd, *J* = 8.4, 10.7 Hz, 1H), 3.82 (s, 3H), 3.45-3.32 (m, 1H), 3.03-2.90 (m, 1H), 2.31 (t, *J* = 1.5 Hz, 3H), 2.21 (s, 3H) ppm; **^{13}C-NMR** (75 MHz, $CDCl_3$) δ 194.4, 167.4, 159.9, 142.9, 129.9, 117.8, 113.4, 111.9, 111.3, 83.0, 55.3, 38.8, 29.5, 15.0 ppm; **IR** (ATR) 2938, 2837, 1670, 1586, 1489, 1383,

1259, 1216, 926, 782, 697, 625 cm^{-1}; **HRMS** (ESI) calc. for [$C_{14}H_{16}O_3$ + Na^+]: 255.0992, found: 255.0974.

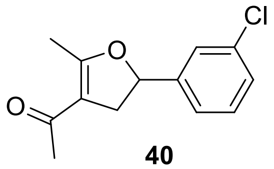

40

1-(5-(3-Chlorophenyl)-2-methyl-4,5-dihydrofuran-3-yl)ethan-1-one (40)[9]

Thermal condition:
The *title compound* was purified by column chromatography on silica gel eluting with *n*-pentane/Et_2O 2:1 (v/v) to afford 53 mg (90%) of **40** as a yellow oil.

UV-Light (180 W, Hg Lamp) condition:
The *title compound* was purified by column chromatography on silica gel eluting with *n*-pentane/Et_2O 2:1 (v/v) to afford 68.1 mg (72%) of **40** as a yellow oil.

R_f = 0.32 (*n*-pentane/Et_2O, 2:1 (v/v)); **^{1}H-NMR** (300 MHz, $CDCl_3$) δ 7.35-7.27 (m, 3H), 7.24-7.15 (m, 1H), 5.56 (dd, *J* = 8.3, 10.7 Hz, 1H), 3.47-3.35 (m, 1H), 2.99-2.87 (m, 1H), 2.32 (t, *J* = 1.5 Hz, 3H), 2.22 (s, 3H) ppm; **^{13}C-NMR** (75 MHz, $CDCl_3$) δ 194.3, 167.2, 143.4, 134.7, 130.1, 128.3, 125.7, 123.7, 111.9, 82.2, 38.8, 29.5, 15.0 ppm; **IR** (ATR) 2998, 2922, 1672, 1593, 1383, 1360, 1215, 923, 785, 693, 624 cm^{-1}; **HRMS** (ESI) calc. for [$C_{13}H_{13}ClO_2$ + Na^+]: 259.0496, found: 259.0492.

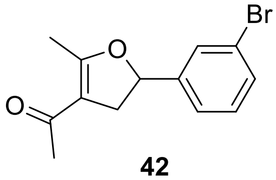

42

1-(5-(3-Bromophenyl)-2-methyl-4,5-dihydrofuran-3-yl)ethan-1-one (42)

Thermal condition:
The *title compound* was purified by column chromatography on silica gel eluting with *n*-pentane/Et_2O 2:1 (v/v) to afford 59 mg (84%) of **42** as a yellow oil.

R_f = 0.29 (*n*-pentane/Et_2O, 2:1 (v/v)); **^{1}H NMR** (300 MHz, $CDCl_3$) δ 7.48 – 7.43 (m, 2H), 7.26 – 7.23 (m, 2H), 5.55 (dd, *J* = 10.7, 8.3 Hz, 1H), 3.40 (m, 1H), 2.93 (m, 1H), 2.32 (t, *J* = 1.5 Hz, 3H), 2.22 (s, 3H) ppm; **^{13}C NMR** (75 MHz, $CDCl_3$) δ 194.3, 167.2, 143.7, 131.3, 130.4, 128.6, 124.2, 122.8, 111.9, 82.1, 38.8, 29.5, 14.9 ppm; **IR** (Film) 2922 (w), 2865 (w),1672 (m), 1595 (s), 1475 (w), 1424 (m), 1385 (m) cm^{-1}; **GC/MS** (ESI) m/z (%) = 303 (100) [M^+ + Na]; **HRMS** (ESI) calc. for [$C_{13}H_{13}BrO_2$ + Na^+]: 302.9991, found: 302.9969.

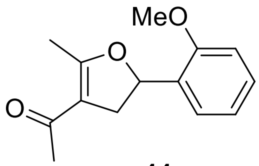

44

1-(5-(2-Methoxyphenyl)-2-methyl-4,5-dihydrofuran-3-yl)ethan-1-one (44)

Thermal condition:
The *title compound* was purified by column chromatography on silica gel eluting with *n*-pentane/Et_2O 2:1 (v/v) to afford 48 mg (83%) of **44** as a yellow oil.

UV-Light (180 W, Hg Lamp) condition:
The *title compound* was purified by column chromatography on silica gel eluting with *n*-pentane/Et_2O 2:1 (v/v) to afford 38.1 mg (41%) of **44** as a yellow oil.

R_f = 0.44 (*n*-pentane/Et_2O, 2:1 (v/v)); **^{1}H NMR** (300 MHz, $CDCl_3$) δ 7.32 – 7.25 (m, 2H), 6.97 (m, 1H), 6.90 (m, 1H), 5.87 (dd, *J* = 10.7, 8.0 Hz, 1H), 3.84 (s, 3H), 3.42 (m, 1H), 2.79 (m, 1H), 2.34 (t, *J* = 1.5 Hz, 3H), 2.18 (s, 3H) ppm; **^{13}C NMR** (75 MHz, $CDCl_3$) δ 194.8, 167.5, 155.9, 129.9, 128.9, 125.3, 120.6, 111.9, 110.4, 78.8, 55.4, 38.2, 29.5, 15.0 ppm; **IR** (Film) 2938 (w), 2838 (w), 1670 (m), 1588 (s), 1492 (m), 1462 (m), 1437 (m) cm^{-1}; **GC/MS** (ESI) m/z (%) = 255 (100) [M^+ + Na]; **HRMS** (ESI) calc. for [$C_{14}H_{16}O_3$ + Na^+]: 255.0992, found: 255.0978.

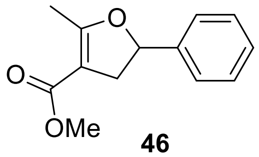

46

Methyl 2-methyl-5-phenyl-4,5-dihydrofuran-3-carboxylate (46)[9]

Thermal condition:
The *title compound* was purified by column chromatography on silica gel eluting with *n*-pentane/Et_2O 2:1 (v/v) to afford 40 mg (74%) of **46** as a colorless oil.

UV-Light (180 W, Hg Lamp) condition:
The *title compound* was purified by column chromatography on silica gel eluting with *n*-pentane/Et_2O 2:1 (v/v) to afford 70.7 mg (81%) of **46** as a colorless oil.

R_f = 0.27 (*n*-pentane/Et_2O, 10:1 (v/v)); **^{1}H-NMR** (300 MHz, $CDCl_3$) δ 7.41-7.27 (m, 5H), 5.59 (dd, *J* = 8.3, 10.7 Hz, 1H), 3.71 (s, 3H), 3.33 (qdd, *J* = 1.6, 10.8, 14.5 Hz, 1H), 2.91 (qdd, *J* = 1.6, 8.3, 14.5 Hz, 1H), 2.29 (t, *J* = 1.6 Hz, 3H) ppm; **^{13}C-NMR** (75 MHz, $CDCl_3$) δ 168.0, 166.4, 141.5, 128.7, 128.2, 125.7, 101.4, 83.2, 50.9, 37.9, 14.1 ppm; **IR** (ATR) 3032, 2949, 1690, 1647, 1495, 1435 cm^{-1}; **HRMS** (ESI): calc. for [$C_{13}H_{14}O_3$ + Na^+]: 241.0835, found: 241.0832.

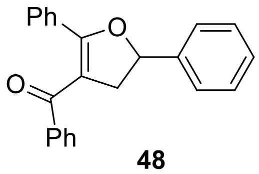

48

(2,5-Diphenyl-4,5-dihydrofuran-3-yl)(phenyl)methanone (48)[11]

Thermal condition:
The *title compound* was purified by column chromatography on silica gel eluting with *n*-pentane/Et_2O 5:1 (v/v) to afford 76 mg (93%) of **48** as a colorless solid.

UV-Light (180 W, Hg Lamp) condition:
The *title compound* was purified by column chromatography on silica gel eluting with *n*-pentane/Et_2O 5:1 (v/v) to afford 52.2 mg (40%) of **48** as a colorless solid.

R_f = 0.33 (*n*-pentane/Et_2O, 5:1 (v/v)); Mp. 111-112 °C; ^{1}H-NMR (250 MHz, $CDCl_3$) δ 7.51-7.05 (m, 15H), 5.85 (bt, J = 9.5 Hz, 1H), 3.71 (dd, J = 10.3, 15.1 Hz, 1H), 3.39 (dd, J = 9.0, 15.1 Hz, 1H) ppm; ^{13}C-NMR (63 MHz, $CDCl_3$) 193.4, 165.5, 141.1, 139.0, 131.2 , 130.1, 129.9, 129.5, 128.9, 128.8, 128.3, 127.7, 127.6, 125.9, 111.8, 83.2, 41.1 ppm; **IR** (ATR) 3061, 3030, 1611, 1592, 1573, 1492, 1446 cm^{-1}; **HRMS** (ESI): calc. for [$C_{23}H_{18}O_2$ + Na^+]: 349.1199, found: 349.1215.

10. ^{1}H and ^{13}C-NMR Spectra

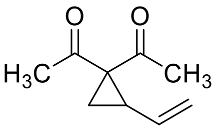

1

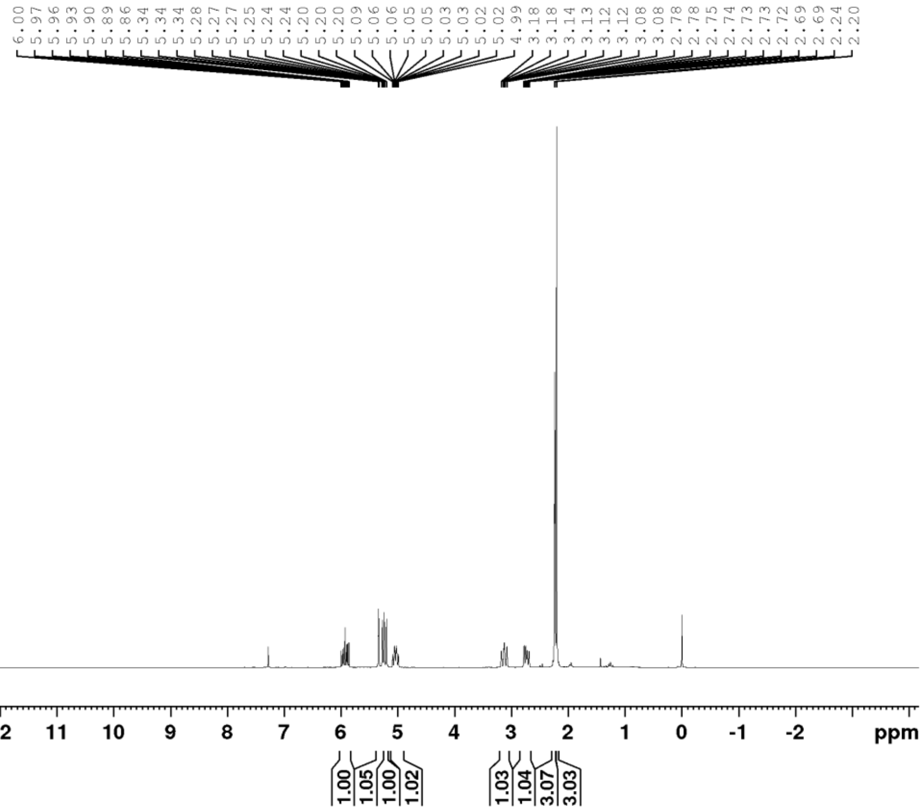

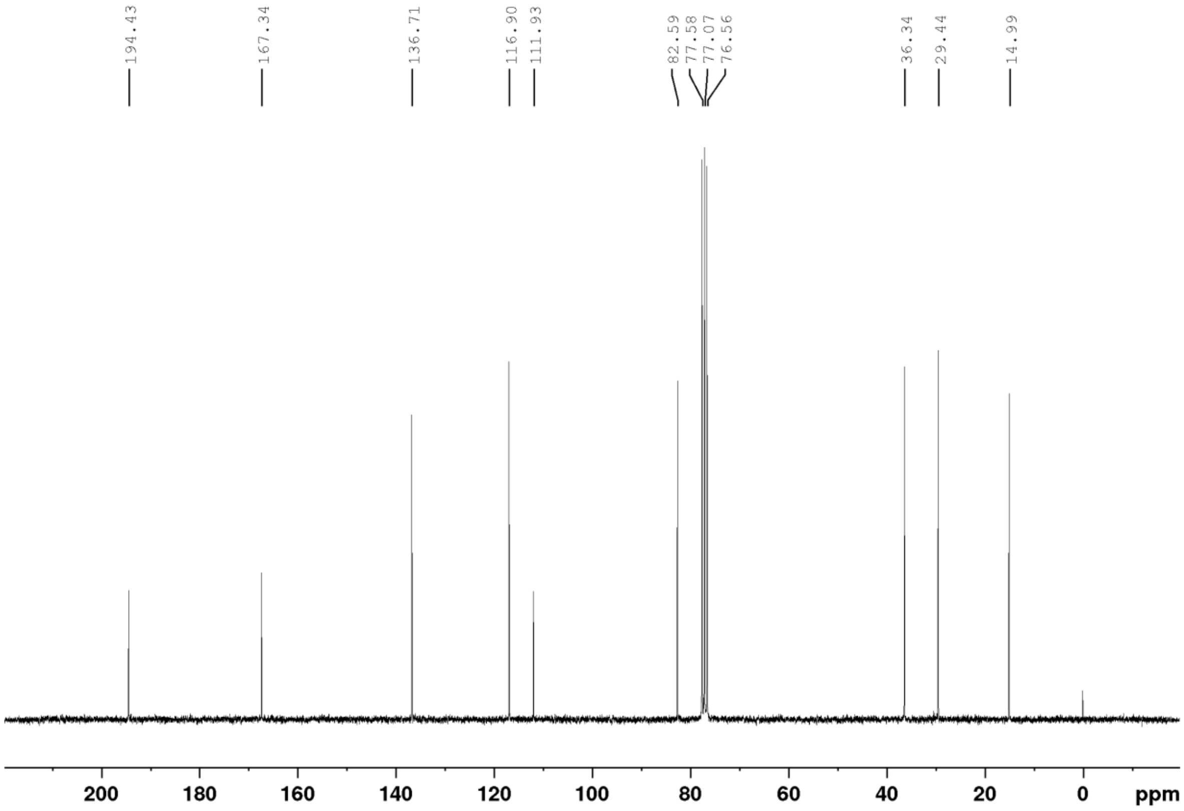

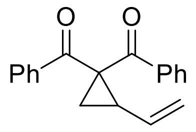
O
O
Ph
Ph

3

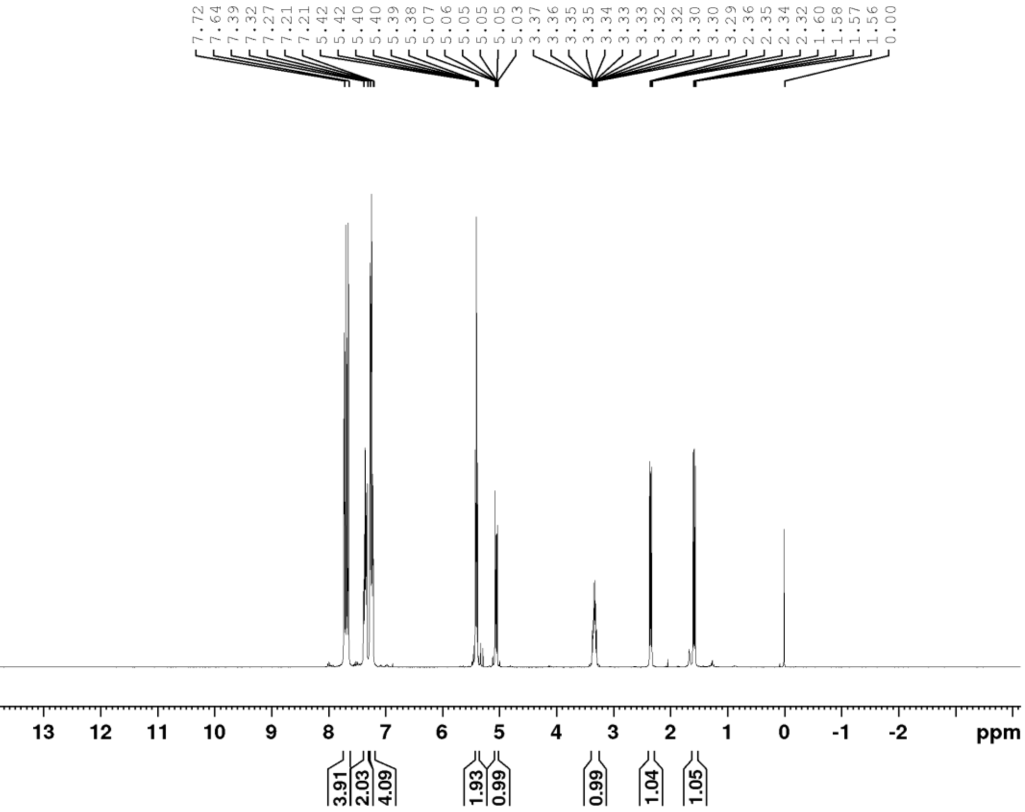
7.72
7.64
7.39
7.32
7.27
7.21
7.21
5.42
5.40
5.40
5.39
5.38
5.07
5.06
5.05
5.05
5.05
5.03
3.37
3.36
3.35
3.35
3.34
3.33
3.33
3.32
3.32
3.30
3.30
3.29
2.36
2.35
2.34
2.32
1.60
1.58
1.57
1.56
0.00
16 15 14 13 12 11 10 9 8 7 6 5 4 3 2 1 0 -1 -2 ppm
3.91
2.03
4.09
1.93
0.99
0.99
1.04
1.05

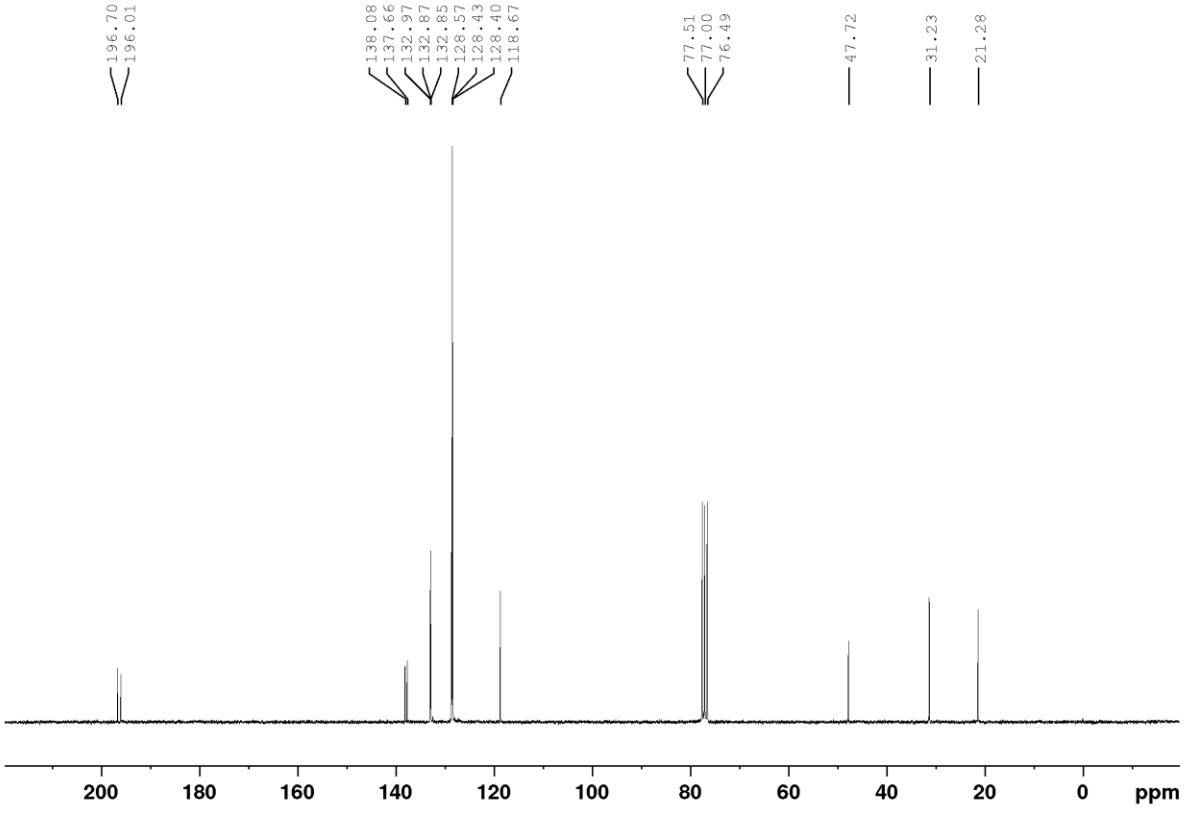
196.70
196.01
138.08
137.66
132.97
132.87
132.85
128.57
128.43
128.40
118.67
77.51
77.00
76.49
47.72
31.23
21.28
200 180 160 140 120 100 80 60 40 20 0 ppm

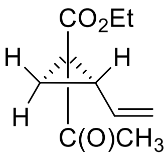
CO2Et
H
H
H
C(O)CH3

5a

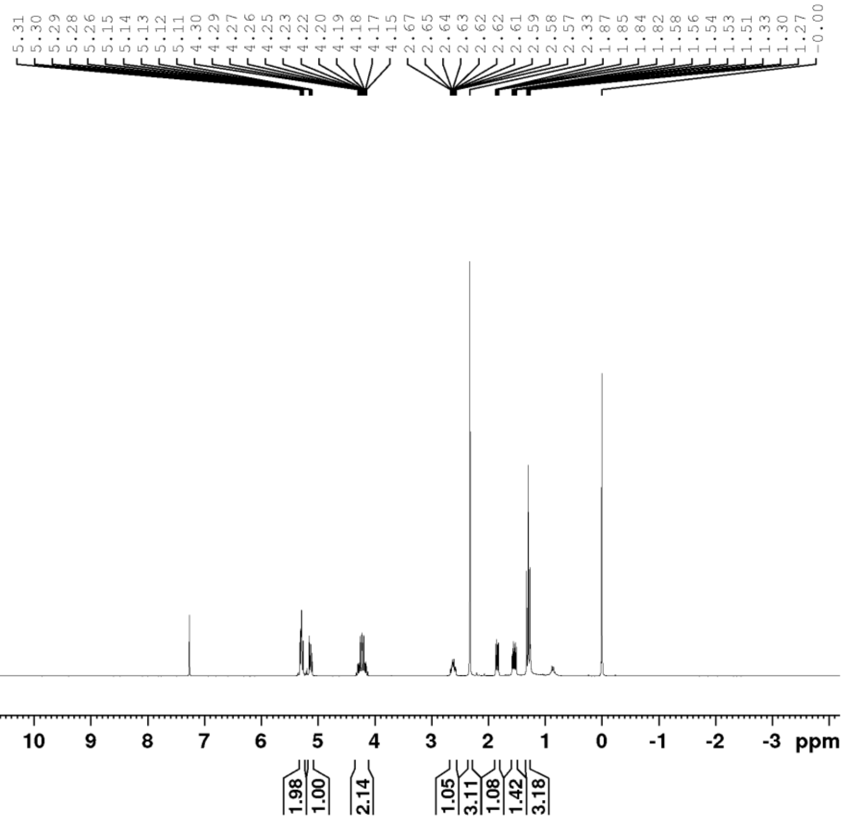
5.31
5.30
5.29
5.28
5.26
5.15
5.14
5.13
5.12
5.11
4.30
4.29
4.27
4.26
4.25
4.23
4.22
4.20
4.19
4.18
4.17
4.15
2.67
2.65
2.64
2.63
2.62
2.62
2.61
2.59
2.58
2.57
2.33
1.87
1.85
1.84
1.82
1.58
1.56
1.54
1.53
1.51
1.33
1.30
1.27
-0.00
16 15 14 13 12 11 10 9 8 7 6 5 4 3 2 1 0 -1 -2 -3 ppm
1.98
1.00
2.14
1.05
3.11
1.08
1.42
3.18

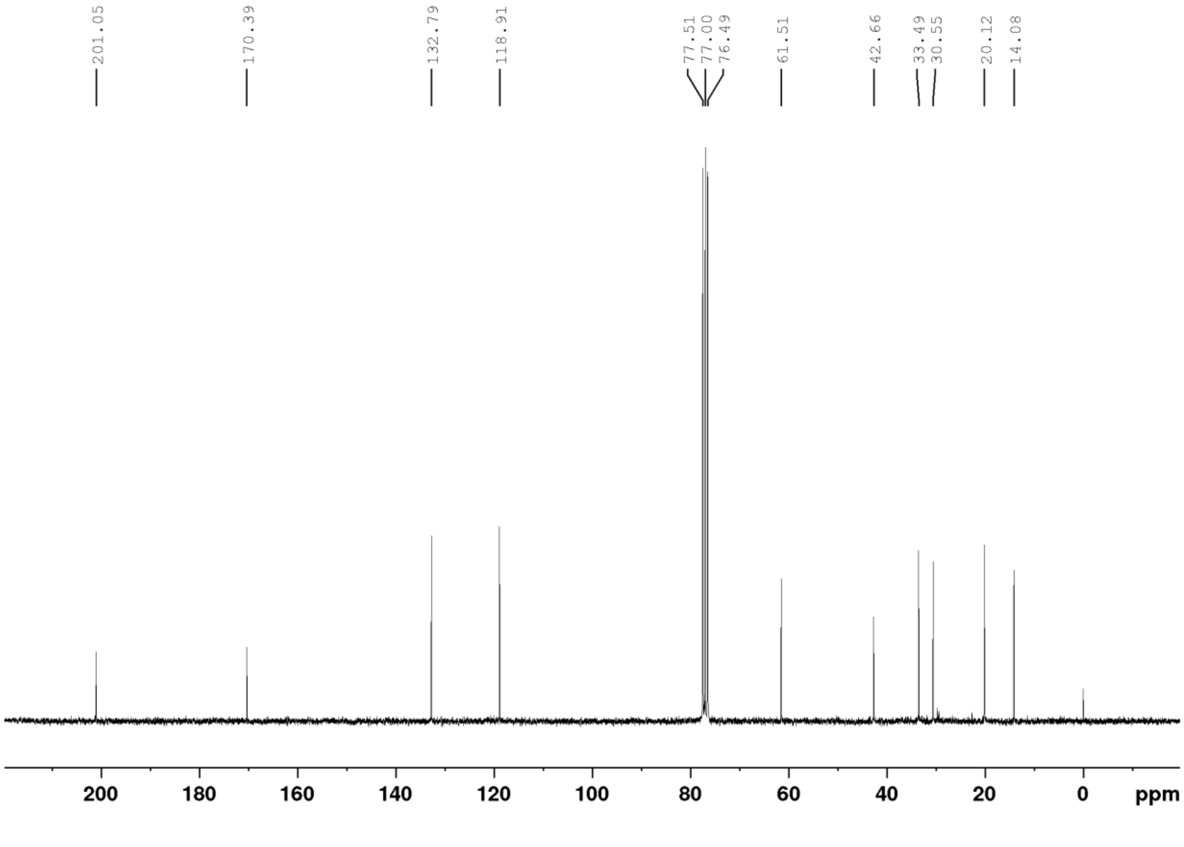
201.05
170.39
132.79
118.91
77.51
77.00
76.49
61.51
42.66
33.49
30.55
20.12
14.08
200 180 160 140 120 100 80 60 40 20 0 ppm

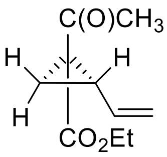
C(O)CH3
H
H
H
CO2Et

5b

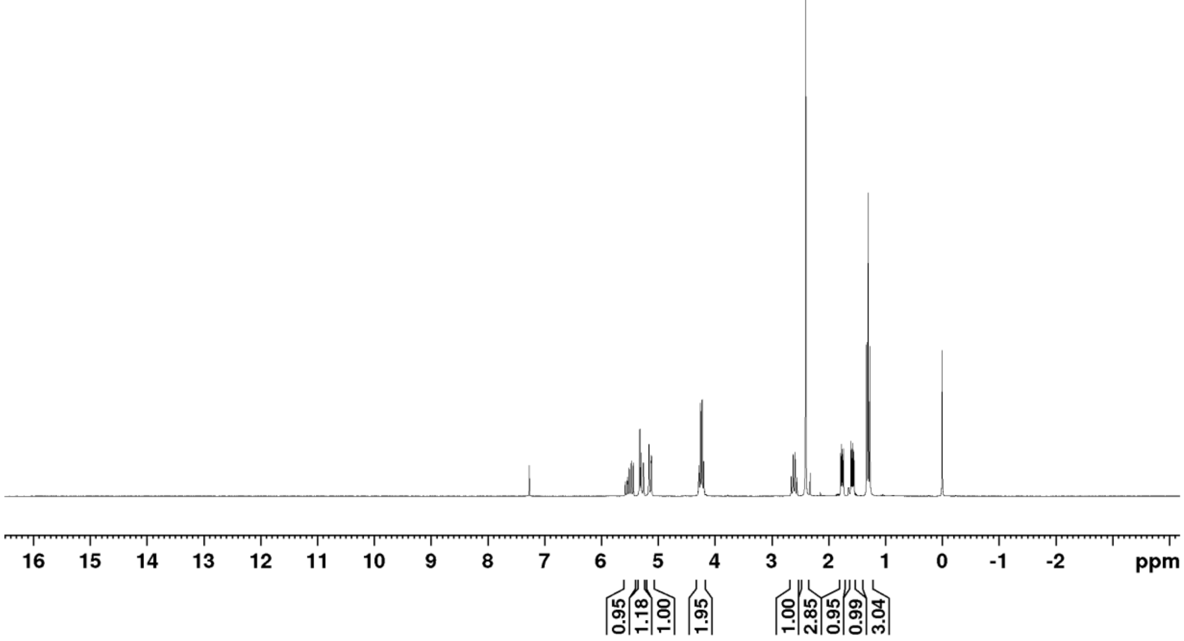
5.58 5.55 5.54 5.51 5.51 5.48 5.47 5.44 5.33 5.32 5.30 5.29 5.28 5.26 5.26 5.17 5.16 5.13 5.12 4.29 4.28 4.26 4.26 4.23 4.23 4.20 4.20 2.66 2.62 2.59 2.56 2.40 1.79 1.77 1.76 1.74 1.61 1.59 1.57 1.56 1.33 1.30 1.27 -0.00
16 15 14 13 12 11 10 9 8 7 6 5 4 3 2 1 0 -1 -2 ppm
0.95 1.18 1.00 1.95 1.00 2.85 0.95 0.99 3.04

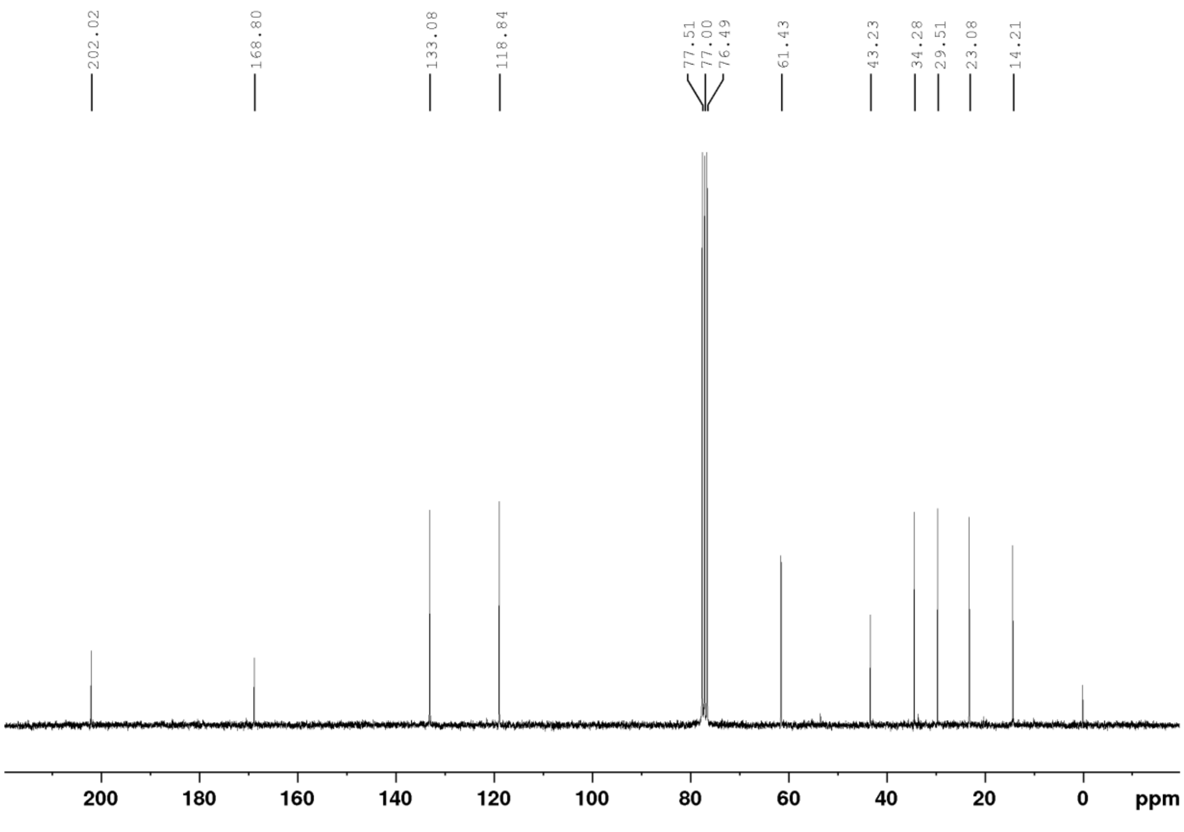
202.02
168.80
133.08
118.84
77.51 77.00 76.49
61.43
43.23
34.28
29.51
23.08
14.21
200 180 160 140 120 100 80 60 40 20 0 ppm

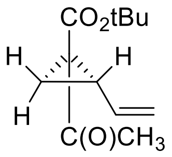
CO2tBu
H
H
H
C(O)CH3

7a

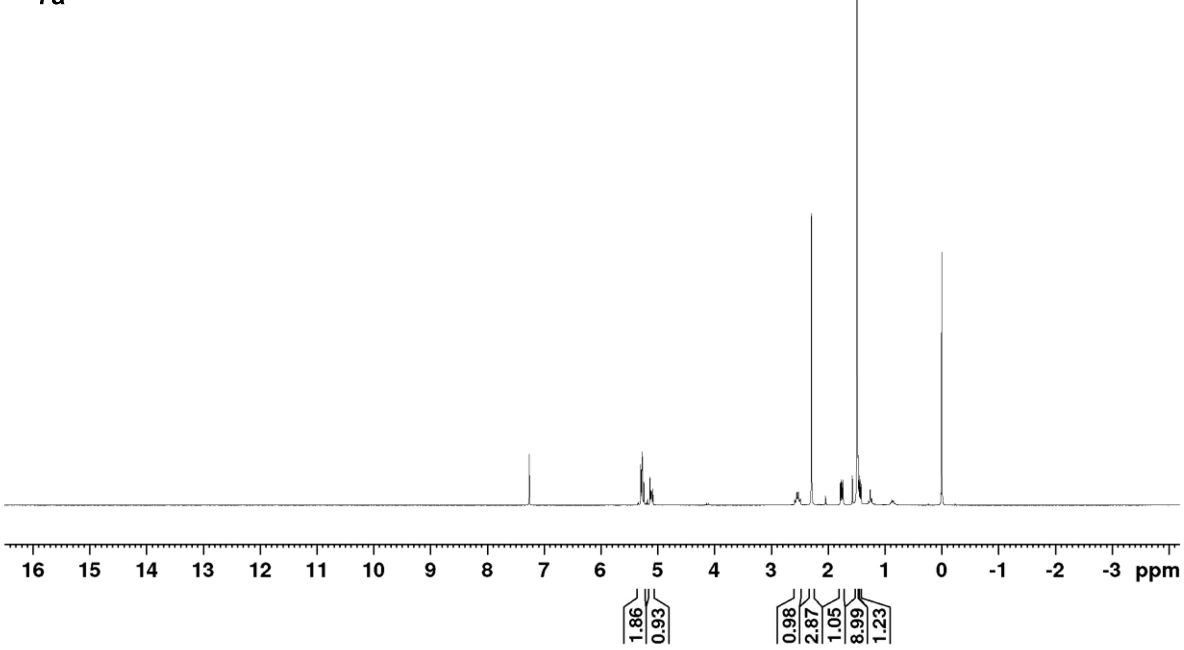
5.30 5.29 5.28 5.27 5.25 5.18 5.14 5.12 5.11 5.11 5.10 5.09 2.59 2.55 2.54 2.53 2.52 2.49 2.29 1.79 1.77 1.76 1.74 1.49 1.45 1.44 1.42 -0.00
16 15 14 13 12 11 10 9 8 7 6 5 4 3 2 1 0 -1 -2 -3 ppm
1.86 0.93 0.98 2.87 1.05 8.99 1.23

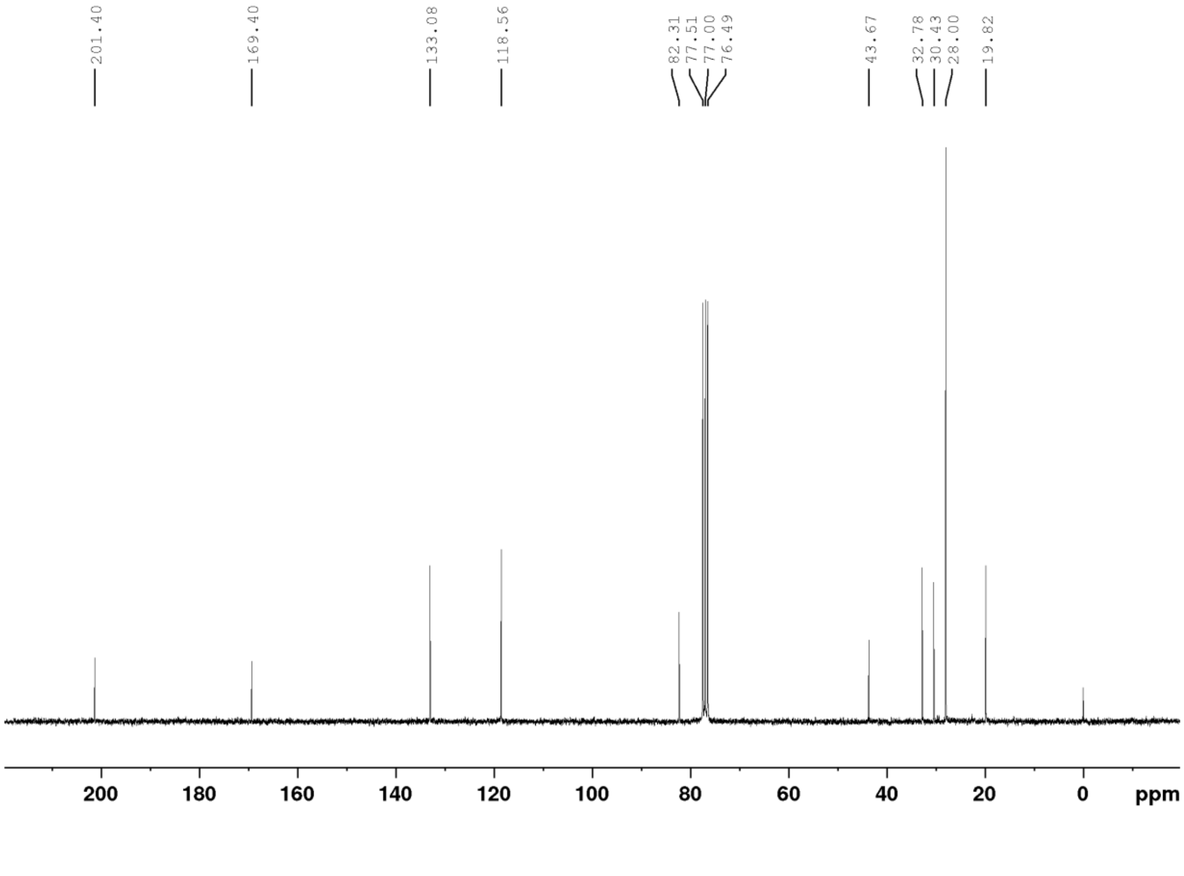
201.40 169.40 133.08 118.56 82.31 77.51 77.00 76.49 43.67 32.78 30.43 28.00 19.82
200 180 160 140 120 100 80 60 40 20 0 ppm

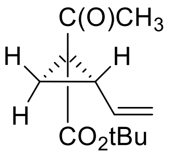
C(O)CH3
H
H
H
CO2tBu

7b

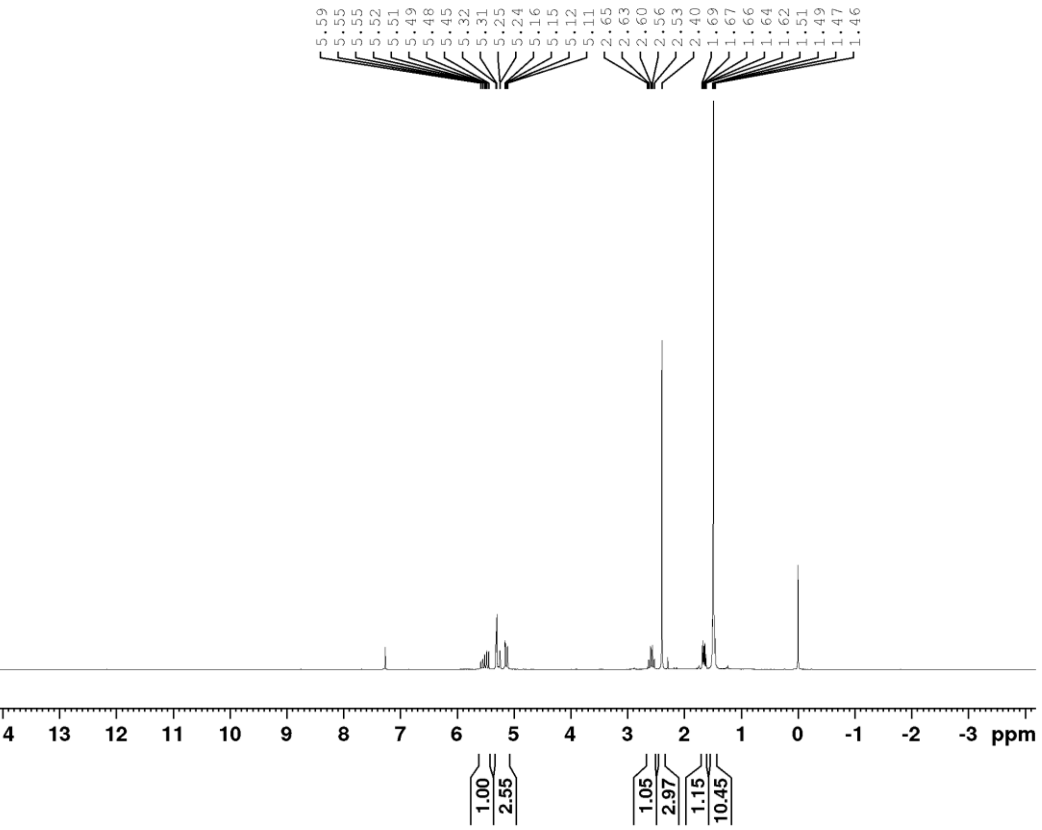
5.59 5.55 5.55 5.52 5.51 5.49 5.48 5.45 5.32 5.31 5.25 5.24 5.16 5.15 5.12 5.11 2.65 2.63 2.60 2.56 2.53 2.40 1.69 1.67 1.66 1.64 1.62 1.51 1.49 1.47 1.46
16 15 14 13 12 11 10 9 8 7 6 5 4 3 2 1 0 -1 -2 -3 ppm
1.00 2.55 1.05 2.97 1.15 10.45

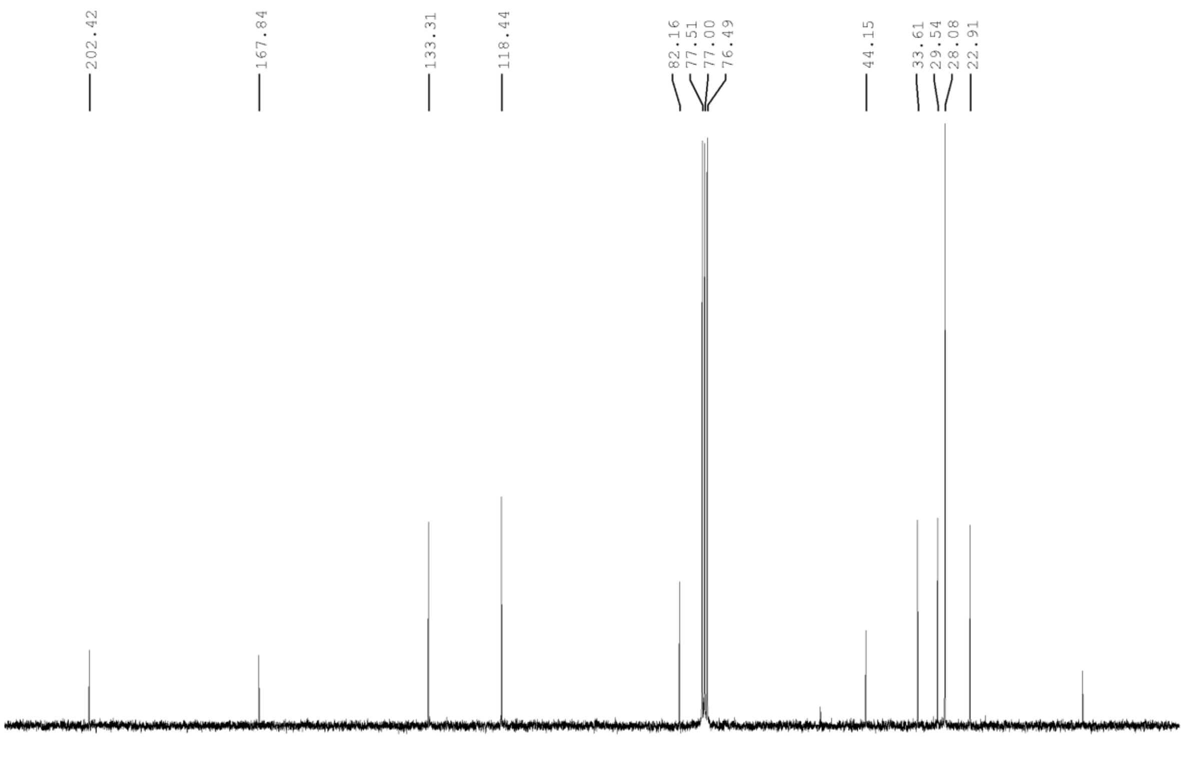
202.42 167.84 133.31 118.44 82.16 77.51 77.00 76.49 44.15 33.61 29.54 28.08 22.91
200 180 160 140 120 100 80 60 40 20 0 ppm

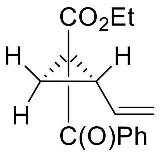
CO2Et
H
H
H
C(O)Ph

9a

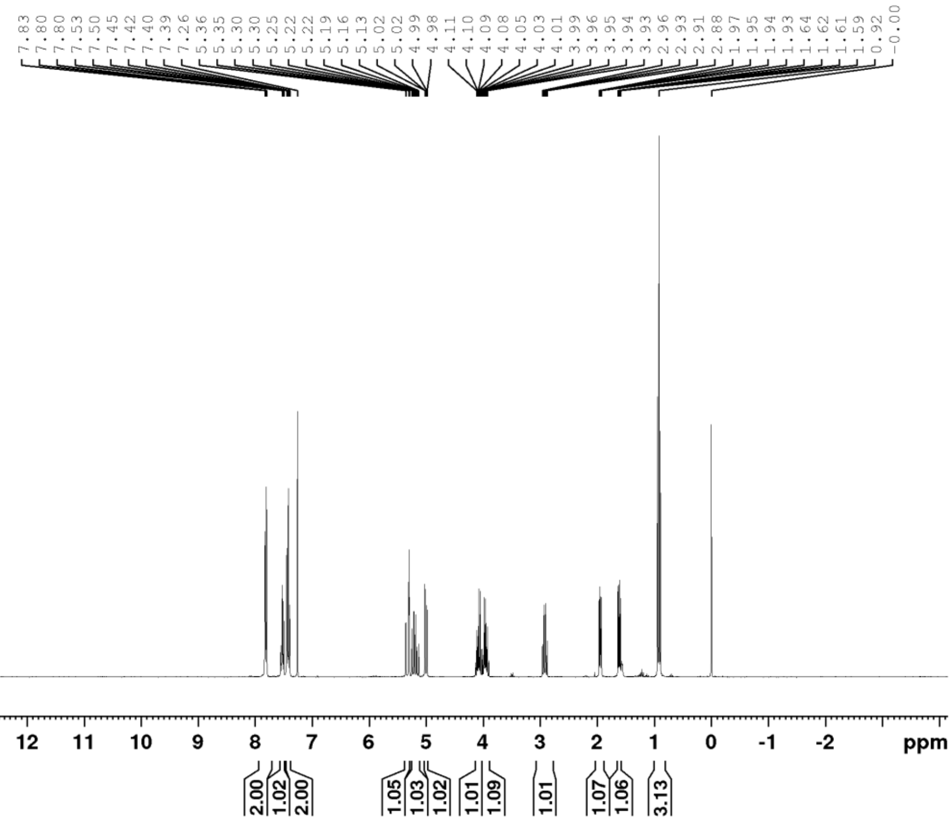
7.83 7.80 7.53 7.50 7.45 7.42 7.40 7.39 7.26 5.36 5.35 5.30 5.30 5.25 5.22 5.22 5.19 5.16 5.13 5.02 5.02 4.99 4.98 4.11 4.10 4.09 4.08 4.05 4.03 4.01 3.99 3.96 3.95 3.94 3.93 2.96 2.93 2.91 2.88 1.97 1.95 1.94 1.93 1.64 1.62 1.61 1.59 0.92 -0.00
2.00 1.02 2.00 1.05 1.03 1.02 1.01 1.09 1.01 1.07 1.06 3.13
ppm

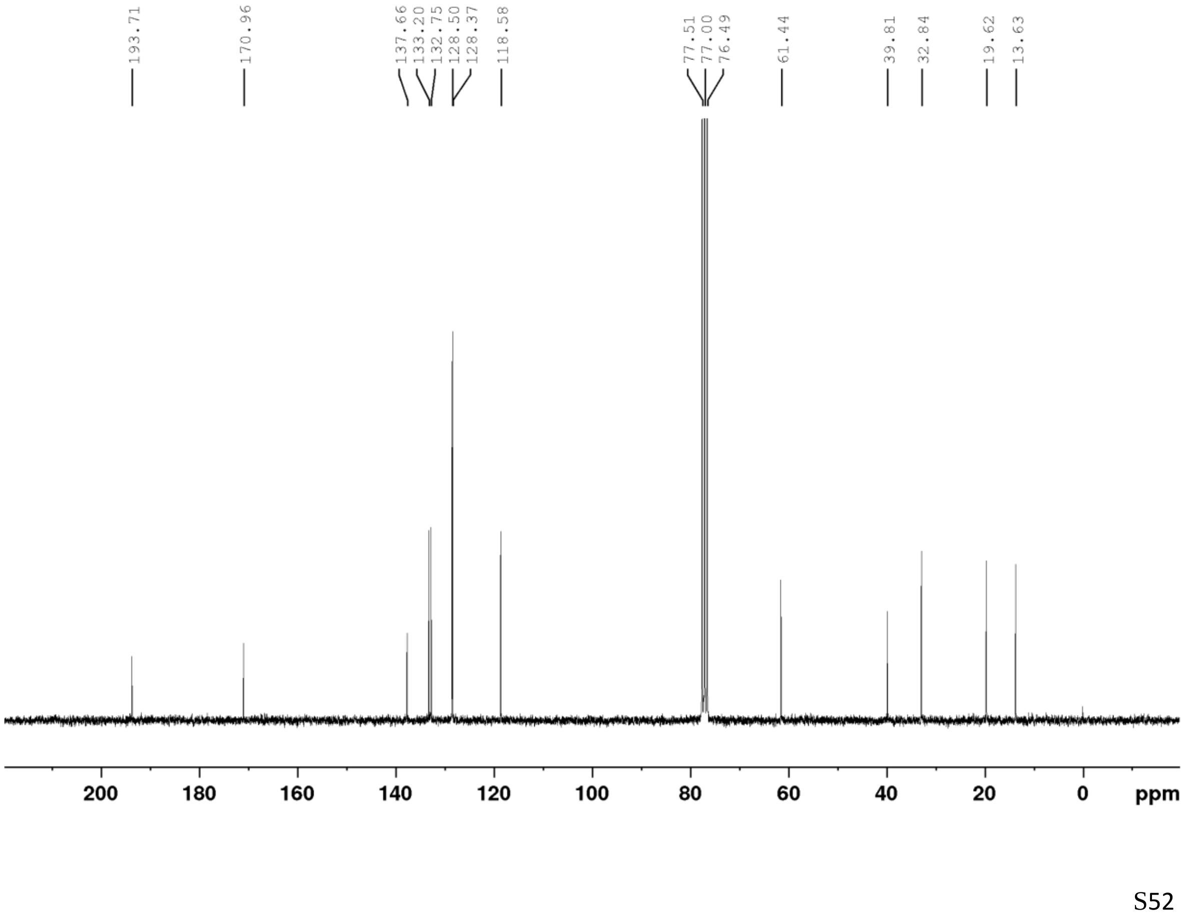
193.71 170.96 137.66 133.20 132.75 128.50 128.37 118.58 77.51 77.00 76.49 61.44 39.81 32.84 19.62 13.63
ppm

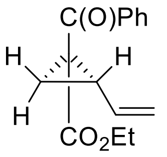
C(O)Ph
H
H
H
CO2Et

9b

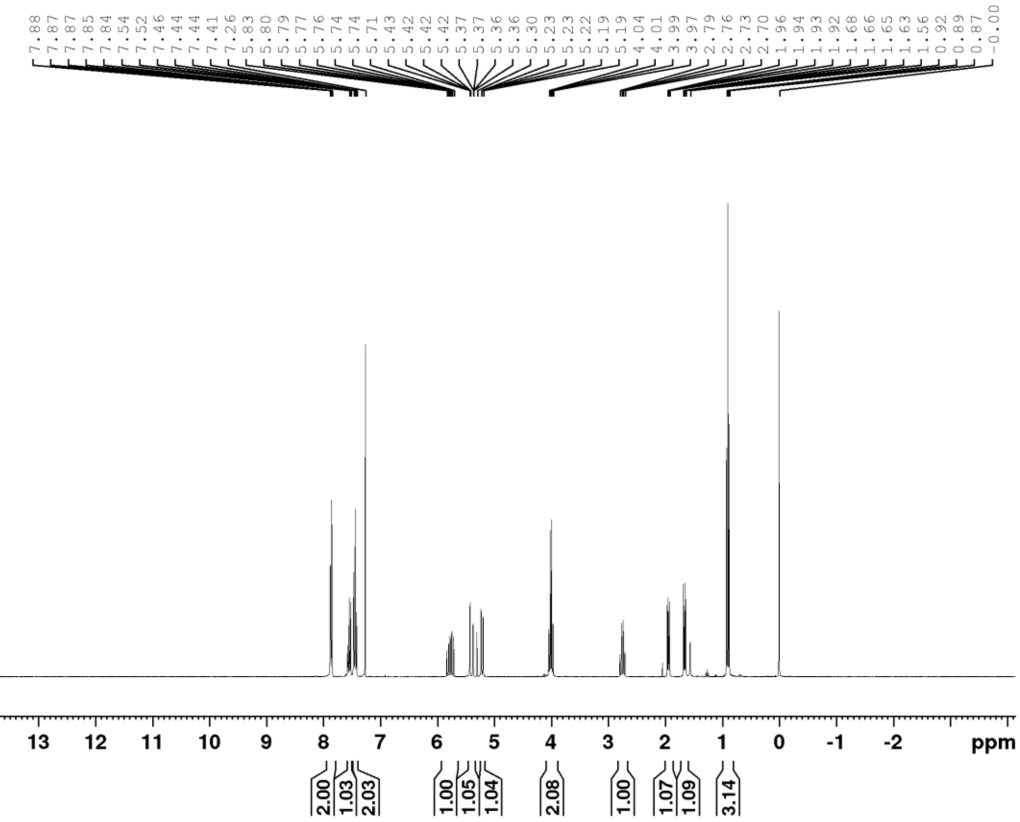
7.88 7.87 7.87 7.85 7.84 7.54 7.52 7.46 7.44 7.44 7.41 7.26 5.83 5.80 5.79 5.77 5.76 5.74 5.74 5.71 5.43 5.42 5.42 5.42 5.37 5.37 5.36 5.30 5.23 5.23 5.22 5.19 5.19 4.04 4.01 3.99 3.97 2.79 2.76 2.73 2.70 1.96 1.94 1.93 1.92 1.68 1.66 1.65 1.63 1.56 0.92 0.89 0.87 -0.00
16 15 14 13 12 11 10 9 8 7 6 5 4 3 2 1 0 -1 -2 ppm
2.00 1.03 2.03 1.00 1.05 1.04 2.08 1.00 1.07 1.09 3.14

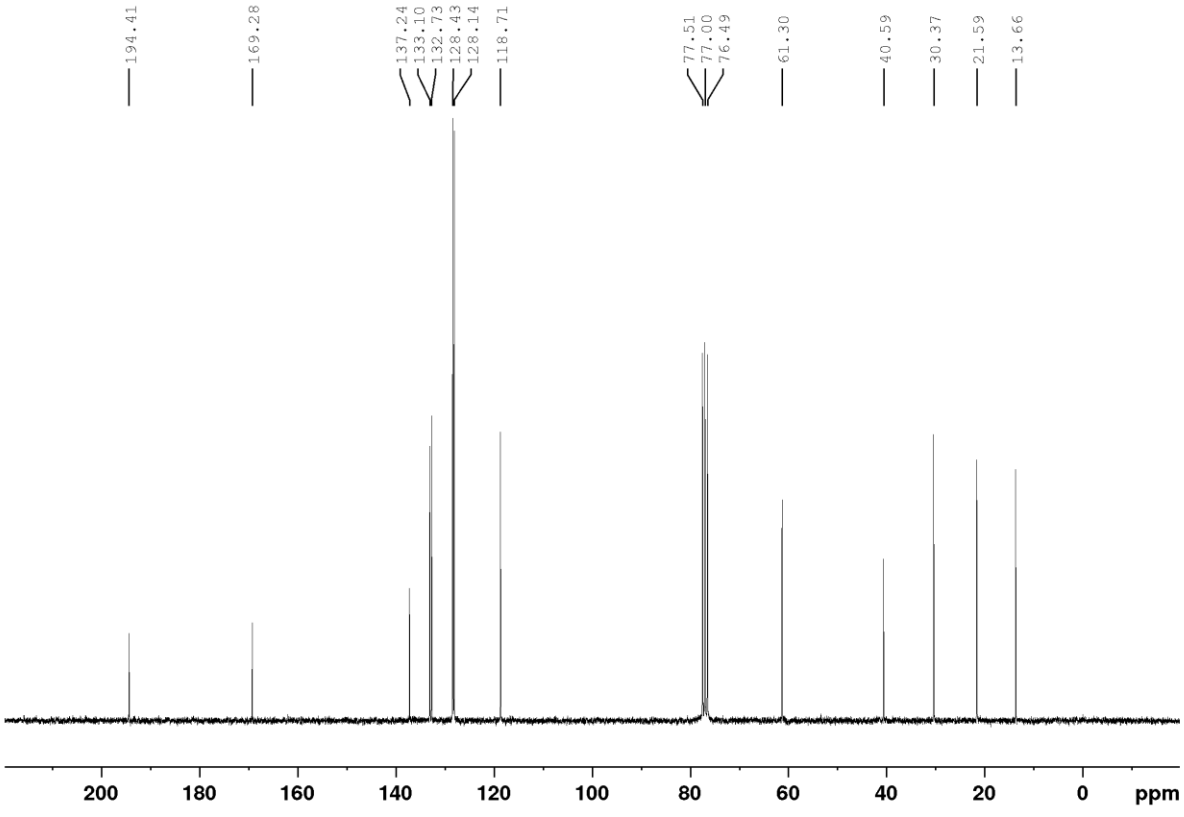
194.41 169.28 137.24 133.10 132.73 128.43 128.14 118.71 77.51 77.00 76.49 61.30 40.59 30.37 21.59 13.66
200 180 160 140 120 100 80 60 40 20 0 ppm

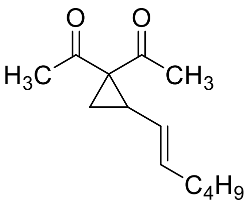
O
O
H3C
CH3
C4H9

11

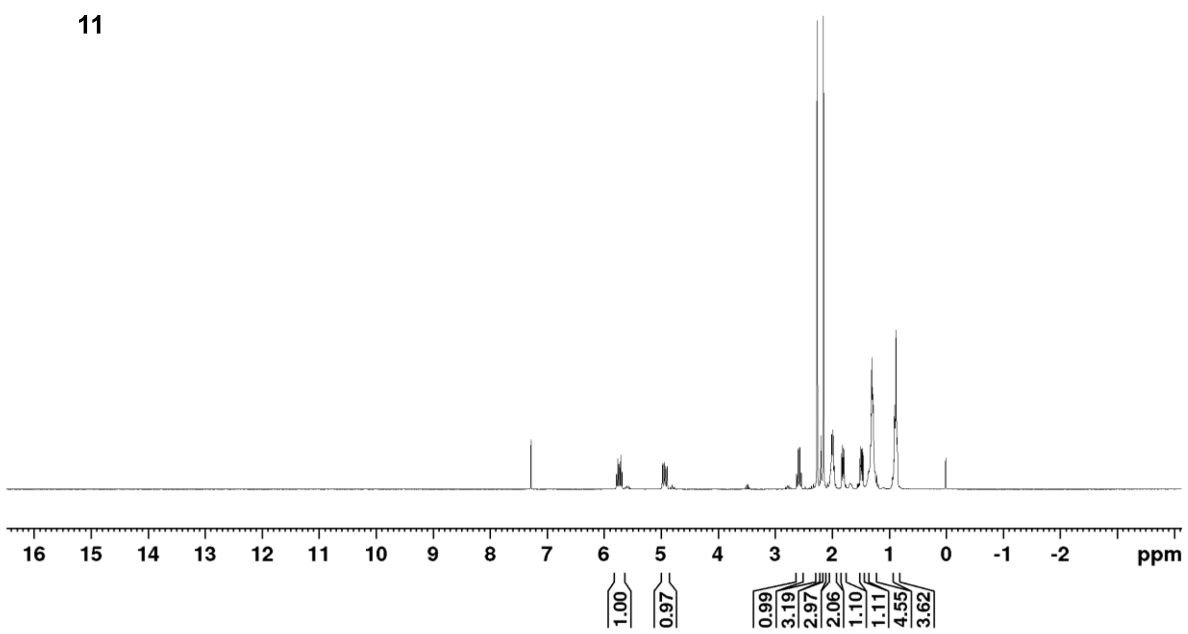
5.77 5.75 5.73 5.70 5.68 4.97 4.94 4.94 4.92 4.92 4.89 4.89 2.61 2.59 2.56 2.53 2.25 2.14 2.02 2.00 1.98 1.95 1.82 1.81 1.80 1.78 1.50 1.48 1.47 1.45 1.32 1.31 1.29 1.28 1.27 0.91 0.89 0.89 0.88 0.87 0.85
16 15 14 13 12 11 10 9 8 7 6 5 4 3 2 1 0 -1 -2 ppm
1.00 0.97 0.99 3.19 2.97 2.06 1.10 1.11 4.55 3.62

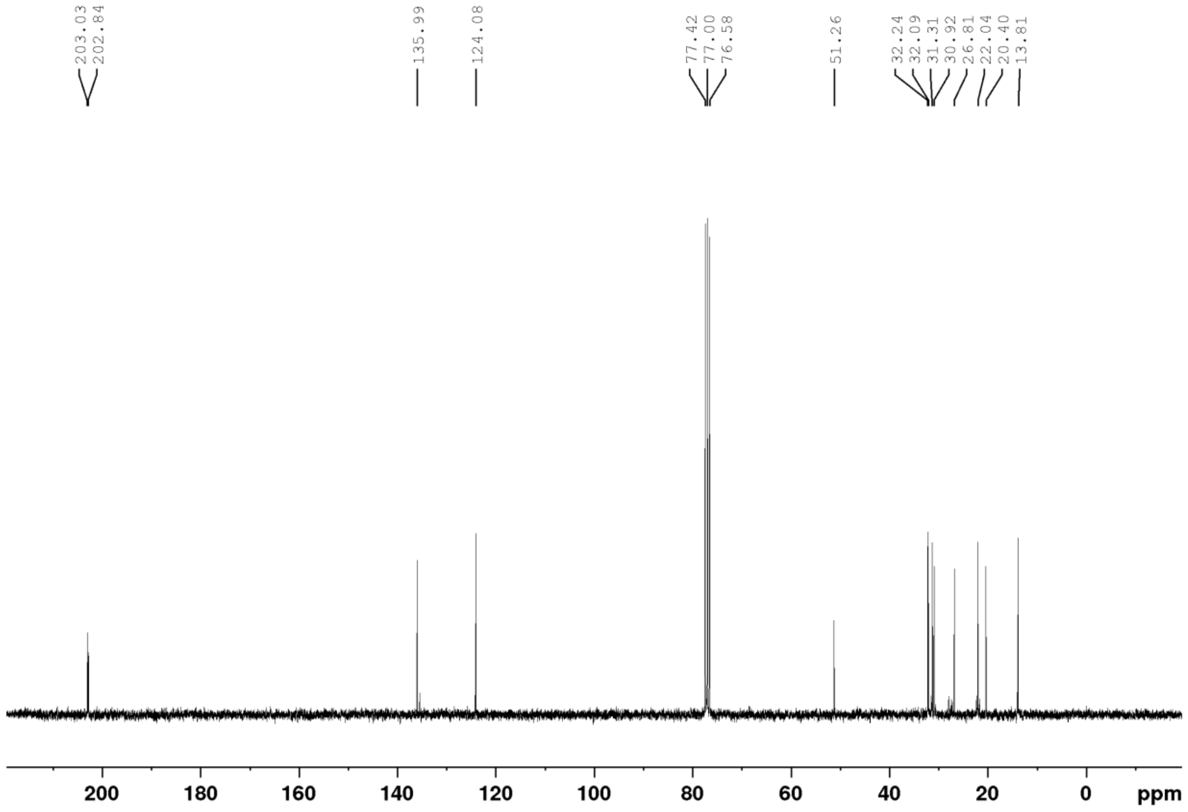
203.03 202.84 135.99 124.08 77.42 77.00 76.58 51.26 32.24 32.09 31.31 30.92 26.81 22.04 20.40 13.81
200 180 160 140 120 100 80 60 40 20 0 ppm

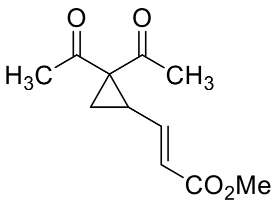
O
O
H3C
CH3
CO2Me

13

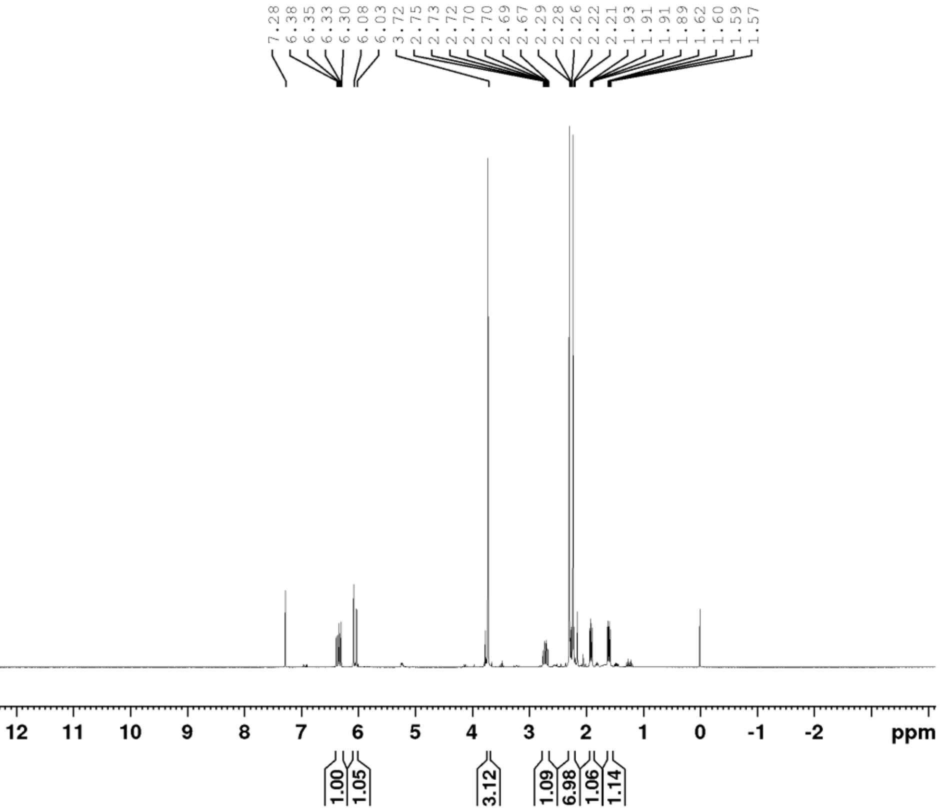
7.28
6.38
6.35
6.33
6.30
6.08
6.03
3.72
2.75
2.73
2.72
2.70
2.69
2.67
2.29
2.28
2.26
2.22
2.21
1.93
1.91
1.91
1.89
1.62
1.60
1.59
1.57
16 15 14 13 12 11 10 9 8 7 6 5 4 3 2 1 0 -1 -2 ppm
1.00
1.05
3.12
1.09
6.98
1.06
1.14

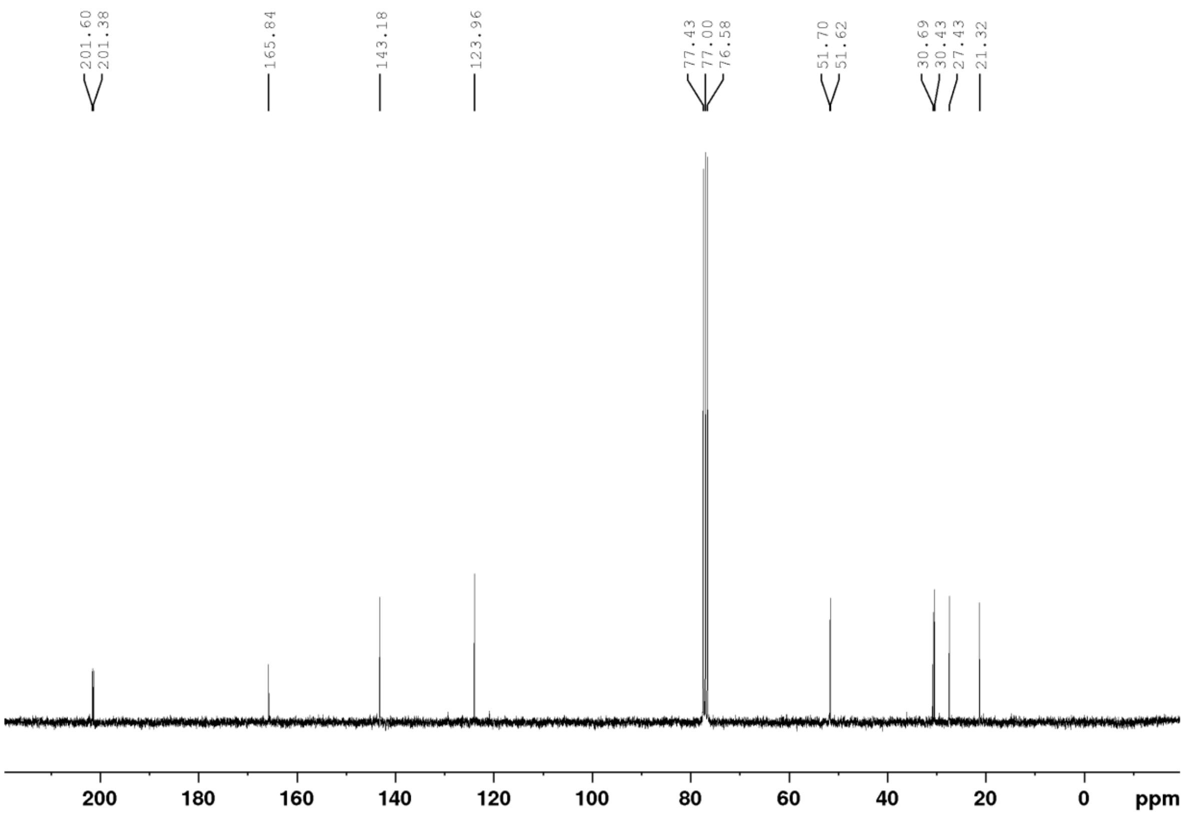
201.60
201.38
165.84
143.18
123.96
77.43
77.00
76.58
51.70
51.62
30.69
30.43
27.43
21.32
200 180 160 140 120 100 80 60 40 20 0 ppm

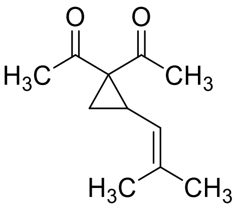
O
O
H_3C
CH_3
H_3C
CH_3

15

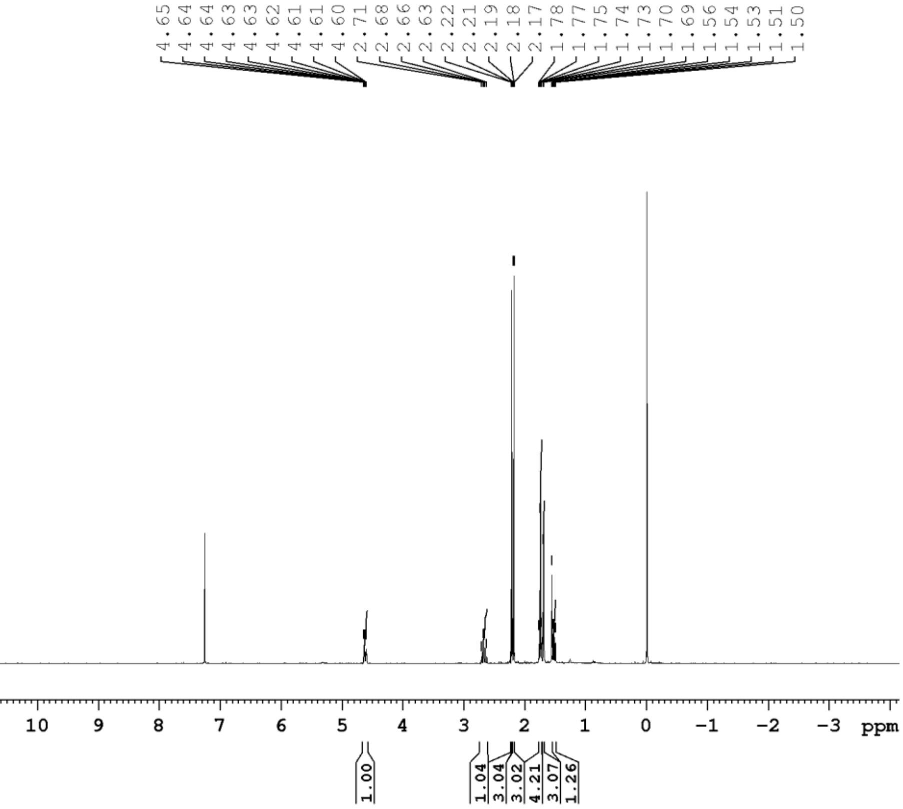
4.65
4.64
4.64
4.63
4.63
4.62
4.61
4.61
4.60
2.71
2.68
2.66
2.63
2.22
2.21
2.19
2.18
2.17
1.78
1.77
1.75
1.74
1.73
1.70
1.69
1.56
1.54
1.53
1.51
1.50
1.00
1.04
3.04
3.02
4.21
3.07
1.26
ppm

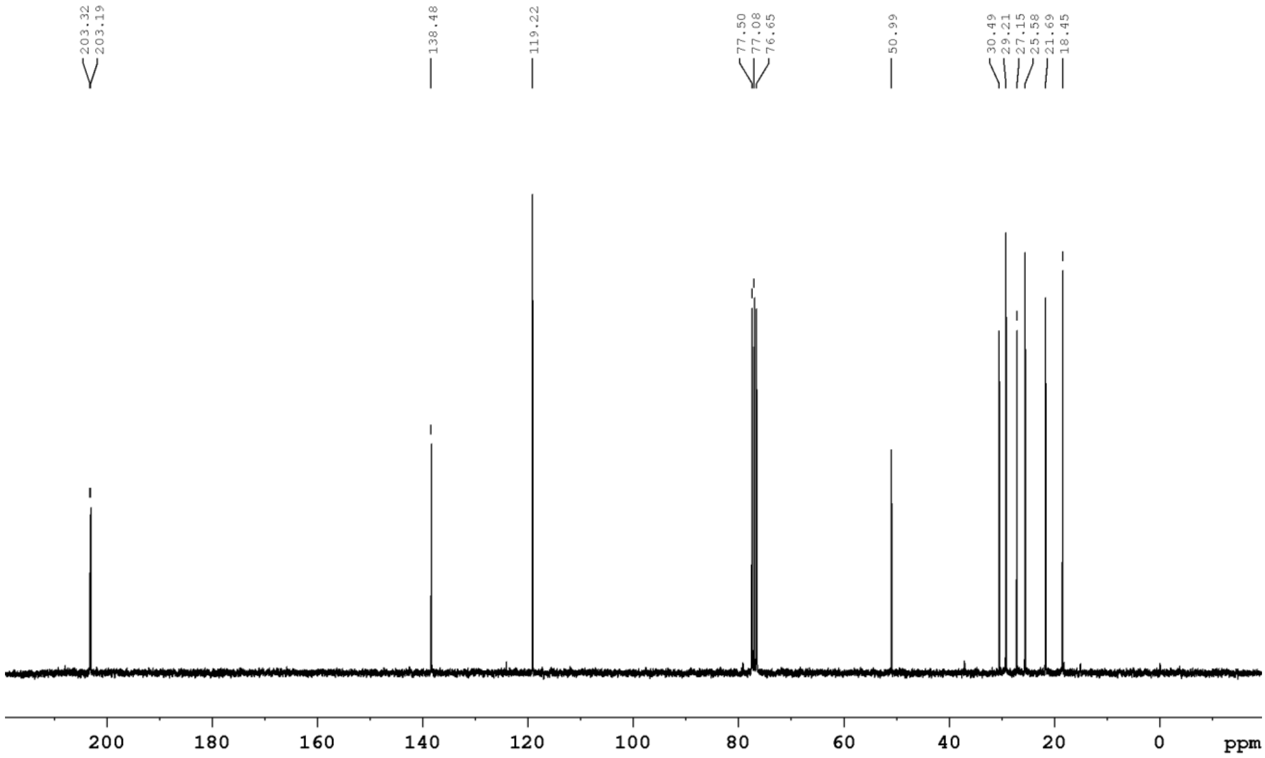
203.32
203.19
138.48
119.22
77.50
77.08
76.65
50.99
30.49
29.21
27.15
25.58
21.69
18.45
ppm

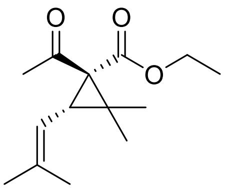

17

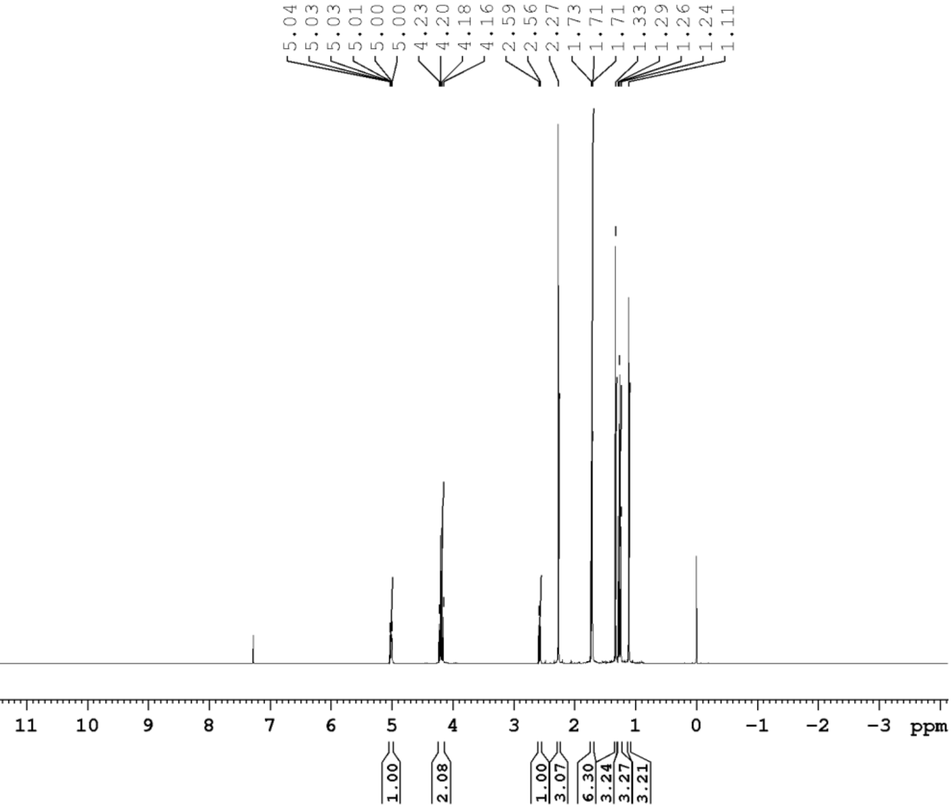
5.04
5.03
5.03
5.01
5.00
5.00
4.23
4.20
4.18
4.16
2.59
2.56
2.27
1.73
1.71
1.71
1.33
1.29
1.26
1.24
1.11
16 15 14 13 12 11 10 9 8 7 6 5 4 3 2 1 0 −1 −2 −3 ppm
1.00
2.08
1.00
3.07
6.30
3.24
3.27
3.21

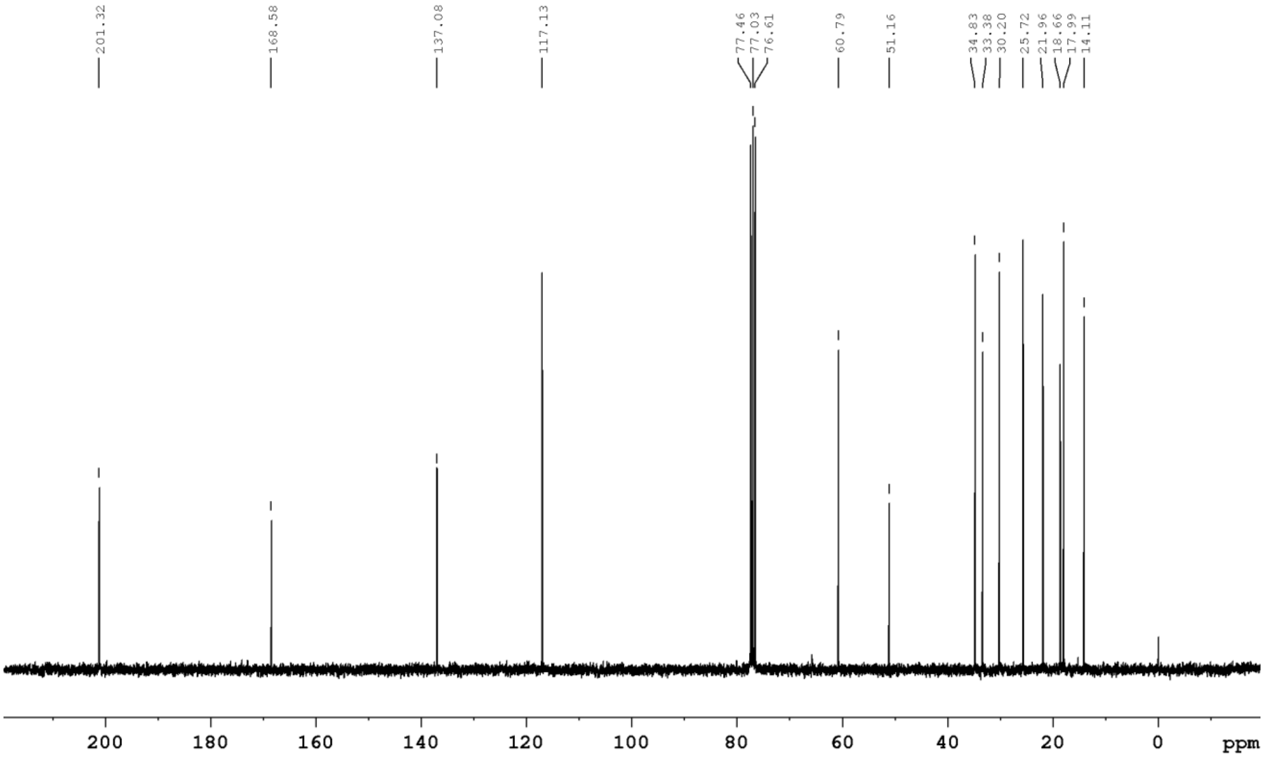
201.32
168.58
137.08
117.13
77.46
77.03
76.61
60.79
51.16
34.83
33.38
30.20
25.72
21.96
18.66
17.99
14.11
200 180 160 140 120 100 80 60 40 20 0 ppm

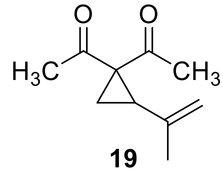

19

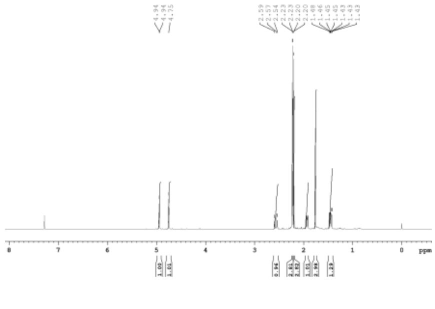

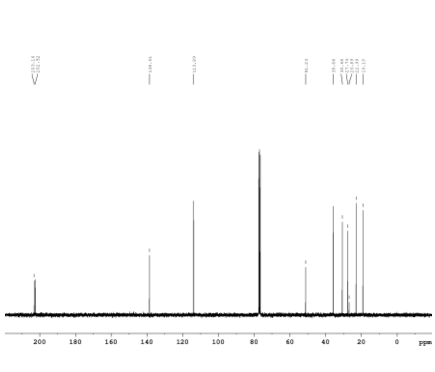

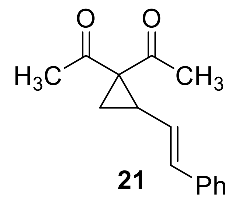

21

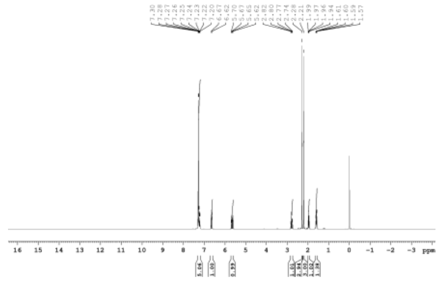

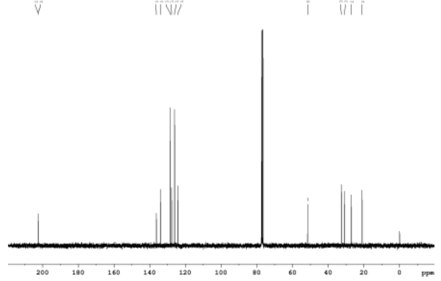

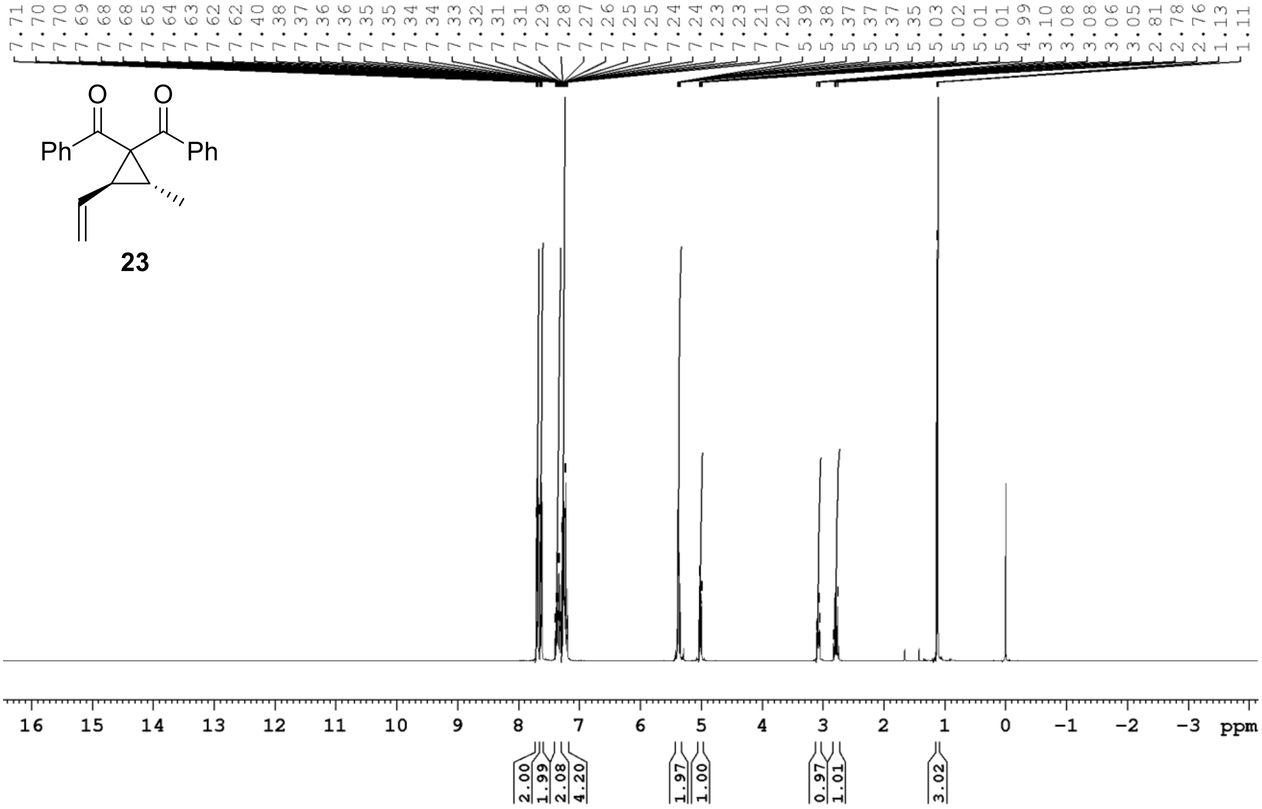
7.71 7.70 7.70 7.69 7.68 7.68 7.65 7.64 7.63 7.62 7.62 7.40 7.38 7.37 7.36 7.36 7.35 7.35 7.34 7.34 7.33 7.32 7.31 7.31 7.29 7.28 7.27 7.26 7.25 7.25 7.24 7.24 7.23 7.23 7.21 7.20 5.39 5.38 5.37 5.37 5.37 5.35 5.03 5.02 5.01 5.01 4.99 3.10 3.08 3.06 3.05 2.81 2.78 2.76 1.13 1.11
O
O
Ph
Ph
23
16 15 14 13 12 11 10 9 8 7 6 5 4 3 2 1 0 −1 −2 −3 ppm
2.00 1.99 2.08 4.20 1.97 1.00 0.97 1.01 3.02

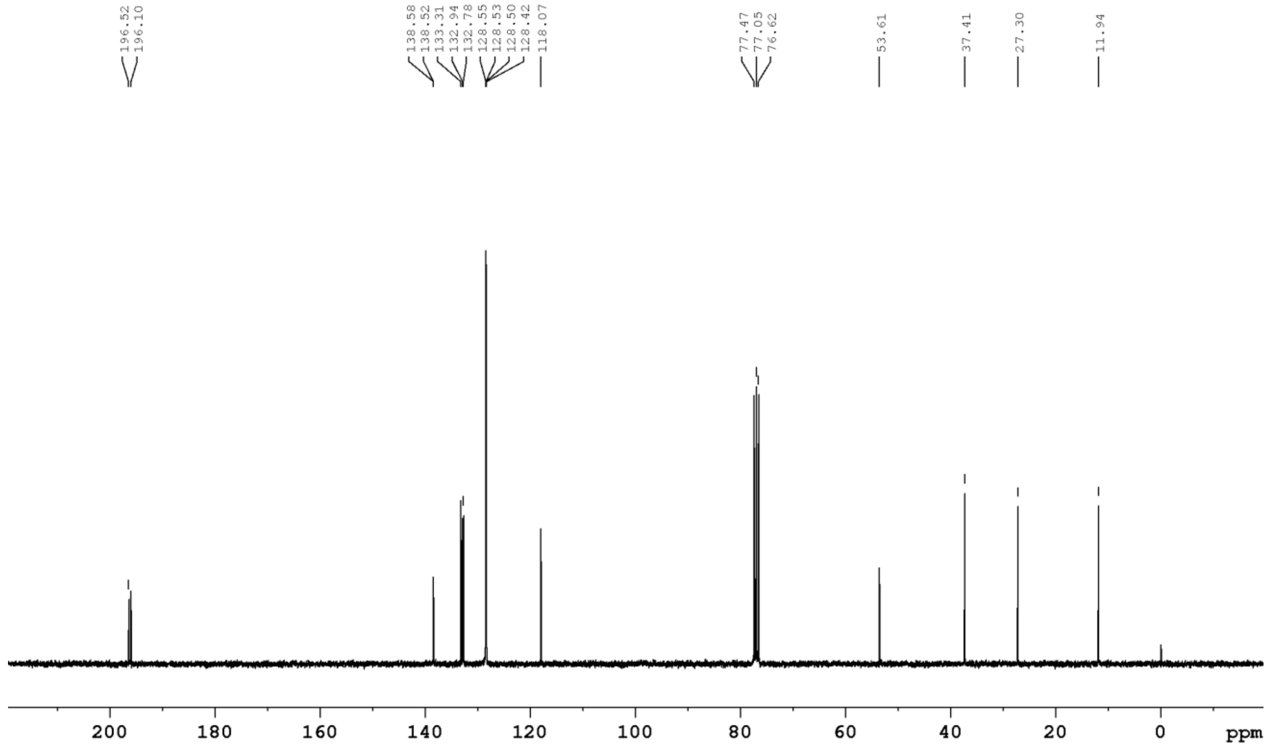
196.52 196.10
138.58 138.52 133.31 132.94 132.78 128.55 128.53 128.50 128.42 118.07
77.47 77.05 76.62
53.61
37.41
27.30
11.94
200 180 160 140 120 100 80 60 40 20 0 ppm

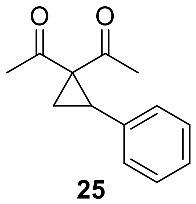

25

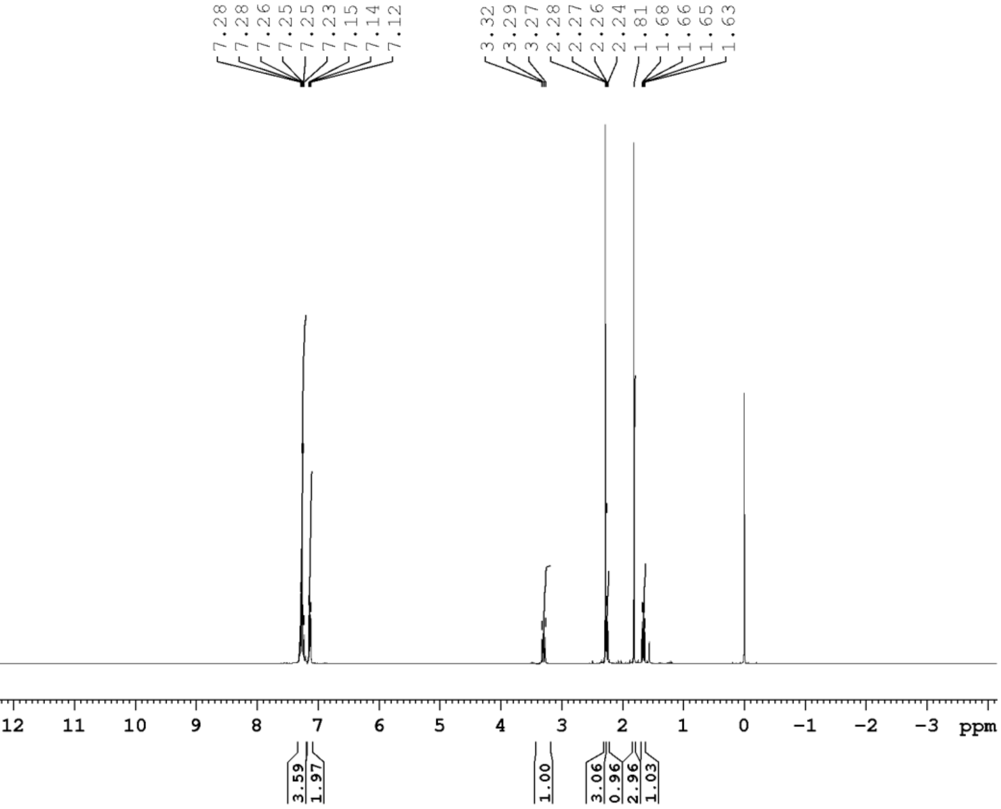

7.28 7.28 7.26 7.25 7.25 7.23 7.15 7.14 7.12
3.32 3.29 3.27 2.28 2.27 2.26 2.24 1.81 1.68 1.66 1.65 1.63
16 15 14 13 12 11 10 9 8 7 6 5 4 3 2 1 0 −1 −2 −3 ppm
3.59 1.97 1.00 3.06 0.96 2.96 1.03

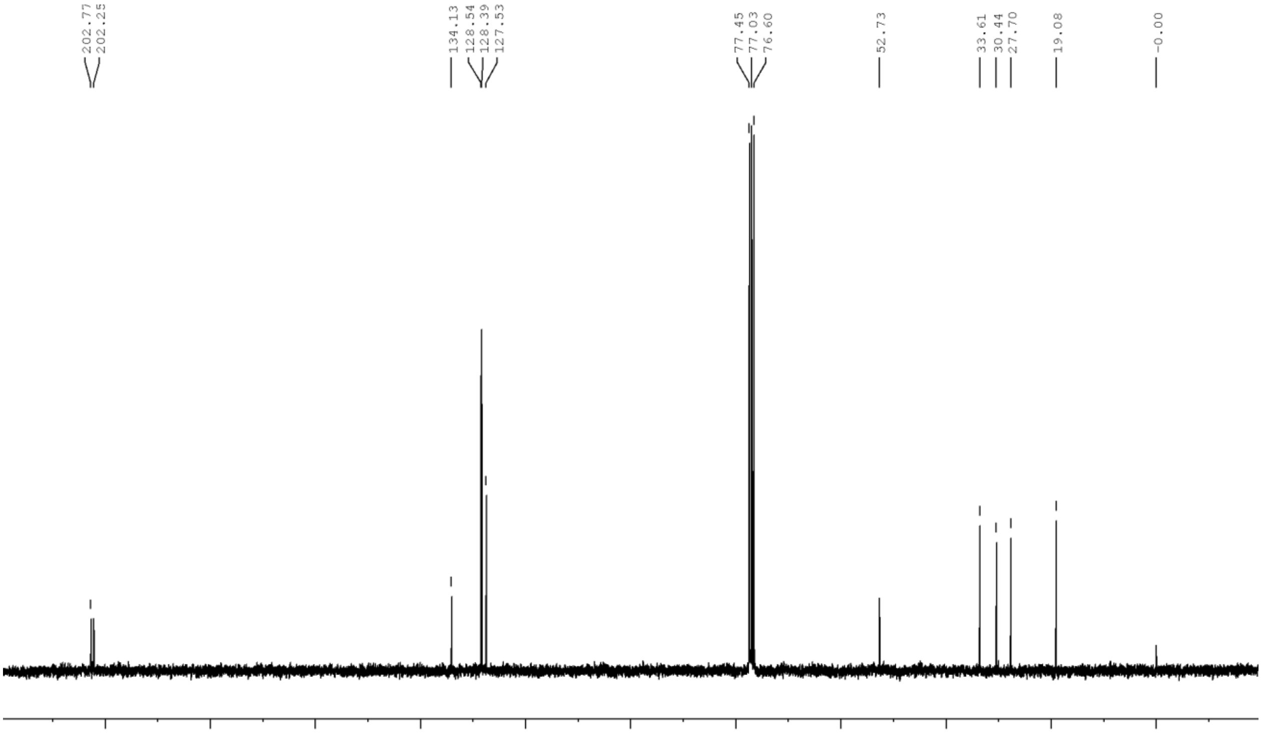

202.77 202.25
134.13 128.54 128.39 127.53
77.45 77.03 76.60
52.73
33.61 30.44 27.70
19.08
-0.00
200 180 160 140 120 100 80 60 40 20 0 ppm

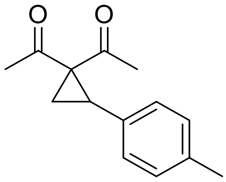

27

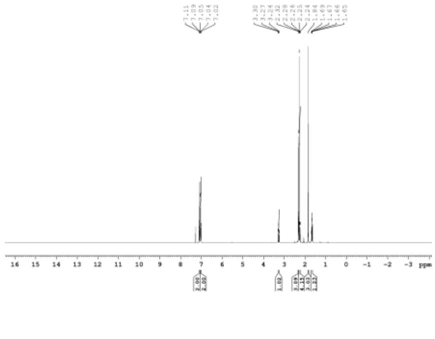

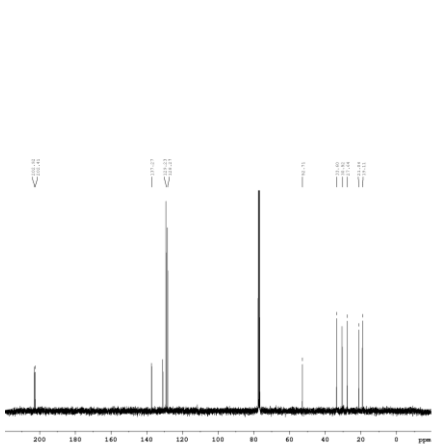

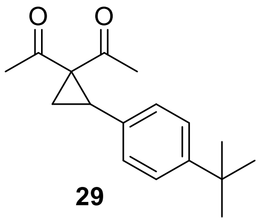

29

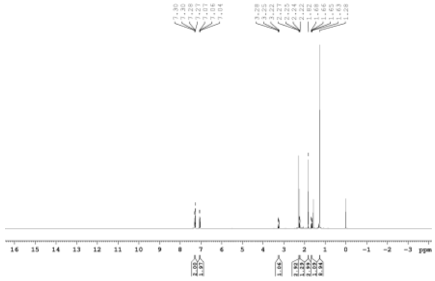

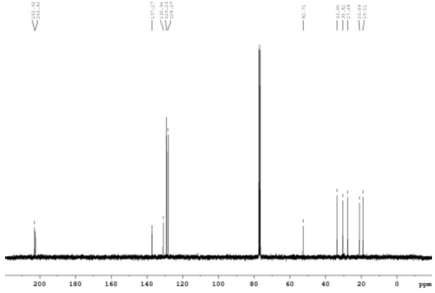

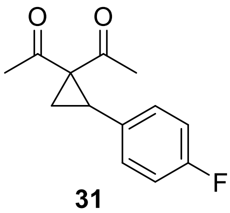
O
O
F

31

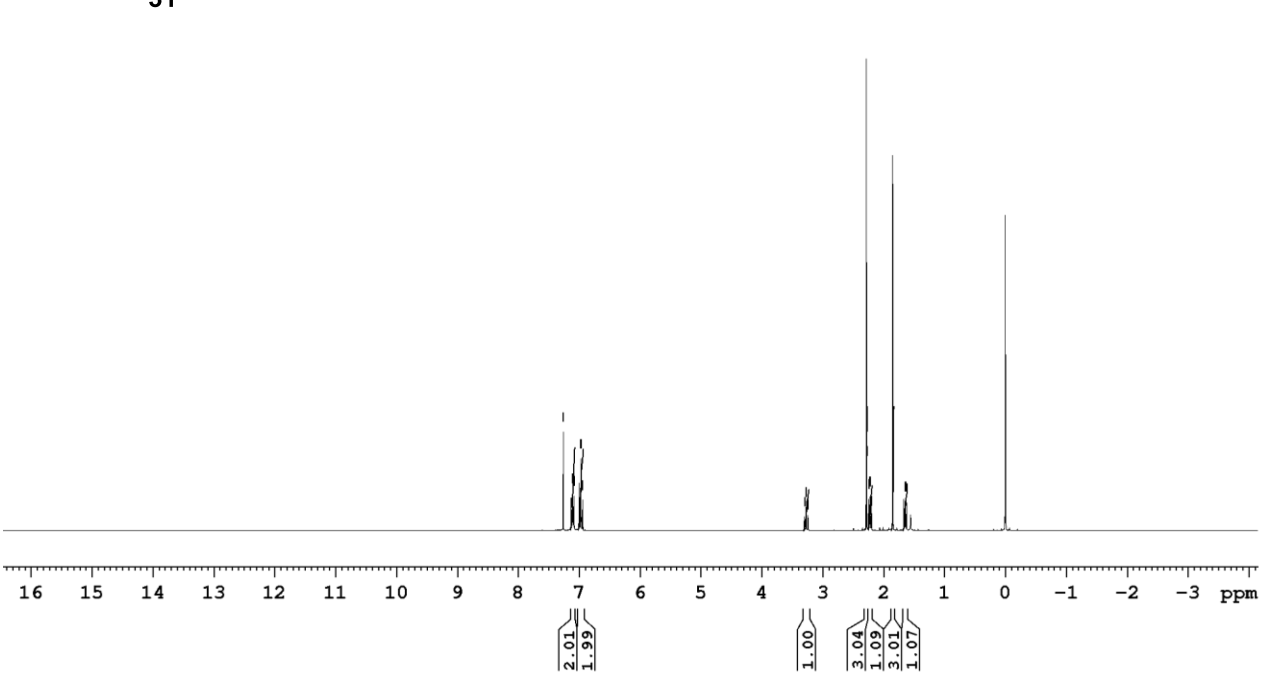
7.26 7.13 7.13 7.12 7.11 7.10 7.09 7.00 6.99 6.98 6.98 6.97 6.97 6.95 6.94 3.30 3.27 3.24 2.28 2.27 2.25 2.23 2.22 2.20 1.85 1.67 1.65 1.64 1.62
2.01 1.99 1.00 3.04 1.09 3.01 1.07
16 15 14 13 12 11 10 9 8 7 6 5 4 3 2 1 0 −1 −2 −3 ppm

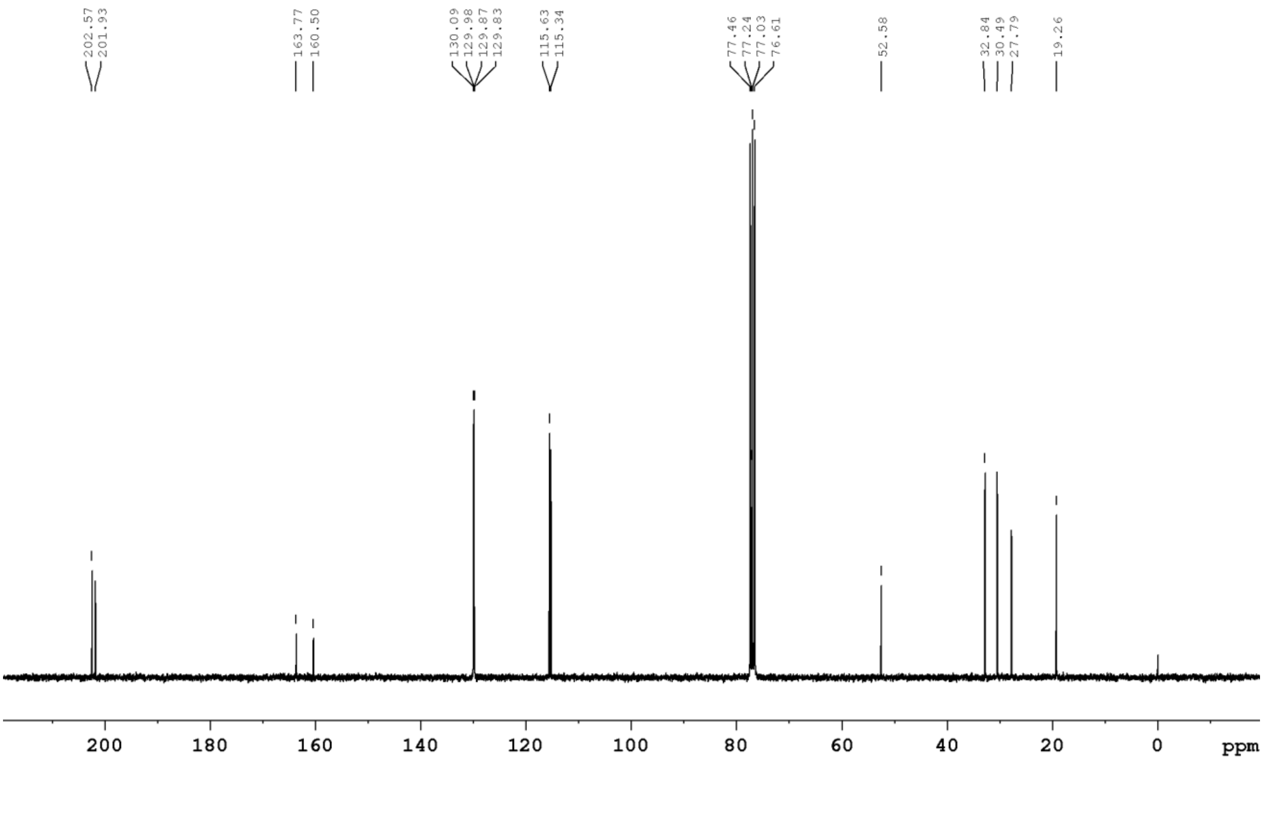
202.57 201.93 163.77 160.50 130.09 129.98 129.87 129.83 115.63 115.34 77.46 77.24 77.03 76.61 52.58 32.84 30.49 27.79 19.26
200 180 160 140 120 100 80 60 40 20 0 ppm

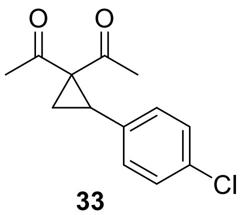

33

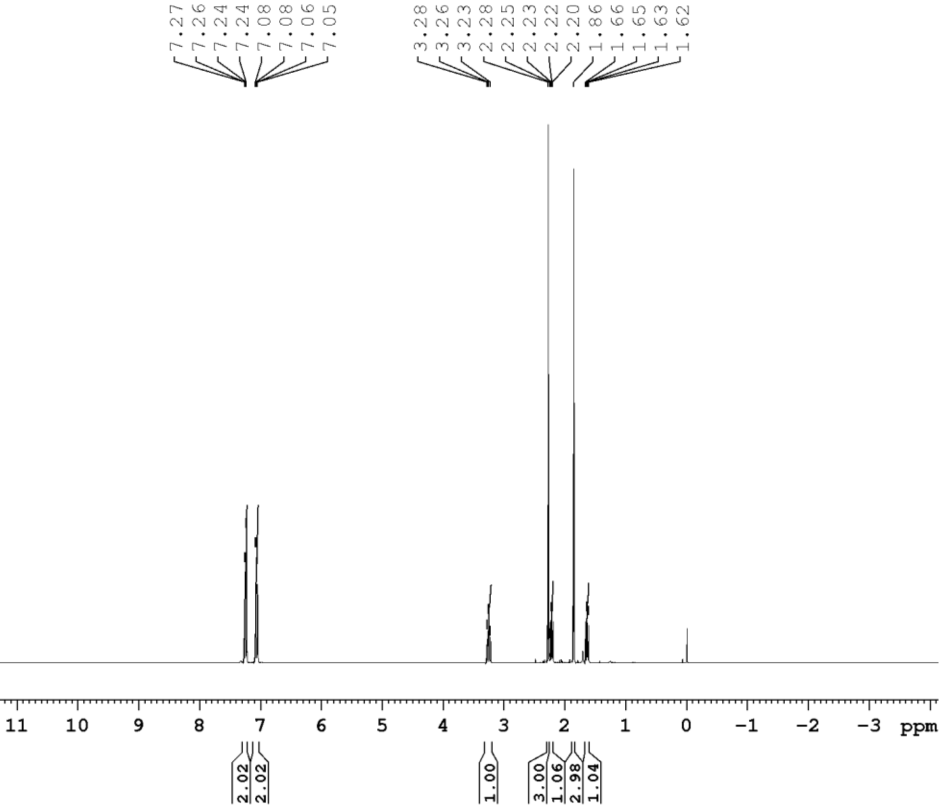

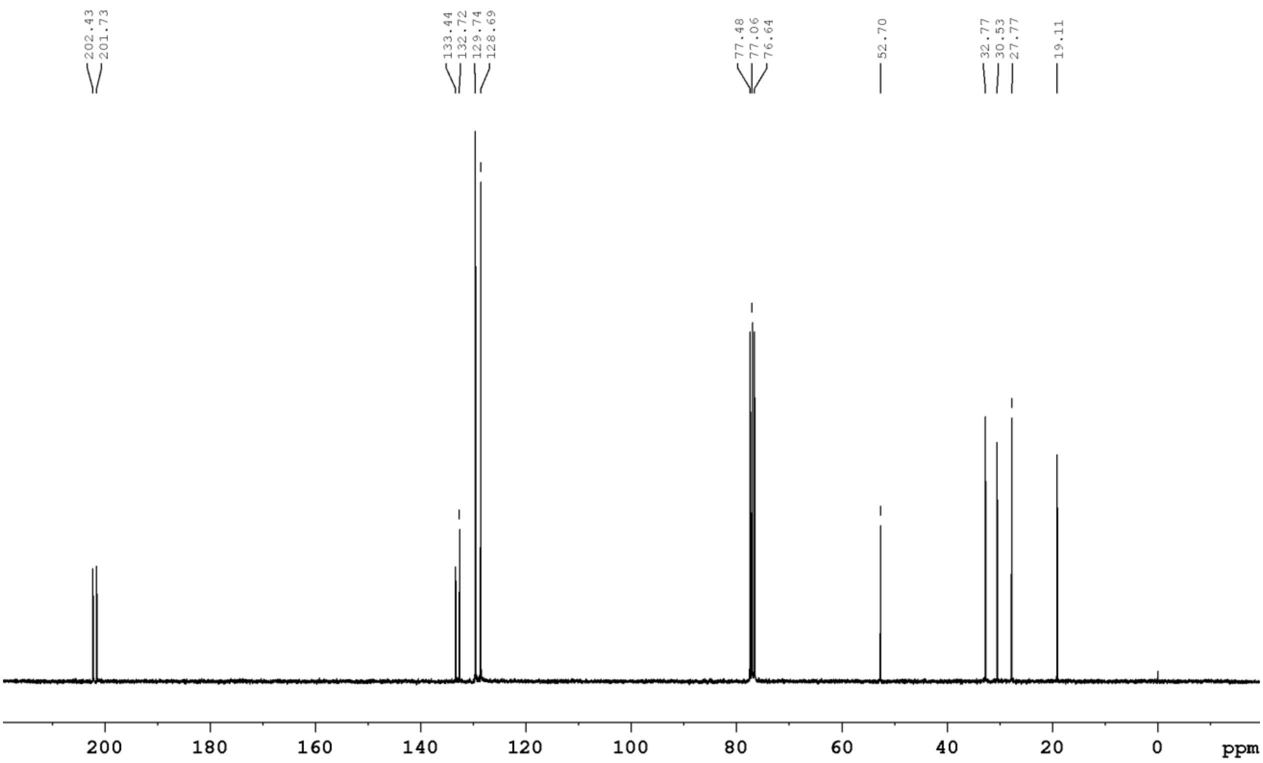

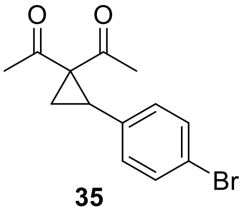
Br

35

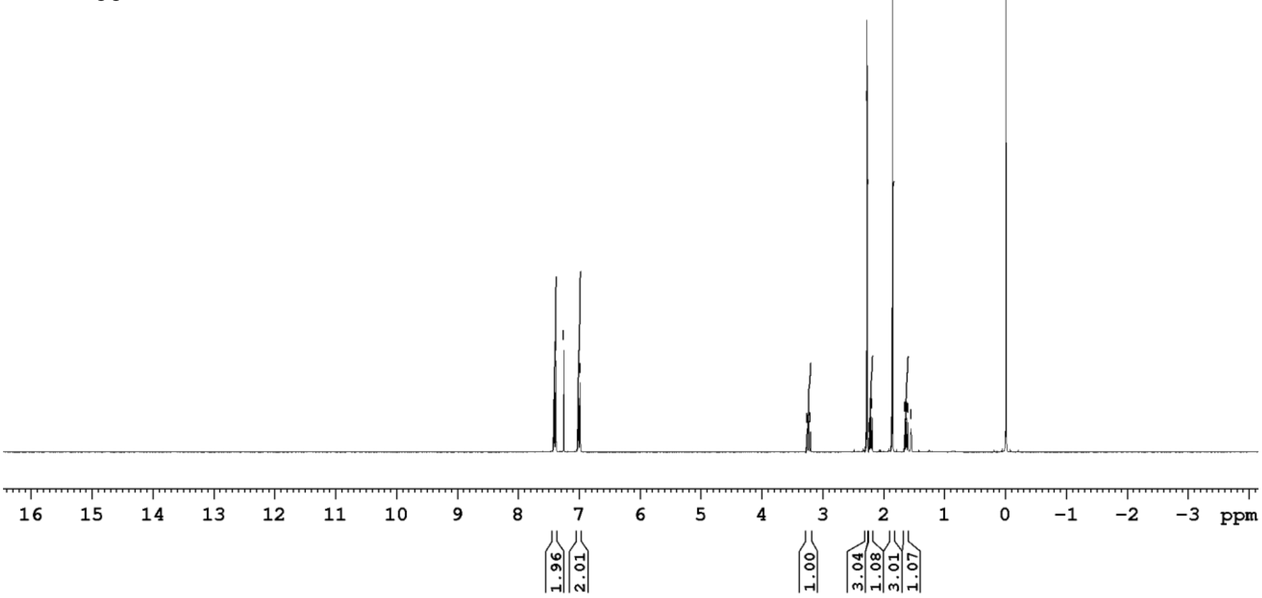
7.42
7.41
7.40
7.39
7.26
7.02
7.00
6.99
3.27
3.24
3.21
2.28
2.24
2.22
2.21
2.20
1.86
1.66
1.64
1.63
1.61
1.56
1.96
2.01
1.00
3.04
1.08
3.01
1.07
ppm

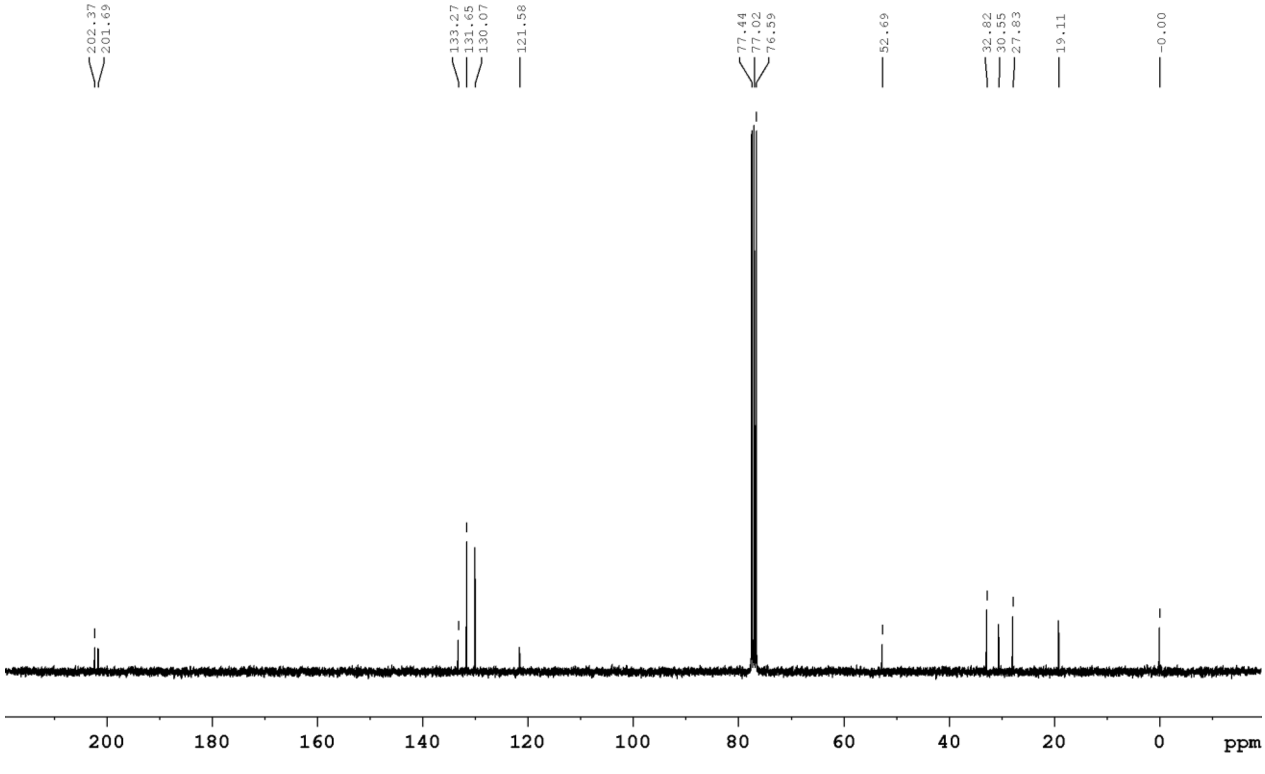
202.37
201.69
133.27
131.65
130.07
121.58
77.44
77.02
76.59
52.69
32.82
30.55
27.83
19.11
-0.00
ppm

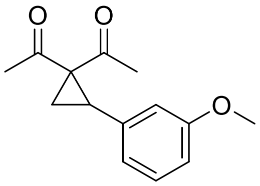

37

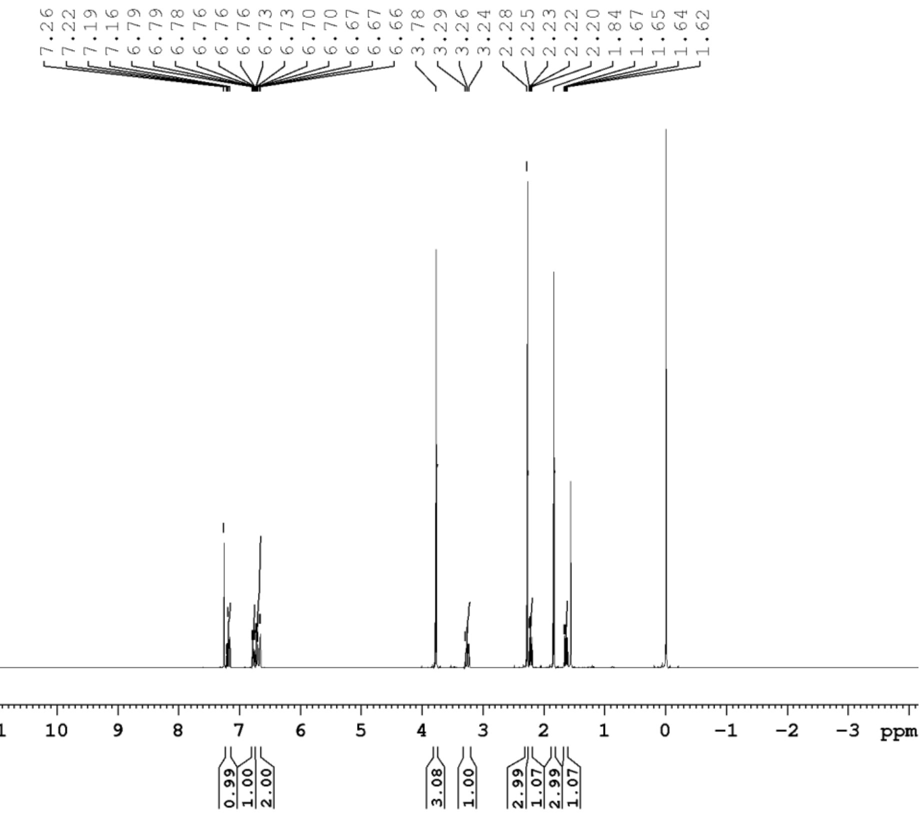

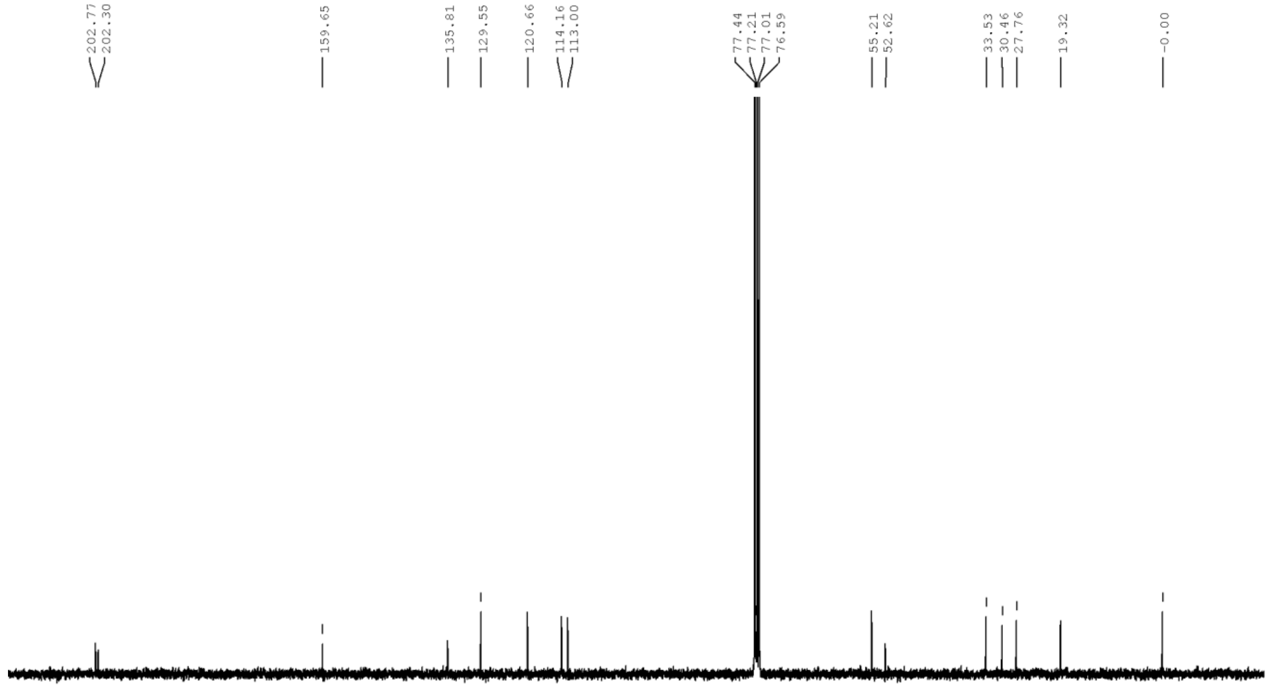

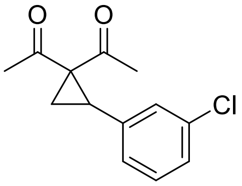
O
O
Cl

39

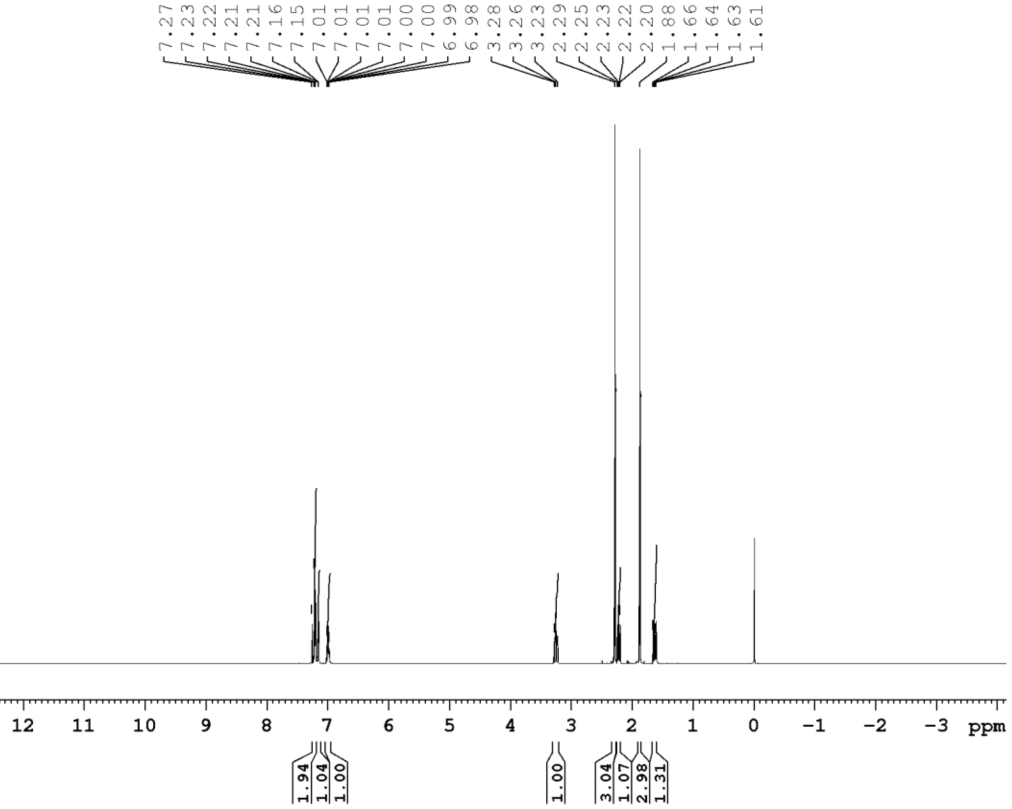
7.27 7.23 7.22 7.21 7.21 7.16 7.15 7.01 7.01 7.01 7.01 7.00 7.00 6.99 6.98 3.28 3.26 3.23 2.29 2.25 2.23 2.22 2.20 1.88 1.66 1.64 1.63 1.61
16 15 14 13 12 11 10 9 8 7 6 5 4 3 2 1 0 −1 −2 −3 ppm
1.94 1.04 1.00 1.00 3.04 1.07 2.98 1.31

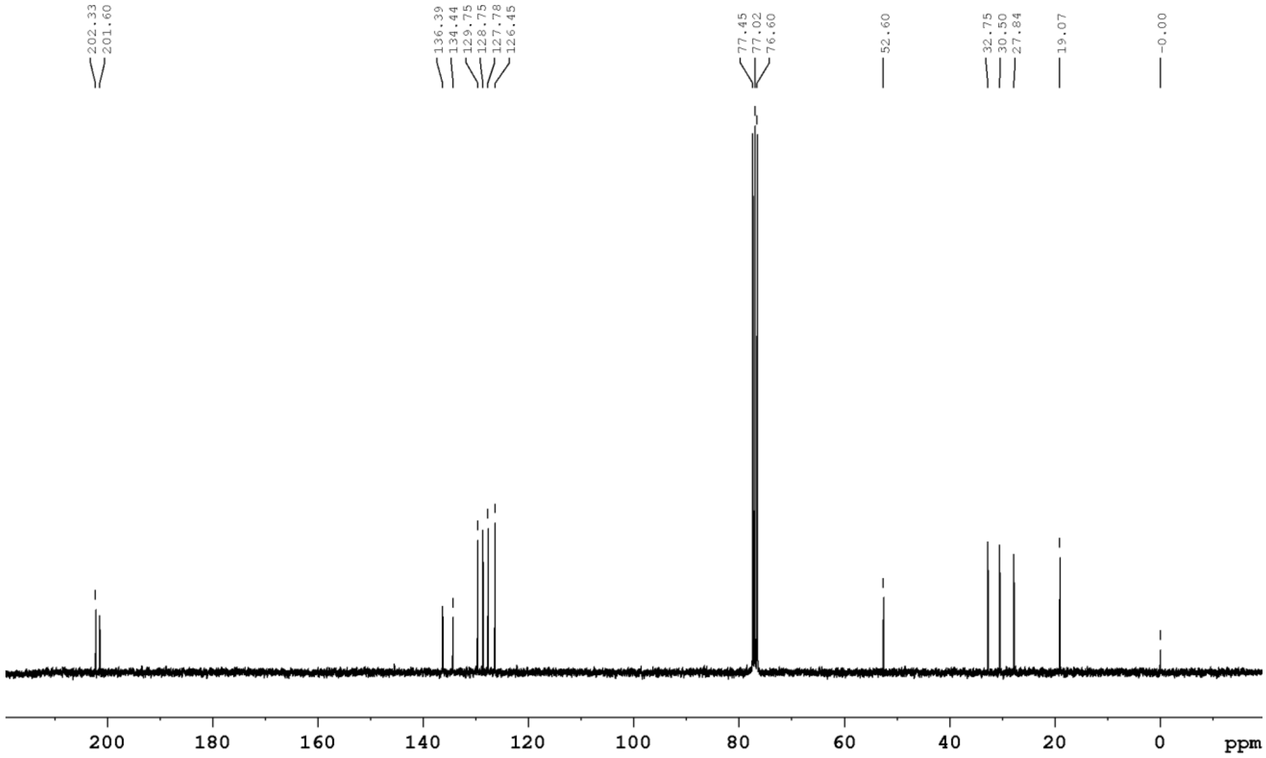
202.33 201.60 136.39 134.44 129.75 128.75 127.78 126.45 77.45 77.02 76.60 52.60 32.75 30.50 27.84 19.07 -0.00
200 180 160 140 120 100 80 60 40 20 0 ppm

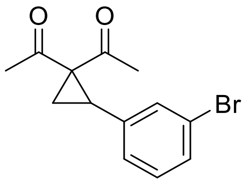

41

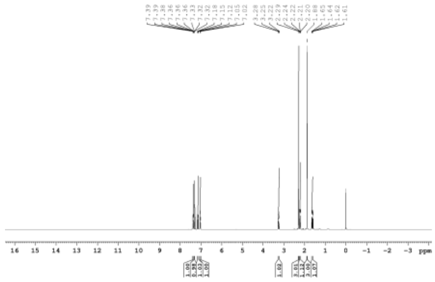

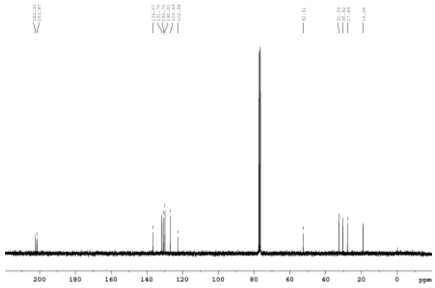

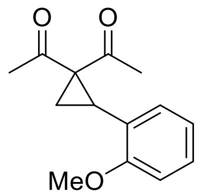

43

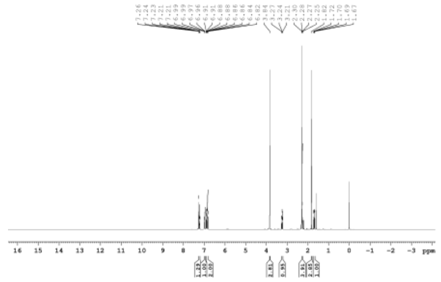

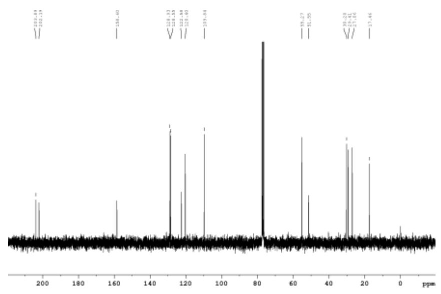

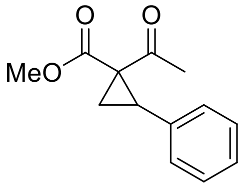

45

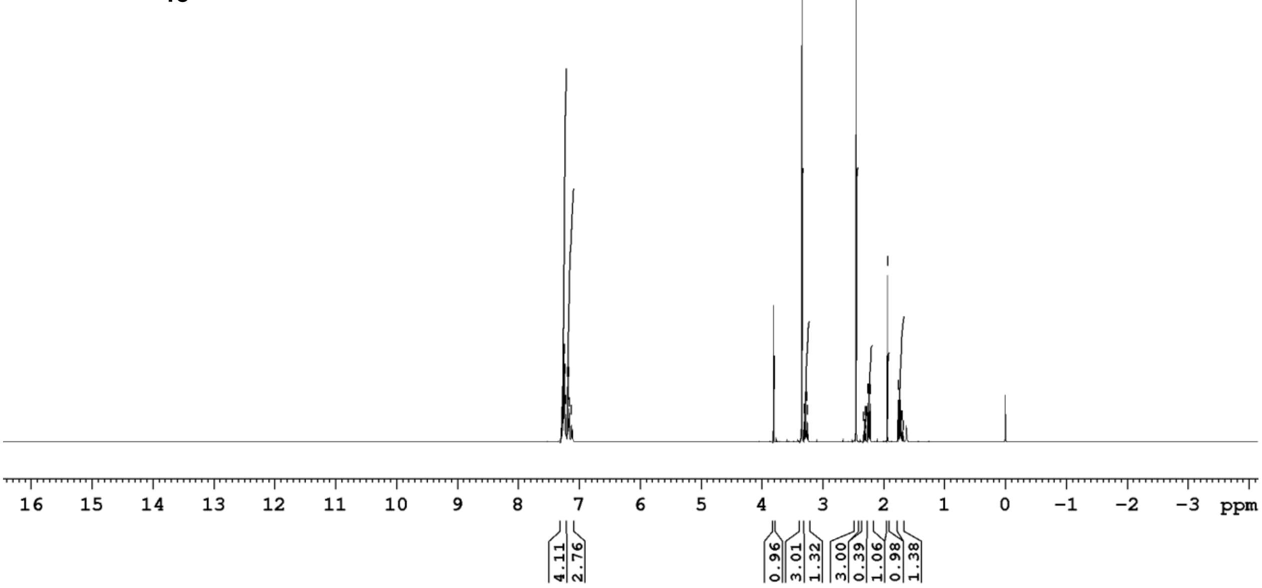

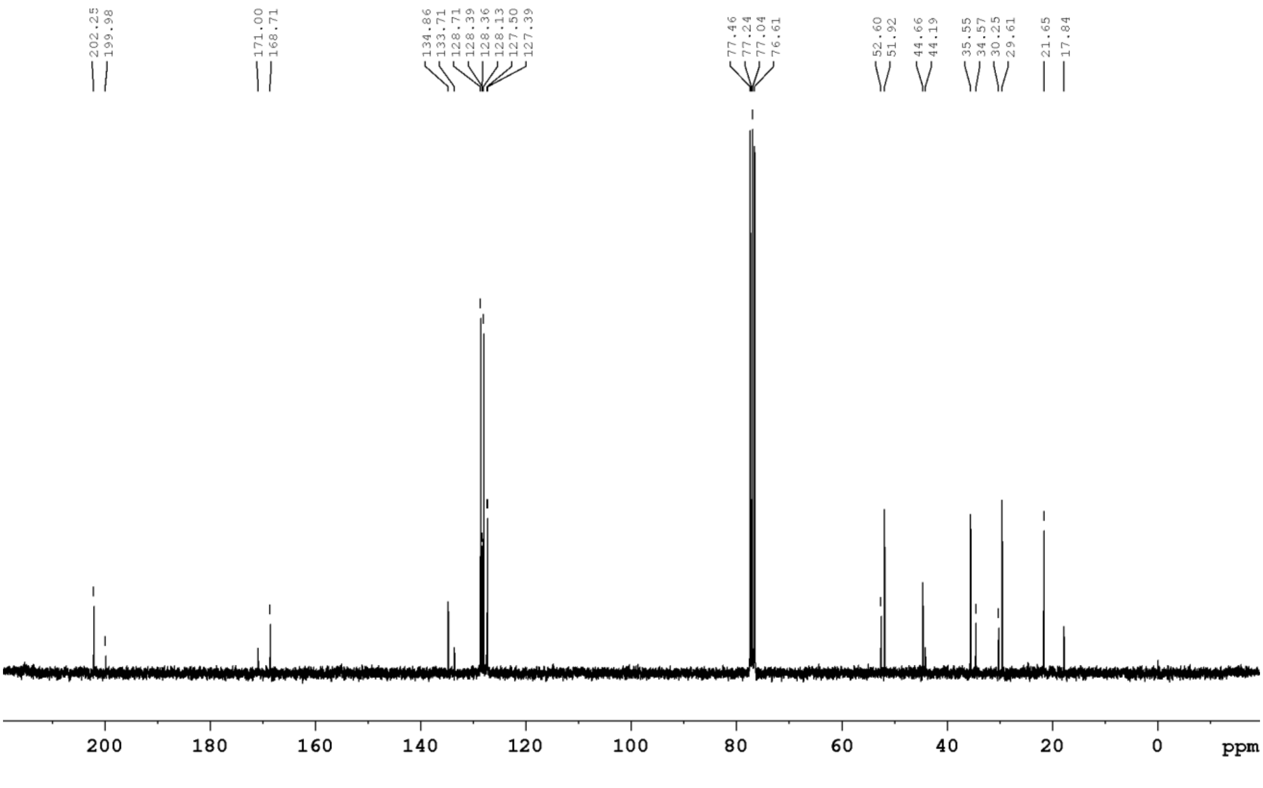

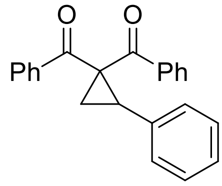

47

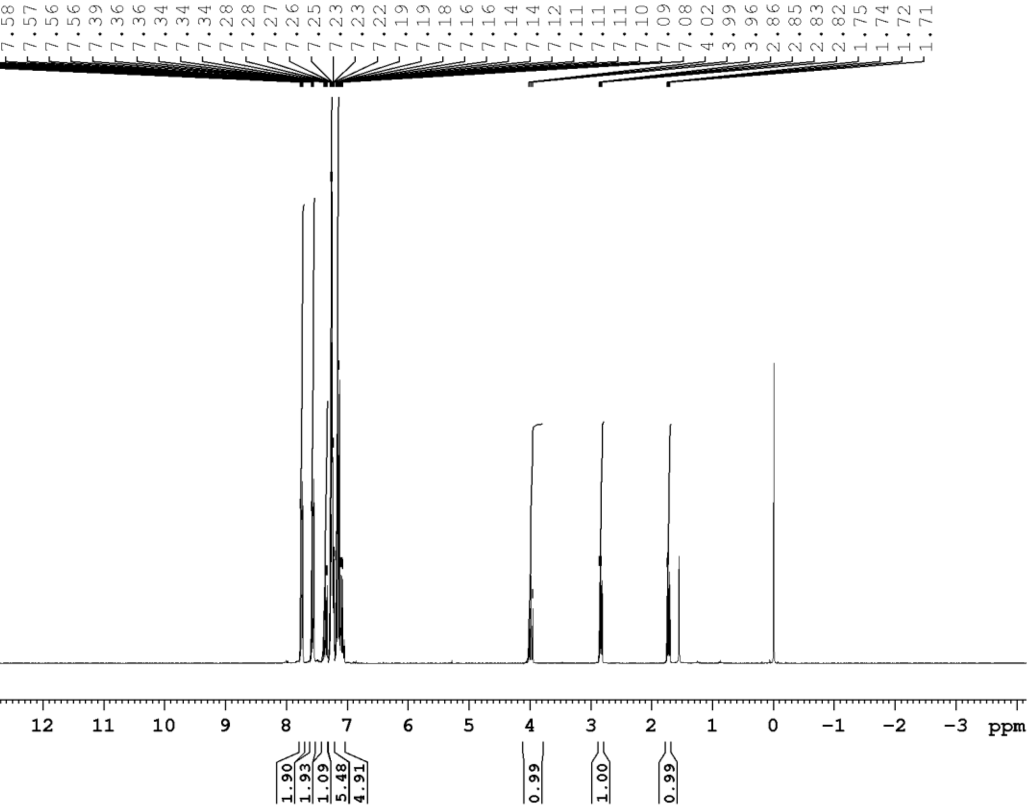
7.77 7.77 7.75 7.74 7.74 7.59 7.58 7.58 7.57 7.56 7.56 7.39 7.36 7.36 7.34 7.34 7.34 7.28 7.28 7.27 7.26 7.25 7.23 7.23 7.22 7.19 7.19 7.18 7.16 7.16 7.14 7.14 7.12 7.11 7.11 7.11 7.10 7.09 7.08 4.02 3.99 3.96 2.86 2.85 2.83 2.82 1.75 1.74 1.72 1.71
16 15 14 13 12 11 10 9 8 7 6 5 4 3 2 1 0 −1 −2 −3 ppm
1.90 1.93 1.09 5.48 4.91
0.99
1.00
0.99

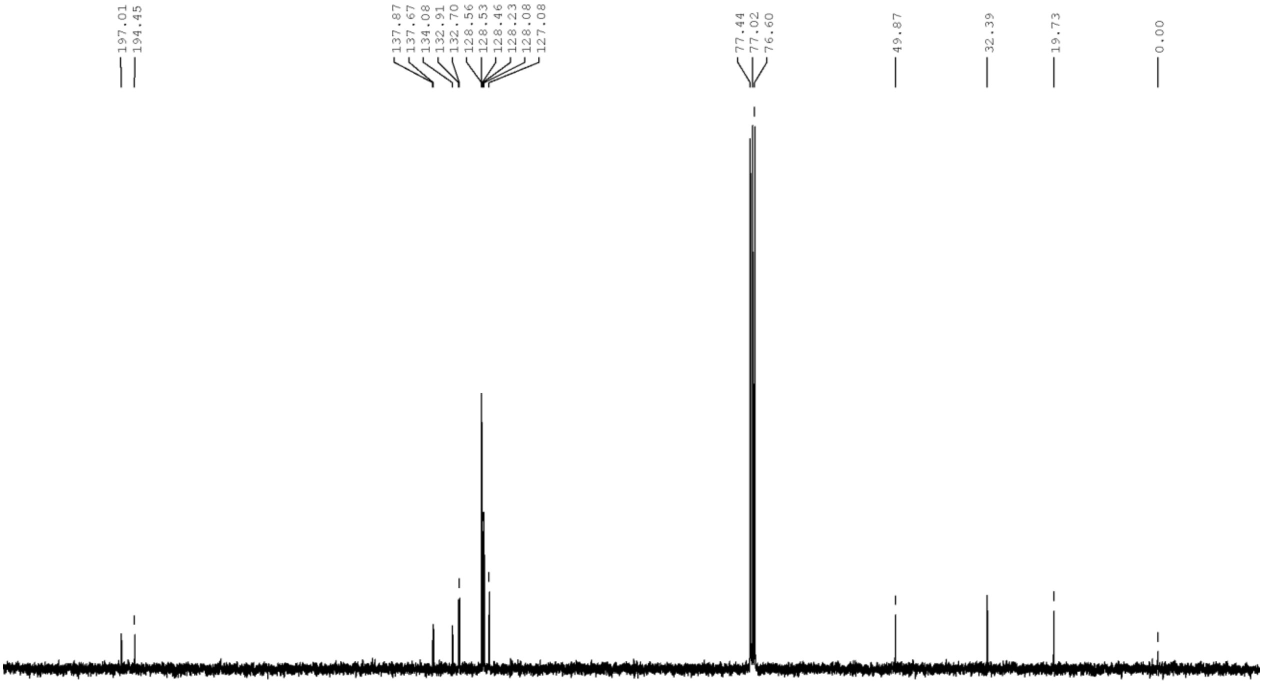
197.01 194.45
137.87 137.67 134.08 132.91 132.70 128.56 128.53 128.46 128.23 128.08 127.08
77.44 77.02 76.60
49.87
32.39
19.73
0.00
200 180 160 140 120 100 80 60 40 20 0 ppm

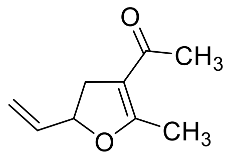
O
CH3
O
CH3

2

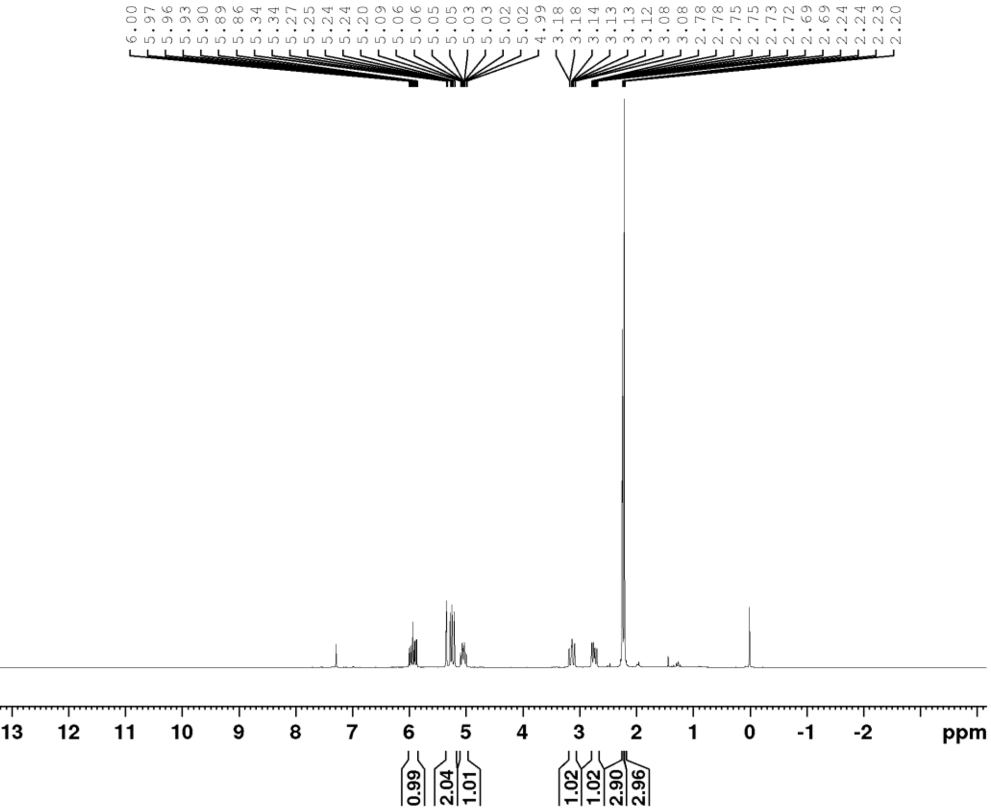
6.00
5.97
5.96
5.93
5.90
5.89
5.86
5.34
5.34
5.27
5.25
5.24
5.24
5.20
5.09
5.06
5.05
5.05
5.03
5.03
5.02
5.02
4.99
3.18
3.18
3.14
3.13
3.13
3.12
3.08
3.08
2.78
2.78
2.75
2.73
2.72
2.69
2.69
2.24
2.24
2.23
2.20
0.99
2.04
1.01
1.02
1.02
2.90
2.96
16
15
14
13
12
11
10
9
8
7
6
5
4
3
2
1
0
-1
-2
ppm

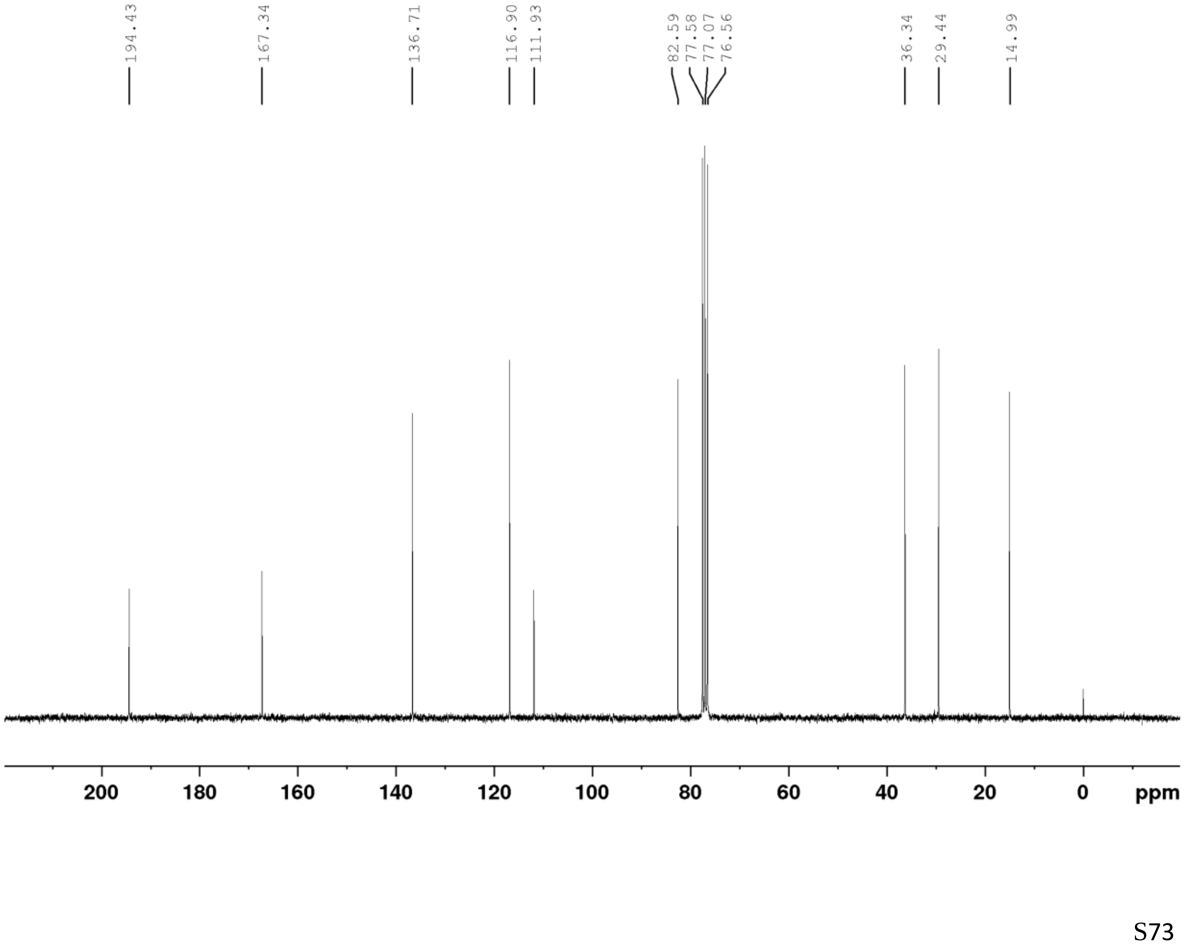
194.43
167.34
136.71
116.90
111.93
82.59
77.58
77.07
76.56
36.34
29.44
14.99
200
180
160
140
120
100
80
60
40
20
0
ppm

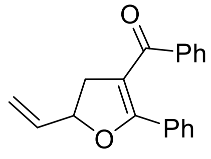
O
Ph
O
Ph

4

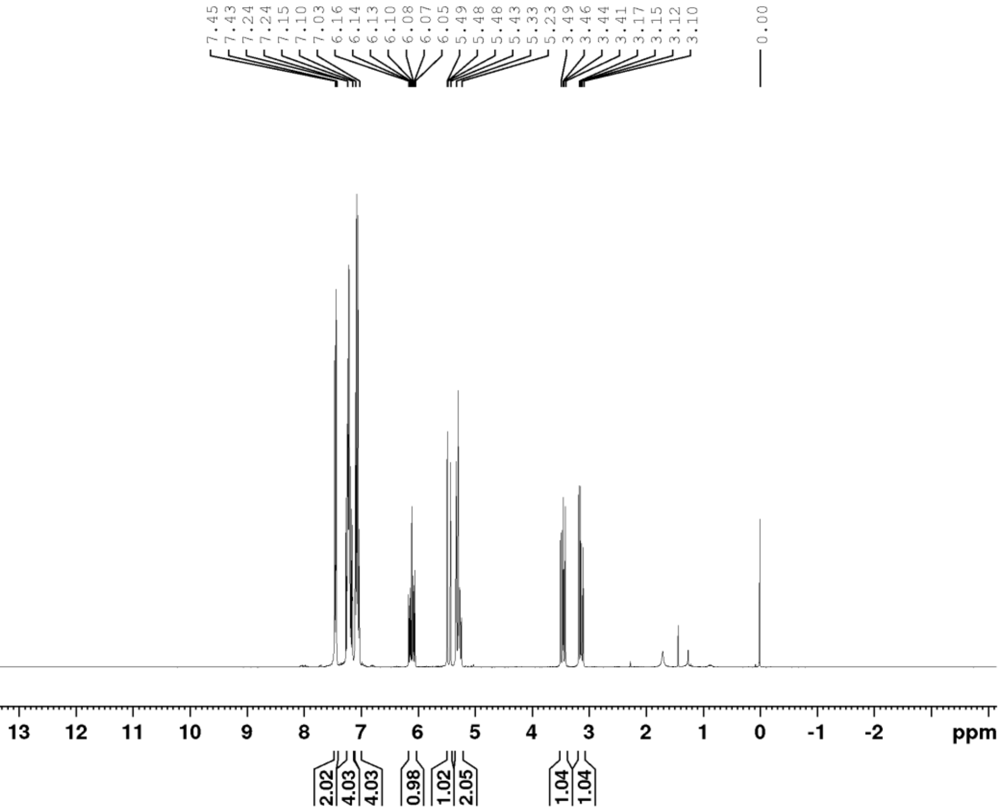
7.45 7.43 7.24 7.24 7.15 7.10 7.03 6.16 6.14 6.13 6.10 6.08 6.07 6.05 5.49 5.48 5.48 5.43 5.33 5.23 3.49 3.46 3.44 3.41 3.17 3.15 3.12 3.10 0.00
16 15 14 13 12 11 10 9 8 7 6 5 4 3 2 1 0 -1 -2 ppm
2.02 4.03 4.03 0.98 1.02 2.05 1.04 1.04

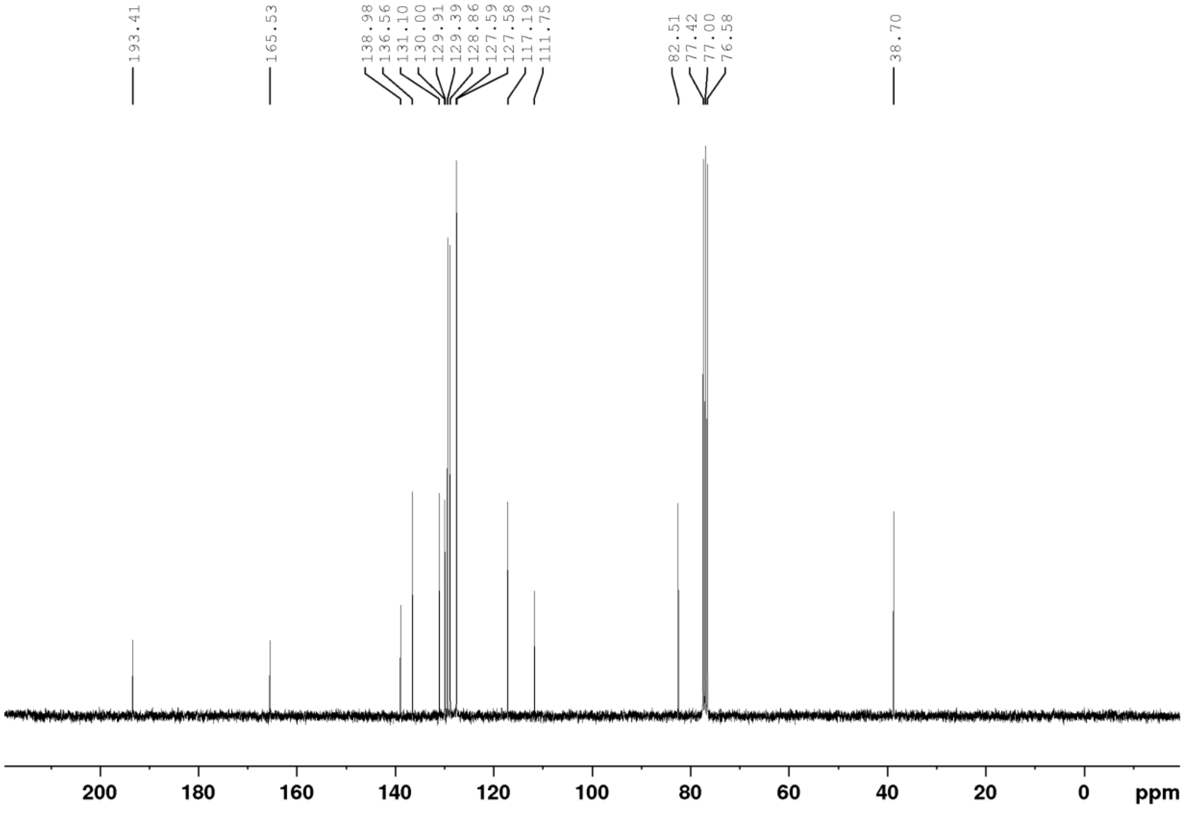
193.41 165.53 138.98 136.56 131.10 130.00 129.91 129.39 128.86 127.59 127.58 117.19 111.75 82.51 77.42 77.00 76.58 38.70
200 180 160 140 120 100 80 60 40 20 0 ppm

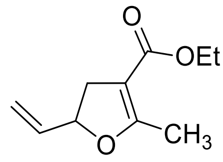
O
OEt
O
CH3

6

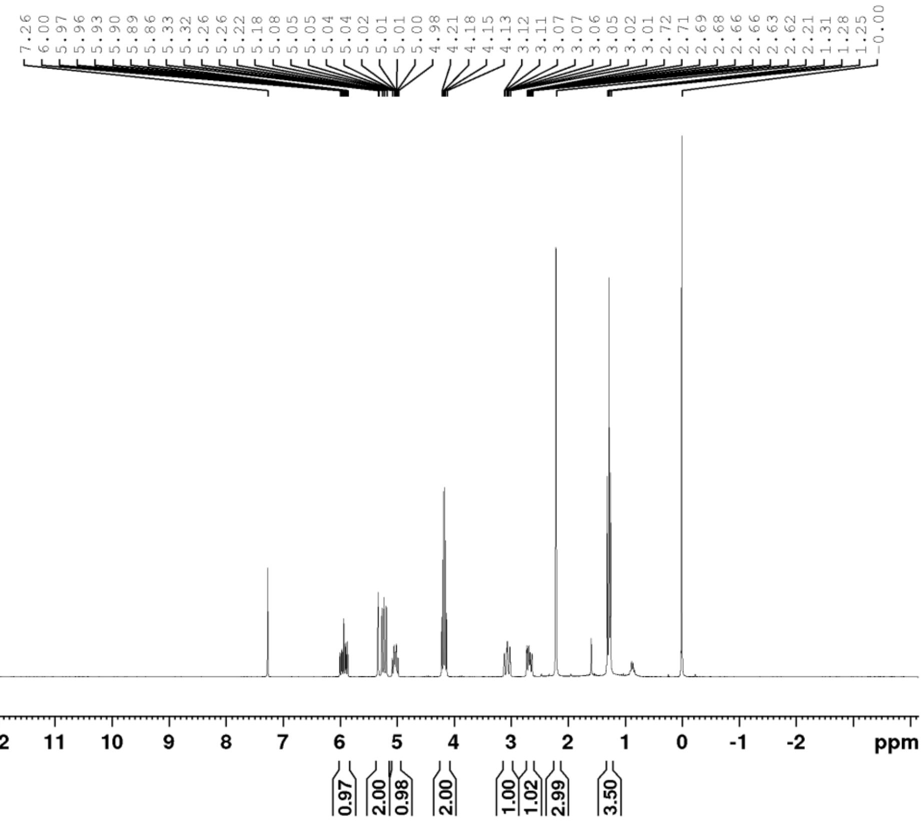
7.26
6.00
5.97
5.96
5.93
5.90
5.89
5.86
5.33
5.32
5.26
5.22
5.18
5.08
5.05
5.05
5.04
5.04
5.02
5.01
5.01
5.00
4.98
4.21
4.18
4.15
4.13
3.12
3.11
3.07
3.07
3.06
3.05
3.02
3.01
2.72
2.71
2.69
2.68
2.66
2.66
2.63
2.62
2.21
1.31
1.28
1.25
-0.00
16 15 14 13 12 11 10 9 8 7 6 5 4 3 2 1 0 -1 -2 ppm
0.97
2.00
0.98
2.00
1.00
1.02
2.99
3.50

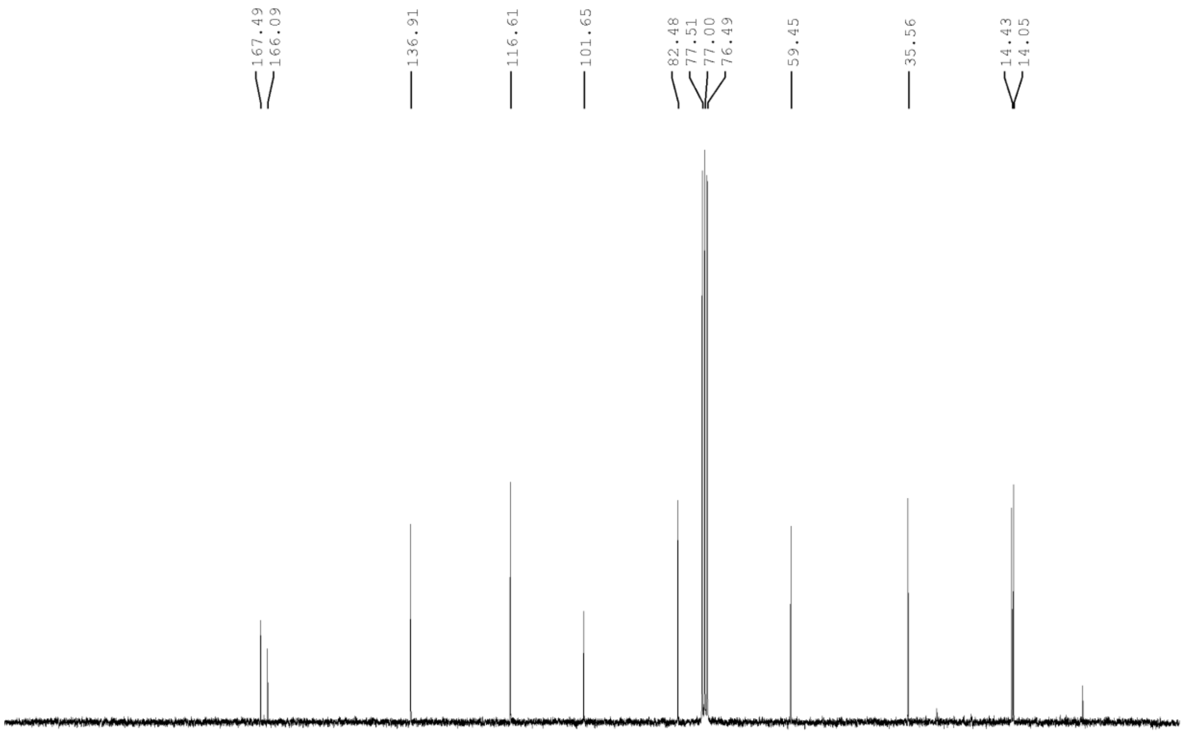
167.49
166.09
136.91
116.61
101.65
82.48
77.51
77.00
76.49
59.45
35.56
14.43
14.05
200 180 160 140 120 100 80 60 40 20 0 ppm

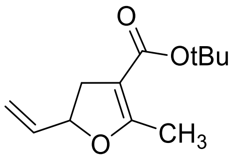
O
OtBu
O
CH3

8

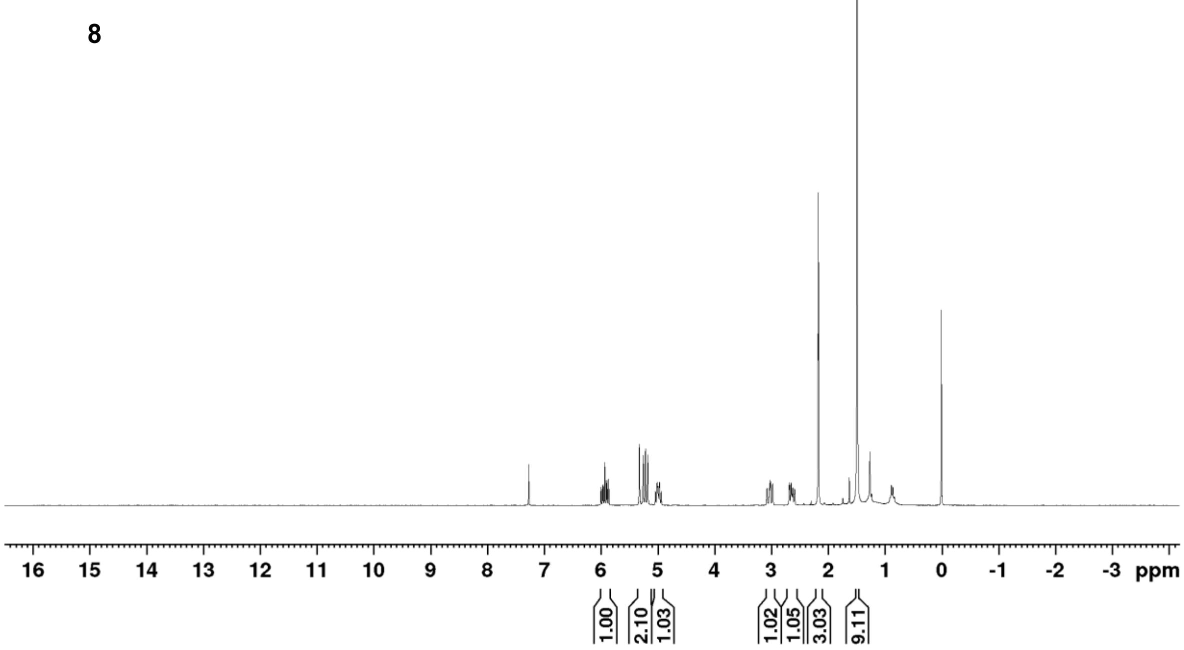
7.27
5.99
5.97
5.95
5.93
5.90
5.88
5.86
5.32
5.32
5.31
5.25
5.25
5.24
5.22
5.21
5.21
5.17
5.17
5.17
5.03
5.01
5.00
5.00
4.99
4.98
4.97
4.97
4.96
4.93
3.07
3.07
3.03
3.02
3.02
3.01
2.97
2.97
2.68
2.67
2.65
2.64
2.62
2.61
2.59
2.58
2.17
1.48
-0.00
16 15 14 13 12 11 10 9 8 7 6 5 4 3 2 1 0 -1 -2 -3 ppm
1.00
2.10
1.03
1.02
1.05
3.03
9.11

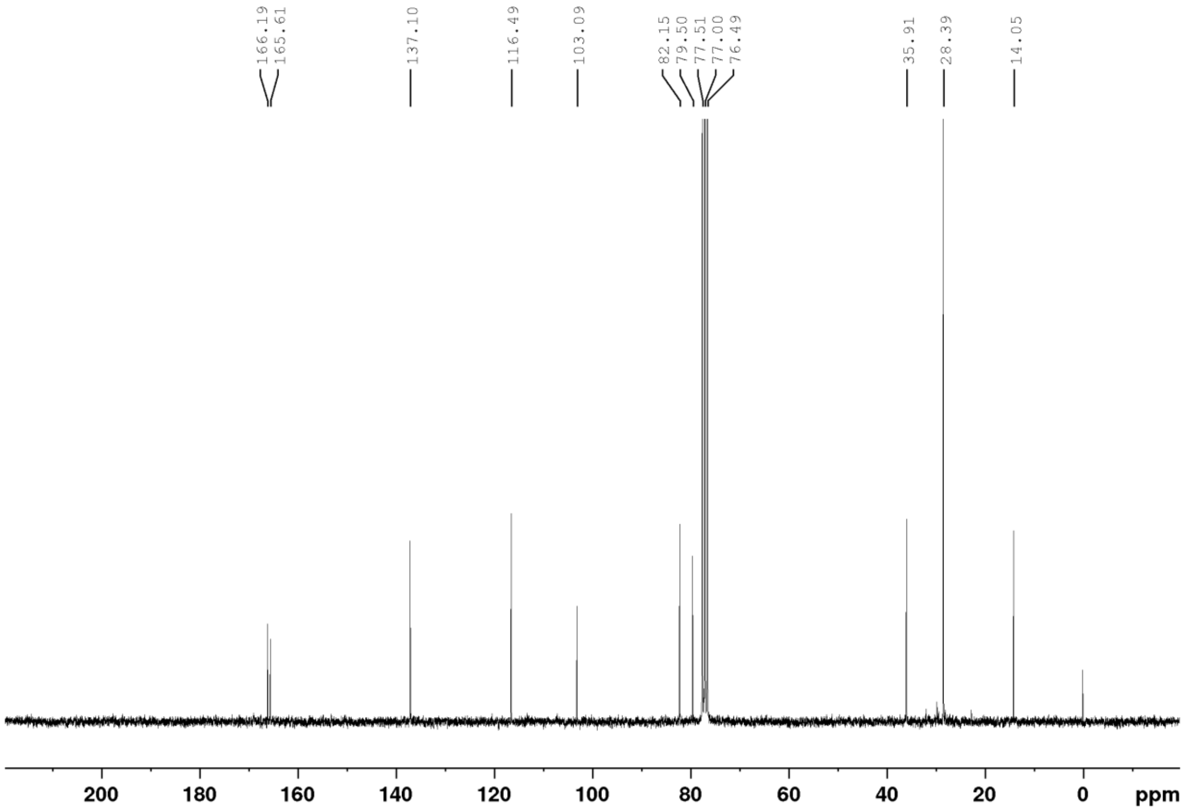
166.19
165.61
137.10
116.49
103.09
82.15
79.50
77.51
77.00
76.49
35.91
28.39
14.05
200 180 160 140 120 100 80 60 40 20 0 ppm

10

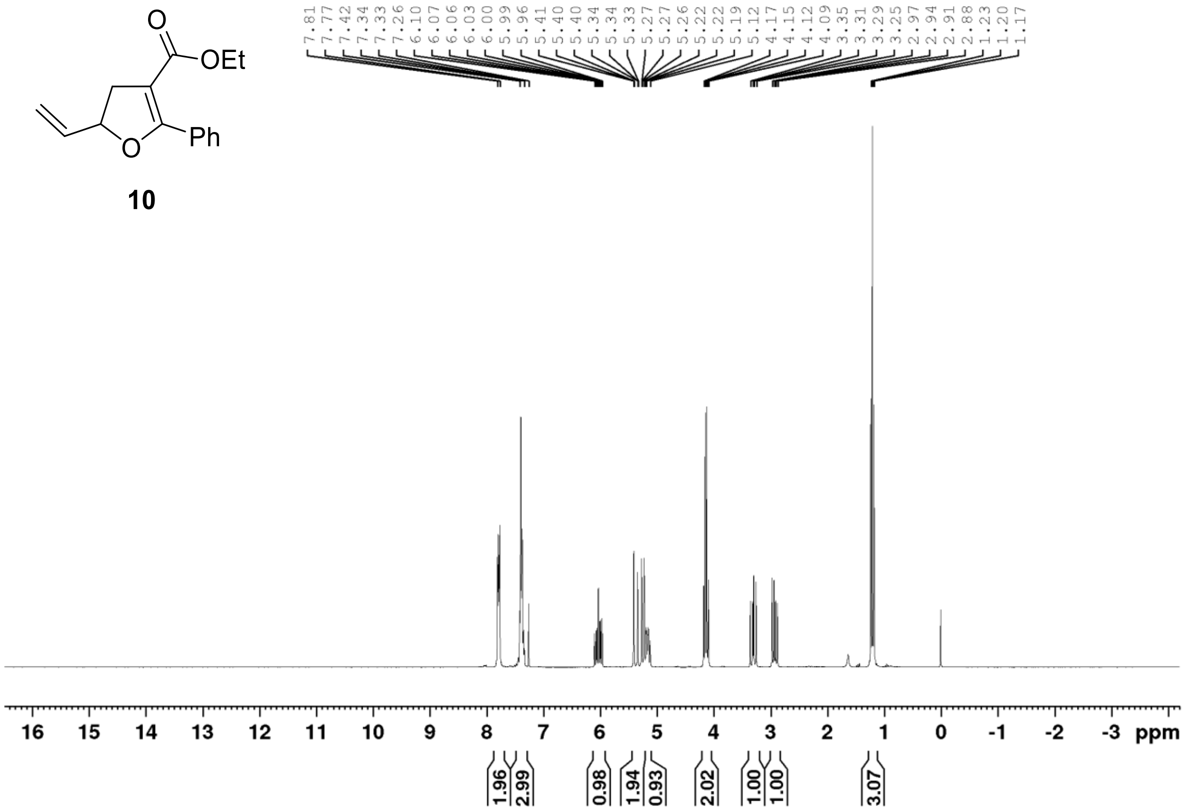
O
OEt
Ph
O
7.81 7.77 7.42 7.34 7.33 7.26 6.10 6.07 6.06 6.03 6.00 5.99 5.96 5.41 5.40 5.40 5.34 5.34 5.33 5.27 5.27 5.26 5.22 5.22 5.19 5.12 4.17 4.15 4.12 4.09 3.35 3.31 3.29 3.25 2.97 2.94 2.91 2.88 1.23 1.20 1.17
16 15 14 13 12 11 10 9 8 7 6 5 4 3 2 1 0 -1 -2 -3 ppm
1.96 2.99 0.98 1.94 0.93 2.02 1.00 1.00 3.07

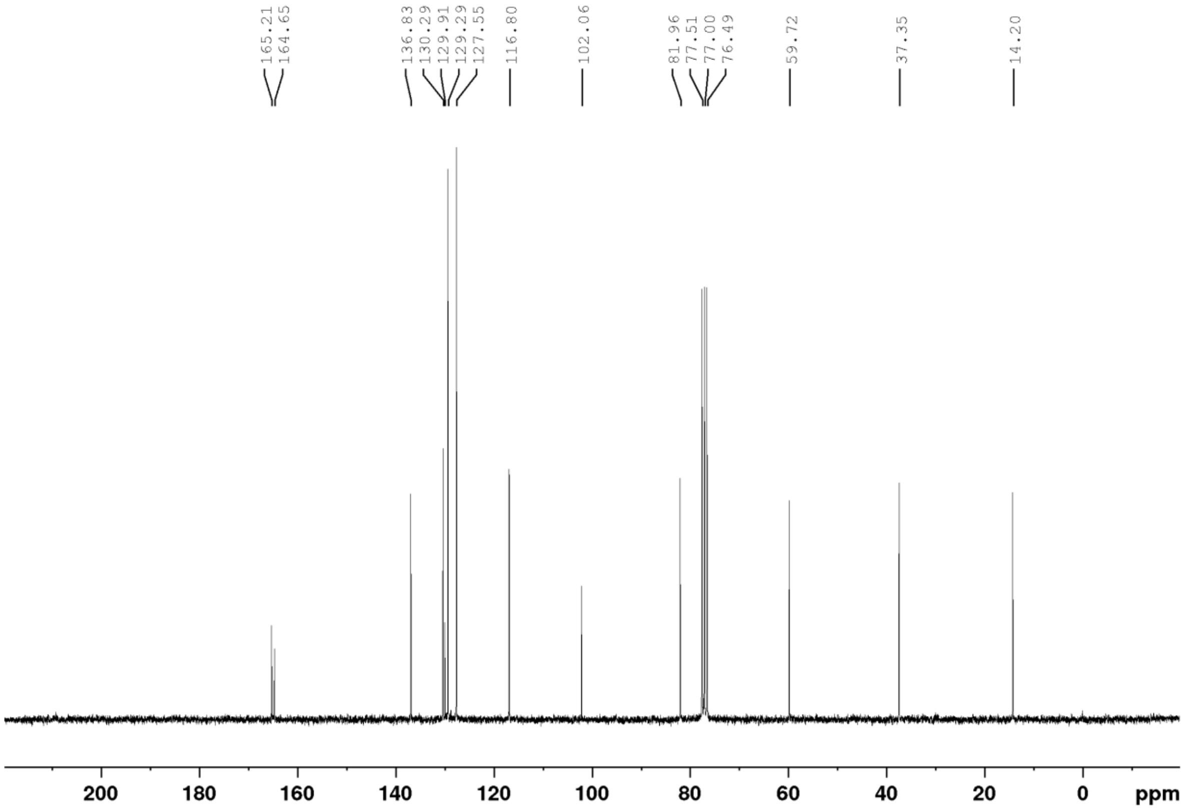
165.21 164.65 136.83 130.29 129.91 129.29 127.55 116.80 102.06 81.96 77.51 77.00 76.49 59.72 37.35 14.20
200 180 160 140 120 100 80 60 40 20 0 ppm

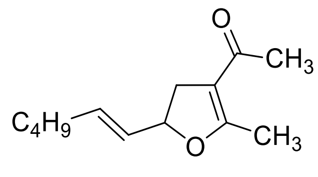

O
CH3
C4H9
O
CH3

12

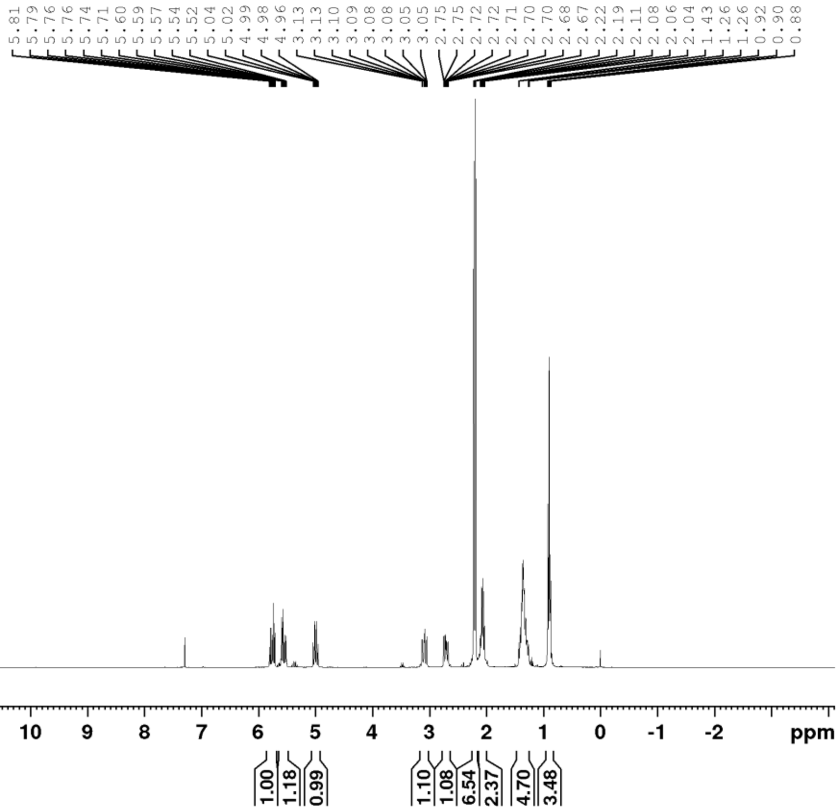

5.81 5.79 5.76 5.76 5.74 5.71 5.60 5.59 5.57 5.54 5.52 5.04 5.02 4.99 4.98 4.96 3.13 3.13 3.10 3.09 3.08 3.05 3.05 2.75 2.75 2.72 2.72 2.71 2.70 2.70 2.68 2.67 2.22 2.19 2.11 2.08 2.06 2.04 1.43 1.26 0.92 0.90 0.88
16 15 14 13 12 11 10 9 8 7 6 5 4 3 2 1 0 -1 -2 ppm
1.00 1.18 0.99 1.10 1.08 6.54 2.37 4.70 3.48

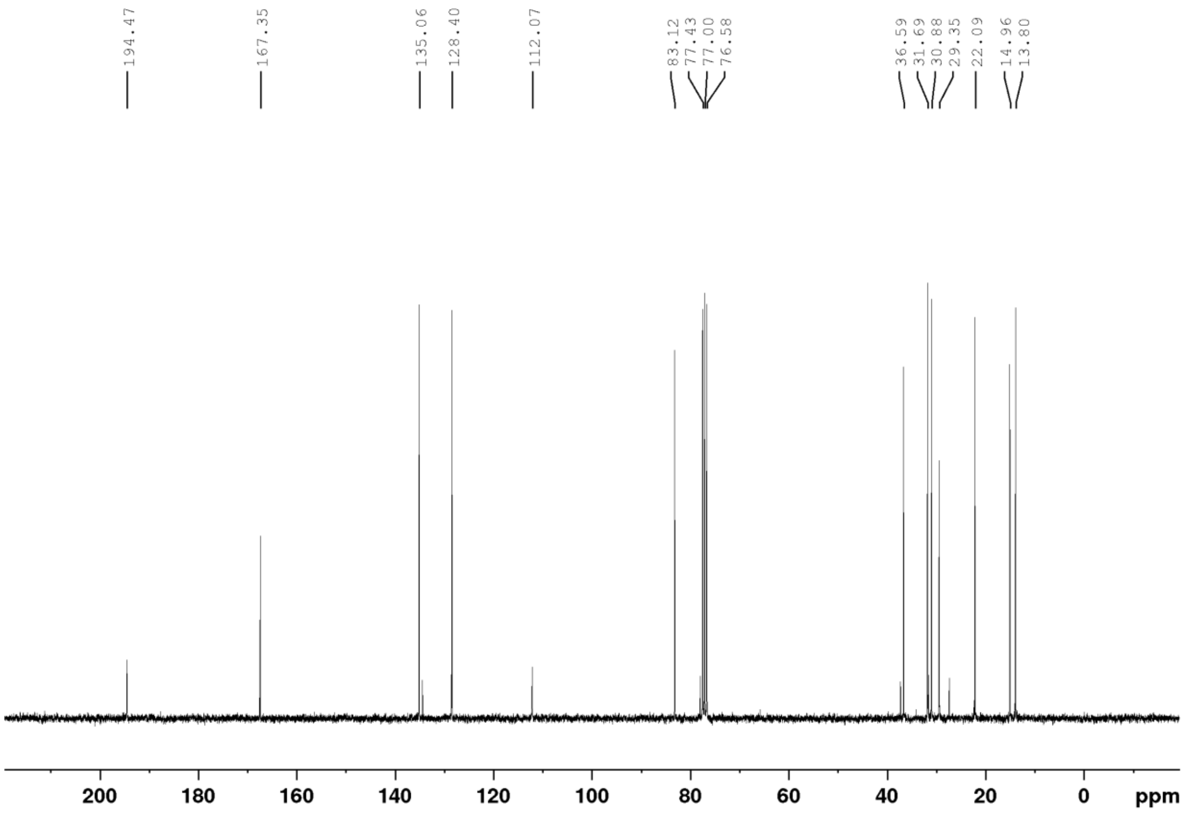

194.47 167.35 135.06 128.40 112.07 83.12 77.43 77.00 76.58 36.59 31.69 30.88 29.35 22.09 14.96 13.80
200 180 160 140 120 100 80 60 40 20 0 ppm

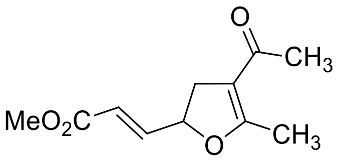

O
CH3
MeO2C
CH3
O

14

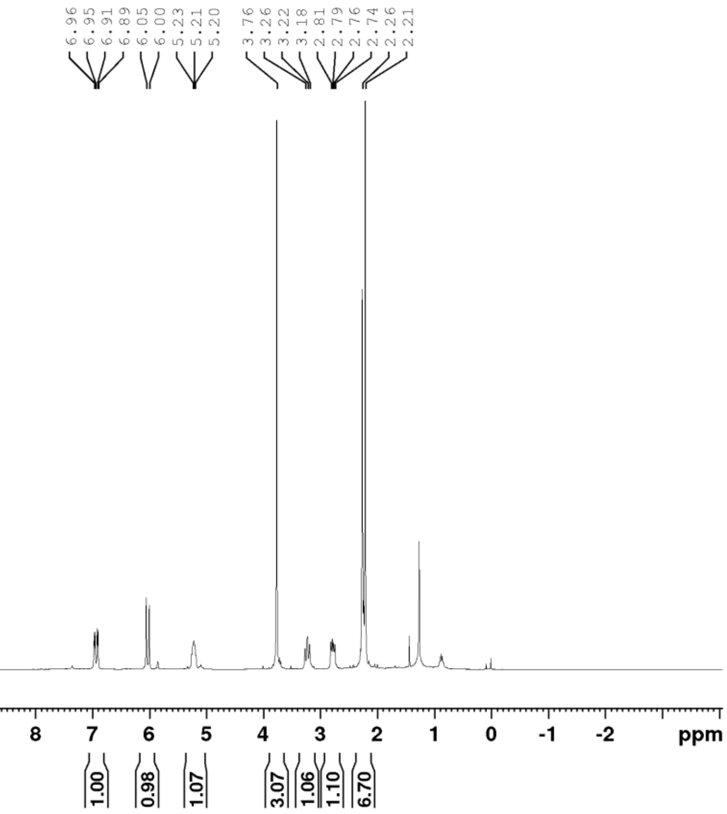

6.96
6.95
6.91
6.89
6.05
6.00
5.23
5.21
5.20
3.76
3.26
3.22
3.18
2.81
2.79
2.76
2.74
2.26
2.21
16 15 14 13 12 11 10 9 8 7 6 5 4 3 2 1 0 -1 -2 ppm
1.00
0.98
1.07
3.07
1.06
1.10
6.70

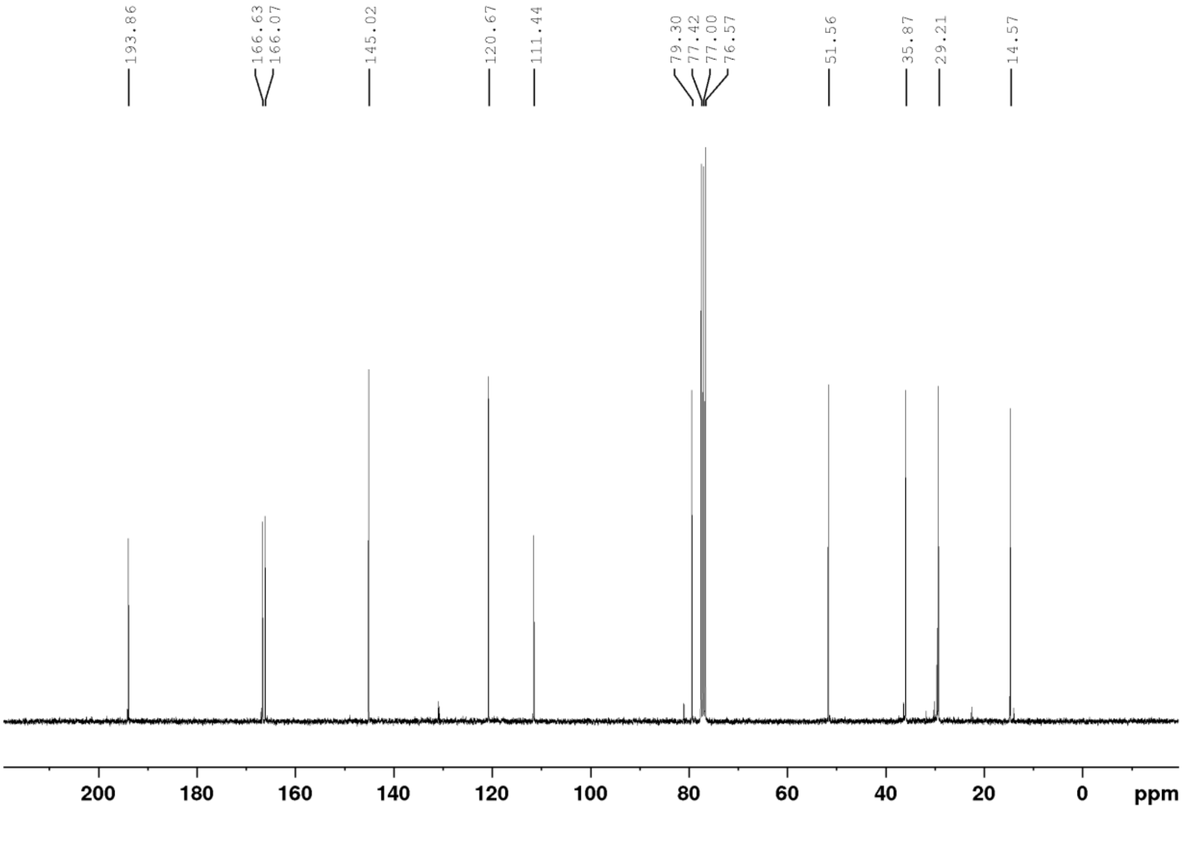

193.86
166.63
166.07
145.02
120.67
111.44
79.30
77.42
77.00
76.57
51.56
35.87
29.21
14.57
200 180 160 140 120 100 80 60 40 20 0 ppm

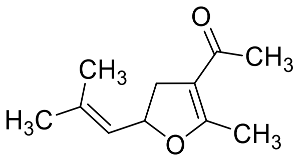

16

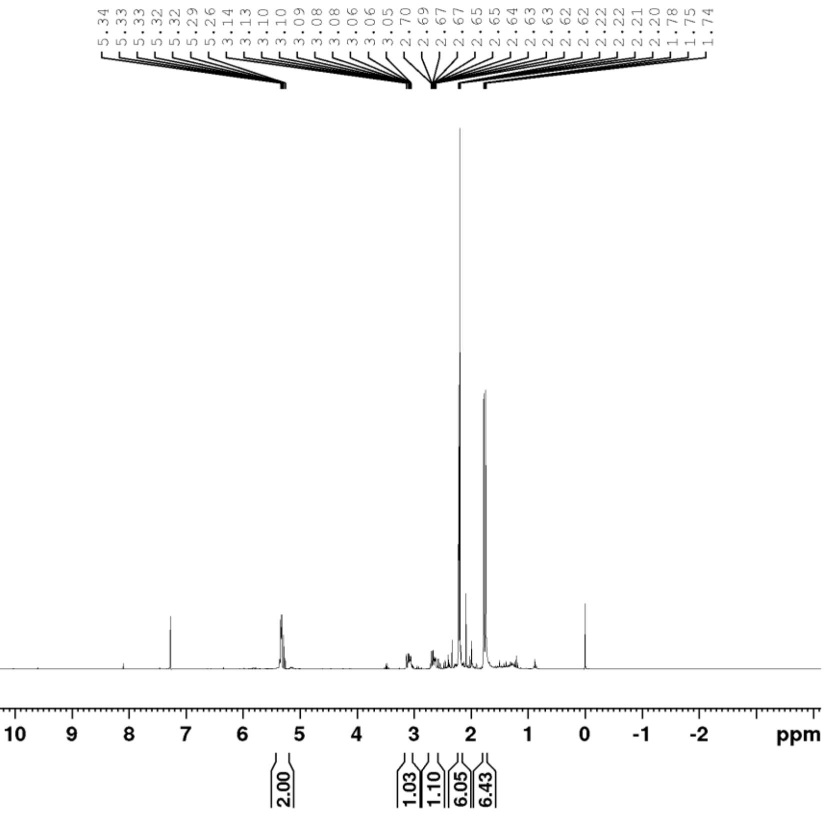
5.34 5.33 5.33 5.32 5.32 5.29 5.26 3.14 3.13 3.10 3.10 3.09 3.08 3.06 3.06 3.05 2.70 2.69 2.67 2.67 2.65 2.65 2.64 2.63 2.63 2.62 2.62 2.22 2.22 2.21 2.20 1.78 1.75 1.74
16 15 14 13 12 11 10 9 8 7 6 5 4 3 2 1 0 -1 -2 ppm
2.00
1.03
1.10
6.05
6.43

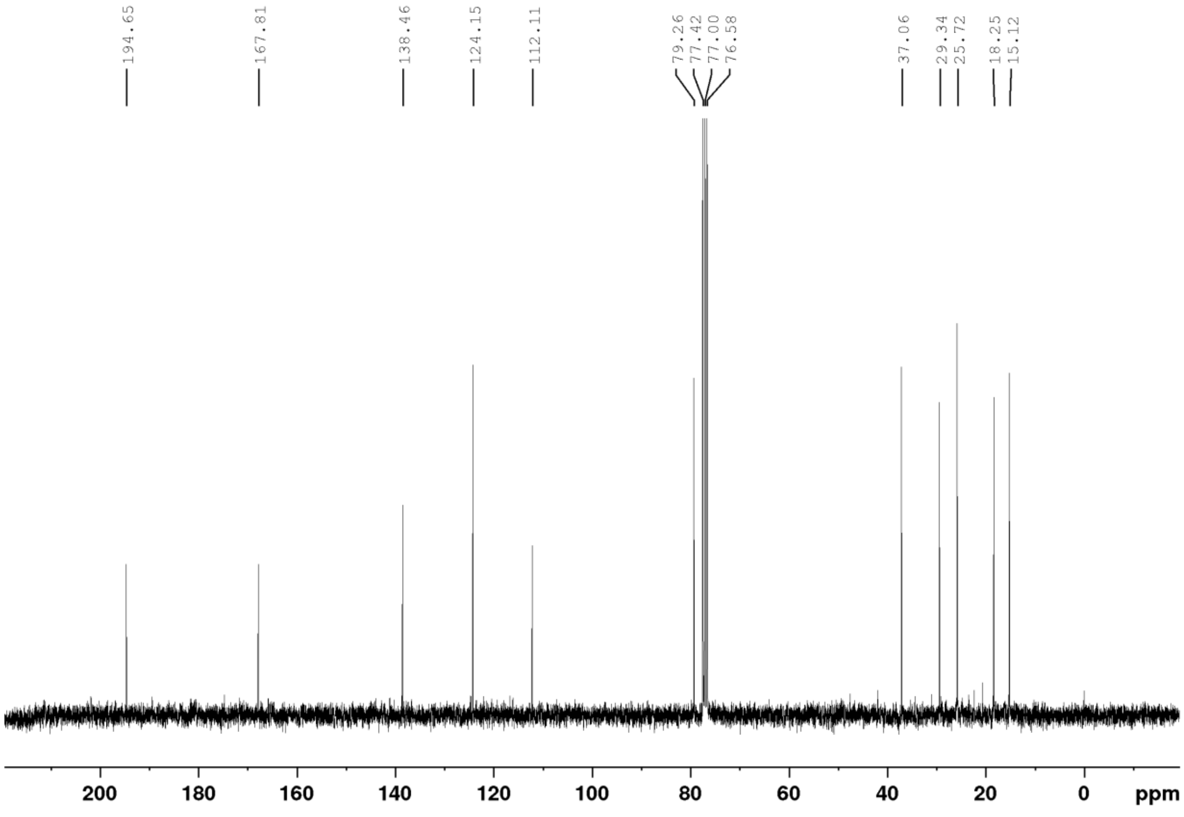
194.65
167.81
138.46
124.15
112.11
79.26 77.42 77.00 76.58
37.06
29.34
25.72
18.25
15.12
200 180 160 140 120 100 80 60 40 20 0 ppm

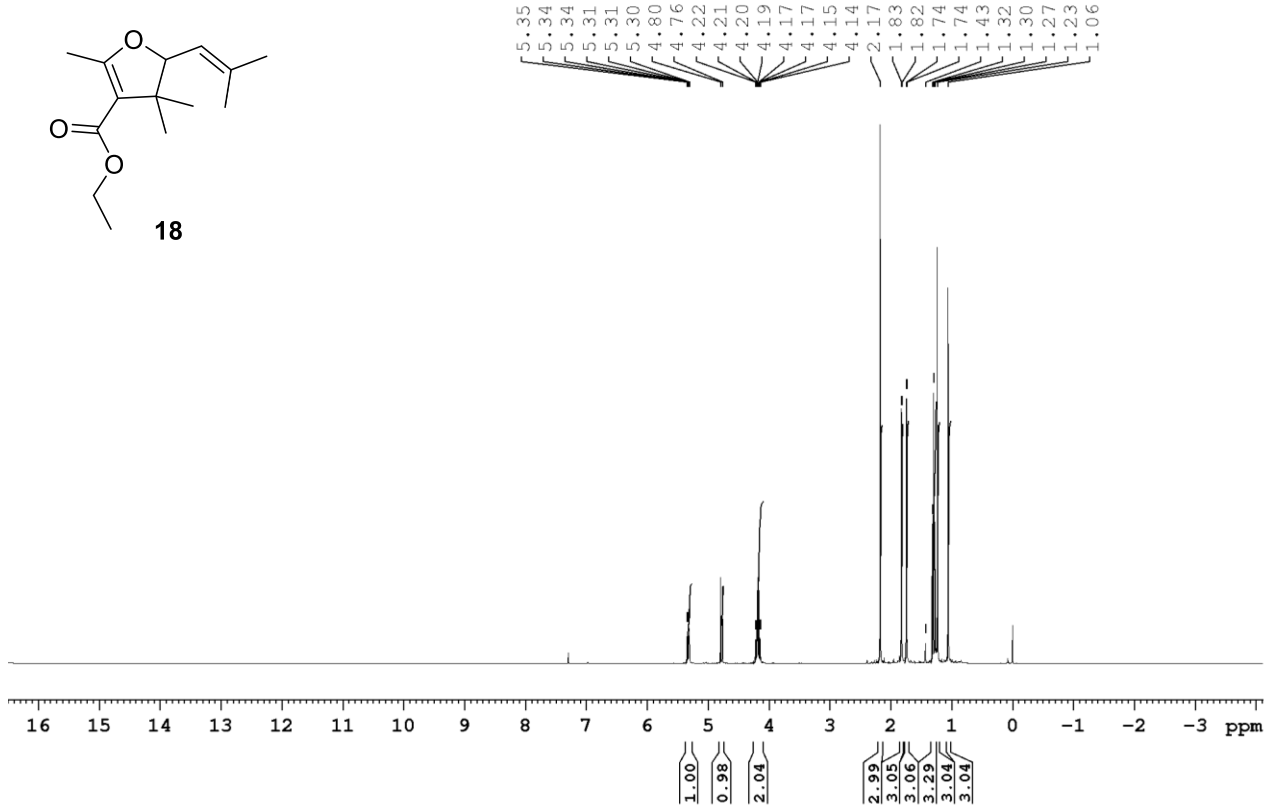
O
O
O
18
5.35
5.34
5.34
5.31
5.31
5.30
4.80
4.76
4.22
4.21
4.20
4.19
4.17
4.17
4.15
4.14
2.17
1.83
1.82
1.74
1.74
1.43
1.32
1.30
1.27
1.23
1.06
16
15
14
13
12
11
10
9
8
7
6
5
4
3
2
1
0
−1
−2
−3
ppm
1.00
0.98
2.04
2.99
3.05
3.06
3.29
3.04
3.04

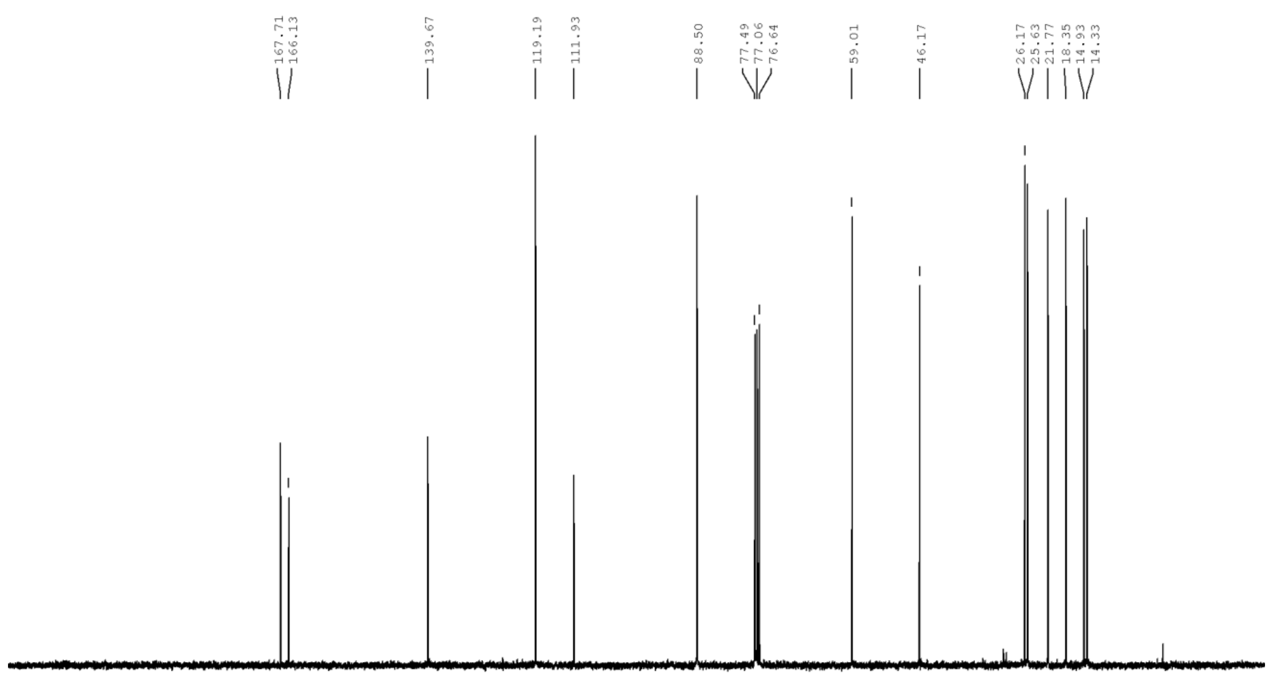
167.71
166.13
139.67
119.19
111.93
88.50
77.49
77.06
76.64
59.01
46.17
26.17
25.63
21.77
18.35
14.93
14.33
200
180
160
140
120
100
80
60
40
20
0
ppm

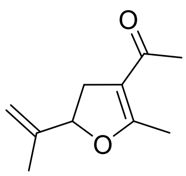

20

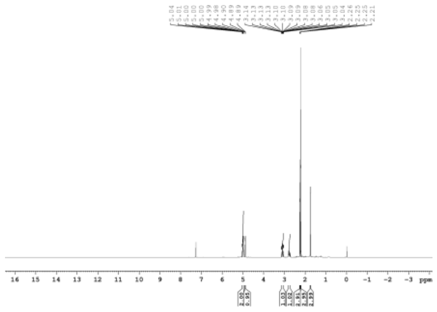

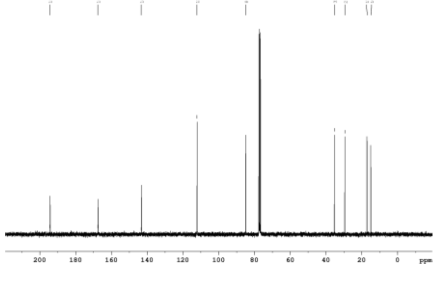

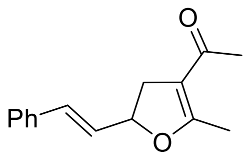

22

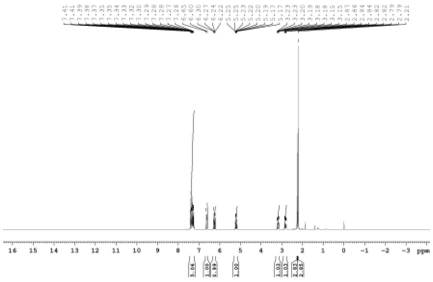

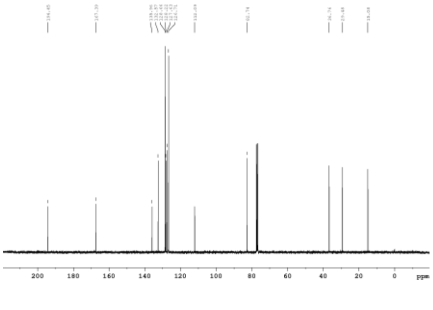

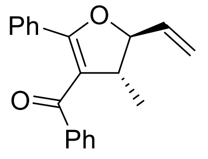
Ph
O
O
Ph

24

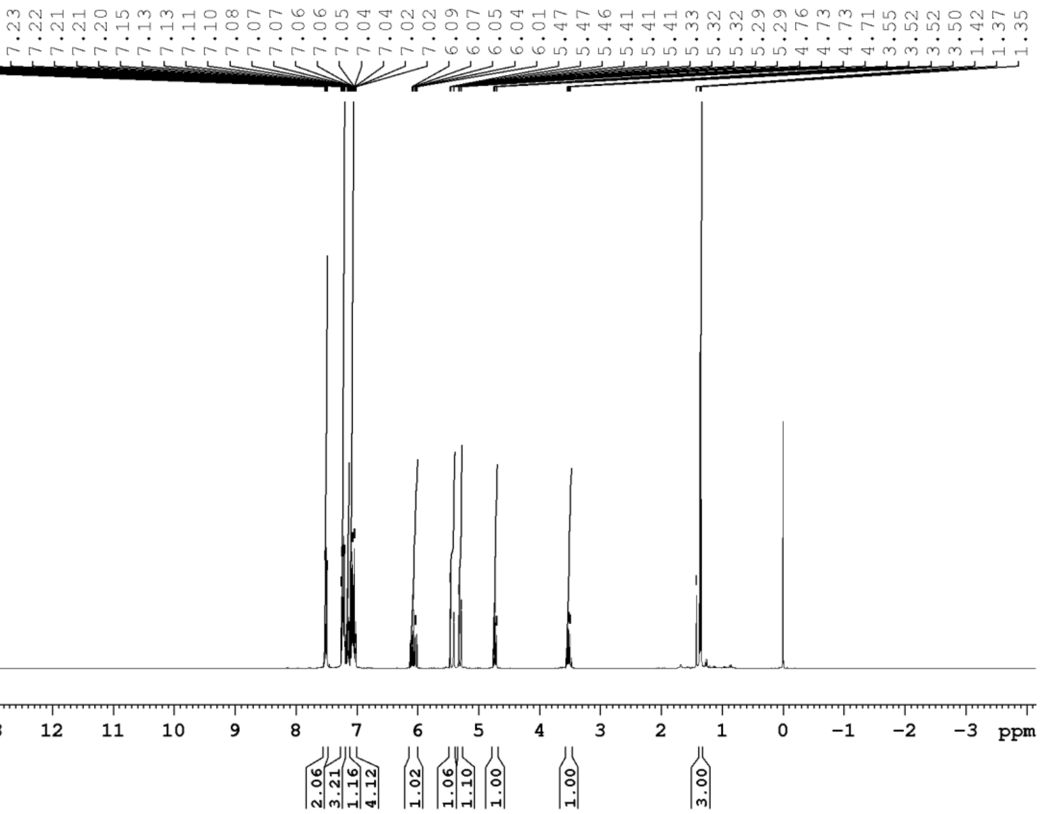
7.52 7.52 7.50 7.49 7.26 7.25 7.24 7.24 7.23 7.23 7.22 7.21 7.21 7.20 7.15 7.13 7.13 7.11 7.10 7.08 7.07 7.07 7.06 7.06 7.05 7.04 7.04 7.02 7.02 6.09 6.07 6.05 6.04 6.01 5.47 5.46 5.41 5.41 5.41 5.33 5.32 5.32 5.29 5.29 4.76 4.73 4.73 4.71 3.55 3.52 3.52 3.50 1.42 1.37 1.35
16 15 14 13 12 11 10 9 8 7 6 5 4 3 2 1 0 −1 −2 −3 ppm
2.06 3.21 1.16 4.12 1.02 1.06 1.10 1.00 1.00 3.00

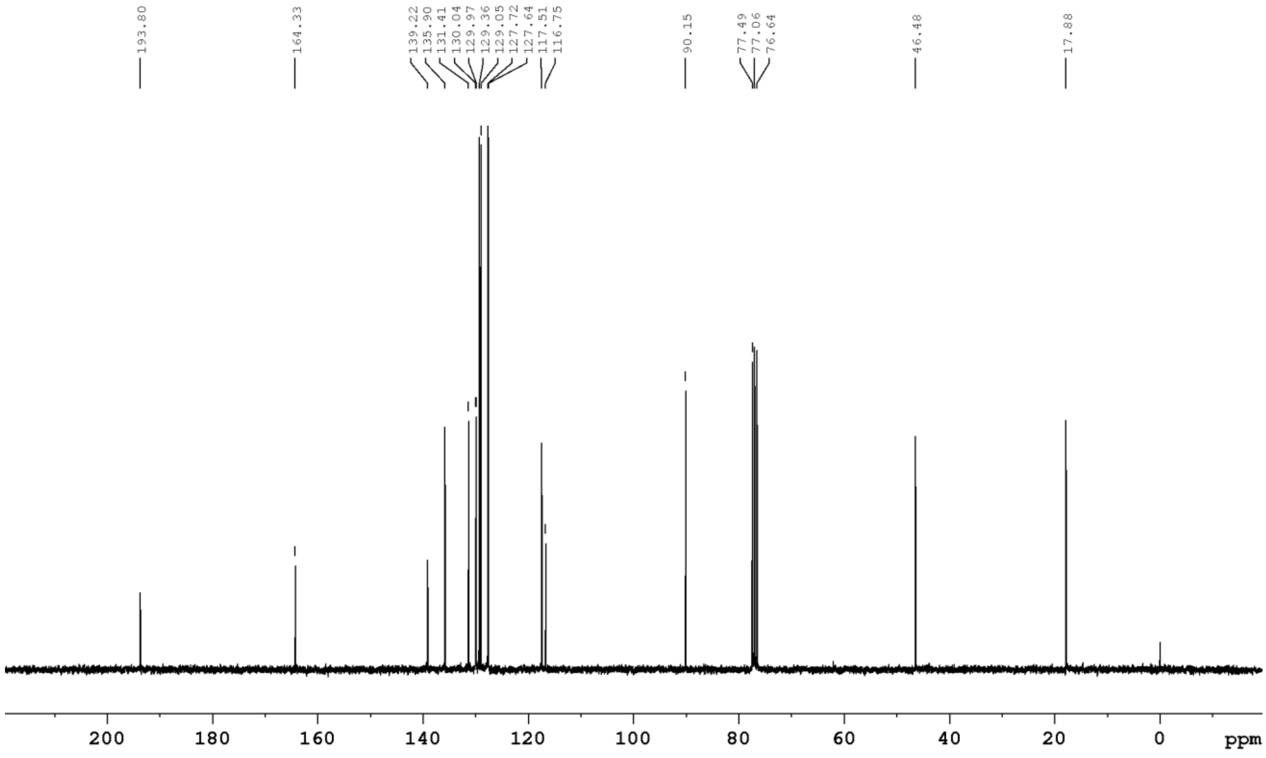
193.80
164.33
139.22 135.90 131.41 130.04 129.97 129.36 129.05 127.64 127.72 117.51 116.75
90.15
77.49 77.06 76.64
46.48
17.88
200 180 160 140 120 100 80 60 40 20 0 ppm

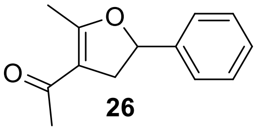

26

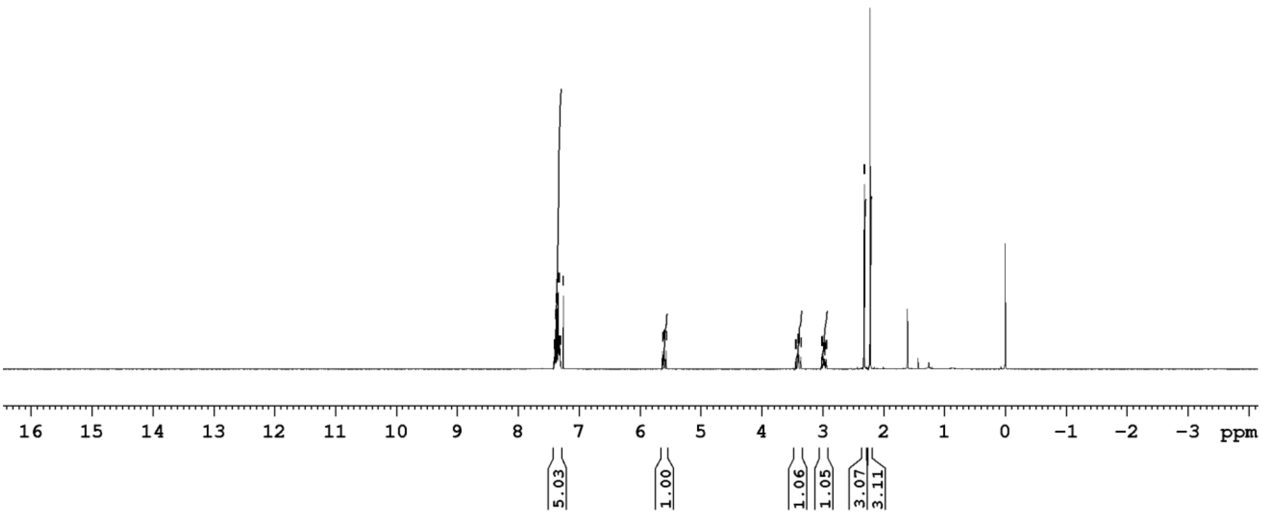
7.41 7.41 7.39 7.38 7.37 7.37 7.36 7.35 7.34 7.33 7.33 7.32 7.32 7.30 7.26 5.63 5.60 5.60 5.57 3.44 3.44 3.41 3.40 3.40 3.39 3.36 3.36 3.02 3.01 2.99 2.99 2.97 2.97 2.94 2.94 2.32 2.32 2.31 2.22
16 15 14 13 12 11 10 9 8 7 6 5 4 3 2 1 0 −1 −2 −3 ppm
5.03 1.00 1.06 1.05 3.07 3.11

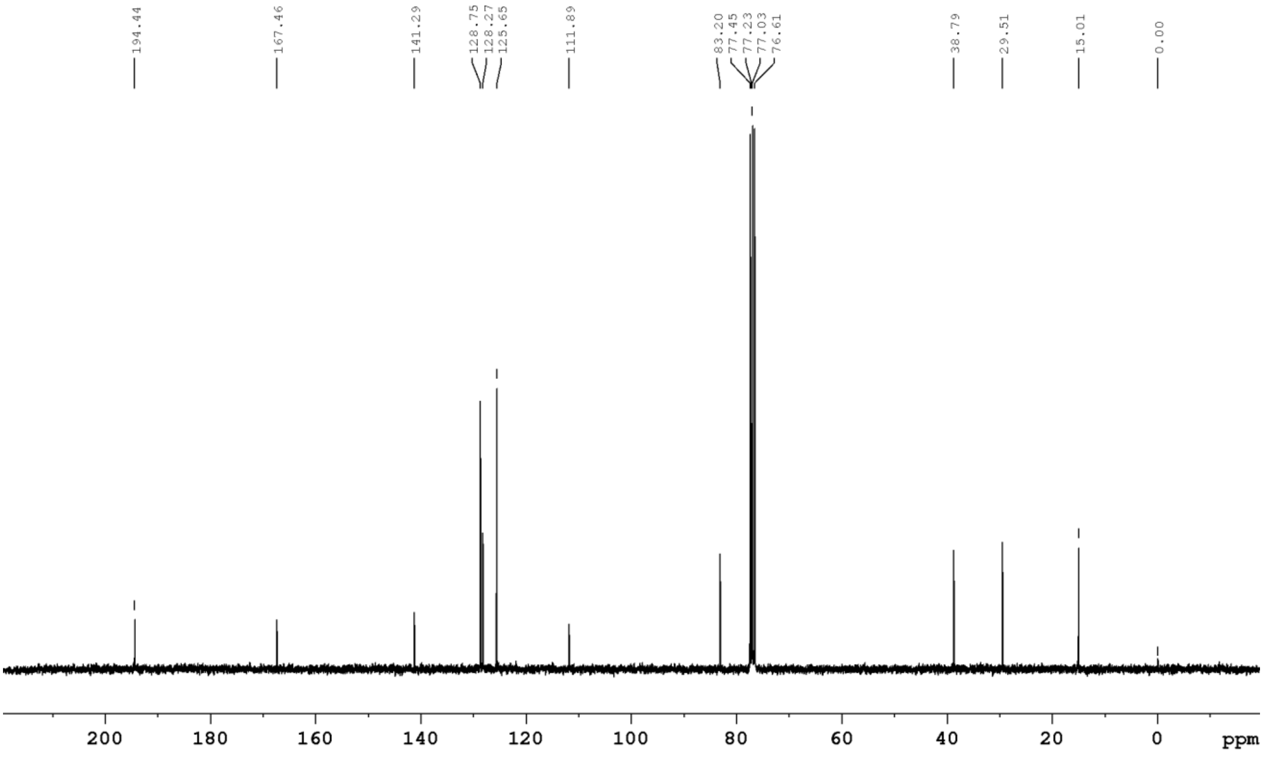
194.44 167.46 141.29 128.75 128.27 125.65 111.89 83.20 77.45 77.23 77.03 76.61 38.79 29.51 15.01 0.00
200 180 160 140 120 100 80 60 40 20 0 ppm

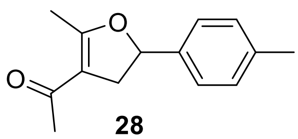

28

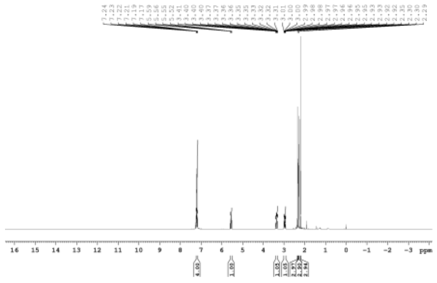

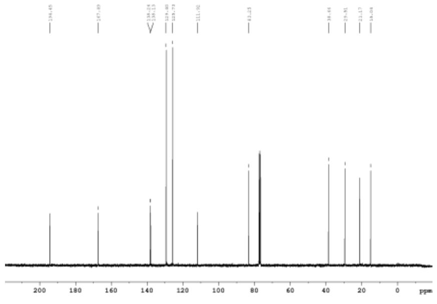

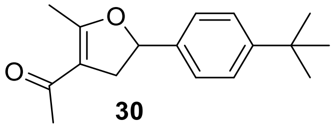

30

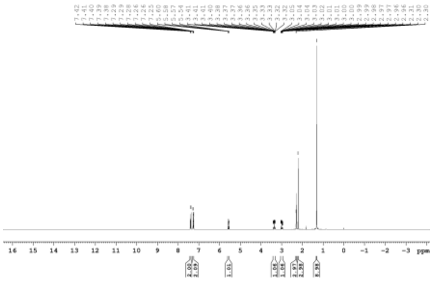

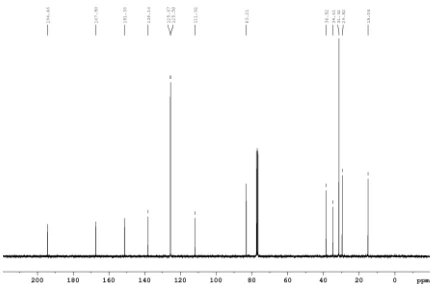

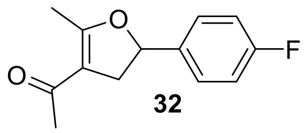
O
O
F

32

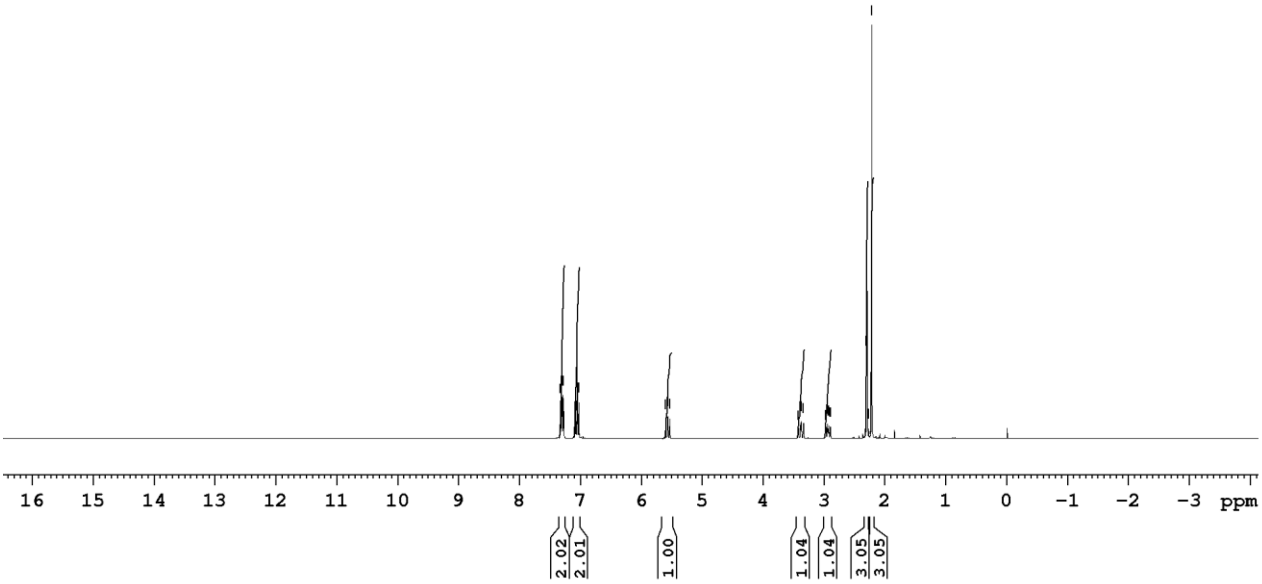
7.33
7.32
7.31
7.31
7.30
7.29
7.28
7.09
7.08
7.07
7.06
7.05
7.04
7.03
5.60
5.57
5.57
5.54
3.43
3.42
3.39
3.39
3.38
3.38
3.35
3.34
2.98
2.97
2.95
2.95
2.93
2.93
2.90
2.90
2.31
2.30
2.30
2.28
2.22
16 15 14 13 12 11 10 9 8 7 6 5 4 3 2 1 0 −1 −2 −3 ppm
2.02
2.01
1.00
1.04
1.04
3.05
3.05

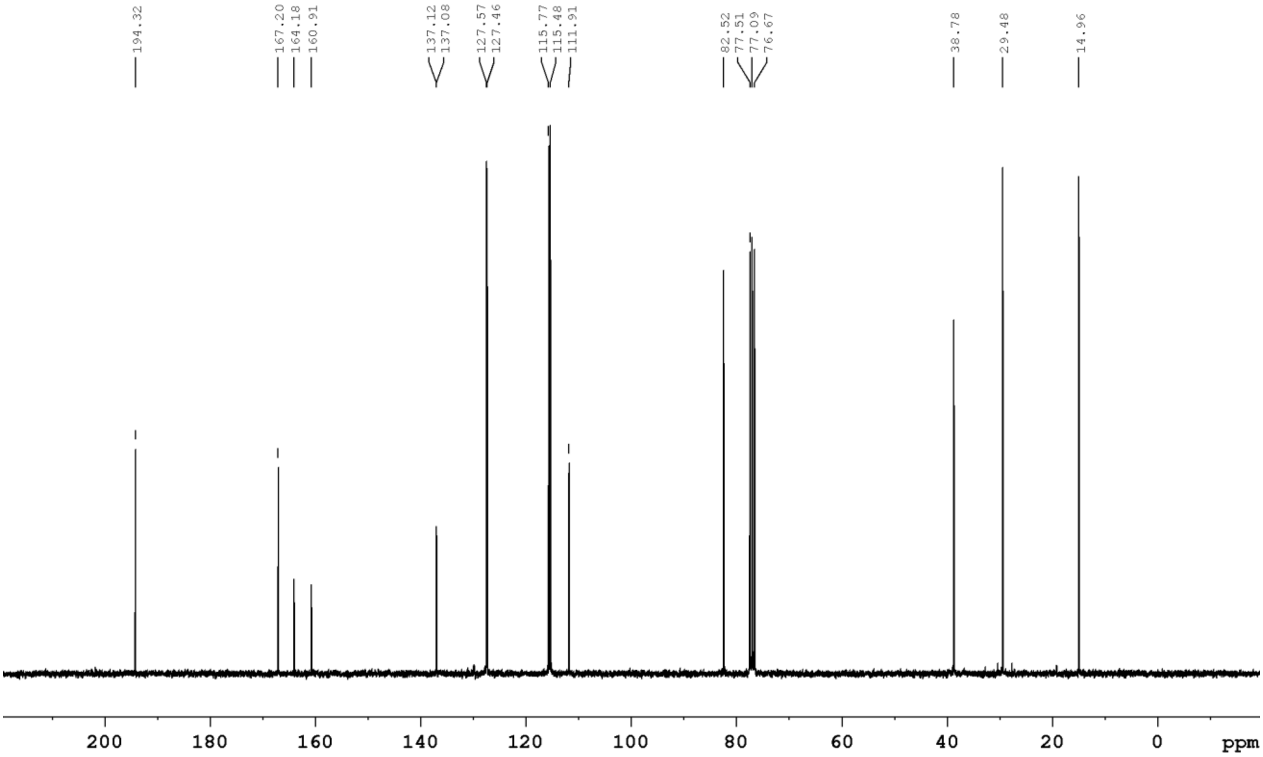
194.32
167.20
164.18
160.91
137.12
137.08
127.57
127.46
115.77
115.48
111.91
82.52
77.51
77.09
76.67
38.78
29.48
14.96
200 180 160 140 120 100 80 60 40 20 0 ppm

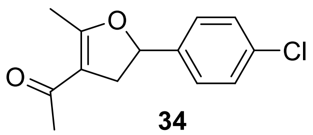
O
Cl

34

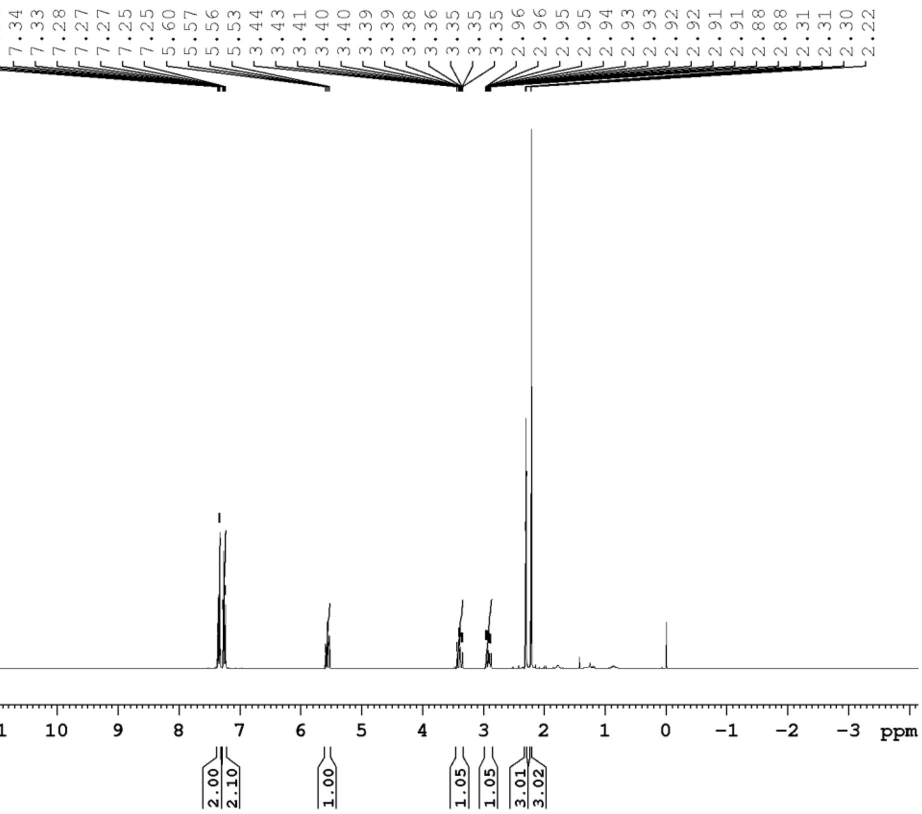
7.37
7.36
7.34
7.34
7.33
7.28
7.27
7.27
7.25
7.25
5.60
5.57
5.56
5.53
3.44
3.43
3.41
3.40
3.39
3.39
3.38
3.36
3.35
3.35
3.35
2.96
2.96
2.95
2.95
2.94
2.93
2.93
2.92
2.92
2.91
2.91
2.88
2.31
2.31
2.30
2.22
16 15 14 13 12 11 10 9 8 7 6 5 4 3 2 1 0 −1 −2 −3 ppm
2.00
2.10
1.00
1.05
1.05
3.01
3.02

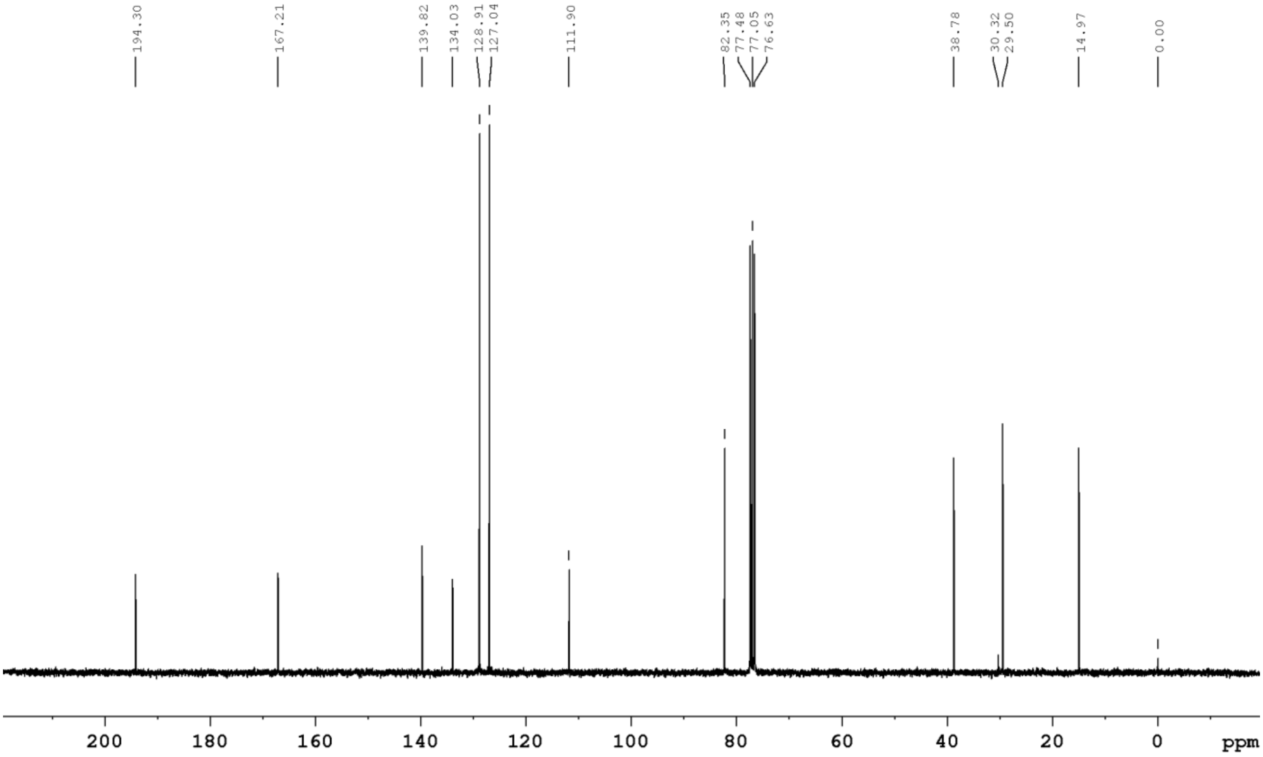
194.30
167.21
139.82
134.03
128.91
127.04
111.90
82.35
77.48
77.05
76.63
38.78
30.32
29.50
14.97
0.00
200 180 160 140 120 100 80 60 40 20 0 ppm

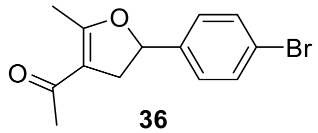

36

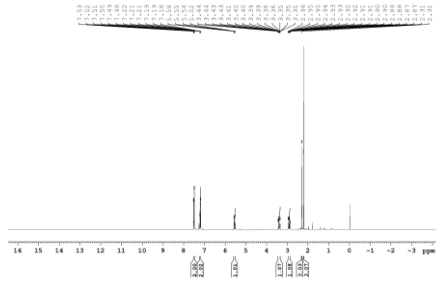

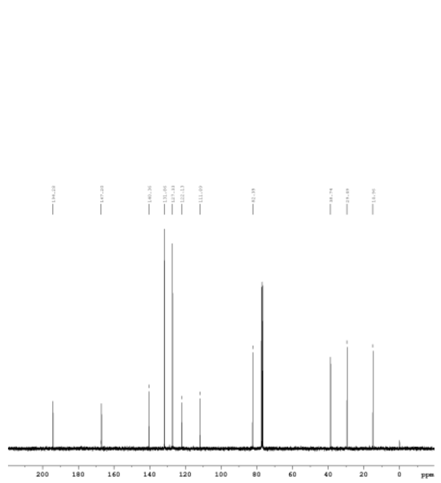

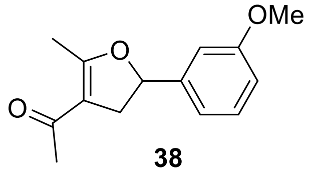
OMe
O

38

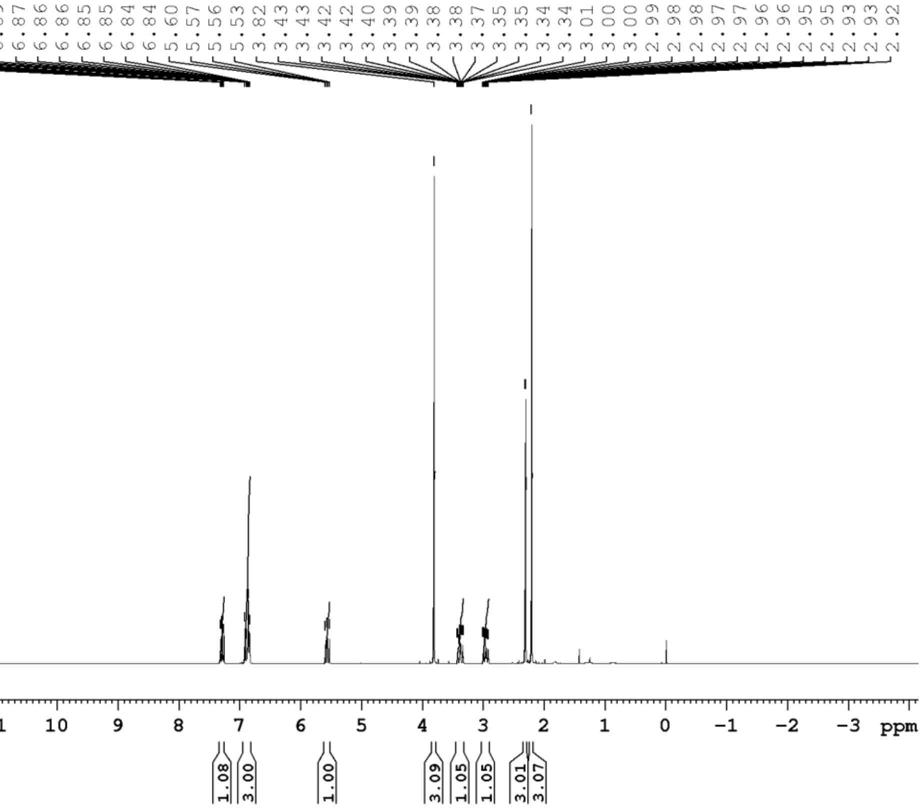
7.32 7.31 7.30 7.29 7.27 6.92 6.92 6.89 6.87 6.86 6.86 6.85 6.85 6.84 6.84 5.60 5.57 5.56 5.53 3.82 3.43 3.43 3.42 3.42 3.40 3.39 3.39 3.38 3.38 3.37 3.35 3.35 3.34 3.34 3.01 3.00 3.00 2.99 2.98 2.98 2.97 2.97 2.96 2.95 2.95 2.93 2.93 2.92
16 15 14 13 12 11 10 9 8 7 6 5 4 3 2 1 0 −1 −2 −3 ppm
1.08 3.00 1.00 3.09 1.05 1.05 3.01 3.07

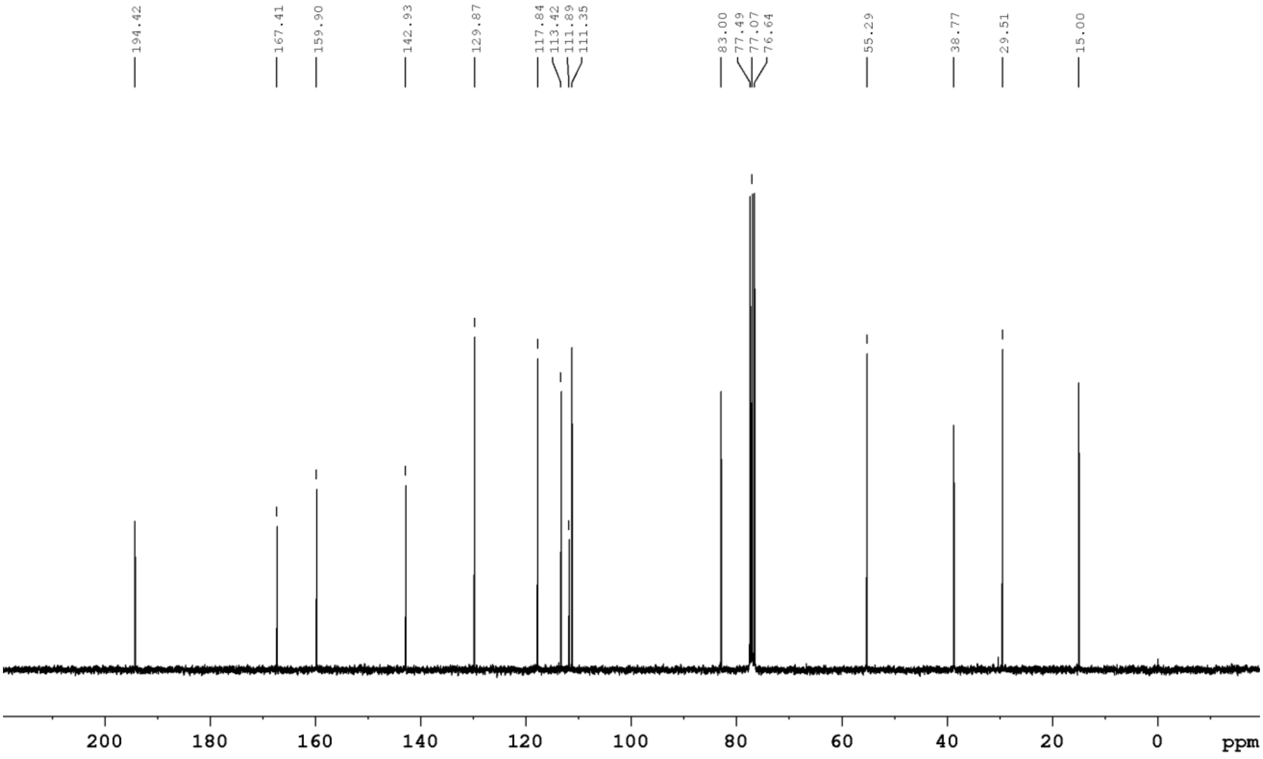
194.42 167.41 159.90 142.93 129.87 117.84 113.42 111.89 111.35 83.00 77.49 77.07 76.64 55.29 38.77 29.51 15.00
200 180 160 140 120 100 80 60 40 20 0 ppm

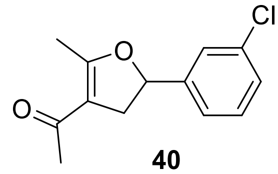
O
O
Cl

40

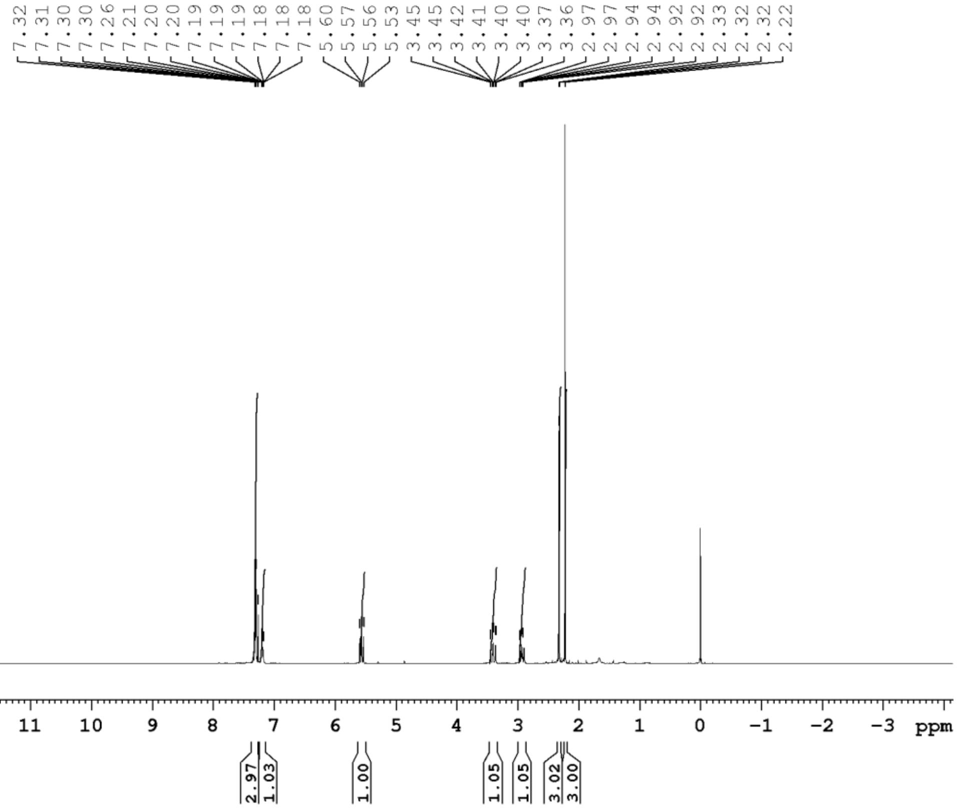
7.32
7.31
7.30
7.30
7.26
7.21
7.20
7.20
7.19
7.19
7.19
7.18
7.18
7.18
5.60
5.57
5.56
5.53
3.45
3.45
3.42
3.41
3.40
3.40
3.37
3.36
2.97
2.97
2.94
2.94
2.92
2.92
2.33
2.32
2.32
2.22
16 15 14 13 12 11 10 9 8 7 6 5 4 3 2 1 0 −1 −2 −3 ppm
2.97
1.03
1.00
1.05
1.05
3.02
3.00

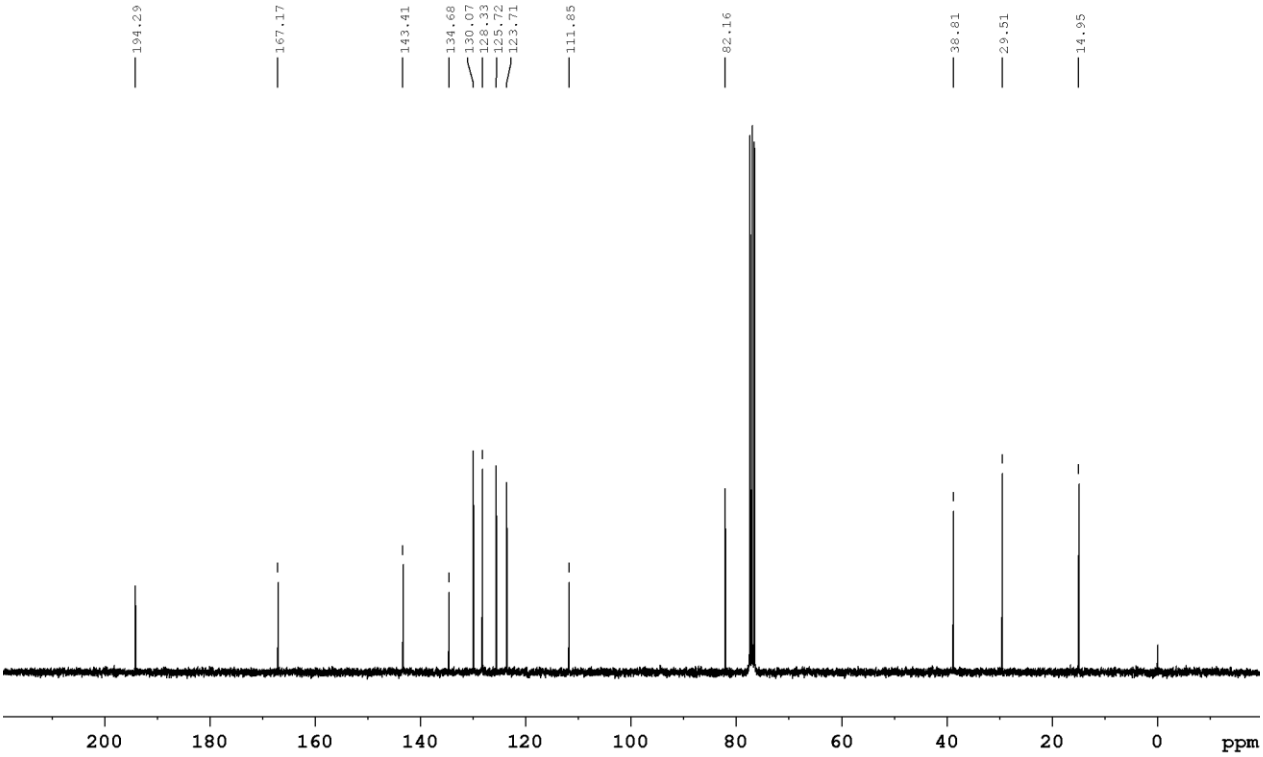
194.29
167.17
143.41
134.68
130.07
128.33
125.72
123.71
111.85
82.16
38.81
29.51
14.95
200 180 160 140 120 100 80 60 40 20 0 ppm

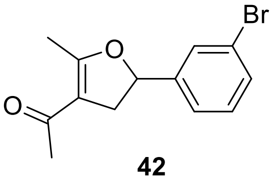

42

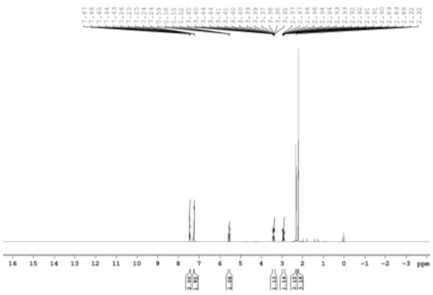

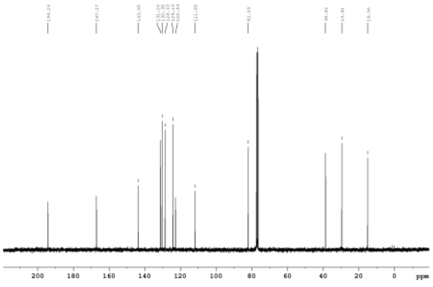

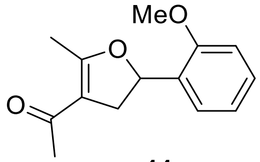

44

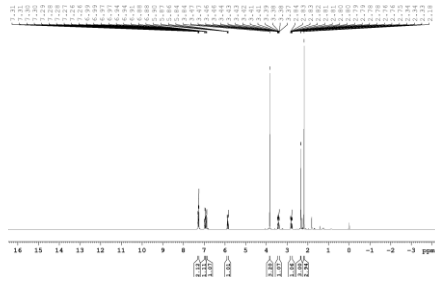

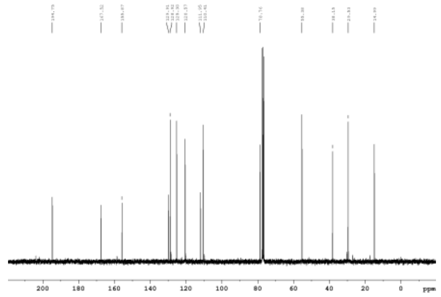

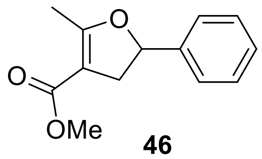
O
O
OMe

46

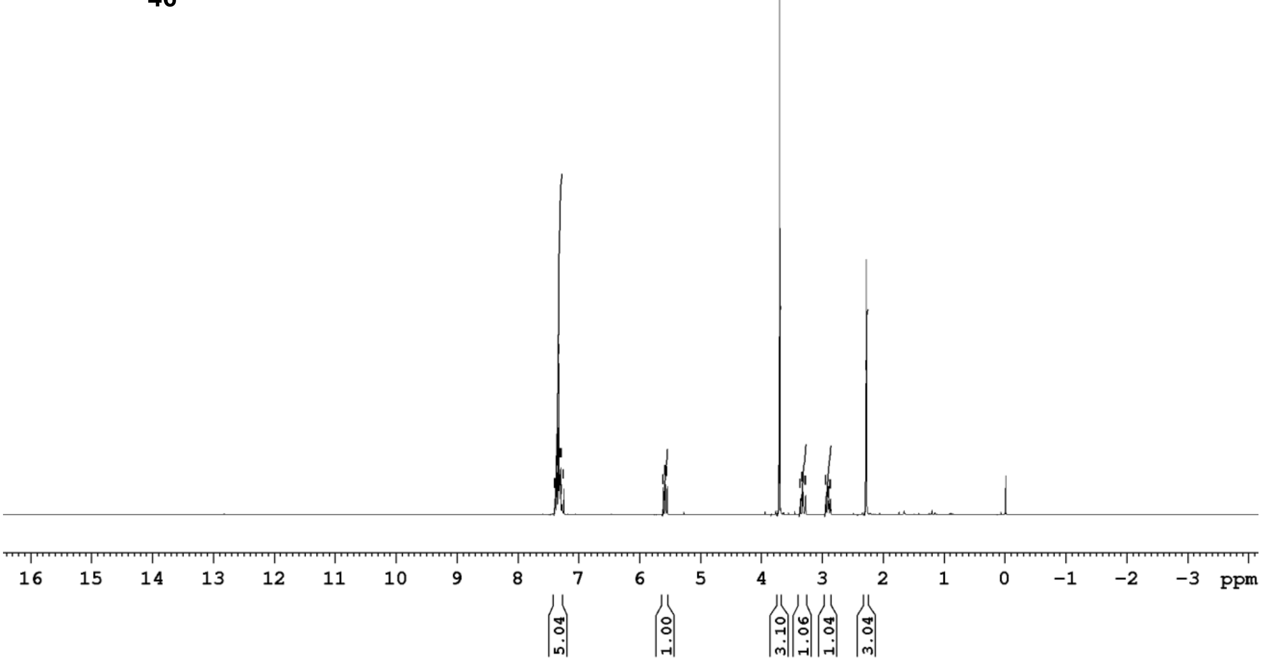
7.40 7.40 7.39 7.37 7.37 7.36 7.35 7.33 7.32 7.31 7.31 7.29 7.29 7.25 5.62 5.59 5.58 5.56 3.71 3.37 3.37 3.34 3.33 3.32 3.32 3.29 3.28 2.95 2.92 2.92 2.90 2.90 2.88 2.87 2.29
16 15 14 13 12 11 10 9 8 7 6 5 4 3 2 1 0 −1 −2 −3 ppm
5.04 1.00 3.10 1.06 1.04 3.04

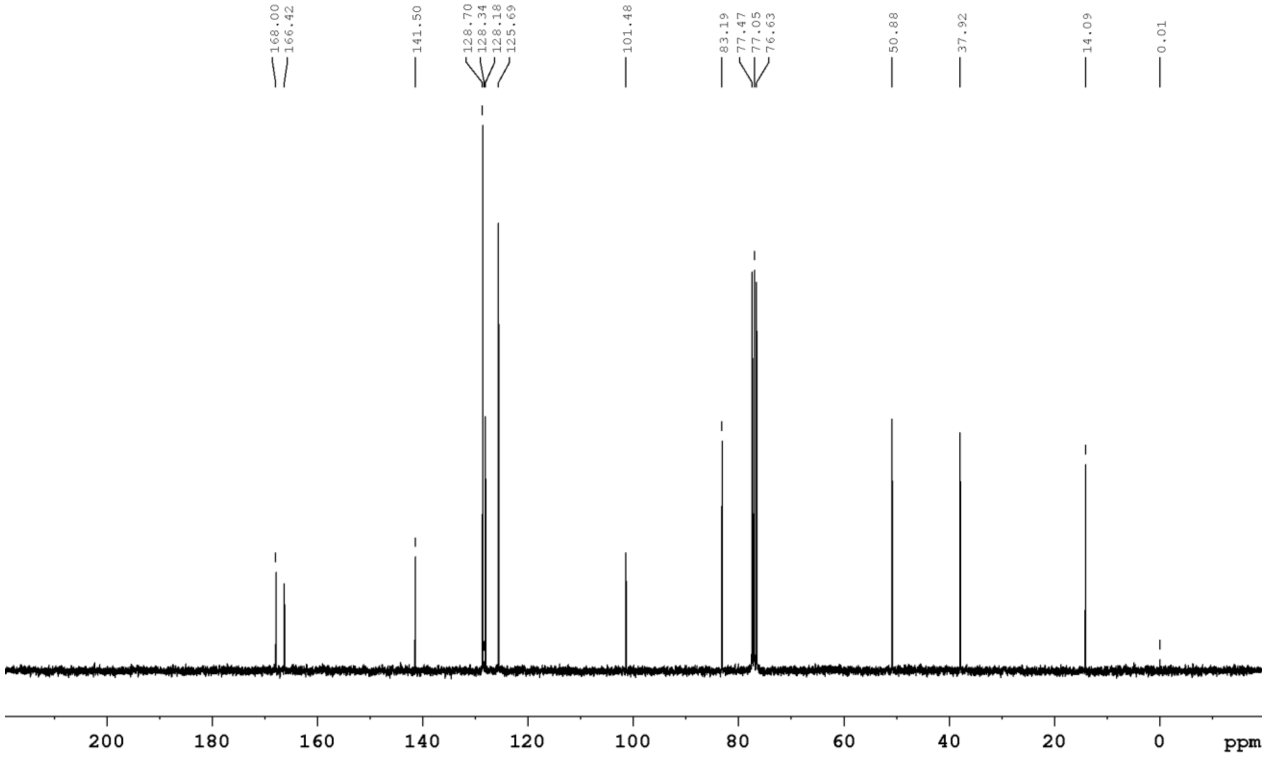
168.00 166.42 141.50 128.70 128.34 128.18 125.69 101.48 83.19 77.47 77.05 76.63 50.88 37.92 14.09 0.01
200 180 160 140 120 100 80 60 40 20 0 ppm

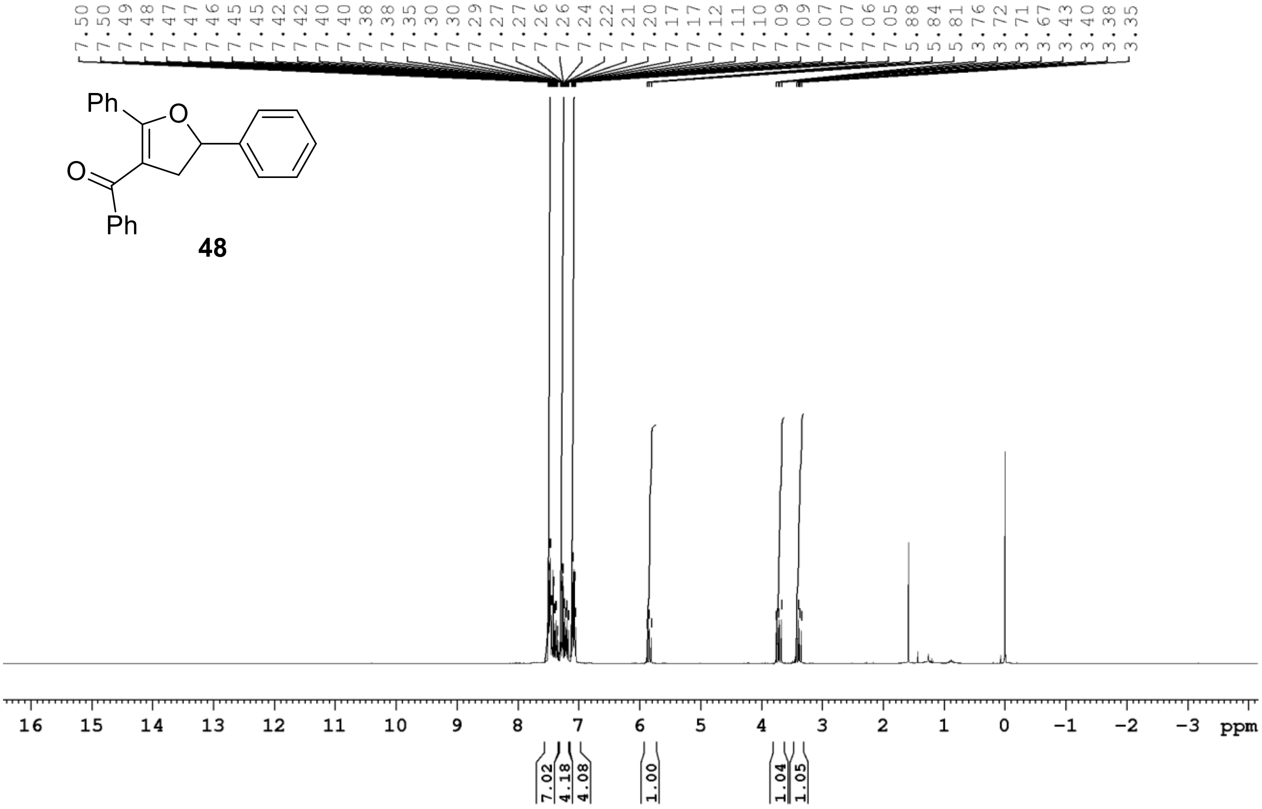
7.50 7.50 7.49 7.48 7.47 7.47 7.46 7.45 7.45 7.42 7.42 7.40 7.40 7.38 7.38 7.35 7.30 7.30 7.29 7.27 7.27 7.26 7.26 7.24 7.22 7.21 7.20 7.17 7.17 7.12 7.11 7.10 7.09 7.09 7.07 7.07 7.06 7.05 5.88 5.84 5.81 3.76 3.72 3.71 3.67 3.43 3.40 3.38 3.35
Ph
O
O
Ph
48
16 15 14 13 12 11 10 9 8 7 6 5 4 3 2 1 0 −1 −2 −3 ppm
7.02 4.18 4.08 1.00 1.04 1.05

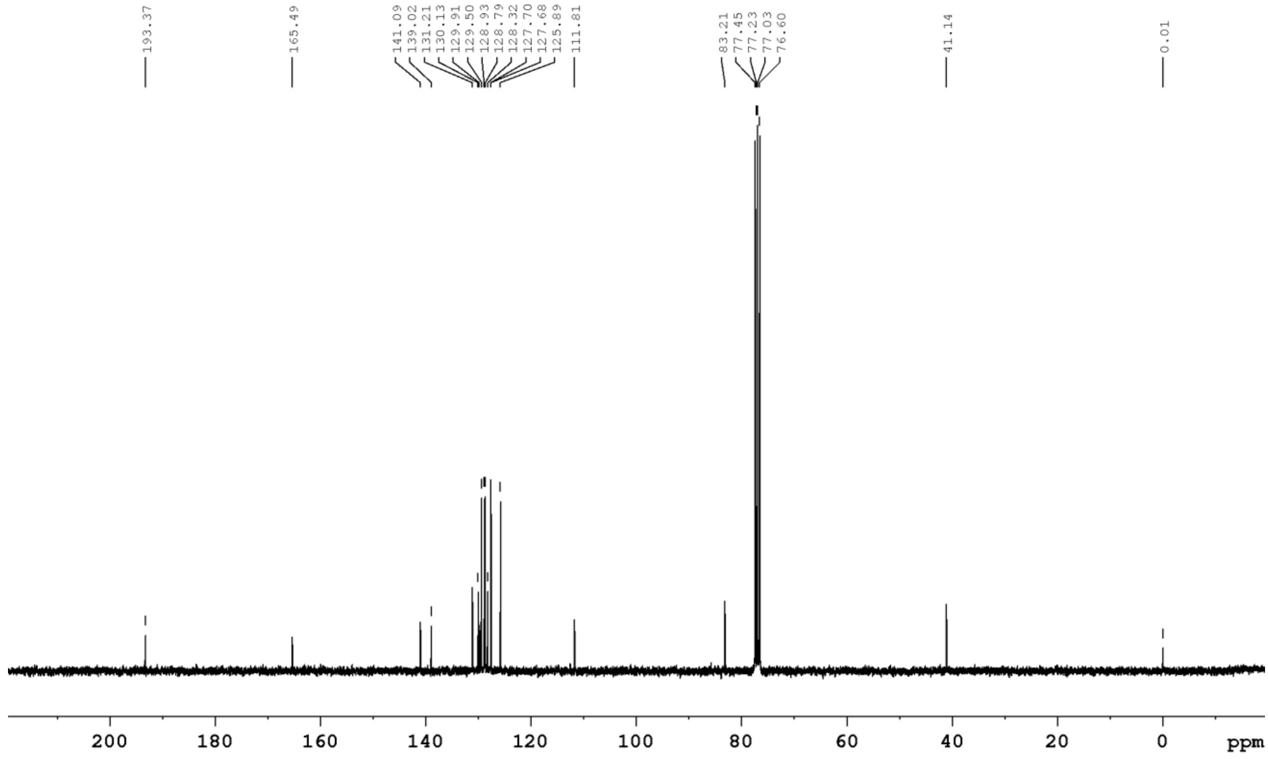
193.37
165.49
141.09 139.02 131.21 130.13 129.91 129.50 128.93 128.79 128.32 127.70 127.68 125.89
111.81
83.21 77.45 77.23 77.03 76.60
41.14
0.01
200 180 160 140 120 100 80 60 40 20 0 ppm

11. Additional information for Crystals

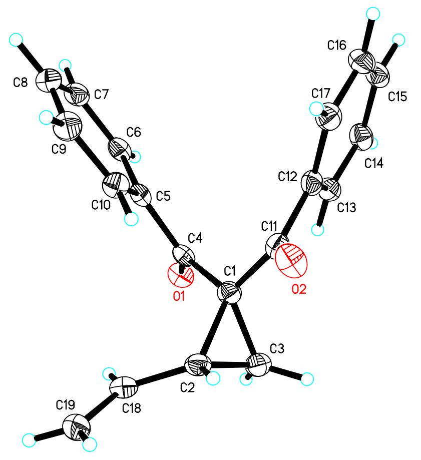

Figure S14: X-ray structure of compound **(R)-3**

Table 3. Crystal data and structure refinement for compound **(R)-3**.

Identification code	s2179lc
Empirical formula	C19 H16 O2
Formula weight	276.32
Temperature	100(2) K
Wavelength	1.54178 A
Crystal system, space group	Monoclinic, C 2
Unit cell dimensions	a = 14.3210(8) A alpha = 90 deg.
	b = 8.9769(5) A beta = 91.660(2)
	c = 11.7100(7) A gamma = 90 deg.

Volume	1504.78(15) A^3
Z, Calculated density	4, 1.220 Mg/m^3
Absorption coefficient	0.618 mm^-1
F(000)	584
Crystal size	0.59 x 0.40 x 0.23 mm
Theta range for data collection	3.78 to 65.82 deg.
Limiting indices	-16<=h<=16, -9<=k<=10, -13<=l<=13
Reflections collected / unique	8918 / 2432 [R(int) = 0.0386]
Completeness to theta = 65.82	98.3 %
Absorption correction	Semi-empirical from equivalents
Max. and min. transmission	0.7528 and 0.6481
Refinement method	Full-matrix least-squares on F^2
Data / restraints / parameters	2432 / 1 / 191
Goodness-of-fit on F^2	1.032

Final R indices [I>2sigma(I)]   R1 = 0.0232, wR2 = 0.0611

R indices (all data)   R1 = 0.0232, wR2 = 0.0612

Absolute structure parameter   0.04(16)

Extinction coefficient   0.0046(3)

Largest diff. peak and hole   0.152 and -0.117 e.A^-3

Table 4. Atomic coordinates (x 10^4) and equivalent isotropic displacement parameters (A^2 x 10^3) for s2179lc. U(eq) is defined as one third of the trace of the orthogonalized Uij tensor.

	x	y	z	U(eq)
O(1)	8220(1)	9840(1)	1002(1)	24(1)
C(1)	6872(1)	11133(1)	1633(1)	20(1)
O(2)	5476(1)	11548(1)	2652(1)	30(1)
C(2)	7035(1)	12823(1)	1512(1)	22(1)
C(3)	6736(1)	11912(2)	491(1)	25(1)
C(4)	7733(1)	10182(1)	1798(1)	19(1)
C(5)	7995(1)	9741(1)	2991(1)	19(1)
C(6)	8571(1)	8501(1)	3169(1)	22(1)
C(7)	8859(1)	8112(2)	4265(1)	26(1)

C(8)	8596(1)	8970(2)	5191(1)	28(1)
C(9)	8031(1)	10208(2)	5016(1)	28(1)
C(10)	7723(1)	10585(1)	3923(1)	23(1)
C(11)	6006(1)	10654(1)	2228(1)	21(1)
C(12)	5796(1)	9026(1)	2243(1)	21(1)
C(13)	6034(1)	8118(1)	1328(1)	22(1)
C(14)	5790(1)	6619(1)	1320(1)	25(1)
C(15)	5331(1)	6018(2)	2237(1)	26(1)
C(16)	5107(1)	6911(2)	3162(1)	27(1)
C(17)	5330(1)	8410(2)	3161(1)	25(1)
C(18)	7985(1)	13444(1)	1643(1)	23(1)
C(19)	8159(1)	14773(2)	2084(1)	26(1)

Table 5. Bond lengths [A] and angles [deg] for s2179lc.

O(1)-C(4)	1.2193(14)
C(1)-C(11)	1.5030(16)
C(1)-C(4)	1.5080(17)
C(1)-C(3)	1.5170(16)
C(1)-C(2)	1.5418(17)
O(2)-C(11)	1.2202(15)
C(2)-C(18)	1.4742(17)
C(2)-C(3)	1.5007(17)
C(2)-H(2)	1.0000
C(3)-H(3A)	0.9900
C(3)-H(3B)	0.9900

C(4)-C(5)	1.4898(17)
C(5)-C(10)	1.3926(17)
C(5)-C(6)	1.3972(17)
C(6)-C(7)	1.3818(18)
C(6)-H(6)	0.9500
C(7)-C(8)	1.3898(19)
C(7)-H(7)	0.9500
C(8)-C(9)	1.3863(19)
C(8)-H(8)	0.9500
C(9)-C(10)	1.3838(18)
C(9)-H(9)	0.9500
C(10)-H(10)	0.9500
C(11)-C(12)	1.4921(17)
C(12)-C(17)	1.3964(17)
C(12)-C(13)	1.3969(17)
C(13)-C(14)	1.3903(19)
C(13)-H(13)	0.9500
C(14)-C(15)	1.3841(18)
C(14)-H(14)	0.9500
C(15)-C(16)	1.3933(19)
C(15)-H(15)	0.9500
C(16)-C(17)	1.382(2)
C(16)-H(16)	0.9500
C(17)-H(17)	0.9500
C(18)-C(19)	1.321(2)
C(18)-H(18)	0.9500
C(19)-H(19A)	0.9500
C(19)-H(19B)	0.9500

C(11)-C(1)-C(4)	117.47(10)
C(11)-C(1)-C(3)	117.00(10)
C(4)-C(1)-C(3)	117.27(10)
C(11)-C(1)-C(2)	116.97(10)
C(4)-C(1)-C(2)	116.31(10)
C(3)-C(1)-C(2)	58.75(8)
C(18)-C(2)-C(3)	122.09(10)
C(18)-C(2)-C(1)	120.30(10)
C(3)-C(2)-C(1)	59.80(8)
C(18)-C(2)-H(2)	114.6
C(3)-C(2)-H(2)	114.6
C(1)-C(2)-H(2)	114.6
C(2)-C(3)-C(1)	61.45(7)
C(2)-C(3)-H(3A)	117.6
C(1)-C(3)-H(3A)	117.6
C(2)-C(3)-H(3B)	117.6
C(1)-C(3)-H(3B)	117.6
H(3A)-C(3)-H(3B)	114.7
O(1)-C(4)-C(5)	121.11(10)
O(1)-C(4)-C(1)	121.88(10)
C(5)-C(4)-C(1)	116.93(10)
C(10)-C(5)-C(6)	119.57(11)
C(10)-C(5)-C(4)	121.48(10)
C(6)-C(5)-C(4)	118.84(10)
C(7)-C(6)-C(5)	119.92(11)
C(7)-C(6)-H(6)	120.0
C(5)-C(6)-H(6)	120.0

C(6)-C(7)-C(8)	120.27(12)
C(6)-C(7)-H(7)	119.9
C(8)-C(7)-H(7)	119.9
C(9)-C(8)-C(7)	119.93(12)
C(9)-C(8)-H(8)	120.0
C(7)-C(8)-H(8)	120.0
C(10)-C(9)-C(8)	120.10(12)
C(10)-C(9)-H(9)	120.0
C(8)-C(9)-H(9)	120.0
C(9)-C(10)-C(5)	120.19(11)
C(9)-C(10)-H(10)	119.9
C(5)-C(10)-H(10)	119.9
O(2)-C(11)-C(12)	120.76(11)
O(2)-C(11)-C(1)	122.12(11)
C(12)-C(11)-C(1)	117.08(10)
C(17)-C(12)-C(13)	119.48(11)
C(17)-C(12)-C(11)	119.90(11)
C(13)-C(12)-C(11)	120.58(11)
C(14)-C(13)-C(12)	120.20(11)
C(14)-C(13)-H(13)	119.9
C(12)-C(13)-H(13)	119.9
C(15)-C(14)-C(13)	119.82(12)
C(15)-C(14)-H(14)	120.1
C(13)-C(14)-H(14)	120.1
C(14)-C(15)-C(16)	120.27(12)
C(14)-C(15)-H(15)	119.9
C(16)-C(15)-H(15)	119.9
C(17)-C(16)-C(15)	120.07(11)

C(17)-C(16)-H(16)	120.0
C(15)-C(16)-H(16)	120.0
C(16)-C(17)-C(12)	120.12(11)
C(16)-C(17)-H(17)	119.9
C(12)-C(17)-H(17)	119.9
C(19)-C(18)-C(2)	123.10(11)
C(19)-C(18)-H(18)	118.5
C(2)-C(18)-H(18)	118.5
C(18)-C(19)-H(19A)	120.0
C(18)-C(19)-H(19B)	120.0
H(19A)-C(19)-H(19B)	120.0

Symmetry transformations used to generate equivalent atoms:

Table 6. Anisotropic displacement parameters (A^2 x 10^3) for s2179lc. The anisotropic displacement factor exponent takes the form: -2 pi^2 [h^2 a*^2 U11 + ... + 2 h k a* b* U12]

	U11	U22	U33	U23	U13	U12
O(1)	26(1)	22(1)	25(1)	-4(1)	6(1)	0(1)
C(1)	24(1)	16(1)	21(1)	-1(1)	-1(1)	0(1)
O(2)	26(1)	24(1)	41(1)	-7(1)	7(1)	1(1)
C(2)	26(1)	16(1)	23(1)	2(1)	-1(1)	1(1)
C(3)	30(1)	22(1)	23(1)	3(1)	-3(1)	-1(1)
C(4)	21(1)	13(1)	23(1)	-2(1)	3(1)	-4(1)

C(5)	16(1)	16(1)	25(1)	-1(1)	2(1)	-3(1)
C(6)	20(1)	16(1)	30(1)	-1(1)	3(1)	-2(1)
C(7)	24(1)	18(1)	36(1)	6(1)	-3(1)	-1(1)
C(8)	27(1)	31(1)	26(1)	9(1)	-3(1)	-6(1)
C(9)	28(1)	31(1)	24(1)	-2(1)	4(1)	-1(1)
C(10)	22(1)	21(1)	25(1)	0(1)	2(1)	3(1)
C(11)	20(1)	22(1)	22(1)	-1(1)	-2(1)	2(1)
C(12)	16(1)	22(1)	24(1)	0(1)	-1(1)	1(1)
C(13)	22(1)	22(1)	24(1)	1(1)	1(1)	-1(1)
C(14)	22(1)	22(1)	31(1)	-4(1)	1(1)	0(1)
C(15)	21(1)	19(1)	40(1)	3(1)	0(1)	-1(1)
C(16)	21(1)	29(1)	31(1)	7(1)	4(1)	-3(1)
C(17)	21(1)	28(1)	25(1)	0(1)	2(1)	1(1)
C(18)	27(1)	19(1)	22(1)	4(1)	2(1)	3(1)
C(19)	26(1)	22(1)	31(1)	1(1)	-1(1)	-1(1)

Table 7. Hydrogen coordinates (x 10^4) and isotropic displacement parameters (A^2 x 10^3) for s2179lc.

	x	y	z	U(eq)
H(2)	6532	13460	1830	26
H(3A)	7204	11725	-99	30
H(3B)	6087	12041	189	30

H(6)	8764	7926	2537	27
H(7)	9238	7255	4387	31
H(8)	8804	8708	5942	34
H(9)	7855	10798	5648	33
H(10)	7325	11421	3808	27
H(13)	6364	8525	709	27
H(14)	5937	6009	688	30
H(15)	5169	4992	2235	32
H(16)	4800	6491	3795	32
H(17)	5165	9021	3786	30
H(18)	8496	12862	1399	27
H(19A)	7660	15377	2334	32
H(19B)	8784	15126	2152	32

Table 8. Torsion angles [deg] for s2179lc.

C(11)-C(1)-C(2)-C(18)	141.50(11)
C(4)-C(1)-C(2)-C(18)	-4.50(16)
C(3)-C(1)-C(2)-C(18)	-111.82(13)
C(11)-C(1)-C(2)-C(3)	-106.68(11)
C(4)-C(1)-C(2)-C(3)	107.33(11)
C(18)-C(2)-C(3)-C(1)	108.91(12)
C(11)-C(1)-C(3)-C(2)	106.63(11)
C(4)-C(1)-C(3)-C(2)	-105.70(11)
C(11)-C(1)-C(4)-O(1)	135.43(11)
C(3)-C(1)-C(4)-O(1)	-12.09(16)

C(2)-C(1)-C(4)-O(1)	-78.75(14)
C(11)-C(1)-C(4)-C(5)	-47.73(14)
C(3)-C(1)-C(4)-C(5)	164.75(10)
C(2)-C(1)-C(4)-C(5)	98.09(12)
O(1)-C(4)-C(5)-C(10)	151.98(11)
C(1)-C(4)-C(5)-C(10)	-24.89(16)
O(1)-C(4)-C(5)-C(6)	-24.20(17)
C(1)-C(4)-C(5)-C(6)	158.93(10)
C(10)-C(5)-C(6)-C(7)	0.65(17)
C(4)-C(5)-C(6)-C(7)	176.91(10)
C(5)-C(6)-C(7)-C(8)	-1.54(17)
C(6)-C(7)-C(8)-C(9)	1.02(18)
C(7)-C(8)-C(9)-C(10)	0.41(18)
C(8)-C(9)-C(10)-C(5)	-1.30(18)
C(6)-C(5)-C(10)-C(9)	0.77(17)
C(4)-C(5)-C(10)-C(9)	-175.39(11)
C(4)-C(1)-C(11)-O(2)	141.85(12)
C(3)-C(1)-C(11)-O(2)	-70.54(15)
C(2)-C(1)-C(11)-O(2)	-3.75(16)
C(4)-C(1)-C(11)-C(12)	-40.41(14)
C(3)-C(1)-C(11)-C(12)	107.20(12)
C(2)-C(1)-C(11)-C(12)	173.99(10)
O(2)-C(11)-C(12)-C(17)	-33.00(17)
C(1)-C(11)-C(12)-C(17)	149.23(11)
O(2)-C(11)-C(12)-C(13)	144.85(11)
C(1)-C(11)-C(12)-C(13)	-32.92(16)
C(17)-C(12)-C(13)-C(14)	1.42(17)
C(11)-C(12)-C(13)-C(14)	-176.44(11)

C(12)-C(13)-C(14)-C(15)	-1.71(18)
C(13)-C(14)-C(15)-C(16)	0.54(18)
C(14)-C(15)-C(16)-C(17)	0.93(18)
C(15)-C(16)-C(17)-C(12)	-1.22(18)
C(13)-C(12)-C(17)-C(16)	0.05(17)
C(11)-C(12)-C(17)-C(16)	177.93(11)
C(3)-C(2)-C(18)-C(19)	142.07(12)
C(1)-C(2)-C(18)-C(19)	-146.68(12)

Symmetry transformations used to generate equivalent atoms:

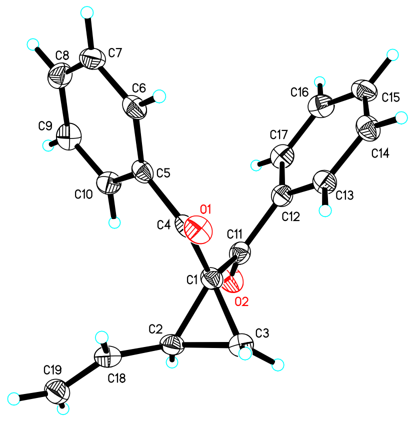

Figure S15: X-ray structure of compound **(S)-3**

Table 9. Crystal data and structure refinement for **(S)-3**.

Identification code	s2181lc
Empirical formula	C19 H16 O2
Formula weight	276.32
Temperature	100(2) K
Wavelength	1.54178 A
Crystal system, space group	Monoclinic, C 2
Unit cell dimensions	a = 14.3185(10) A alpha = 90 deg.
	b = 8.9738(6) A beta = 91.675(3)
	c = 11.7148(8) A gamma = 90 deg.

Volume 1504.61(18) A^3

Z, Calculated density 4, 1.220 Mg/m^3

Absorption coefficient 0.619 mm^-1

F(000) 584

Crystal size 0.53 x 0.49 x 0.27 mm

Theta range for data collection 3.77 to 65.86 deg.

Limiting indices -16<=h<=16, -9<=k<=10, -13<=l<=13

Reflections collected / unique 8708 / 2353 [R(int) = 0.0340]

Completeness to theta = 65.86 96.5 %

Absorption correction Semi-empirical from equivalents

Max. and min. transmission 0.7528 and 0.6797

Refinement method Full-matrix least-squares on F^2

Data / restraints / parameters 2353 / 1 / 191

Goodness-of-fit on F^2 1.055

Final R indices [I>2sigma(I)]   R1 = 0.0233, wR2 = 0.0593

R indices (all data)   R1 = 0.0233, wR2 = 0.0594

Absolute structure parameter   0.00(16)

Extinction coefficient   0.0041(2)

Largest diff. peak and hole   0.137 and -0.101 e.A^-3

Table 10. Atomic coordinates (x 10^4) and equivalent isotropic displacement parameters (A^2 x 10^3) for s2181lc. U(eq) is defined as one third of the trace of the orthogonalized Uij tensor.

	x	y	z	U(eq)
O(1)	1780(1)	159(1)	8999(1)	25(1)
C(1)	3128(1)	-1133(1)	8367(1)	21(1)
O(2)	4524(1)	-1548(1)	7348(1)	31(1)
C(2)	2965(1)	-2823(1)	8489(1)	23(1)
C(3)	3264(1)	-1913(2)	9509(1)	26(1)
C(4)	2267(1)	-183(1)	8201(1)	20(1)
C(5)	2006(1)	258(1)	7010(1)	20(1)
C(6)	1431(1)	1499(1)	6832(1)	23(1)
C(7)	1142(1)	1889(2)	5737(1)	27(1)
C(8)	1404(1)	1031(2)	4810(1)	29(1)

C(9)	1968(1)	-206(2)	4985(1)	28(1)
C(10)	2276(1)	-584(1)	6078(1)	23(1)
C(11)	3994(1)	-654(1)	7773(1)	22(1)
C(12)	4204(1)	975(1)	7759(1)	22(1)
C(13)	3967(1)	1883(2)	8671(1)	23(1)
C(14)	4210(1)	3380(2)	8681(1)	26(1)
C(15)	4669(1)	3982(2)	7762(1)	27(1)
C(16)	4893(1)	3089(2)	6839(1)	28(1)
C(17)	4670(1)	1591(2)	6839(1)	26(1)
C(18)	2016(1)	-3445(1)	8357(1)	23(1)
C(19)	1841(1)	-4772(2)	7916(1)	27(1)

Table 11. Bond lengths [A] and angles [deg] for s2181lc.

O(1)-C(4)	1.2221(14)
C(1)-C(11)	1.5026(16)
C(1)-C(4)	1.5064(17)
C(1)-C(3)	1.5167(17)
C(1)-C(2)	1.5423(18)
O(2)-C(11)	1.2198(15)
C(2)-C(18)	1.4722(17)
C(2)-C(3)	1.4992(18)
C(2)-H(2)	1.0000
C(3)-H(3A)	0.9900
C(3)-H(3B)	0.9900
C(4)-C(5)	1.4877(17)

C(5)-C(10)	1.3923(17)
C(5)-C(6)	1.3972(18)
C(6)-C(7)	1.3809(18)
C(6)-H(6)	0.9500
C(7)-C(8)	1.392(2)
C(7)-H(7)	0.9500
C(8)-C(9)	1.384(2)
C(8)-H(8)	0.9500
C(9)-C(10)	1.3839(18)
C(9)-H(9)	0.9500
C(10)-H(10)	0.9500
C(11)-C(12)	1.4931(18)
C(12)-C(13)	1.3940(18)
C(12)-C(17)	1.3972(17)
C(13)-C(14)	1.388(2)
C(13)-H(13)	0.9500
C(14)-C(15)	1.3870(19)
C(14)-H(14)	0.9500
C(15)-C(16)	1.391(2)
C(15)-H(15)	0.9500
C(16)-C(17)	1.381(2)
C(16)-H(16)	0.9500
C(17)-H(17)	0.9500
C(18)-C(19)	1.320(2)
C(18)-H(18)	0.9500
C(19)-H(19A)	0.9500
C(19)-H(19B)	0.9500

C(11)-C(1)-C(4)	117.52(10)
C(11)-C(1)-C(3)	117.00(10)
C(4)-C(1)-C(3)	117.29(10)
C(11)-C(1)-C(2)	116.97(10)
C(4)-C(1)-C(2)	116.24(10)
C(3)-C(1)-C(2)	58.69(8)
C(18)-C(2)-C(3)	122.11(11)
C(18)-C(2)-C(1)	120.34(11)
C(3)-C(2)-C(1)	59.80(8)
C(18)-C(2)-H(2)	114.6
C(3)-C(2)-H(2)	114.6
C(1)-C(2)-H(2)	114.6
C(2)-C(3)-C(1)	61.51(8)
C(2)-C(3)-H(3A)	117.6
C(1)-C(3)-H(3A)	117.6
C(2)-C(3)-H(3B)	117.6
C(1)-C(3)-H(3B)	117.6
H(3A)-C(3)-H(3B)	114.7
O(1)-C(4)-C(5)	121.09(11)
O(1)-C(4)-C(1)	121.81(11)
C(5)-C(4)-C(1)	117.03(10)
C(10)-C(5)-C(6)	119.41(11)
C(10)-C(5)-C(4)	121.56(11)
C(6)-C(5)-C(4)	118.93(10)
C(7)-C(6)-C(5)	120.03(11)
C(7)-C(6)-H(6)	120.0
C(5)-C(6)-H(6)	120.0
C(6)-C(7)-C(8)	120.25(12)

C(6)-C(7)-H(7)	119.9
C(8)-C(7)-H(7)	119.9
C(9)-C(8)-C(7)	119.87(12)
C(9)-C(8)-H(8)	120.1
C(7)-C(8)-H(8)	120.1
C(10)-C(9)-C(8)	120.12(12)
C(10)-C(9)-H(9)	119.9
C(8)-C(9)-H(9)	119.9
C(9)-C(10)-C(5)	120.31(12)
C(9)-C(10)-H(10)	119.8
C(5)-C(10)-H(10)	119.8
O(2)-C(11)-C(12)	120.79(11)
O(2)-C(11)-C(1)	122.18(12)
C(12)-C(11)-C(1)	116.99(10)
C(13)-C(12)-C(17)	119.51(11)
C(13)-C(12)-C(11)	120.69(11)
C(17)-C(12)-C(11)	119.76(11)
C(14)-C(13)-C(12)	120.34(11)
C(14)-C(13)-H(13)	119.8
C(12)-C(13)-H(13)	119.8
C(15)-C(14)-C(13)	119.71(12)
C(15)-C(14)-H(14)	120.1
C(13)-C(14)-H(14)	120.1
C(14)-C(15)-C(16)	120.23(13)
C(14)-C(15)-H(15)	119.9
C(16)-C(15)-H(15)	119.9
C(17)-C(16)-C(15)	120.18(11)
C(17)-C(16)-H(16)	119.9

C(15)-C(16)-H(16)	119.9
C(16)-C(17)-C(12)	120.00(12)
C(16)-C(17)-H(17)	120.0
C(12)-C(17)-H(17)	120.0
C(19)-C(18)-C(2)	123.14(11)
C(19)-C(18)-H(18)	118.4
C(2)-C(18)-H(18)	118.4
C(18)-C(19)-H(19A)	120.0
C(18)-C(19)-H(19B)	120.0
H(19A)-C(19)-H(19B)	120.0

Symmetry transformations used to generate equivalent atoms:

Table 12. Anisotropic displacement parameters (A^2 x 10^3) for s2181lc. The anisotropic displacement factor exponent takes the form: -2 pi^2 [h^2 a*^2 U11 + ... + 2 h k a* b* U12]

	U11	U22	U33	U23	U13	U12
O(1)	27(1)	24(1)	25(1)	-3(1)	7(1)	0(1)
C(1)	24(1)	18(1)	22(1)	-2(1)	1(1)	0(1)
O(2)	25(1)	27(1)	41(1)	-7(1)	7(1)	2(1)
C(2)	27(1)	17(1)	24(1)	2(1)	1(1)	2(1)
C(3)	30(1)	24(1)	24(1)	3(1)	-2(1)	-1(1)
C(4)	21(1)	15(1)	23(1)	-3(1)	4(1)	-4(1)

C(5)	16(1)	18(1)	25(1)	-1(1)	3(1)	-3(1)
C(6)	21(1)	18(1)	31(1)	-1(1)	3(1)	-3(1)
C(7)	24(1)	20(1)	36(1)	6(1)	-1(1)	-1(1)
C(8)	27(1)	34(1)	26(1)	8(1)	-3(1)	-6(1)
C(9)	27(1)	33(1)	25(1)	-2(1)	4(1)	-1(1)
C(10)	23(1)	22(1)	25(1)	0(1)	3(1)	2(1)
C(11)	20(1)	24(1)	22(1)	-1(1)	-1(1)	2(1)
C(12)	17(1)	23(1)	25(1)	0(1)	-1(1)	1(1)
C(13)	22(1)	24(1)	25(1)	1(1)	2(1)	-2(1)
C(14)	22(1)	23(1)	32(1)	-4(1)	2(1)	-1(1)
C(15)	22(1)	20(1)	40(1)	4(1)	0(1)	-1(1)
C(16)	22(1)	31(1)	31(1)	8(1)	5(1)	-2(1)
C(17)	21(1)	30(1)	26(1)	0(1)	3(1)	1(1)
C(18)	27(1)	21(1)	22(1)	4(1)	3(1)	3(1)
C(19)	26(1)	23(1)	32(1)	1(1)	0(1)	-1(1)

Table 13. Hydrogen coordinates (x 10^4) and isotropic displacement parameters (A^2 x 10^3) for s2181lc.

	x	y	z	U(eq)
H(2)	3467	-3461	8171	27
H(3A)	3912	-2043	9810	31
H(3B)	2796	-1726	10097	31

H(6)	1239	2076	7463	27
H(7)	762	2746	5616	32
H(8)	1196	1293	4059	35
H(9)	2144	-797	4353	34
H(10)	2673	-1422	6192	28
H(13)	3637	1475	9290	28
H(14)	4063	3990	9313	31
H(15)	4832	5009	7763	33
H(16)	5199	3509	6207	33
H(17)	4834	979	6214	31
H(18)	1505	-2862	8602	28
H(19A)	2340	-5376	7666	32
H(19B)	1216	-5124	7849	32

Table 14. Torsion angles [deg] for s2181lc.

C(11)-C(1)-C(2)-C(18)	-141.50(11)
C(4)-C(1)-C(2)-C(18)	4.48(16)
C(3)-C(1)-C(2)-C(18)	111.84(13)
C(11)-C(1)-C(2)-C(3)	106.65(12)
C(4)-C(1)-C(2)-C(3)	-107.36(11)
C(18)-C(2)-C(3)-C(1)	-108.95(13)
C(11)-C(1)-C(3)-C(2)	-106.61(12)
C(4)-C(1)-C(3)-C(2)	105.58(12)
C(11)-C(1)-C(4)-O(1)	-135.45(12)
C(3)-C(1)-C(4)-O(1)	12.19(17)

C(2)-C(1)-C(4)-O(1)	78.76(15)
C(11)-C(1)-C(4)-C(5)	47.69(15)
C(3)-C(1)-C(4)-C(5)	-164.67(10)
C(2)-C(1)-C(4)-C(5)	-98.10(12)
O(1)-C(4)-C(5)-C(10)	-151.97(12)
C(1)-C(4)-C(5)-C(10)	24.92(16)
O(1)-C(4)-C(5)-C(6)	24.36(17)
C(1)-C(4)-C(5)-C(6)	-158.75(10)
C(10)-C(5)-C(6)-C(7)	-0.49(17)
C(4)-C(5)-C(6)-C(7)	-176.90(11)
C(5)-C(6)-C(7)-C(8)	1.36(18)
C(6)-C(7)-C(8)-C(9)	-0.91(18)
C(7)-C(8)-C(9)-C(10)	-0.41(19)
C(8)-C(9)-C(10)-C(5)	1.28(19)
C(6)-C(5)-C(10)-C(9)	-0.83(18)
C(4)-C(5)-C(10)-C(9)	175.49(11)
C(4)-C(1)-C(11)-O(2)	-141.75(12)
C(3)-C(1)-C(11)-O(2)	70.52(15)
C(2)-C(1)-C(11)-O(2)	3.80(17)
C(4)-C(1)-C(11)-C(12)	40.50(15)
C(3)-C(1)-C(11)-C(12)	-107.24(13)
C(2)-C(1)-C(11)-C(12)	-173.96(11)
O(2)-C(11)-C(12)-C(13)	-144.85(12)
C(1)-C(11)-C(12)-C(13)	32.94(16)
O(2)-C(11)-C(12)-C(17)	33.06(18)
C(1)-C(11)-C(12)-C(17)	-149.15(11)
C(17)-C(12)-C(13)-C(14)	-1.49(18)
C(11)-C(12)-C(13)-C(14)	176.43(11)

C(12)-C(13)-C(14)-C(15)	1.75(18)
C(13)-C(14)-C(15)-C(16)	-0.56(18)
C(14)-C(15)-C(16)-C(17)	-0.91(18)
C(15)-C(16)-C(17)-C(12)	1.17(18)
C(13)-C(12)-C(17)-C(16)	0.02(18)
C(11)-C(12)-C(17)-C(16)	-177.92(11)
C(3)-C(2)-C(18)-C(19)	-142.11(13)
C(1)-C(2)-C(18)-C(19)	146.59(12)

Symmetry transformations used to generate equivalent atoms:

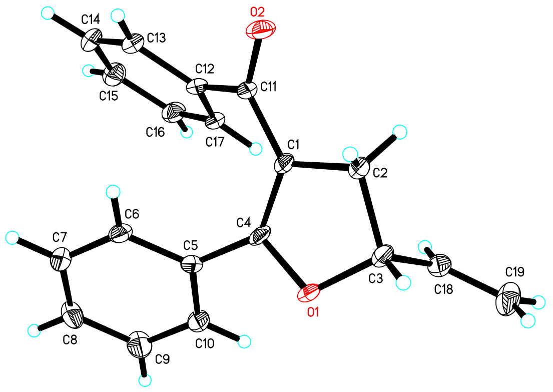

Figure S16: X-ray structure of compound **(R)-4**

Table 15. Crystal data and structure refinement for **(R)-4**.

Identification code	s2180lc
Empirical formula	C19 H16 O2
Formula weight	276.32
Temperature	100(2) K
Wavelength	1.54178 A
Crystal system, space group	Monoclinic, P 21
Unit cell dimensions	a = 6.3155(3) A alpha = 90 deg.
	b = 7.7433(4) A beta = 100.579(3)
	c = 15.0935(9) A gamma = 90 deg.

Volume	725.57(7) A^3
Z, Calculated density	2, 1.265 Mg/m^3
Absorption coefficient	0.641 mm^-1
F(000)	292
Crystal size	0.54 x 0.20 x 0.08 mm
Theta range for data collection	2.98 to 65.87 deg.
Limiting indices	-6<=h<=7, -8<=k<=9, -17<=l<=17
Reflections collected / unique	8588 / 2439 [R(int) = 0.0583]
Completeness to theta = 65.87	98.7 %
Absorption correction	Semi-empirical from equivalents
Max. and min. transmission	0.7528 and 0.5323
Refinement method	Full-matrix least-squares on F^2
Data / restraints / parameters	2439 / 1 / 191
Goodness-of-fit on F^2	1.043

Final R indices [I>2sigma(I)]   R1 = 0.0382, wR2 = 0.0971

R indices (all data)   R1 = 0.0386, wR2 = 0.0976

Absolute structure parameter   0.2(2)

Extinction coefficient   0.0060(16)

Largest diff. peak and hole   0.198 and -0.246 e.A^-3

Table 16. Atomic coordinates (x 10^4) and equivalent isotropic displacement parameters (A^2 x 10^3) for s2180lc. U(eq) is defined as one third of the trace of the orthogonalized Uij tensor.

	x	y	z	U(eq)
O(1)	5947(2)	3292(2)	4181(1)	18(1)
C(1)	3155(3)	4361(2)	3164(1)	15(1)
O(2)	-162(2)	5438(2)	2431(1)	22(1)
C(2)	3397(3)	5597(2)	3948(1)	18(1)
C(3)	5585(3)	5046(2)	4509(1)	18(1)
C(4)	4597(2)	3063(2)	3371(1)	16(1)
C(5)	4920(3)	1410(2)	2937(1)	16(1)
C(6)	3169(3)	413(2)	2536(1)	19(1)
C(7)	3479(3)	-1129(2)	2117(1)	21(1)
C(8)	5556(3)	-1704(2)	2108(1)	24(1)

C(9)	7312(3)	-743(3)	2520(1)	26(1)
C(10)	7004(3)	808(2)	2930(1)	21(1)
C(11)	1423(3)	4550(2)	2379(1)	16(1)
C(12)	1598(3)	3720(2)	1498(1)	17(1)
C(13)	-243(3)	2969(2)	994(1)	19(1)
C(14)	-154(3)	2200(2)	172(1)	23(1)
C(15)	1751(3)	2234(3)	-167(1)	23(1)
C(16)	3570(3)	3008(3)	322(1)	21(1)
C(17)	3514(3)	3723(2)	1161(1)	18(1)
C(18)	7429(3)	6141(2)	4363(1)	21(1)
C(19)	8672(3)	7004(3)	5014(2)	30(1)

Table 17. Bond lengths [A] and angles [deg] for s2180lc.

O(1)-C(4)	1.367(2)
O(1)-C(3)	1.478(2)
C(1)-C(4)	1.354(2)
C(1)-C(11)	1.465(2)
C(1)-C(2)	1.507(2)
O(2)-C(11)	1.229(2)
C(2)-C(3)	1.542(2)
C(2)-H(2A)	0.9900
C(2)-H(2B)	0.9900
C(3)-C(18)	1.489(2)
C(3)-H(3)	1.0000
C(4)-C(5)	1.469(2)

C(5)-C(6)	1.392(2)
C(5)-C(10)	1.398(2)
C(6)-C(7)	1.383(3)
C(6)-H(6)	0.9500
C(7)-C(8)	1.387(3)
C(7)-H(7)	0.9500
C(8)-C(9)	1.385(3)
C(8)-H(8)	0.9500
C(9)-C(10)	1.381(3)
C(9)-H(9)	0.9500
C(10)-H(10)	0.9500
C(11)-C(12)	1.499(2)
C(12)-C(13)	1.394(2)
C(12)-C(17)	1.396(2)
C(13)-C(14)	1.386(3)
C(13)-H(13)	0.9500
C(14)-C(15)	1.392(3)
C(14)-H(14)	0.9500
C(15)-C(16)	1.383(3)
C(15)-H(15)	0.9500
C(16)-C(17)	1.389(3)
C(16)-H(16)	0.9500
C(17)-H(17)	0.9500
C(18)-C(19)	1.321(3)
C(18)-H(18)	0.9500
C(19)-H(19A)	0.9500
C(19)-H(19B)	0.9500

C(4)-O(1)-C(3)	108.01(12)
C(4)-C(1)-C(11)	129.13(15)
C(4)-C(1)-C(2)	108.89(14)
C(11)-C(1)-C(2)	121.64(14)
C(1)-C(2)-C(3)	101.91(13)
C(1)-C(2)-H(2A)	111.4
C(3)-C(2)-H(2A)	111.4
C(1)-C(2)-H(2B)	111.4
C(3)-C(2)-H(2B)	111.4
H(2A)-C(2)-H(2B)	109.3
O(1)-C(3)-C(18)	107.50(13)
O(1)-C(3)-C(2)	104.38(13)
C(18)-C(3)-C(2)	114.07(15)
O(1)-C(3)-H(3)	110.2
C(18)-C(3)-H(3)	110.2
C(2)-C(3)-H(3)	110.2
C(1)-C(4)-O(1)	113.11(15)
C(1)-C(4)-C(5)	133.39(15)
O(1)-C(4)-C(5)	113.35(14)
C(6)-C(5)-C(10)	119.05(16)
C(6)-C(5)-C(4)	120.82(15)
C(10)-C(5)-C(4)	120.13(15)
C(7)-C(6)-C(5)	120.64(15)
C(7)-C(6)-H(6)	119.7
C(5)-C(6)-H(6)	119.7
C(6)-C(7)-C(8)	119.71(16)
C(6)-C(7)-H(7)	120.1
C(8)-C(7)-H(7)	120.1

C(9)-C(8)-C(7)	120.23(17)
C(9)-C(8)-H(8)	119.9
C(7)-C(8)-H(8)	119.9
C(10)-C(9)-C(8)	120.11(16)
C(10)-C(9)-H(9)	119.9
C(8)-C(9)-H(9)	119.9
C(9)-C(10)-C(5)	120.24(17)
C(9)-C(10)-H(10)	119.9
C(5)-C(10)-H(10)	119.9
O(2)-C(11)-C(1)	120.00(15)
O(2)-C(11)-C(12)	119.35(14)
C(1)-C(11)-C(12)	120.62(14)
C(13)-C(12)-C(17)	119.60(15)
C(13)-C(12)-C(11)	118.23(14)
C(17)-C(12)-C(11)	122.15(14)
C(14)-C(13)-C(12)	120.04(16)
C(14)-C(13)-H(13)	120.0
C(12)-C(13)-H(13)	120.0
C(13)-C(14)-C(15)	120.04(16)
C(13)-C(14)-H(14)	120.0
C(15)-C(14)-H(14)	120.0
C(16)-C(15)-C(14)	120.12(16)
C(16)-C(15)-H(15)	119.9
C(14)-C(15)-H(15)	119.9
C(15)-C(16)-C(17)	120.10(15)
C(15)-C(16)-H(16)	120.0
C(17)-C(16)-H(16)	120.0
C(16)-C(17)-C(12)	120.03(15)

C(16)-C(17)-H(17)	120.0
C(12)-C(17)-H(17)	120.0
C(19)-C(18)-C(3)	123.55(18)
C(19)-C(18)-H(18)	118.2
C(3)-C(18)-H(18)	118.2
C(18)-C(19)-H(19A)	120.0
C(18)-C(19)-H(19B)	120.0
H(19A)-C(19)-H(19B)	120.0

Symmetry transformations used to generate equivalent atoms:

Table 18. Anisotropic displacement parameters (A^2 x 10^3) for s2180lc. The anisotropic displacement factor exponent takes the form: -2 pi^2 [h^2 a*^2 U11 + ... + 2 h k a* b* U12]

	U11	U22	U33	U23	U13	U12
O(1)	13(1)	19(1)	18(1)	-2(1)	-7(1)	3(1)
C(1)	10(1)	16(1)	18(1)	-3(1)	-1(1)	-1(1)
O(2)	14(1)	26(1)	25(1)	-2(1)	-3(1)	7(1)
C(2)	14(1)	17(1)	20(1)	-4(1)	-2(1)	1(1)
C(3)	15(1)	20(1)	17(1)	-4(1)	-4(1)	0(1)
C(4)	8(1)	20(1)	17(1)	1(1)	-3(1)	-3(1)
C(5)	14(1)	15(1)	17(1)	3(1)	-1(1)	2(1)
C(6)	12(1)	20(1)	22(1)	5(1)	-2(1)	-1(1)

C(7)	20(1)	18(1)	23(1)	1(1)	-1(1)	-4(1)
C(8)	28(1)	15(1)	30(1)	-2(1)	7(1)	0(1)
C(9)	16(1)	22(1)	42(1)	-3(1)	8(1)	3(1)
C(10)	12(1)	19(1)	31(1)	-1(1)	-1(1)	0(1)
C(11)	10(1)	13(1)	21(1)	1(1)	-3(1)	0(1)
C(12)	12(1)	16(1)	19(1)	1(1)	-4(1)	3(1)
C(13)	12(1)	19(1)	23(1)	0(1)	-2(1)	0(1)
C(14)	18(1)	23(1)	23(1)	-4(1)	-6(1)	-3(1)
C(15)	23(1)	25(1)	20(1)	-3(1)	-1(1)	-1(1)
C(16)	15(1)	24(1)	24(1)	-1(1)	2(1)	2(1)
C(17)	12(1)	16(1)	22(1)	1(1)	-4(1)	0(1)
C(18)	14(1)	19(1)	27(1)	-3(1)	-1(1)	2(1)
C(19)	13(1)	32(1)	43(1)	-16(1)	2(1)	-2(1)

Table 19. Hydrogen coordinates (x 10^4) and isotropic displacement parameters (A^2 x 10^3) for s2180lc.

	x	y	z	U(eq)
H(2A)	2218	5453	4294	21
H(2B)	3429	6811	3745	21
H(3)	5502	5011	5164	22
H(6)	1748	798	2551	22
H(7)	2276	-1793	1837	25

H(8)	5774	-2761	1818	29
H(9)	8731	-1151	2521	31
H(10)	8212	1468	3208	25
H(13)	-1559	2984	1214	22
H(14)	-1395	1649	-160	27
H(15)	1802	1725	-735	28
H(16)	4861	3051	83	25
H(17)	4781	4214	1506	21
H(18)	7727	6224	3770	25
H(19A)	8411	6945	5613	36
H(19B)	9828	7684	4883	36

Table 20. Torsion angles [deg] for s2180lc.

C(4)-C(1)-C(2)-C(3)	14.66(17)
C(11)-C(1)-C(2)-C(3)	-171.48(15)
C(4)-O(1)-C(3)-C(18)	-104.38(15)
C(4)-O(1)-C(3)-C(2)	17.11(16)
C(1)-C(2)-C(3)-O(1)	-18.63(16)
C(1)-C(2)-C(3)-C(18)	98.41(16)
C(11)-C(1)-C(4)-O(1)	-177.88(16)
C(2)-C(1)-C(4)-O(1)	-4.62(18)
C(11)-C(1)-C(4)-C(5)	-2.7(3)
C(2)-C(1)-C(4)-C(5)	170.54(17)
C(3)-O(1)-C(4)-C(1)	-8.32(18)
C(3)-O(1)-C(4)-C(5)	175.51(12)

C(1)-C(4)-C(5)-C(6)	-38.6(3)
O(1)-C(4)-C(5)-C(6)	136.56(15)
C(1)-C(4)-C(5)-C(10)	141.99(19)
O(1)-C(4)-C(5)-C(10)	-42.9(2)
C(10)-C(5)-C(6)-C(7)	-1.5(2)
C(4)-C(5)-C(6)-C(7)	179.04(15)
C(5)-C(6)-C(7)-C(8)	1.0(3)
C(6)-C(7)-C(8)-C(9)	0.3(3)
C(7)-C(8)-C(9)-C(10)	-1.1(3)
C(8)-C(9)-C(10)-C(5)	0.5(3)
C(6)-C(5)-C(10)-C(9)	0.8(3)
C(4)-C(5)-C(10)-C(9)	-179.78(16)
C(4)-C(1)-C(11)-O(2)	154.56(17)
C(2)-C(1)-C(11)-O(2)	-17.9(3)
C(4)-C(1)-C(11)-C(12)	-27.6(3)
C(2)-C(1)-C(11)-C(12)	159.87(15)
O(2)-C(11)-C(12)-C(13)	-42.0(2)
C(1)-C(11)-C(12)-C(13)	140.22(16)
O(2)-C(11)-C(12)-C(17)	136.18(17)
C(1)-C(11)-C(12)-C(17)	-41.6(2)
C(17)-C(12)-C(13)-C(14)	1.3(3)
C(11)-C(12)-C(13)-C(14)	179.49(16)
C(12)-C(13)-C(14)-C(15)	-2.4(3)
C(13)-C(14)-C(15)-C(16)	1.2(3)
C(14)-C(15)-C(16)-C(17)	1.3(3)
C(15)-C(16)-C(17)-C(12)	-2.4(3)
C(13)-C(12)-C(17)-C(16)	1.1(2)
C(11)-C(12)-C(17)-C(16)	-176.98(16)

O(1)-C(3)-C(18)-C(19)	-123.66(19)
C(2)-C(3)-C(18)-C(19)	121.1(2)

Symmetry transformations used to generate equivalent atoms:

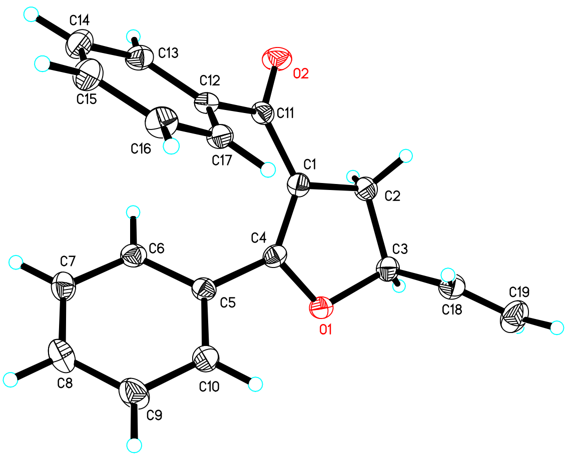

Figure S17: X-ray structure of compound **(S)-4**

Table 21. Crystal data and structure refinement for **(S)-4**.

Identification code	s2182lc
Empirical formula	C19 H16 O2
Formula weight	276.32
Temperature	100(2) K
Wavelength	1.54178 A
Crystal system, space group	Monoclinic, P 21
Unit cell dimensions	a = 6.309(2) A alpha = 90 deg.
	b = 7.753(2) A beta = 100.736(14)
	c = 15.117(5) A gamma = 90 deg.

Volume	726.5(4) A^3
Z, Calculated density	2, 1.263 Mg/m^3
Absorption coefficient	0.640 mm^-1
F(000)	292
Crystal size	0.41 x 0.37 x 0.31 mm
Theta range for data collection	2.98 to 65.60 deg.
Limiting indices	-4<=h<=7, -9<=k<=9, -17<=l<=16
Reflections collected / unique	8149 / 2402 [R(int) = 0.0335]
Completeness to theta = 65.60	96.6 %
Absorption correction	Semi-empirical from equivalents
Max. and min. transmission	0.7528 and 0.6318
Refinement method	Full-matrix least-squares on F^2
Data / restraints / parameters	2402 / 1 / 191
Goodness-of-fit on F^2	1.014

Final R indices [I>2sigma(I)]   R1 = 0.0234, wR2 = 0.0573

R indices (all data)   R1 = 0.0237, wR2 = 0.0576

Absolute structure parameter   -0.03(16)

Extinction coefficient   0.0085(7)

Largest diff. peak and hole   0.112 and -0.133 e.A^-3

Table 22. Atomic coordinates (x 10^4) and equivalent isotropic displacement parameters (A^2 x 10^3) for s2182lc. U(eq) is defined as one third of the trace of the orthogonalized Uij tensor.

	x	y	z	U(eq)
O(1)	4054(1)	6709(1)	5822(1)	20(1)
C(1)	6846(2)	5641(2)	6839(1)	18(1)
O(2)	10165(1)	4563(1)	7568(1)	25(1)
C(2)	6601(2)	4406(2)	6050(1)	20(1)
C(3)	4420(2)	4950(2)	5495(1)	20(1)
C(4)	5397(2)	6933(2)	6627(1)	17(1)
C(5)	5073(2)	8589(2)	7065(1)	18(1)
C(6)	6831(2)	9587(2)	7464(1)	20(1)
C(7)	6520(2)	11131(2)	7884(1)	22(1)

C(8)	4446(2)	11706(2)	7893(1)	26(1)
C(9)	2685(2)	10747(2)	7483(1)	28(1)
C(10)	2996(2)	9190(2)	7068(1)	23(1)
C(11)	8566(2)	5453(2)	7621(1)	18(1)
C(12)	8397(2)	6281(2)	8502(1)	17(1)
C(13)	10238(2)	7028(2)	9007(1)	21(1)
C(14)	10150(2)	7799(2)	9827(1)	24(1)
C(15)	8252(2)	7763(2)	10168(1)	25(1)
C(16)	6428(2)	6988(2)	9678(1)	23(1)
C(17)	6487(2)	6274(2)	8840(1)	19(1)
C(18)	2567(2)	3859(2)	5633(1)	23(1)
C(19)	1331(2)	3001(2)	4986(1)	32(1)

Table 23. Bond lengths [A] and angles [deg] for s2182lc.

O(1)-C(4)	1.3586(15)
O(1)-C(3)	1.4835(15)
C(1)-C(4)	1.3540(18)
C(1)-C(11)	1.4553(18)
C(1)-C(2)	1.5143(17)
O(2)-C(11)	1.2373(16)
C(2)-C(3)	1.5312(18)
C(2)-H(2A)	0.9900
C(2)-H(2B)	0.9900
C(3)-C(18)	1.4894(19)
C(3)-H(3)	1.0000

C(4)-C(5)	1.4758(18)
C(5)-C(10)	1.3921(19)
C(5)-C(6)	1.3942(18)
C(6)-C(7)	1.386(2)
C(6)-H(6)	0.9500
C(7)-C(8)	1.385(2)
C(7)-H(7)	0.9500
C(8)-C(9)	1.384(2)
C(8)-H(8)	0.9500
C(9)-C(10)	1.391(2)
C(9)-H(9)	0.9500
C(10)-H(10)	0.9500
C(11)-C(12)	1.4999(17)
C(12)-C(13)	1.3922(18)
C(12)-C(17)	1.3936(18)
C(13)-C(14)	1.3861(19)
C(13)-H(13)	0.9500
C(14)-C(15)	1.390(2)
C(14)-H(14)	0.9500
C(15)-C(16)	1.385(2)
C(15)-H(15)	0.9500
C(16)-C(17)	1.3888(19)
C(16)-H(16)	0.9500
C(17)-H(17)	0.9500
C(18)-C(19)	1.313(2)
C(18)-H(18)	0.9500
C(19)-H(19A)	0.9500
C(19)-H(19B)	0.9500

C(4)-O(1)-C(3)	107.83(9)
C(4)-C(1)-C(11)	129.35(11)
C(4)-C(1)-C(2)	108.43(11)
C(11)-C(1)-C(2)	121.88(11)
C(1)-C(2)-C(3)	101.96(10)
C(1)-C(2)-H(2A)	111.4
C(3)-C(2)-H(2A)	111.4
C(1)-C(2)-H(2B)	111.4
C(3)-C(2)-H(2B)	111.4
H(2A)-C(2)-H(2B)	109.2
O(1)-C(3)-C(18)	107.46(10)
O(1)-C(3)-C(2)	104.49(10)
C(18)-C(3)-C(2)	114.54(11)
O(1)-C(3)-H(3)	110.0
C(18)-C(3)-H(3)	110.0
C(2)-C(3)-H(3)	110.0
C(1)-C(4)-O(1)	113.46(11)
C(1)-C(4)-C(5)	132.97(11)
O(1)-C(4)-C(5)	113.42(10)
C(10)-C(5)-C(6)	119.09(12)
C(10)-C(5)-C(4)	120.13(11)
C(6)-C(5)-C(4)	120.76(11)
C(7)-C(6)-C(5)	120.56(12)
C(7)-C(6)-H(6)	119.7
C(5)-C(6)-H(6)	119.7
C(8)-C(7)-C(6)	119.81(12)
C(8)-C(7)-H(7)	120.1

C(6)-C(7)-H(7)	120.1
C(9)-C(8)-C(7)	120.26(13)
C(9)-C(8)-H(8)	119.9
C(7)-C(8)-H(8)	119.9
C(8)-C(9)-C(10)	119.96(13)
C(8)-C(9)-H(9)	120.0
C(10)-C(9)-H(9)	120.0
C(9)-C(10)-C(5)	120.28(12)
C(9)-C(10)-H(10)	119.9
C(5)-C(10)-H(10)	119.9
O(2)-C(11)-C(1)	119.80(11)
O(2)-C(11)-C(12)	119.29(11)
C(1)-C(11)-C(12)	120.89(11)
C(13)-C(12)-C(17)	119.35(11)
C(13)-C(12)-C(11)	118.38(11)
C(17)-C(12)-C(11)	122.23(11)
C(14)-C(13)-C(12)	120.12(12)
C(14)-C(13)-H(13)	119.9
C(12)-C(13)-H(13)	119.9
C(13)-C(14)-C(15)	120.26(12)
C(13)-C(14)-H(14)	119.9
C(15)-C(14)-H(14)	119.9
C(16)-C(15)-C(14)	119.83(12)
C(16)-C(15)-H(15)	120.1
C(14)-C(15)-H(15)	120.1
C(15)-C(16)-C(17)	120.03(12)
C(15)-C(16)-H(16)	120.0
C(17)-C(16)-H(16)	120.0

C(16)-C(17)-C(12)	120.33(12)
C(16)-C(17)-H(17)	119.8
C(12)-C(17)-H(17)	119.8
C(19)-C(18)-C(3)	123.75(13)
C(19)-C(18)-H(18)	118.1
C(3)-C(18)-H(18)	118.1
C(18)-C(19)-H(19A)	120.0
C(18)-C(19)-H(19B)	120.0
H(19A)-C(19)-H(19B)	120.0

Symmetry transformations used to generate equivalent atoms:

Table 24. Anisotropic displacement parameters (A^2 x 10^3) for s2182lc. The anisotropic displacement factor exponent takes the form: -2 pi^2 [h^2 a*^2 U11 + ... + 2 h k a* b* U12]

	U11	U22	U33	U23	U13	U12
O(1)	20(1)	19(1)	20(1)	-2(1)	0(1)	3(1)
C(1)	17(1)	18(1)	19(1)	-2(1)	5(1)	0(1)
O(2)	19(1)	28(1)	26(1)	-2(1)	3(1)	8(1)
C(2)	20(1)	19(1)	21(1)	-3(1)	4(1)	2(1)
C(3)	22(1)	21(1)	17(1)	-4(1)	2(1)	0(1)
C(4)	14(1)	20(1)	18(1)	1(1)	4(1)	-2(1)
C(5)	19(1)	17(1)	17(1)	1(1)	3(1)	1(1)

C(6)	18(1)	20(1)	22(1)	4(1)	3(1)	0(1)
C(7)	25(1)	18(1)	24(1)	0(1)	5(1)	-4(1)
C(8)	32(1)	17(1)	31(1)	-2(1)	13(1)	-1(1)
C(9)	23(1)	23(1)	42(1)	-4(1)	13(1)	2(1)
C(10)	18(1)	21(1)	31(1)	-2(1)	4(1)	0(1)
C(11)	17(1)	15(1)	22(1)	1(1)	4(1)	0(1)
C(12)	17(1)	17(1)	17(1)	2(1)	1(1)	2(1)
C(13)	17(1)	23(1)	22(1)	2(1)	3(1)	1(1)
C(14)	22(1)	26(1)	22(1)	-2(1)	0(1)	-3(1)
C(15)	29(1)	27(1)	18(1)	-3(1)	3(1)	0(1)
C(16)	20(1)	27(1)	24(1)	0(1)	8(1)	1(1)
C(17)	17(1)	19(1)	20(1)	2(1)	1(1)	0(1)
C(18)	19(1)	24(1)	27(1)	-3(1)	5(1)	2(1)
C(19)	20(1)	36(1)	41(1)	-15(1)	7(1)	-2(1)

Table 25. Hydrogen coordinates (x 10^4) and isotropic displacement parameters (A^2 x 10^3) for s2182lc.

	x	y	z	U(eq)
H(2A)	6573	3192	6250	24
H(2B)	7777	4555	5705	24
H(3)	4503	4987	4841	24
H(6)	8254	9206	7447	24

H(7)	7726	11793	8165	27
H(8)	4231	12762	8182	31
H(9)	1265	11152	7484	34
H(10)	1785	8534	6786	28
H(13)	11557	7009	8790	25
H(14)	11392	8354	10157	29
H(15)	8206	8268	10737	30
H(16)	5136	6944	9914	28
H(17)	5220	5779	8496	23
H(18)	2261	3780	6224	28
H(19A)	1598	3056	4389	39
H(19B)	170	2325	5115	39

Table 26. Torsion angles [deg] for s2182lc.

C(4)-C(1)-C(2)-C(3)	-14.87(13)
C(11)-C(1)-C(2)-C(3)	171.19(12)
C(4)-O(1)-C(3)-C(18)	104.62(11)
C(4)-O(1)-C(3)-C(2)	-17.45(12)
C(1)-C(2)-C(3)-O(1)	18.92(12)
C(1)-C(2)-C(3)-C(18)	-98.37(12)
C(11)-C(1)-C(4)-O(1)	177.93(12)
C(2)-C(1)-C(4)-O(1)	4.59(14)
C(11)-C(1)-C(4)-C(5)	2.7(2)
C(2)-C(1)-C(4)-C(5)	-170.64(12)

C(3)-O(1)-C(4)-C(1)	8.45(13)
C(3)-O(1)-C(4)-C(5)	-175.36(10)
C(1)-C(4)-C(5)-C(10)	-142.14(14)
O(1)-C(4)-C(5)-C(10)	42.64(16)
C(1)-C(4)-C(5)-C(6)	39.1(2)
O(1)-C(4)-C(5)-C(6)	-136.11(11)
C(10)-C(5)-C(6)-C(7)	2.06(18)
C(4)-C(5)-C(6)-C(7)	-179.19(11)
C(5)-C(6)-C(7)-C(8)	-1.27(19)
C(6)-C(7)-C(8)-C(9)	-0.2(2)
C(7)-C(8)-C(9)-C(10)	0.8(2)
C(8)-C(9)-C(10)-C(5)	0.1(2)
C(6)-C(5)-C(10)-C(9)	-1.45(19)
C(4)-C(5)-C(10)-C(9)	179.79(12)
C(4)-C(1)-C(11)-O(2)	-154.48(13)
C(2)-C(1)-C(11)-O(2)	18.08(19)
C(4)-C(1)-C(11)-C(12)	27.5(2)
C(2)-C(1)-C(11)-C(12)	-159.98(11)
O(2)-C(11)-C(12)-C(13)	41.75(17)
C(1)-C(11)-C(12)-C(13)	-140.18(12)
O(2)-C(11)-C(12)-C(17)	-136.11(13)
C(1)-C(11)-C(12)-C(17)	41.96(18)
C(17)-C(12)-C(13)-C(14)	-1.81(18)
C(11)-C(12)-C(13)-C(14)	-179.73(12)
C(12)-C(13)-C(14)-C(15)	2.9(2)
C(13)-C(14)-C(15)-C(16)	-1.5(2)
C(14)-C(15)-C(16)-C(17)	-1.1(2)
C(15)-C(16)-C(17)-C(12)	2.2(2)

C(13)-C(12)-C(17)-C(16)	-0.76(18)
C(11)-C(12)-C(17)-C(16)	177.08(12)
O(1)-C(3)-C(18)-C(19)	123.69(14)
C(2)-C(3)-C(18)-C(19)	-120.72(15)

Symmetry transformations used to generate equivalent atoms:

12. Computed Structures and Energies

```
** All coordinates in atomic units (1 Bohr = 52.91772 pm) **
** Energies in atomic units (1 Hartree = 27.2114 eV = 2625.5 kJ/mol) **
** unless noted otherwise **

DFT equilibrium structures
--------------------------

Functional: PBE
Basis set:  def2-TZVPP' (def2-TZVPP with g function deleted)
Standard MOLPRO 2012.1 settings for grid and convergence, no density
fitting

== DFT(PBE) S0 ==
!RKS STATE 1.1 Energy              -1733.360499612880

   1  FE     26.00    0.000919967    0.000000000    0.377887191
   2  N       7.00    0.000616235    0.000000000    3.515450770
   3  O       8.00   -0.000243338    0.000000000    5.774469393
   4  C       6.00    2.998552204    0.000000000   -1.129057268
   5  O       8.00    5.000998269    0.000000000   -2.085985030
   6  C       6.00   -1.498998999   -2.595198162   -1.129280170
   7  C       6.00   -1.498998999    2.595198162   -1.129280170
   8  O       8.00   -2.501423614   -4.328421822   -2.086665401
   9  O       8.00   -2.501423614    4.328421822   -2.086665401

with COSMO: eps=2.5 / f(eps)=0.5
!RKS STATE 1.1 Energy              -1733.396901697604
outlying-charge corrected          -1733.3988297691

   1  FE     26.00    0.439264784   -0.000000000    0.508335874
   2  N       7.00   -0.069957393   -0.000000000    3.649094114
   3  O       8.00   -0.832651617    0.000000000    5.718454654
   4  C       6.00    3.377209198   -0.000000000   -1.398629183
   5  O       8.00    5.150074396    0.000000000   -2.662399212
   6  C       6.00   -1.411618442   -2.403750972   -0.986843255
   7  C       6.00   -1.411618442    2.403750972   -0.986843255
   8  O       8.00   -2.620352187   -3.971228390   -1.910147911
   9  O       8.00   -2.620352187    3.971228390   -1.910147911

with COSMO: eps=infty / f(eps)=1.0
!RKS STATE 1.1 Energy              -1733.433448818465
outlying-charge corrected          -1733.4372900406

   1  FE     26.00    0.000870345    0.000000000    0.387417122
   2  N       7.00    0.000481016    0.000000000    3.521243393
   3  O       8.00   -0.000452780   -0.000000000    5.779415725
   4  C       6.00    2.990223790   -0.000000000   -1.127942718
   5  O       8.00    4.983793759   -0.000000000   -2.094188233
   6  C       6.00   -1.494634868   -2.588193801   -1.127981538
   7  C       6.00   -1.494634868    2.588193801   -1.127981538
   8  O       8.00   -2.492824141   -4.313745485   -2.094554148
   9  O       8.00   -2.492824141    4.313745485   -2.094554148

== DFT(PBE) T1 ==
!RKS STATE 1.1 Energy              -1733.299110756056

   1  FE     26.00   -0.092964634    0.000000000    0.417659140
   2  N       7.00    0.567562711    0.000000000    3.746053097
```

```
   3  O       8.00   -0.672857877    0.000000000    5.678530812
   4  C       6.00    2.896872518    0.000000000   -1.174975631
   5  O       8.00    4.719171879    0.000000000   -2.443862833
   6  C       6.00   -1.431390152   -2.722960968   -1.052061424
   7  C       6.00   -1.431390152    2.722960968   -1.052061424
   8  O       8.00   -2.277503091   -4.517936300   -2.049203911
   9  O       8.00   -2.277503091    4.517936300   -2.049203911


== DFT(PBE) T2 ==
!RKS STATE 1.2 Energy              -1733.282036224798

   1  FE     26.00    0.071080246    0.000000000    0.377628746
   2  N       7.00   -0.312563037    0.000000000    3.804117604
   3  O       8.00    0.733964904    0.000000000    5.831574266
   4  C       6.00    3.254489035    0.000000000   -0.770088349
   5  O       8.00    5.173266099    0.000000000   -1.895200958
   6  C       6.00   -1.680740369   -2.311044474   -1.259937137
   7  C       6.00   -1.680740369    2.311044474   -1.259937137
   8  O       8.00   -2.779379199   -3.874115119   -2.403641560
   9  O       8.00   -2.779379199    3.874115119   -2.403641560


== DFT(PBE) D0 ==
!RKS STATE 1.2 Energy              -1733.246849621230

   1  FE     26.00    0.437938693    0.000000000    0.505366761
   2  N       7.00   -0.068428284    0.000000000    3.646486387
   3  O       8.00   -0.825343686    0.000000000    5.718036106
   4  C       6.00    3.380006955    0.000000000   -1.396974805
   5  O       8.00    5.157097646    0.000000000   -2.654107868
   6  C       6.00   -1.416123789   -2.406115239   -0.989169617
   7  C       6.00   -1.416123789    2.406115239   -0.989169617
   8  O       8.00   -2.624512818   -3.973987556   -1.909796716
   9  O       8.00   -2.624512818    3.973987556   -1.909796716

with COSMO: eps=2.5 / f(eps)=0.5
!RKS STATE 1.1 Energy              -1733.248143038234
outlying-charge corrected          -1733.2480858666

   1  FE     26.00    0.439264784   -0.000000000    0.508335874
   2  N       7.00   -0.069957393   -0.000000000    3.649094114
   3  O       8.00   -0.832651617    0.000000000    5.718454654
   4  C       6.00    3.377209198   -0.000000000   -1.398629183
   5  O       8.00    5.150074396    0.000000000   -2.662399212
   6  C       6.00   -1.411618442   -2.403750972   -0.986843255
   7  C       6.00   -1.411618442    2.403750972   -0.986843255
   8  O       8.00   -2.620352187   -3.971228390   -1.910147911
   9  O       8.00   -2.620352187    3.971228390   -1.910147911

with COSMO: eps=infty / f(eps)=1.0
!RKS STATE 1.1 Energy              -1733.249664594353
outlying-charge corrected          -1733.2495476369

   1  FE     26.00    0.438266310   -0.000000000    0.507709324
   2  N       7.00   -0.068450687   -0.000000000    3.649159111
   3  O       8.00   -0.832854601    0.000000000    5.717662314
   4  C       6.00    3.375037243    0.000000000   -1.397755513
   5  O       8.00    5.148288647   -0.000000000   -2.661863355
   6  C       6.00   -1.410140809   -2.400551319   -0.986372688
```

```
   7  C       6.00   -1.410140809    2.400551319   -0.986372688
   8  O       8.00   -2.620003592   -3.968692711   -1.910646295
   9  O       8.00   -2.620003592    3.968692711   -1.910646295
```

With functional PBE0
Basis set: def2-TZVPP' (def2-TZVPP with g function deleted)
Standard MOLPRO 2012.1 settings for grid and convergence, no density fitting

```
== DFT(PBE0) S0 ==
!RKS STATE 1.1 Energy             -1733.231908495653

   1  FE     26.00    0.000870217    0.000000000    0.380884496
   2  N       7.00    0.000548642    0.000000000    3.457509662
   3  O       8.00   -0.000246558    0.000000000    5.684726717
   4  C       6.00    2.973100491    0.000000000   -1.112498206
   5  O       8.00    4.946003263    0.000000000   -2.054371884
   6  C       6.00   -1.486184264   -2.573277054   -1.112677897
   7  C       6.00   -1.486184264    2.573277054   -1.112677897
   8  O       8.00   -2.473954709   -4.280859918   -2.055010538
   9  O       8.00   -2.473954709    4.280859918   -2.055010538

== DFT(PBE0) D0 ==
!RKS STATE 1.2 Energy             -1733.130691912380

   1  FE     26.00    0.452009029    0.000000000    0.530957643
   2  N       7.00   -0.128395527    0.000000000    3.597721730
   3  O       8.00   -0.964670470    0.000000000    5.601681624
   4  C       6.00    3.340337968    0.000000000   -1.460985099
   5  O       8.00    5.057802539    0.000000000   -2.743112007
   6  C       6.00   -1.367564966   -2.499930961   -0.935308307
   7  C       6.00   -1.367564966    2.499930961   -0.935308307
   8  O       8.00   -2.510977749   -4.089440877   -1.817386680
   9  O       8.00   -2.510977749    4.089440877   -1.817386680
```

Summary of main bond distances (Aangstroem units) and angles (degrees)

PBE

	S0	T1	T2	D0
E(rlx)[a]	---	-0.87	-0.72	-0.36
Fe-N	1.66	1.80	1.82	1.68
N-O	1.20	1.22	1.21	1.17
Fe-C	1.78	1.79	1.79	1.85
[b]		1.78	1.76	1.79
C-O	1.17	1.18	1.18	1.15
[b]		1.18	1.18	1.16
Fe-N-O	180.0	136.1	146.3	169.1
N-Fe-C	116.7	106.8	116.2	132.0
[b]		120.3	115.5	110.4

[a] Relaxation energy relative to vertical excitation
[b] Cs symmetric structure: First row is bond (bond angle) in symmetry plane, second row pair of bonds (bond angles) that are mirror images of each other

PBE0

	S0	D0
Fe-N	1.63	1.65

N-O	1.18	1.15
Fe-C	1.76	1.86
		1.81
C-O	1.16	1.15
		1.13
Fe-N-O	180.0	168.1
N-Fe-C	116.7	135.3

CASSCF:

	S0	S1	T1	T2	D0
E(rlx)[a]	---	-1.00	-0.76	-0.82	-1.07
Fe-N	1.68	1.92	1.88	1.88	1.72
N-O	1.18	1.21	1.20	1.20	1.18
Fe-C	1.87	2.13	1.97	2.12	2.22
[b]		2.04	2.05	2.03	2.18
C-O	1.12	1.11	1.14	1.11	1.10
[b]		1.13	1.12	1.13	1.10
Fe-N-O	180.0	144.0	179.7	151.7	178.1
N-Fe-C	116.3	101.4	153.1	98.1	126.0
[b]		126.8	105.3	126.9	117.6

[a],[b] see above (DFT/PBE table)

MRCI calculations based on PBE structures

Active space is always 14 electrons in 9 orbitals (5 in a', 4 in a")
CASSCF uses state-averaging over 5 states (singlets), or 4 states (triplets and neutral doublets)

S0 energies at PBE S0 structure
Q: Davidson correction
P: Pople correction

MRCI	-1731.428254
MRCI+Q	-1731.808903
MRCI+P	-1731.909357

Vertical singlet excitation energies in eV

state	CASCI	MRCI	MRCI+Q	MRCI+P
2 a'	3.19	3.40	3.51	3.57
3 a'	3.22	3.48	3.64	3.72
4 a'	4.41	4.27	4.15	4.08
5 a'	4.87	4.82	4.75	4.71
1 a"	2.78	3.07	3.26	3.35
2 a"	3.17	3.41	3.55	3.62
3 a"	4.41	4.28	4.17	4.11
4 a"	4.24	4.31	4.34	4.36

Vertical triplet excitation energies in eV

state	CASCI	MRCI	MRCI+Q	MRCI+P
1 a'	1.76	2.12	2.32	2.46
2 a'	2.44	2.76	2.94	3.07

```
3 a'     3.31     3.64     3.79     3.89
4 a'     3.54     3.85     3.98     4.08
5 a'     4.95     5.41     5.69     5.87
1 a"     2.44     2.77     2.96     3.09
2 a"     2.56     2.94     3.15     3.29
3 a"     3.54     3.85     4.00     4.10
4 a"     3.85     4.17     4.31     4.42

D0 energies at PBE S0 structure

MRCI   -1731.359731
MRCI+Q -1731.719366
MRCI+P -1731.809112

D0 energies at PBE D0 structure

MRCI  -1731.389292
MRCI+Q  -1731.744156
MRCI+P  -1731.831563


CASSCF equlibrium structures
----------------------------
Active space is always 14 electrons in 9 orbitals (5 in a', 4 in a")
State-averaging over all states for which energies are quoted
(opt) indicates state for which geometry optimization was run

== CASSCF S0 ==
!MCSCF STATE 1.1 Energy            -1730.015514833774 (opt)
!MCSCF STATE 2.1 Energy            -1729.918912514030
!MCSCF STATE 3.1 Energy            -1729.918176160312
!MCSCF STATE 1.2 Energy            -1729.936200784939
!MCSCF STATE 2.2 Energy            -1729.918179504321

   1  FE     26.00   -0.000005123    0.000000000    0.363402310
   2  N       7.00    0.000062883    0.000000000    3.538006347
   3  O       8.00    0.000097210    0.000000000    5.776565653
   4  C       6.00    3.173351063    0.000000000   -1.205681297
   5  O       8.00    5.137812993    0.000000000   -2.013192464
   6  C       6.00   -1.586721153   -2.748162247   -1.205743496
   7  C       6.00   -1.586721153    2.748162247   -1.205743496
   8  O       8.00   -2.568939306   -4.449389851   -2.013369821
   9  O       8.00   -2.568939306    4.449389851   -2.013369821


== CASSCF S1 ==
!MCSCF STATE 1.1 Energy            -1729.982944111080
!MCSCF STATE 2.1 Energy            -1729.946780947037
!MCSCF STATE 3.1 Energy            -1729.920384919964
!MCSCF STATE 1.2 Energy            -1729.972853807584  (opt)
!MCSCF STATE 2.2 Energy            -1729.935905975993

   1  FE     26.00   -0.195740782    0.000000000    0.564863223
   2  N       7.00    0.008030046    0.000000000    4.188436498
   3  O       8.00    1.452623545    0.000000000    5.960390854
   4  C       6.00    3.692569366    0.000000000   -0.449973682
   5  O       8.00    5.721177180    0.000000000   -0.988770543
   6  C       6.00   -2.150169370   -2.489920483   -1.635673302
   7  C       6.00   -2.150169370    2.489920483   -1.635673302
   8  O       8.00   -3.189161253   -3.775594803   -2.991362916
```

```
   9  O       8.00   -3.189161253    3.775594803   -2.991362916

state energies with state-averaging over both singlet and triplet:
 !MCSCF STATE 1.1 Energy            -1729.985935371660  (S0)
 !MCSCF STATE 2.1 Energy            -1729.943104258666
 !MCSCF STATE 3.1 Energy            -1729.918249447044
 !MCSCF STATE 1.2 Energy            -1729.974099986737  (S1)
 !MCSCF STATE 2.2 Energy            -1729.934153143957
 !MCSCF STATE 1.1 Energy            -1729.982761065275  (T1)
 !MCSCF STATE 2.1 Energy            -1729.947850926034
 !MCSCF STATE 1.2 Energy            -1729.973100916846  (T2)
 !MCSCF STATE 2.2 Energy            -1729.949894592875


== CASSCF T1 ==
!MCSCF STATE 1.1 Energy            -1729.990020295988 (opt)
!MCSCF STATE 2.1 Energy            -1729.962349845576
!MCSCF STATE 1.2 Energy            -1729.965227444419
!MCSCF STATE 2.2 Energy            -1729.962354411238

   1  FE     26.00   -0.000131315    0.000000000    0.760092391
   2  N       7.00    0.000654561    0.000000000    4.354400854
   3  O       8.00    0.000141944    0.000000000    6.630012520
   4  C       6.00    3.198874485    0.000000000   -1.325738450
   5  O       8.00    4.914171145    0.000000000   -2.581479216
   6  C       6.00   -1.599595710   -2.770077783   -1.326087426
   7  C       6.00   -1.599595710    2.770077783   -1.326087426
   8  O       8.00   -2.457260645   -4.255341574   -2.582119666
   9  O       8.00   -2.457260645    4.255341574   -2.582119666


== CASSCF T2 ==
!MCSCF STATE 1.1 Energy            -1729.985645768347
!MCSCF STATE 2.1 Energy            -1729.950754330550
!MCSCF STATE 1.2 Energy            -1729.973711712628 (opt)
!MCSCF STATE 2.2 Energy            -1729.951339366952

   1  FE     26.00    0.123433343    0.000000000    0.466637943
   2  N       7.00    0.798099079    0.000000000    3.961209142
   3  O       8.00    0.119113397    0.000000000    6.130120171
   4  C       6.00    3.907206777    0.000000000   -0.834771680
   5  O       8.00    5.891051681    0.000000000   -1.523104539
   6  C       6.00   -2.091713353   -2.480213594   -1.457043497
   7  C       6.00   -2.091713353    2.480213594   -1.457043497
   8  O       8.00   -3.327739730   -3.768140342   -2.632565064
   9  O       8.00   -3.327739730    3.768140342   -2.632565064


== CASSCF D0 ==
!MCSCF STATE 1.1 Energy            -1730.035557829641
!MCSCF STATE 2.1 Energy            -1730.019712230712
!MCSCF STATE 1.2 Energy            -1730.074805310643 (opt)
!MCSCF STATE 2.2 Energy            -1730.004886507394

   1  FE     26.00    0.187714015    0.000000000    0.725973912
   2  N       7.00   -0.052689291    0.000000000    3.964657992
   3  O       8.00   -0.292048538    0.000000000    6.180473532
   4  C       6.00    3.746662950    0.000000000   -1.480569074
   5  O       8.00    5.603785236    0.000000000   -2.412137105
   6  C       6.00   -1.776310055   -2.976010749   -1.334493722
```

7	C	6.00	-1.776310055	2.976010749	-1.334493722
8	O	8.00	-2.820403077	-4.582651913	-2.144268949
9	O	8.00	-2.820403077	4.582651913	-2.144268949

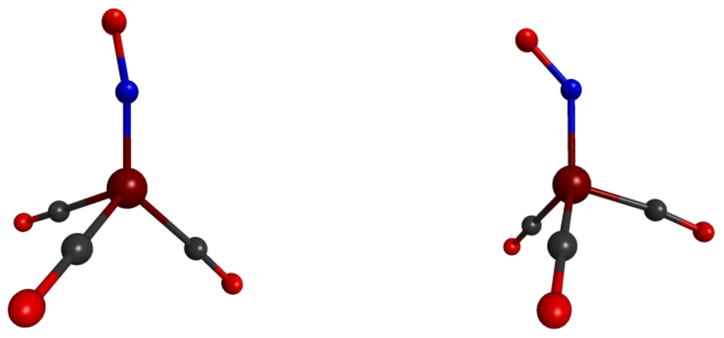

Figure S18. PBE/def2-TZVPP' calculated equilibrium structure in the D_0 state (left) and the T_1 state (right).

13. References

[1] R. K. Bowman and J. S. Johnson, *Org. Lett.* 2006, **8**, 573-576.

[2] H. Braun and G. Huber, *Tetrahedron Lett.* 1976, **17**, 2121-2124.

[3] D. Jacoby, J. P. Celerier, G. Haviari, H. Petit and G. Lhommet, *Synthesis* 1992, **9**, 884-887.

[4] P. Gopinath and S. Chandrasekaran, *J. Org. Chem.*, 2011, **76**, 700-703.

[5] P. Huang, R. Zhang, Y. Liang abd D. Dong, *Org. Lett*. 2012, **14**, 5196-5199.

[6] O. Itoh, N. Yamamoto, K. Nakano, T. Sugita and K. Ichikawa, *Bull. Chem. Soc. Jpn*. 1975, **48**, 3698-370.

[7] H. Murakami, Y. Matsui, F. Ozawa and M. Yoshifuji, *J. Organomet. Chem.* 2006, **691**, 3151-3156.

[8] E. G. Harris and R. G. Fayter, Jr., National Distillers and Chemical Corp., USA . 1987, pp. 11 pp. Cont.-in-part of U.S. 14,515,978.

[9] E. Baciocchi and R. Ruzziconi, *J. Org. Chem*. 1991, **56**, 4772-4778.

[10] M. Lau, M. Sher, A. Villinger, C. Fischer and P. Langer, *Eur. J. Org. Chem*. 2010, 3743-3753.

[11] B. M. Casey, C. A. Eakin, J. Jiao, D. V. Sadasivam and R. A. Flowers, *Tetrahedron* 2009, **65**, 10762-10768.
